# Regional Insect Inventories Require Long Time, Extensive Spatial Sampling and Good Will

**DOI:** 10.1371/journal.pone.0062118

**Published:** 2013-04-22

**Authors:** Simone Fattorini

**Affiliations:** 1 Azorean Biodiversity Group and Platform for Enhancing Ecological Research and Sustainability, Departamento de Ciências Agrárias, Universidade dos Açores, Angra do Heroísmo, Portugal; 2 Water Ecology Team, Department of Biotechnology and Biosciences, University of Milano Bicocca, Milan, Italy; University of California, Berkeley, United States of America

## Abstract

Understanding how faunistic knowledge develops is of paramount importance to correctly evaluate completeness of insect inventories and to plan future research at regional scale, yet this is an unexplored issue. Aim of this paper was to investigate the processes that lead to a complete species inventory at a regional level for a beetle family. The tenebionid beetles of Latium region (Italy) were analysed as a case study representative of general situations. A comprehensive faunistic database including 3,561 records spanning from 1871 to 2010 was realized examining 25,349 museum specimens and published data. Accumulation curves and non-parametric estimators of species richness were applied to model increase in faunistic knowledge over time, through space and by collectors’ number. Long time, large spatial extent and contribution of many collectors were needed to obtain a reliable species inventory. Massive sampling was not effective in recovering more species. Amateur naturalists (here called parafaunists) were more efficient collectors than professional entomologists. Museum materials collected by parafaunists over long periods and large spatial extent resulted to be a cost effective source of faunistic information with small number of collected individuals. It is therefore important to valuate and facilitate the work of parafaunists as already suggested for parataxonomists. By contrast, massive collections by standardized techniques for ecological research seem to be of scarce utility in improving faunistic knowledge, but their value for faunistic studies may be enhanced if they are conducted in poorly surveyed areas.

## Introduction

The vast majority of living animal species are insects [1]. Insect species loss is impressive, with estimated 44,000 insect extinctions in the last 600 years [2]. In spite of the recognized importance of conserving insects, studies in insect conservation are hampered by various kinds of impediments, the most important of which are the Linnean and Wallacean shortfalls [3]. Only a small fraction of the extant species is known (the Linnean shortfall) and data on insect distribution are usually sparse and, for most geographical regions, definitively poor (the Wallacean shortfall). Even groups that may appear taxonomically well known may be still far from a complete species enumeration, especially from regions which have been poorly surveyed [4].

A number of studies have investigated how species descriptions accumulate over time in order to forecast the probable number of still undescribed species at local to global scale (see [4]–[6] for examples and reviews). However, these studies did not explore “the way” taxonomic and faunistic knowledge is acquired. Understanding the relationship between collecting effort and number of species accumulated gives formality to faunistic studies, provides a planning tool for collecting expeditions, and is a predictive tool for biodiversity and conservation studies [7]. Thus, understanding how taxonomic and faunistic knowledge develops is of paramount importance to correctly evaluate completeness of insect inventories and to plan future research. In particular, there is a need for reliable approaches to obtain accurate assessments of species richness at regional scale because biodiversity policies and conservation efforts are increasingly focusing on the landscape, regional and country level, rather than on the local focus [8], [9].

In this paper, I used a comprehensive faunistic database to investigate the processes that lead to a complete species inventory at a regional level. For this, I used the tenebrionid beetles (Coleoptera Tenebrionidae) of Latium (Italy). This choice was driven by various reasons. First, tenebrionids are an example of an insect group that is well investigated, but not particularly favoured (compared with other, most popular insect groups, such as dragonflies, butterflies, ground beetles, scarab beetles, longhorn beetles, etc., see Supporting Information S1). Thus, they can be considered indicative of a relatively well sampled group. Second, tenebrionids are collected very frequently (also by amateur entomologists), but rarely studied. Thus, as for many other insect groups, there is an immense amount of tenebrionid specimens hidden in private and public collections but few published records. Third, Latium is a region of high conservation concern, because it is placed in the centre of the Mediterranean hotspot [10] and includes several areas that present very high values of species richness and percentages of Italian endemics [11]. Thus there is a strong interest in conserving insect diversity in this region.

I used comprehensive collection data (locality, date of collection, and name of collectors of more than 26,700 specimens) to explore how our knowledge of regional diversity (specie richness) increases with time, geographical extent and entomological effort. Understanding these dynamics will be useful to give some prescriptions to how maximize cost effective faunistic research.

## Methods

### Study Area

Latium is situated in the central part of mainland Italy. It comprises a land area of 17,200 km^2^, which is approximatively divided into 210 10×10 km cells in the UTM system. Most of the area is flat and hilly, with relatively small mountains (maxium elevation: 2458 m) of both volcanic and calcareous origin. The coast of Latium is mainly composed of sandy beaches. The central section of the region is occupied by a vast alluvial plain surrounding the city of Rome (about 3 million inhabitants). The southern districts of the region are characterized by flatlands where there are some relics of a swampy area that was largely reclaimed between 1930 and 1940. Along the coasts, temperatures are comprised between 9–10°C in January and 24–25°C in July, whereas in the inner (mountaneous) areas temperatures may be below 0°C in January (−3°C on Mt Terminillo) and below 20°C in July. A detailed description of the study area can be found in [12].

### Data Sources

I compiled 3,561 tenebrionid records from Latium, from which 84 native species are currently known (note that one ‘record’ refers to a unique combination of species, place, altitude, year and source, but may involve from one to several hundreds of specimens). Data originate from museum and private collections (25 collections), publications (334 scrutinized references) and unpublished lists, for a total of 26,743 specimens (25,349 specimens directly examined, plus literature data for 1,394 specimens). The following amateur entomologists allowed me to examine their personal collections: R. Antonelli, A. Cotta’s heirs, E. Migliaccio, P. Maltzeff, R. Pace, U. Pessolano, and G. Di Giulio (now incorporated in my personal collection). P. Leo kindly provided me with unpublished data. A. Vigna Taglianti (Sapienza University of Roma), C. Manicastri (Zoological Museum of Rome) and G. Carpaneto (Roma III University) allowed me to examine the public collections in their care.

Sample sites were georeferenced (latitude and longitude decimal degrees) with the maximum precision allowed by the original datum using digital topographic maps. Then, each point record was assigned to a 10×10 km grid cell using the UTM system. A simplified version of the original database is provided as Supporting Information S2.

Tenebrionids as a whole are an ecologically very diversified group of mainly detritivorous animals, which can be found in virtually all the major environments of the region, including sandy shores, maquis, oak forests, beech forests, high altitude rocky areas, ruderal sites, and wet areas. All these environments have been repeatedly sampled for tenebrionids. Although during the past century the landscape of the region has been affected by increased anthropization, this did not lead to tenebrionid species extinction.

Tenebrionids include certain cosmopolitan species, which are associated with stored food. Entomologists rarely collect these species because they are common pests and their occurrence was not considered in the analyses.

Tenebrionid richness in Latium is very high, accounting for about 49% of the tenebrionid richness of the entire mainland Italy (172 species [13]). Mainland Italy is the third richest country in Europe for tenebrionids: the richest areas are Spain (with 557 species) and mainland Greece (205 species) [13].

### Species Richness Estimates

There are many techniques to estimate species richness through repeated samples and accumulation curves [14], [15]. However, comparative studies have indicated that results may vary considerably depending upon different attributes of the data (e.g. [6], [16], [17]). In this study, I choose to concentrate on measures that are commonly used at local scale sampling, but that appeared also promising for applications at regional scales (see [6], [18]). I used the software EstimateS (version 8.2.0 [19]) to calculate the following non-parametric estimators: Chao 1, Chao 2, Abundance-based coverage estimator (ACE), Incidence-based coverage estimator (ICE), first- and second order jackknife (Jack 1 and Jack 2, respectively) and bootstrap (Boot). Formulas and details about the properties of these estimators can be found in [19]–[21].

The same software was also used to generate sample-based rarefaction (species accumulation) curves using the analytical formulas of Colwell et al. [14]. These curves, also known as Mao Tau curves, were then fitted with two parametric models: Clench and exponential [7]. I used an iterative algorithm (the Levenberg-Marquardt method in Statistica 6.0 [22]) to fit each function to the data (see equations in [7]), and then calculated the asymptote value from the so-obtained parameters as described in [23]. For comparison purposes, a Michaelis Menten asymptote with two different procedures (MMRuns and MMMeans) was also calculated with the software EstimateS [19]. In all cases, 100 randomizations were applied.

In addition to the aforementioned estimators, I also applied two methods (known as F3 and F5) that were specifically designed and recommended for estimating regional diversity in unsampled habitats [24] but which have been rarely used [6]. F3 and F5 are asymptotic methods forced through the point (1,1: one species at a sample size of one individual), but differ for the curvature (see [24] for details). To calculate F3 and F5 I used the software Ws2 m [25] with 100 iterations of sample shuffling. Individuals were alternatively not shuffled or reshuffled making samples exchangeable. Finally, I also computed Clench and exponential asymptotes using a different approach based on pure-birth stochastic processes and which takes into account non constancy of error variance and autocorrelation between observations [26]. For this, I used the Species Accumulation Functions freeware [26], which generates improved model parameters by likelihood non-linear regression functions.

### Analyses

I constructed separate datasets to explore different ways of species accumulation over time, spatial extent and collector intensity.

Species accumulation over time was first analysed using a matrix of number of individuals collected for each species in each year from 1871 to 2010. In this analysis, I constructed an accumulation curve using the chronological ordering of years to investigate temporal growth of faunistic knowledge. This temporal accumulation curve may be viewed as analogous to a “rate of discovery” curve [27] and reflects differences in sampling intensity through time. To remove biases introduced by inconsistencies and discontinuities in faunistic effort, I used a sample-based smoothed curves as recommended by [16]. This way, I obtained “ideal” curves assuming equal sampling intensity through years. Because this year-by-year approach would fragment excessively the data, I also performed analogous analyses grouping years into decades. In both cases, the datasets comprised 82 species.

Species accumulation by geographical extent was analysed using UTM cells as sampling units. In this analysis, I used a dataset including two additional species, for which there was a detailed geographical datum but no information of collecting date. For this dataset I used only smoothed curves to remove any spatial influence of UTM cells.

Finally, I constructed a matrix of number of individuals collected for each species by each of 232 collectors recorded in the database. In these analyses, specimens collected by more than one entomologist were considered as if they were collected by a single collector. This dataset included 80 species, because for four species there is no information about collector. For this dataset I used only smoothed curves because any ordering of collectors would be arbitrary. I also analysed separately smoothed curves for professional and amateur entomologists. I considered as professional entomologists: (1) people employed in universities, museum, research institutes, natural parks, etc.; (2) students who collected insects during their degree or thesis work; (3) insect sellers; (4) nobles who dedicated their personal funds to collect beetles and maintain museums. All other collectors were considered as amateurs. Because the two datasets differed systematically in the mean number of individuals per collector, I used individual based rarefaction (Coleman) curves (see [16] for the rationale).

Some temporal turnover might be expected for cosmopolitan species, but not for the other species. It is very unlikely that the region has acquired “new” species from adjacent areas during the study period (from 1871 to 2010). So, any addition of new species in the accumulation curves should be viewed as a collection of previously undetected species. There is evidence that some species experienced a range contraction, but information contained in the database indicates that no species disappeared from the study area.

## Results

### Species Accumulation Over Time

**Figure 1:**
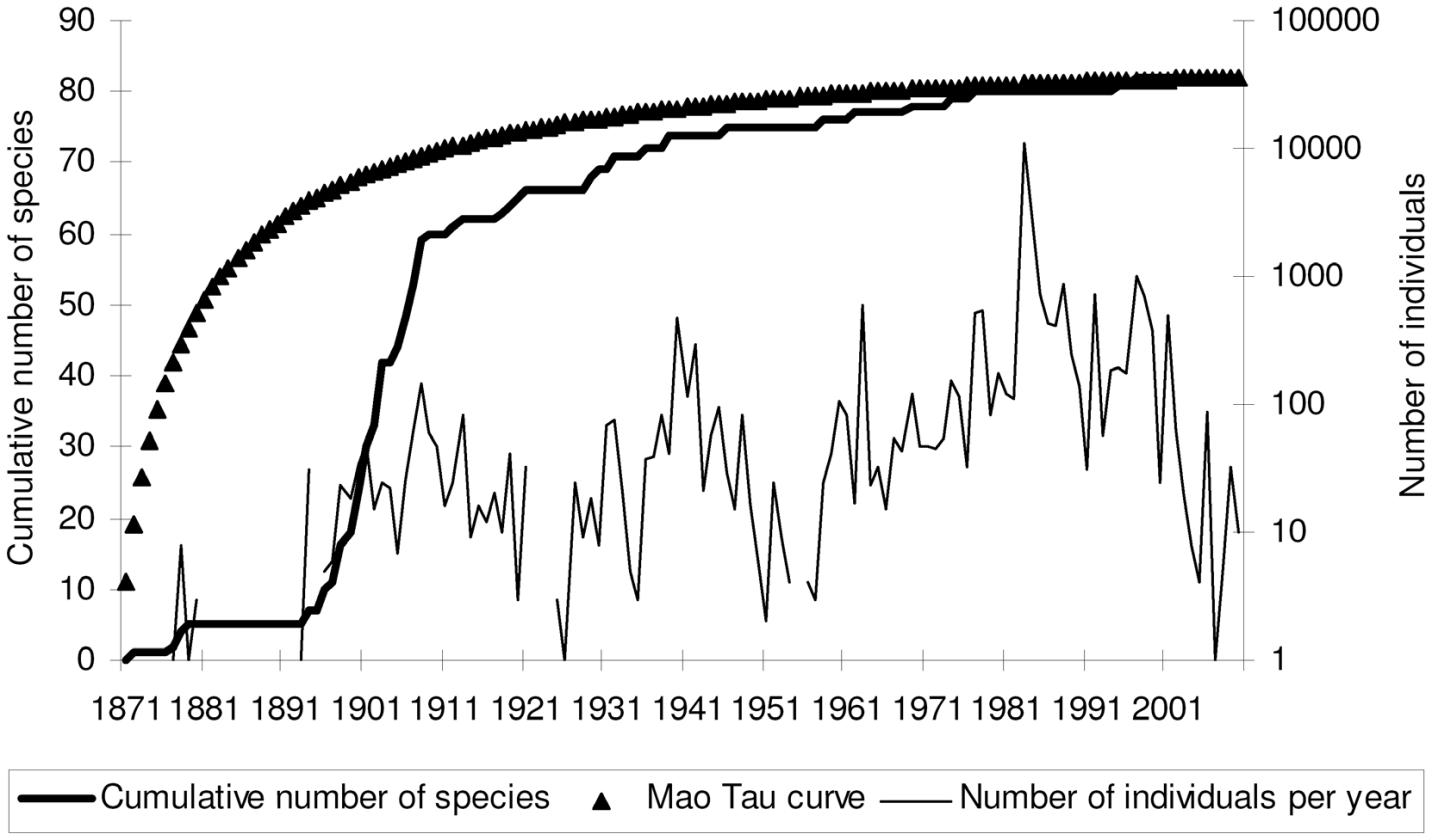
Accumulation curve and number of collected individuals per year for the tenebrionid beetles of Latium (Italy).

The cumulative number of species shows a sigmoid, three-phasic pattern (Fig. 1). In a first phase, species accumulate with increasing rate. Then, they tend to increase more linearly. Finally, a plateau is reached at 82 known species. The first phase (which characterizes the years 1871–1910) is well modelled by an exponential fit (*y* = 0.884*e* ^0.106*x*^; *R* ^2^ = 0.917) whereas the second phase (1911–1980) is well fitted by a linear model (*y* = 0.268*x* +62.827; *R* ^2^ = 0.922) with a mean rate of about 0.3 new species per year. A 90% of total known species richness (i.e. about 74 species) is reached in 1939, i.e. after 69 years of sampling (and 1309 sampled individuals). However, use of a smoothed curve (MaoTau), indicates that about 50 years (but with more than 9000 individuals) should be needed to reach 90% of known richness. This indicates that sampling effort was very uneven among years. Number of sampled individuals varied greatly among years, with a peak in the 1980 s. As expected, there is a correlation between number of species and number of individuals collected each year (Spearman rank correlation *r*_s = _0.311, *P*<0.0001, *N = *140), but data show that the maximum annual number of species was obtained with about 1000 sampled individuals, whereas even a tenfold sampling effort did not allow an increase in the number species per year (Fig. S1 in Supporting Information S3). A plot of cumulative number of species against collected individuals, indicates that about 1300 individuals were needed to accumulate 95% of species (Fig. S2 in Supporting Information S3).

The exponential model of species accumulation [*y* = (6.474/0.082)(1−*e* ^−0.0822*x*^)] applied to smoothed data explained a good percentage of variance (*R* ^2^ = 0.943), but the asymptote (78.7) is lower than the total known richness. A Clench function [*y* = (11.037*x*)/(1+0.128*x*)] fitted the data in a virtually perfect way (*R* ^2^ = 0.999), predicting a total species richness of 86.3 species. Use of a method that maximizes the likelihood function, gave slightly different values [*y* = (5.592/−0.067)(1−*e* ^−0.067*x*^), with 82.96 predicted species for the exponential model; *y* = (11.044*x*)/(1+0.128*x*), with 86.42 predicted species for the Clench model, which was identified as the best model].

Most of statistical estimators of true species richness gave values between 82.6 and 86 species, which means that known species are at least 95% of total richness, and possibly 100% (tab. 1). However, F3 and F5 gave higher estimates (96 species).

**Table 1:** Species richness of tenebrionid beetles in Latium (Central Italy) estimated by various non parametric estimators for different measures of sampling efforts (number of years, number of decades, number of UTM 10×10 cells and number of collectors).

Estimator	Years	Decades	UTM cells	Collectors
ACE	83.34	83.34	86.72	81.30
ICE	83.64	83.54	86.31	82.84
Chao1	82.6	82.6	85.20	81.00
Chao2	83.19	82.43	85.86	81.49
Jack 1	85.97	85.71	89.96	85.97
Jack 2	86.00	79.65	89.02	83.04
Bootstrap	84.47	85.08	87.52	83.83
MMRuns	84.41	91.3	89.45	84.80
MMMeans	85.55	90.43	90.48	85.31
F3	96. 03	92.72	106.39	100.60
F5	96.33	92.75	109.37	104.34

ACE: Abundance-based coverage estimator; ICE: Incidence-based coverage estimator; Chao 1: Abundance-based estimator of species richness; Chao 2: Incidence-based estimator of species richness; Jack 1: First-order jackknife richness estimator; Jack 2: Second-order jackknife richness estimator; Bootstrap: Bootstrap richness estimator; MMRuns: Michaelis–Menten nonparametric estimator with values averaged over randomizations; MMMeans: Michaelis-Menten richness estimator computed once for Mao Tau species accumulation curve; F3 Extrapolation nonparametric estimator 3; F5 Extrapolation nonparametric estimator 5.

**Figure 2:**
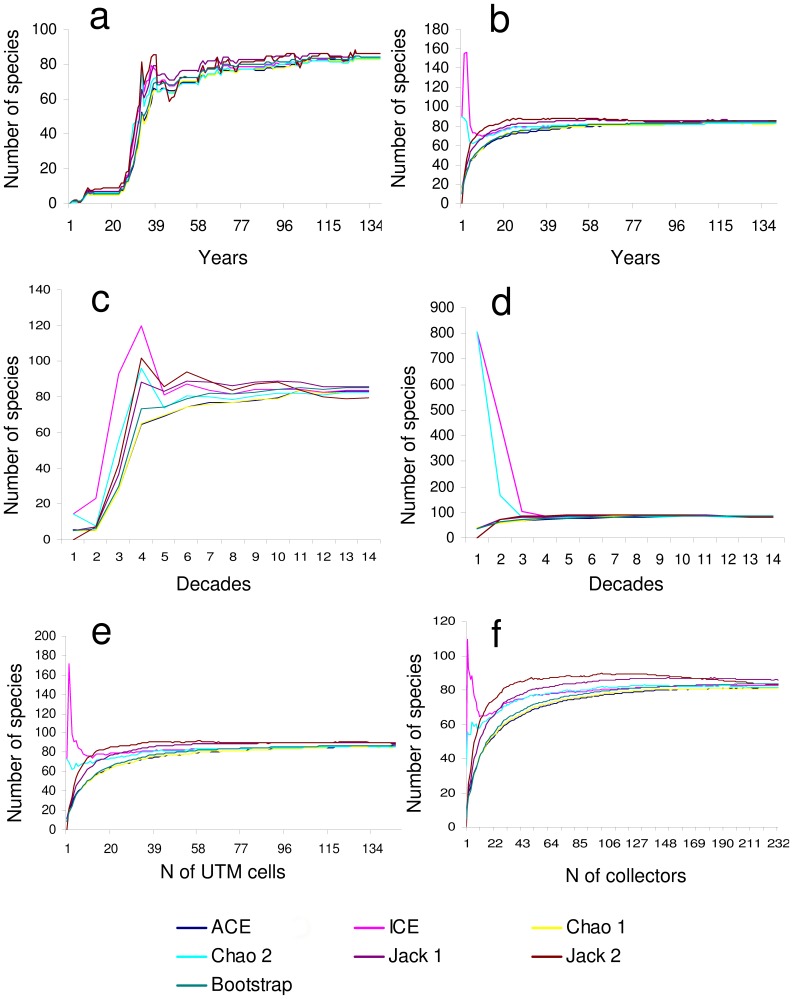
Behaviour of non parametric species richness estimators. Estimates obtained for species sampled year-by-year in the chronological order (a), with the chronological order removed by randomizing years (b), decade-by-decade in the chronological order (c), with the chronological order removed by randomizing decades(d), using different numbers of sampled cells (e), and using different numbers of collectors (f).

If the original temporal trend is used without randomisation, ACE, ICE, Chao1 and Chao2 estimators were able to predict 90% of observed total richness between the years 1929–1930, when known species were 68–69 (Fig. 2a). However, the first- and second-order jackknife estimators predicted 90% of observed total richness already for the data available to 1905–1910, and bootstrap for the data available to 1929 (Fig. 2a).

If exponential and Clench functions are applied to the accumulation curve with the original time trend, the exponential model predict 82.27 species [*y* = (3.430/0.0417)(1−*e* ^−0.0417*x*^), best model] and the Clench function 105.48 species [*y* = (2.977*x*)/(1+0.028*x*)].

An analysis of the ability of each estimator to predict known species richness at different cumulative sampling efforts (Fig. 2b), indicates that first- and second-order jackknife estimators were those which predicted known species richness with smaller collection efforts (about 17–24 years assuming a uniform annual sampling effort). ICE e Chao2 had an erratic behaviour at low sampling efforts, but all estimators tended to stabilize at their final values with a sampling effort of about 80–100 years assuming a uniform yearly sampling effort. The two Michaelis Menten estimators (MMRuns and MMMeans) predicted respectively 84.41 and 85.55 species. A value of 82 species was reached after 20 and 27 years respectively.

A temporal analysis based on decades gave very similar outcomes. The cumulative number of species per decade showed a sigmoid pattern, with a first phase where species accumulated rapidly (1871–1910), a second phase where species accumulated in a more linear way (1911–1980), and a final plateau (from 1981) (Fig. S3 in Supporting Information S3). A 90% of total known species richness was reached in the 1940 s. However, use of a smoothed curve showed that about 50 years (with 9576 individuals) would be needed to reach 90% of known richness, which indicates that sampling effort was very uneven among decades, with a peak in the 1980 s (some 17000 individuals). However, “well sampled” decades (with about 1000 sampled individuals) were also the 1930 s, 1940 s, 1960 s, and 2000 s; particularly well sampled were the 1970 s (about 1750 individuals) and the 1990 s (3500 individuals). Number of species and number of individuals collected in each decade were positively correlated (*r*_s = _0.884, *P*<0.0001, *N* = 14), but a high number of species (50–60) was obtained with about 1000 sampled individuals (Fig. S4 in Supporting Information S3). About 1800 individuals were needed to accumulate 95% of species (Fig. S5 in Supporting Information S3).

The exponential model of species accumulation [*y* = (44.251/0.556)(1−*e* ^−0.556*x*^)] applied to smoothed data explained a very good percentage of variance (*R* ^2^ = 0.966), but the asymptote (79.63) is lower than total known richness. A Clench function [*y* = (69.541*x*)/(1+0.771*x*)] fitted the data in a virtually perfect way (*R* ^2^ = 0.9997), predicting a total species richness of 90.23 species. Use of a method that maximizes the likelihood function, gave slightly different values [*y* = (39.767/0.480)(1−*e* ^−0.480*x*^), with 82.85 predicted species for the exponential model; *y* = (69.566*x*)/(1+0.775*x*), with 89.76 predicted species for the Clench model, which was identified as the best model].

Statistical estimators of true species richness gave values between 80 and 85 species, which means that known species are at least 95% of total richness, and possibly 100% (tab. 1).

If the original temporal trend is preserved, ACE and Chao1 predicted 90% of observed total richness in the 1920 s, when known species were 69 (Fig. 2c). For the same decade ICE predicted 88 species, Chao2 81 species, and bootstrap 79 species. The first- and second-order jackknife estimators gave even higher predicted values (89 and 94 respectively). ICE e Chao2 tended to overestimate richness at low sampling efforts, and all estimators tended to stabilize at their final values with a sampling effort of about 100 years (15,000–20,000 individuals) if sampling effort per decade is assumed to be uniform (Fig. 2d). Michaelis Menten estimators predicted 91 (MMRuns) and 90 (MMMeans) species. Use of a method that maximizes the likelihood function identified the exponential model [*y* = (24.883/0.300)(1−*e* ^−0.300*x*^), with 83.02 predicted species] as better than the Clench one [*y* = (17.733*x*)/(1+0.132*x*), with 134.32 predicted species].

### Species Accumulation Through Space

**Figure 3:**
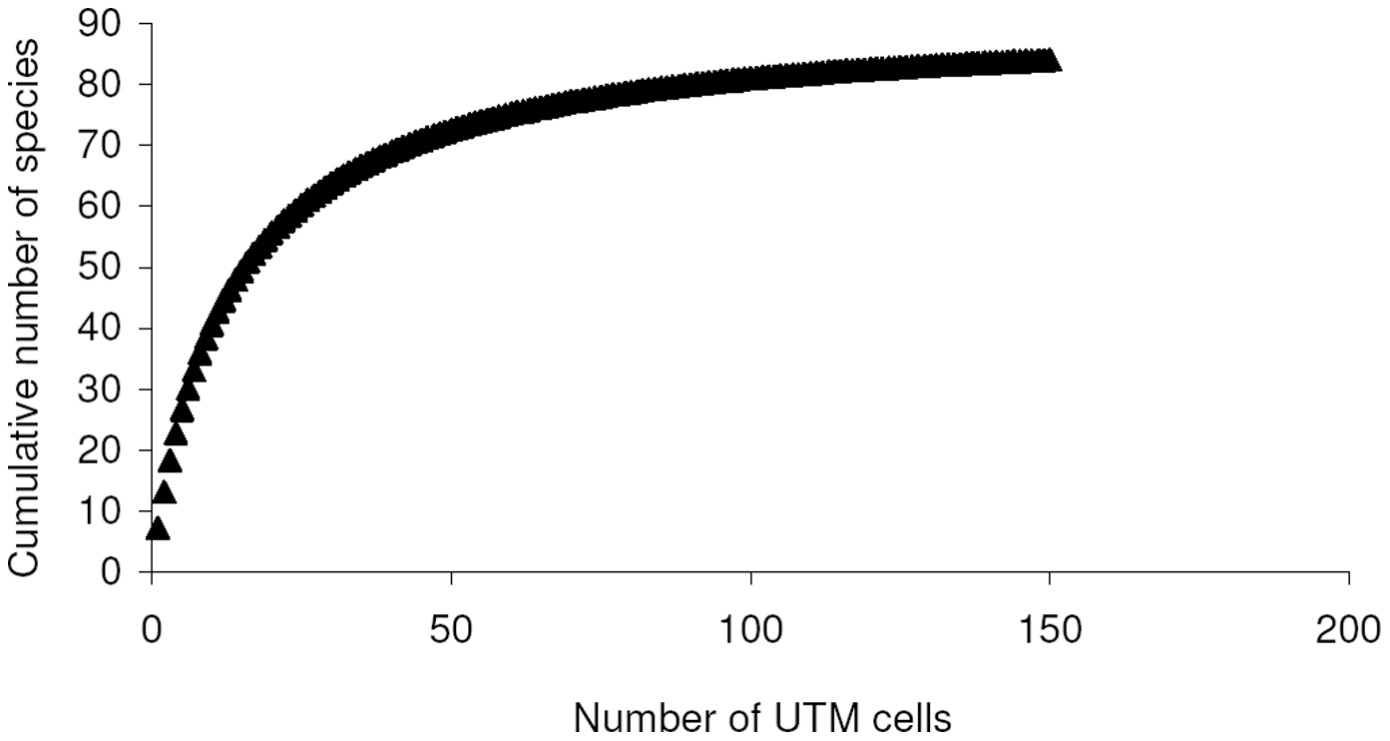
Species accumulation curve constructed by adding UTM 10 ×10 cells.

Use of UTM cells allowed the analysis of a dataset comprising 84 species, with two species for which a geographical datum without collecting date was available. For this dataset I used only smoothed curves to remove any spatial influence on the ordering of UTM cells. A MaoTau curve indicates that a 90% of total known species richness (i.e. about 76 species) is reached after about 64 sampled UTM cells (Fig. 3), with a cumulative number of about 11720 individuals. However, a Coleman (individual based) curve was able to predict 90% of total known richness with fewer than 5500 individuals (Fig. S6 in Supporting Information S3), which indicates that unequal sampling intensity among UTM cells had a serious effect on accumulation curves. Number of sampled individuals varied among UTM cells from 1 to 7846 (median value: 13 individuals per cell).

Number of species and number of individuals collected in each UTM cell were correlated (*r*_s_ = 0.553, *P*<0.0001, *N* = 150), but the maximum number of species per UTM cell (46 species) was obtained with about 1000 sampled individuals, whereas even a sevenfold sampling effort did not allow the addiction of more species (Fig. S7 in Supporting Information S3).

The exponential model of species accumulation [*y* = (4.545/0.056)(1−*e* ^−0.056*x*^)] applied to smoothed data explained a good percentage of variance (*R* ^2^ = 0.969), but the asymptote (81.3) is lower than total richness. A Clench function [*y* = (7.206*x*)/(1+0.0791*x*)] fitted the data in a virtually perfect way (*R* ^2^ = 0.9997), predicting a total species richness of 91.22 species. Use of a method that maximizes the likelihood function, gave slightly different values [*y* = (4.623/0.054)(1−*e* ^−0.054*x*^), with 85.00 predicted species for the exponential model; *y* = (7.157*x*)/(1+0.078*x*), with 91.22 predicted species for the Clench model, which was identified as the best model].

Most of statistical estimators of true species richness gave values between 85 and 90 species, which means that known species are at least 94% of total richness (tab. 1). However, F3 and F5 gave higher estimates (106–109 species). ICE e Chao2 had an erratic behaviour at low sampling efforts, but all estimators tended to stabilize at their final values with a sampling effort of about 120 UTM cells (Fig. 2e).

### Species accumulation by collectors

**Figure 4:**
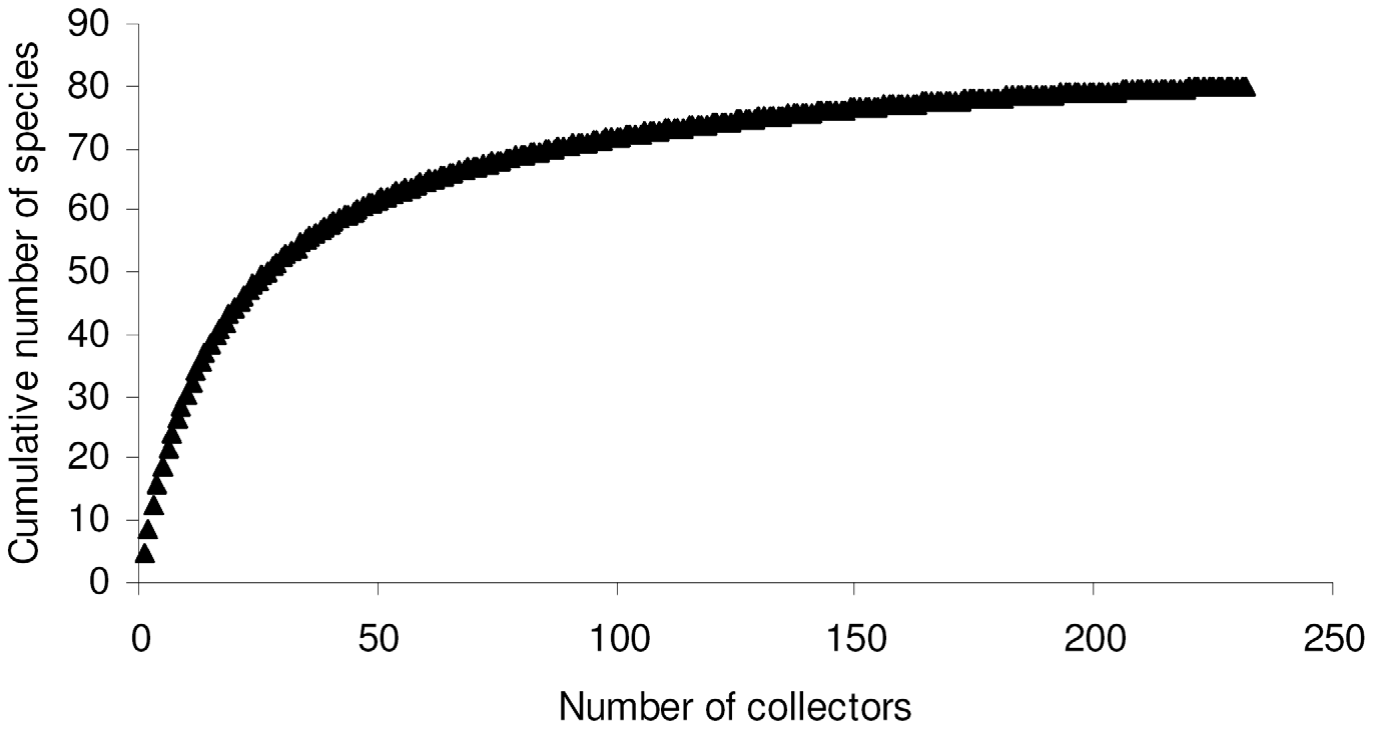
Species accumulation curve constructed by adding collectors.

This dataset allowed the inclusion of 80 species for which collectors were known. For this dataset I used only smoothed curves because there is no obvious way to order collectors into a logical sequence. A MaoTau curve indicates that a 90% of total known species richness (i.e. 72 species) was reached with about 100 collectors (Fig. 4) and with a cumulative number of about 11000 individuals. However, a Coleman curve was able to predict 90% of total known richness with about 5500 individuals, which indicates that unequal collecting intensity among entomologists had a serious effect on accumulation curves. Number of sampled individuals varied among collectors from 1 to 1304, but median value was only 4 individuals per collector.

Number of species and number of individuals collected by each entomologist were slightly correlated (*r*_s = _0.137, *P = *0.037, *N* = 232), and the maximum number of species per collector (48 species) was obtained with about 300 sampled individuals (Fig. S8 in Supporting Information S3).

Most entomologists collected very few species: out of a total of 237 collectors, 101 (42.6%) collected only one tenebrionid species. Only one entomologist collected more than 40 species, and very few (13) entomologists collected more than 20 species.

The exponential model of species accumulation [*y* = (2.790/0.036)(1−*e* ^−0.036*x*^)] applied to smoothed data explained a good percentage of variance (*R* ^2^ = 0.998), but the asymptote (76.5) is lower than total richness. A Clench function [*y* = (4.420*x*)/(1+0.051*x*)] fitted the data in a virtually perfect way (*R* ^2^ = 1.000), predicting a total species richness of 86.24 species. Use of a method that maximizes the likelihood function, gave slightly different values [*y* = (3.030/0.037)(1−*e* ^−0.037*x*^), with 81.00 predicted species for the exponential model; *y* = (4.310x)/(1+0.050*x*), with 86.62 predicted species for the Clench model, which was identified as the best model].

Most of statistical estimators of true species richness gave values between 81 and 86 species, with F3 and F5 providing higher estimates (100–104 species) (tab. 1). ICE had an erratic behaviour at low sampling efforts, but all estimators tended to stabilize at their final values with a sampling effort of about 180 collectors (Fig. 2f).

**Figure 5:**
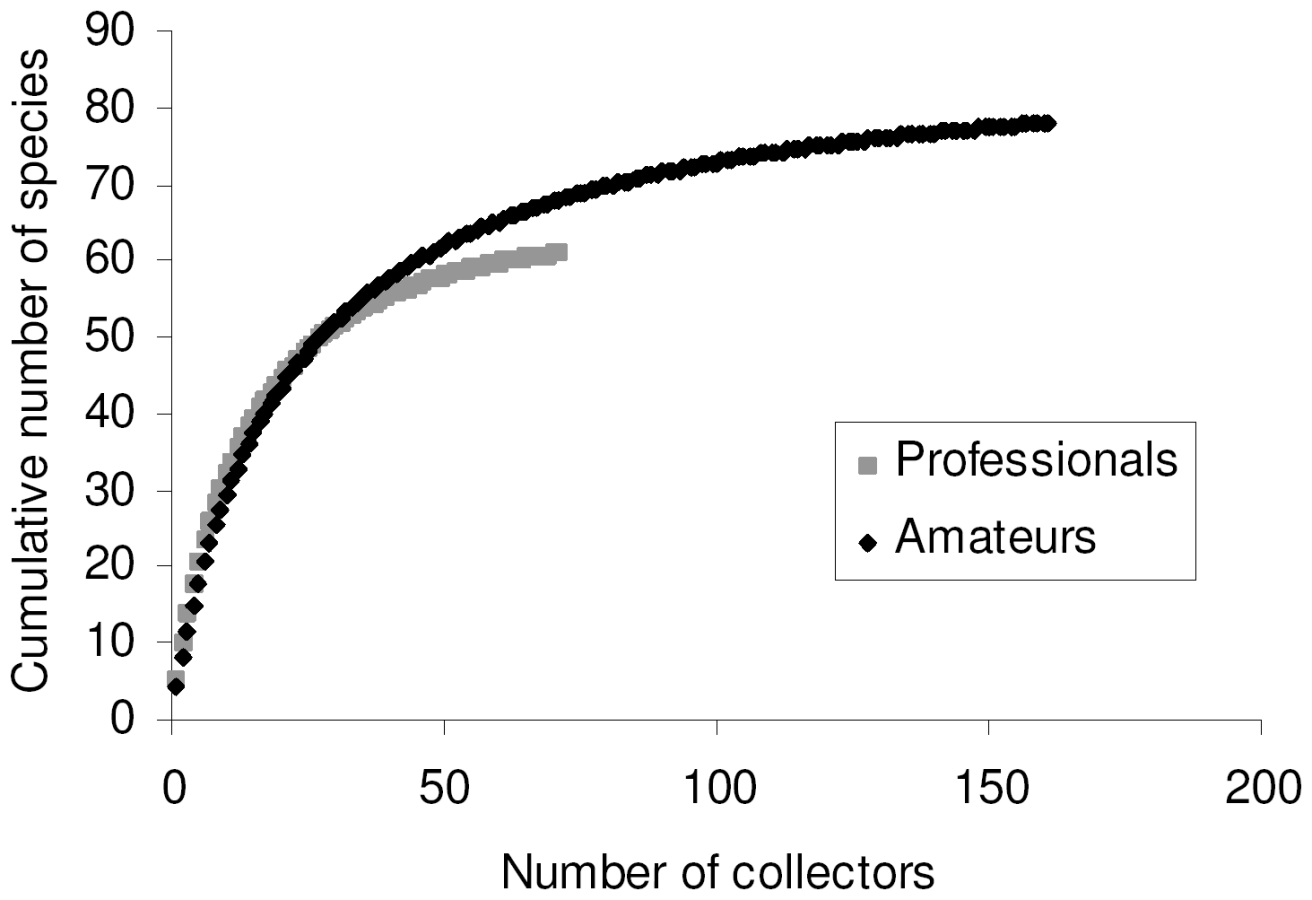
Species accumulation curve constructed by adding number of collectors divided into “professionals” and “amateurs”.

A comparison of rarefaction curves for professional and amateur entomologists indicates that amateurs outperformed greatly (Fig. S9 in Supporting Information S3). First, with a cumulative number of 5000 collected individuals, amateurs collected 78 species, whereas professionals, with 20,000 collected individuals, accumulated 61 species. The two curves also have different shapes. The amateur curve lies above the professional curve and reached 90% of richness with about 1500 individuals. However, a sample based (Mao Tao) curve (Fig. 5) shows that the number of collectors matters. Below 30 collectors the two curves were virtually indistinguishable, but after this value the amateur curve lies above the professional curve. Thus, amateurs allowed a more complete inventory because (1) a single amateur was, on average, able to collect more species with a lower sampling effort (number of collected individuals) and (2) there were more amateur collectors.

## Discussion

Many authors have stressed the importance of considering non-charismatic, little-known taxa in conservation biology [2], [28], [29]. However, conservation studies require adequate information that cannot be available for “excessively” neglected groups. Few arthropod groups in few areas can be considered very well known, such as butterflies in European countries (see [30]), and probably most are very poorly known, with many still undescribed species in the richest areas, such as most tropical arthropods [31]. Yet, there is no doubt that there are many non-charismatic groups for which a valuable amount of data, hidden in museum collections or specialized literature, is available [32], [33]. Moreover, as collection label data are becoming available through collection databasing [34], faunistic assessments, similar to those achieved in this study, might be easily done with other selected insect families in the future.

Tenebrionid beetles certainly qualify for non-charismatic animals, and are not particularly favoured by entomologists, yet they are not among the least investigated insects in Italy, and their level of knowledge can be considered similar to that of most insect groups in relatively well investigated areas. Thus, conclusions reached in this study can be confidently extended to other insect groups in a variety of contexts, although further research on different taxa would provide important comparative results.

The very large sampling effort made through more than a century by hundreds of collectors, who were not specifically interested in tenebrionids, and who used any kind of collecting method, ensure that the data used are not affected by collector preferences for certain biotope, site or species characteristics. In general, museum data are based on specimens collected with a variety of unstandardized methods, which may produce biased abundance distributions. However, such data can be properly analysed with species richness estimation methods, although not all estimators perform equally well [32], [33].

This study was not aimed at evaluating the performance of statistical estimators of species richness, which is considered usually difficult because of the lack of a reference datum (see [17], [35]). In this study, I used estimators to evaluate if the asymptotes of the accumulation curves were not simply due to the lack of adequate sampling, but reflected a real state of affairs. Theoretically, only those datasets in which the observed species accumulation curves reached an asymptote should be used to draw conclusions about the behaviour of the estimators [17]. In general, all curves obtained in this study had a plateau, and most estimators (including asymptotic equations) gave estimates of species richness close to the observed ones, which indicates that the observed plateau is a true asymptote. This consistence allows the possibility of drawing some conclusions about the performance of estimators at lower sampling intensity, i.e. before the asymptote is reached. ICE and Chao2 tended to have an erratic behaviour and to overestimate richness at low sampling efforts, as also noticed in other studies (e.g. [17]). The two estimators F3 and F5 consistently overestimated species richness, especially when using reduced values of known species richness, thus performing poorly, as found in previous work [6]. In general, jackknife estimators appeared particularly efficient in predicting true species richness at low sampling intensity. More in general, the second-order jackknife estimator was also found the least biased estimator in a comparative studies for museum data [32], [33].

Questions regularly asked in biodiversity studies include how many species are in an area, and how much effort is required to predict total species richness [16]. Appropriate species accumulation curves should provide answers to these questions. In this study, I constructed accumulation curves to study how species accumulate with increasing time, spatial coverage and collectors. Patterns of species accumulation over time indicate that a complete faunal inventory required a long time: about a century of insect collecting was needed before the accumulation curve reached a plateau. Development of faunistic knowledge proceeded initially slowly, probably because of the low number of entomologists and poor taxonomic knowledge that characterized the end of the 19th century. Then, with the beginning of the 20th century, an increase in the interest for insect taxonomy coupled with technical progresses (like a wider use of high quality microscopes) determined a rapid increase in faunistic knowledge. After the first decade of the 20th century, development of faunistic knowledge proceeded with a constant rate until the 80s, when known species richness reached a plateau. Although this overall pattern is strongly conditioned by historical factors, use of a smoothed curve that removed the temporal sequence, still indicates that a plateau may be reached only after about 100 years. Thus, the main message of temporal analysis is that a regional insect inventory needs long time, and that this is only in part a consequence of inadequate sampling in the first phases. Even assuming a uniform sampling, an adequate faunistic knowledge would request some 50 years, and even the best estimators need several decades to predict species richness with sufficient precision. This indicates that there is high turnover among species collected in each year, even when large numbers of beetles are collected. A possible explanation of this pattern can be the presence of many rare species, which are seldom collected even when large samples are taken.

In fact, analyses based on number of collected individuals indicate that number of species does not increase linearly with number of collected individuals, and an increased sampling effort, over certain threshold values, does not increase sensibly known species richness. Thus, increasing sampling intensity is not a good way to increase faunistic knowledge at a regional scale. Reasons for this relationship between collected individuals and number of species cannot be deduced directly from the pattern, yet some inference is possible taking into account the way insects were collected. Large numbers of collected individuals typically result from quantitative sampling conducted by using standard methods, such as pitfall trapping or examination of fixed volumes or mass of substrate [36]. These techniques may allow massive collection of beetles, but typically from a few localized sampling sites, and therefore contribute marginally to regional knowledge, especially because of the presence of many rare and restricted species. Examination of label data confirmed that massive captures of tenebrionids in Latium originated from few sites where pitfall traps and sand sieving were used.

Analyses based on grid cells indicate that an adequate faunistic knowledge requires a relatively large spatial extent, because of non-random spatial distribution of the species [37]. Also in this case, massive sampling does not allow the collection of a substantially larger number of species, after a “threshold” value is reached. To obtain a reliable faunal inventory, it is more important to extend the geographic coverage than intensifying local sampling. Although massive sampling is not particularly useful to increase faunistic knowledge, it is the unavoidable by-product of standardized techniques, such as the use pitfall traps, which are known to collect insects massively and to kill non target species [38]. To maximize the contribution of such massive sampling to faunistic knowledge, ecologists should try to perform quantitative researches in areas that are faunistically poorly known. This way, a by-product of ecological research would be an extent of the geographical coverage of species records. Moreover, it would be important that material from massive sampling efforts, including the non target species, should be adequately preserved (e.g. in alcohol or at low temperature) and made available to other entomologists and taxonomists for their studies.

Species accumulation by collectors showed that amateurs contributed to faunistic knowledge more than professional entomologists. The role of amateur natural historians in biological research has become into focus with decreasing interest of professional biologists in taxonomic studies [39]. The importance of non-professional biologists is now widely recognized in taxonomic research [40], [41] and biodiversity monitoring [42] for which the term “parataxonomist” is frequently used to indicate retired professional taxonomists, part-time amateur natural historians, and professional practitioners of disciplines related to taxonomy, such environmental sciences [43], [44]. In analogy with this term, I propose “parafaunists” to indicate people who contribute to faunistic knowledge collecting faunistic data for pleasure, during their spare time, and who get their income from other occupations.

In this paper, I divided entomologists into “amateurs” and “professionals”, but another classification might be based on expertise rather than profession. For example, one might compare accumulation curves developed from collections by “specialists” vs. “incidental collectors”. Professionals who made incidental collections during studies of other insects, or during visits from elsewhere, may produce “occasional” records, whereas many amateurs may become specialists and are much more efficient as collectors than professionals who are as yet naïve, sample by fixed protocols, or collect specimens only incidentally. In this approach, specialists could be defined by numbers of species they have collected, e.g. persons who have contributed a substantial number (say more than 30%) of species. In the case of the tenebrionids of Latium, this was not possible because of the very low number of entomologists that collected a relatively large number of species, but might be applicable in other cases.

For the tenebrionids of Latium, the most efficient collector (with 54 collected species) was an amateur entomologist active between 1894 and 1939. The second most efficient collector (40 species) was another amateur operating between 1933 and 1994. Thus, it does not seem that recent improvements in trapping methods and equipment, as well as change in leisure time, familiarity of habitats, and more efficient transportation, made collectors in recent decades better than their predecessors. However, in other circumstances these factors may be important and, coupled with a partial loss of older records (e.g. by deterioration of historical collections), can lead to an overrepresentation of recent records, and this should be taken into account as a possible bias in this type of studies.

This study showed that “parafaunists” had an essential role in generating data for faunistic knowledge. Although parafaunists collect fewer specimens they accumulate more species then professionals, probably because they are typically interested in increasing taxonomic completeness and faunistic coverage of their collections. It is therefore of paramount importance to valuate and facilitate the work of parafaunists by promoting cooperation between professionals and amateurs, as indicated for parataxonomists [39].

### Conclusions

This study demonstrates that, if knowledge of diversity is important, rapid surveys involving few sampling areas are inadequate to obtain a “complete” species inventory of a region. This implies long time, large spatial extent and contribution of many collectors. For these reasons, materials collected by parafaunists, and preserved in their personal collections or in museums, are an extremely useful and a cost effective source of records. By contrast, massive collections seem to be of scarce utility in improving faunistic knowledge. However, because they are the by-product of sampling methods largely used in ecological research, the value of such massive collections for faunistic studies may enhanced if they are conducted in poorly surveyed areas.

## Supporting Information

Supporting Information S1 Preliminary assessment of entomologists’ interest in tenebrionid beetles.
(DOC)

Supporting Information S2 Tenebrionids records from Latium and scrutinized references.
(DOC)

Supporting Information S3 Supporting Figures S1–S9.
(DOC)
