# Supplementary material for: Regional Insect Inventories Require Long Time, Extensive Spatial Sampling and Good Will
Source: PLoS One. 2013 Apr 22;8(4):e62118. doi: 10.1371/journal.pone.0062118 (PMC3632580; doi:10.1371/journal.pone.0062118)
Supplement: Supporting Information S1 — Preliminary assessment of entomologists’ interest in tenebrionid beetles. (DOC) [file pone.0062118.s001.doc]

## **Supporting Information S1**

## Preliminary assessment of entomologists’ interest in tenebrionid beetles

It is virtually impossible to quantify the interest of Italian entomologists in tenebrionid beetles. However, to have an idea of the appeal of these insects in comparison with other groups, I have used taxonomic preferences expressed by entomologists belonging to the Italian Entomological Society (SEI) as given by the last available survey (year 2005). Obviously, not all Italian entomologists are SEI members, but SEI is the largest entomological association in Italy, so their members may be considered a good sample of Italian entomologists. Many members did not declare taxonomic interests, or expressed interests in non-insect arthropod groups (e.g. arachnids, centipedes, crustaceans, etc.). Thus, the actual sample available for the analysis comprised 426 entomologists. Among these, 244 declared an interest in beetles, and 214 expressed a preference for one or more beetle families. The most favoured beetle families were Carabidae (64 entomologists), Scarabaidae (27), Cerambycidae (24) and Curculionidae (21). Ten entomologists were interested in tenebrionids, which ranked 5th (within 23 families for which a preference has been expressed). Because beetles families vary greatly in number of species, a ratio between number of species and number of entomologists can better express intensity of entomologists’ interest. If such ratio is used, tenebrionids ranked 8th. These data indicate that tenebrionids qualify as a group of “intermediate” interest among Italian entomologists, being in the last quartile for number of entomologists, and in the third quartile for the ratio species:entomologists.
