# Supplementary material for: Regional Insect Inventories Require Long Time, Extensive Spatial Sampling and Good Will
Source: PLoS One. 2013 Apr 22;8(4):e62118. doi: 10.1371/journal.pone.0062118 (PMC3632580; doi:10.1371/journal.pone.0062118)
Supplement: Supporting Information S3 — Supporting Figures S1–S9. (DOC) [file pone.0062118.s003.doc]

## **Supporting Information S2**

*Supporting Figures S1-S9*

**Figure S1** Relationship between number of species and number of collected individuals per year. Note that years with large number of collected individuals (>1000) did not add more species than years with smaller number of individuals.

**Figure S2** Cumulative number of species as a function of cumulative number of sampled individuals. The curve “flattened” at about 5000 individuals.

**Figure S3** Accumulation curve and number of collected individuals per decade for the tenebrionid beetles of Latium (Italy).

**Figure S4** Relationship between number of species and number of collected individuals per decade. Note that decades with large number of collected individuals (>1000) did not add more species than decades with smaller number of individuals.

**Figure S5** Cumulative number of species as a function of cumulative number of sampled individuals using decades. The curve “flattened” at about 5000 individuals.

**Figure S6** Species accumulation curve constructed by adding individuals.

**Figure S7** Relationship between number of species and number of collected individuals per sampling cell.

**Figure S8** Relationship between number of species and number of individuals sampled by each collector.

**Figure S9** Species accumulation curve constructed by adding number of individuals taken by collectors divided into “professionals” and “amateurs”.
