# Supplementary material for: Regional Insect Inventories Require Long Time, Extensive Spatial Sampling and Good Will
Source: PLoS One. 2013 Apr 22;8(4):e62118. doi: 10.1371/journal.pone.0062118 (PMC3632580; doi:10.1371/journal.pone.0062118)
Supplement: Supplementary file 3 [file pone.0062118.s002.doc]

Database	Data used for the analyses
Acronyms	Acronyms of collections as used in the Database
Scrutinized	List of scrutinized papers
Photos	Species names and links to photographs

Species name	Year
Accanthopus velikensis	1997
Accanthopus velikensis	1998
Accanthopus velikensis	2001
Accanthopus velikensis	1985
Accanthopus velikensis	1985
Accanthopus velikensis	1986
Accanthopus velikensis	1986
Accanthopus velikensis	1986
Accanthopus velikensis	1986
Accanthopus velikensis	1986
Accanthopus velikensis	1986
Accanthopus velikensis	1986
Accanthopus velikensis	1986
Accanthopus velikensis	1986
Accanthopus velikensis	1986
Accanthopus velikensis	1986
Accanthopus velikensis	1986
Accanthopus velikensis	1986
Accanthopus velikensis	1986
Accanthopus velikensis	1986
Accanthopus velikensis	1986
Accanthopus velikensis	1986
Accanthopus velikensis	1986
Accanthopus velikensis	1986
Accanthopus velikensis	1986
Accanthopus velikensis	1986
Accanthopus velikensis	1986
Accanthopus velikensis	1986
Accanthopus velikensis	1986
Accanthopus velikensis	1986
Accanthopus velikensis	1986
Accanthopus velikensis	1986
Accanthopus velikensis	1986
Accanthopus velikensis	1987
Accanthopus velikensis	1987
Accanthopus velikensis	1987
Accanthopus velikensis	1987
Accanthopus velikensis	1987
Accanthopus velikensis	1987
Accanthopus velikensis	1987
Accanthopus velikensis	1987
Accanthopus velikensis	1987
Accanthopus velikensis	1987
Accanthopus velikensis	1987
Accanthopus velikensis	1987
Accanthopus velikensis	1987
Accanthopus velikensis	1987
Accanthopus velikensis	1999
Accanthopus velikensis	1974
Accanthopus velikensis	1998
Accanthopus velikensis	1992
Accanthopus velikensis	1992

Accanthopus velikensis	1992
Accanthopus velikensis	1992
Accanthopus velikensis	1992
Accanthopus velikensis	1992
Accanthopus velikensis	1992
Accanthopus velikensis	1992
Accanthopus velikensis	1992
Accanthopus velikensis	1988
Accanthopus velikensis	1988
Accanthopus velikensis	1988
Accanthopus velikensis	1988
Accanthopus velikensis	1988
Accanthopus velikensis	1988
Accanthopus velikensis	1988
Accanthopus velikensis	1988
Accanthopus velikensis	1988
Accanthopus velikensis	1988
Accanthopus velikensis	1988
Accanthopus velikensis	1988
Accanthopus velikensis	1961
Accanthopus velikensis	1981
Accanthopus velikensis	1981
Accanthopus velikensis	1981
Accanthopus velikensis	1981
Accanthopus velikensis	1981
Accanthopus velikensis	1981
Accanthopus velikensis	1981
Accanthopus velikensis	1981
Accanthopus velikensis	1981
Accanthopus velikensis	1981
Accanthopus velikensis	1981
Accanthopus velikensis	1981
Accanthopus velikensis	1982
Accanthopus velikensis	1988
Accanthopus velikensis	1988
Accanthopus velikensis	1988
Accanthopus velikensis	1988
Accanthopus velikensis	1988
Accanthopus velikensis	1988
Accanthopus velikensis	1988
Accanthopus velikensis	1941
Accanthopus velikensis	1980
Accanthopus velikensis	1980
Accanthopus velikensis	1970
Accanthopus velikensis	1987
Accanthopus velikensis	1987
Accanthopus velikensis	1987
Accanthopus velikensis	1987
Accanthopus velikensis	1987
Accanthopus velikensis	1987
Accanthopus velikensis	1988
Accanthopus velikensis	1988
Accanthopus velikensis	1988
Accanthopus velikensis	1988

Accanthopus velikensis	1988
Accanthopus velikensis	1988
Accanthopus velikensis	1988
Accanthopus velikensis	1988
Accanthopus velikensis	1988
Accanthopus velikensis	1988
Accanthopus velikensis	1988
Accanthopus velikensis	1959
Accanthopus velikensis	1960
Accanthopus velikensis	1964
Accanthopus velikensis	1973
Accanthopus velikensis	1985
Accanthopus velikensis	1988
Accanthopus velikensis	1979
Accanthopus velikensis	1979
Accanthopus velikensis	1979
Accanthopus velikensis	1979
Accanthopus velikensis	1979
Accanthopus velikensis	1979
Accanthopus velikensis	1979
Accanthopus velikensis	1979
Accanthopus velikensis	1980
Accanthopus velikensis	1980
Accanthopus velikensis	1980
Accanthopus velikensis	1980
Accanthopus velikensis	1980
Accanthopus velikensis	1983
Accanthopus velikensis	1983
Accanthopus velikensis	1999
Accanthopus velikensis	1999
Accanthopus velikensis	1999
Accanthopus velikensis	1999
Accanthopus velikensis	1999
Accanthopus velikensis	1999
Accanthopus velikensis	1999
Accanthopus velikensis	1999
Accanthopus velikensis	1999
Accanthopus velikensis	1980
Accanthopus velikensis	1931
Accanthopus velikensis	1980
Accanthopus velikensis	1982
Accanthopus velikensis	1982
Accanthopus velikensis	1983
Accanthopus velikensis	1996
Accanthopus velikensis	1997
Accanthopus velikensis	1974
Accanthopus velikensis	1986
Accanthopus velikensis	1965
Accanthopus velikensis	1997
Accanthopus velikensis	1978
Accanthopus velikensis	1980
Accanthopus velikensis	1999
Accanthopus velikensis	1936
Accanthopus velikensis	1937

Accanthopus velikensis	1992
Accanthopus velikensis	1992
Accanthopus velikensis	1992
Accanthopus velikensis	1992
Accanthopus velikensis	1992
Accanthopus velikensis	1993
Accanthopus velikensis	1989
Accanthopus velikensis	1998
Accanthopus velikensis	1994
Accanthopus velikensis	2001
Accanthopus velikensis	2001
Accanthopus velikensis	2001
Accanthopus velikensis	2001
Accanthopus velikensis	1999
Accanthopus velikensis	1998
Accanthopus velikensis	1998
Accanthopus velikensis	1972
Accanthopus velikensis	1958
Accanthopus velikensis	1994
Accanthopus velikensis	2001
Accanthopus velikensis	1907
Accanthopus velikensis	1910
Accanthopus velikensis	1910
Accanthopus velikensis	1952
Accanthopus velikensis	1952
Accanthopus velikensis	1969
Accanthopus velikensis	1969
Accanthopus velikensis	1969
Accanthopus velikensis	1970
Accanthopus velikensis	1972
Accanthopus velikensis	1972
Accanthopus velikensis	1991
Accanthopus velikensis	1994
Accanthopus velikensis	1996
Accanthopus velikensis	1997
Accanthopus velikensis	1997
Accanthopus velikensis	1998
Accanthopus velikensis	1999
Accanthopus velikensis	1999
Accanthopus velikensis	2000
Accanthopus velikensis	2000
Accanthopus velikensis	1983
Accanthopus velikensis	1985
Accanthopus velikensis	1985
Accanthopus velikensis	1985
Accanthopus velikensis	1985
Accanthopus velikensis	1985
Accanthopus velikensis	1985
Accanthopus velikensis	1985
Accanthopus velikensis	1985
Accanthopus velikensis	1985
Accanthopus velikensis	1985
Accanthopus velikensis	1985
Accanthopus velikensis	1985

Accanthopus velikensis	1985
Accanthopus velikensis	1985
Accanthopus velikensis	1985
Accanthopus velikensis	1985
Accanthopus velikensis	1985
Accanthopus velikensis	1985
Accanthopus velikensis	1985
Accanthopus velikensis	1985
Accanthopus velikensis	1985
Accanthopus velikensis	1985
Accanthopus velikensis	1985
Accanthopus velikensis	1985
Accanthopus velikensis	1985
Accanthopus velikensis	1985
Accanthopus velikensis	1985
Accanthopus velikensis	1985
Accanthopus velikensis	1985
Accanthopus velikensis	1985
Accanthopus velikensis	1985
Accanthopus velikensis	1985
Accanthopus velikensis	1985
Accanthopus velikensis	1985
Accanthopus velikensis	1985
Accanthopus velikensis	1985
Accanthopus velikensis	1985
Accanthopus velikensis	1985
Accanthopus velikensis	1985
Accanthopus velikensis	1985
Accanthopus velikensis	1985
Accanthopus velikensis	1985
Accanthopus velikensis	1986
Accanthopus velikensis	1986
Accanthopus velikensis	1986
Accanthopus velikensis	1986
Accanthopus velikensis	1986
Accanthopus velikensis	1986
Accanthopus velikensis	1986
Accanthopus velikensis	1986
Accanthopus velikensis	1986
Accanthopus velikensis	1986
Accanthopus velikensis	1986
Accanthopus velikensis	1986
Accanthopus velikensis	1986
Accanthopus velikensis	1986
Accanthopus velikensis	1986
Accanthopus velikensis	1986
Accanthopus velikensis	1986
Accanthopus velikensis	1986
Accanthopus velikensis	1986
Accanthopus velikensis	1986
Accanthopus velikensis	1986
Accanthopus velikensis	1986
Accanthopus velikensis	1986
Accanthopus velikensis	1986

Accanthopus velikensis	1986
Accanthopus velikensis	1986
Accanthopus velikensis	1986
Accanthopus velikensis	1986
Accanthopus velikensis	1986
Accanthopus velikensis	1986
Accanthopus velikensis	1986
Accanthopus velikensis	1986
Accanthopus velikensis	1986
Accanthopus velikensis	1986
Accanthopus velikensis	1987
Accanthopus velikensis	1939
Accanthopus velikensis	1942
Accanthopus velikensis	1988
Accanthopus velikensis	1989
Accanthopus velikensis	2002
Accanthopus velikensis	1971
Accanthopus velikensis	1984
Accanthopus velikensis	1985
Accanthopus velikensis	1985
Accanthopus velikensis	1985
Accanthopus velikensis	1985
Accanthopus velikensis	1985
Accanthopus velikensis	1985
Accanthopus velikensis	1985
Accanthopus velikensis	1985
Accanthopus velikensis	1985
Accanthopus velikensis	1985
Accanthopus velikensis	1985
Accanthopus velikensis	1985
Accanthopus velikensis	1985
Accanthopus velikensis	1985
Accanthopus velikensis	1985
Accanthopus velikensis	1985
Accanthopus velikensis	1985
Accanthopus velikensis	1985
Accanthopus velikensis	1985
Accanthopus velikensis	1985
Accanthopus velikensis	1985
Accanthopus velikensis	1985
Accanthopus velikensis	1985
Accanthopus velikensis	1985
Accanthopus velikensis	1985
Accanthopus velikensis	1985
Accanthopus velikensis	1985
Accanthopus velikensis	1985
Accanthopus velikensis	1996
Accanthopus velikensis	1997
Accanthopus velikensis	1997
Accanthopus velikensis	1997
Accanthopus velikensis	1997
Accanthopus velikensis	1998
Accanthopus velikensis	1999
Accanthopus velikensis	1999
Accanthopus velikensis	1999

Accanthopus velikensis	1999
Accanthopus velikensis	1999
Accanthopus velikensis	2001
Accanthopus velikensis	1997
Accanthopus velikensis	1997
Accanthopus velikensis	1997
Accanthopus velikensis	1997
Accanthopus velikensis	1997
Accanthopus velikensis	1997
Accanthopus velikensis	1969
Accanthopus velikensis	1948
Accanthopus velikensis	1978
Accanthopus velikensis	1978
Accanthopus velikensis	1978
Accanthopus velikensis	1967
Accanthopus velikensis	1967
Accanthopus velikensis	1989
Accanthopus velikensis	1989
Accanthopus velikensis	1989
Accanthopus velikensis	1989
Accanthopus velikensis	1989
Accanthopus velikensis	1989
Accanthopus velikensis	1989
Accanthopus velikensis	1989
Accanthopus velikensis	1989
Accanthopus velikensis	1989
Accanthopus velikensis	1989
Accanthopus velikensis	1989
Accanthopus velikensis	1989
Accanthopus velikensis	1989
Accanthopus velikensis	1989
Accanthopus velikensis	1989
Accanthopus velikensis	1989
Accanthopus velikensis	1989
Accanthopus velikensis	1989
Accanthopus velikensis	1900
Accanthopus velikensis	1900
Accanthopus velikensis	1901
Accanthopus velikensis	1902
Accanthopus velikensis	1902
Accanthopus velikensis	1903
Accanthopus velikensis	1907
Accanthopus velikensis	1907
Accanthopus velikensis	1907
Accanthopus velikensis	1907
Accanthopus velikensis	1908
Accanthopus velikensis	1908
Accanthopus velikensis	1913
Accanthopus velikensis	1915
Accanthopus velikensis	1932
Accanthopus velikensis	1940
Accanthopus velikensis	1941
Accanthopus velikensis	1942
Accanthopus velikensis	1943

Accanthopus velikensis	1970
Accanthopus velikensis	1970
Accanthopus velikensis	1972
Accanthopus velikensis	1987
Accanthopus velikensis	1994
Accanthopus velikensis	1998
Akis bacarozzo	1996
Akis bacarozzo	1985
Akis bacarozzo	2001
Akis bacarozzo	1926
Akis bacarozzo	1996
Akis bacarozzo	1946
Akis bacarozzo	1956
Akis bacarozzo	1994
Akis bacarozzo	1937
Akis bacarozzo	1938
Akis bacarozzo	1960
Akis bacarozzo	1960
Akis bacarozzo	1960
Akis bacarozzo	1981
Akis bacarozzo	1966
Akis bacarozzo	1967
Akis bacarozzo	1969
Akis bacarozzo	1969
Akis bacarozzo	1961
Akis bacarozzo	1981
Akis bacarozzo	1997
Akis bacarozzo	1986
Akis bacarozzo	1986
Akis bacarozzo	1931
Akis bacarozzo	1937
Akis bacarozzo	1982
Akis bacarozzo	1990
Akis bacarozzo	1990
Akis bacarozzo	1978
Akis bacarozzo	1975
Akis bacarozzo	1896
Akis bacarozzo	1897
Akis bacarozzo	1915
Akis bacarozzo	1915
Akis bacarozzo	1921
Akis bacarozzo	1927
Akis bacarozzo	1968
Akis bacarozzo	1969
Akis bacarozzo	1994
Akis bacarozzo	1997
Akis bacarozzo	1997
Akis bacarozzo	1939
Akis bacarozzo	1942
Akis bacarozzo	1994
Akis bacarozzo	1990
Akis bacarozzo	1990
Akis bacarozzo	1990
Akis bacarozzo	1990

Akis bacarozzo	1986
Akis bacarozzo	1998
Akis bacarozzo	2008
Akis bacarozzo	2009
Akis bacarozzo	2009
Akis bacarozzo	2009
Akis bacarozzo	2010
Akis bacarozzo	2005
Akis bacarozzo	1992
Akis bacarozzo	1992
Akis bacarozzo	1994
Akis bacarozzo	1994
Akis bacarozzo	1994
Akis bacarozzo	1911
Akis bacarozzo	1992
Akis bacarozzo	1993
Akis bacarozzo	1994
Akis bacarozzo	1994
Akis bacarozzo	1878
Akis bacarozzo	1880
Akis bacarozzo	1882
Akis bacarozzo	1893
Akis bacarozzo	1911
Akis bacarozzo	1913
Akis bacarozzo	1914
Akis bacarozzo	1915
Akis bacarozzo	1928
Akis bacarozzo	1931
Akis bacarozzo	1932
Akis bacarozzo	1933
Akis bacarozzo	1937
Akis bacarozzo	1938
Akis bacarozzo	1938
Akis bacarozzo	1939
Akis bacarozzo	1940
Akis bacarozzo	1940
Akis bacarozzo	1954
Akis bacarozzo	1957
Akis bacarozzo	1960
Akis bacarozzo	1968
Akis bacarozzo	1979
Akis bacarozzo	1985
Akis bacarozzo	1992
Akis bacarozzo	1992
Akis bacarozzo	1994
Akis bacarozzo	1994
Akis bacarozzo	1994
Akis bacarozzo	1994
Akis italica	1973
Akis italica	1986
Akis italica	1916
Akis italica	1960
Akis italica	1981
Akis italica	1931

Akis italica	1938
Akis italica	1948
Akis italica	1994
Akis italica	1896
Akis italica	1897
Akis italica	1897
Akis italica	1898
Akis italica	1898
Akis italica	1901
Akis italica	1901
Akis italica	1927
Akis italica	1928
Akis italica	1930
Akis italica	1969
Akis italica	1969
Akis italica	1969
Akis italica	1969
Akis italica	1906
Akis italica	1906
Akis italica	1906
Akis italica	1910
Akis italica	2005
Akis italica	1994
Akis italica	1901
Akis italica	1901
Akis italica	1901
Akis italica	1904
Akis italica	1904
Akis italica	1914
Akis italica	1921
Akis italica	1957
Akis italica	1958
Akis italica	1960
Akis italica	1968
Akis italica	1971
Akis italica	1974
Akis italica	1974
Akis italica	1983
Akis italica	1995
Allophylax picipes picipes	1921
Allophylax picipes picipes	1971
Allophylax picipes picipes	1972
Ammobius rufus	1904
Ammobius rufus	1947
Ammobius rufus	1983
Ammobius rufus	1983
Ammobius rufus	1983
Ammobius rufus	1983
Ammobius rufus	1983
Ammobius rufus	1983
Ammobius rufus	1983
Ammobius rufus	1983
Ammobius rufus	1983
Ammobius rufus	1983

Ammobius rufus	1983
Ammobius rufus	1983
Ammobius rufus	1983
Ammobius rufus	1983
Ammobius rufus	1983
Ammobius rufus	1983
Ammobius rufus	1983
Ammobius rufus	1983
Ammobius rufus	1983
Ammobius rufus	1983
Ammobius rufus	1984
Ammobius rufus	1984
Ammobius rufus	1984
Ammobius rufus	1984
Ammobius rufus	1975
Ammobius rufus	1975
Ammobius rufus	1988
Ammobius rufus	1986
Ammobius rufus	1963
Ammobius rufus	1977
Ammobius rufus	1978
Ammobius rufus	1978
Ammobius rufus	1978
Ammobius rufus	1988
Ammobius rufus	1975
Ammobius rufus	1896
Ammobius rufus	1997
Ammobius rufus	1997
Ammobius rufus	1997
Ammobius rufus	1997
Ammobius rufus	1997
Ammobius rufus	1997
Ammobius rufus	1997
Ammobius rufus	1997
Ammobius rufus	1997
Ammobius rufus	1997
Ammobius rufus	1998
Ammobius rufus	1998
Ammobius rufus	1998
Ammobius rufus	1998
Ammobius rufus	1998
Ammobius rufus	1998
Ammobius rufus	1998
Ammobius rufus	1998
Ammobius rufus	1998
Ammobius rufus	1998
Ammobius rufus	1998
Ammobius rufus	1998
Ammobius rufus	1999
Ammobius rufus	1999
Ammobius rufus	2004
Ammobius rufus	1997
Ammobius rufus	1997
Ammobius rufus	1997

Ammobius rufus	1932
Asida bayardi	1916
Asida bayardi	1987
Asida bayardi	1988
Asida bayardi	1988
Asida bayardi	1988
Asida bayardi	1981
Asida bayardi	1982
Asida bayardi	1982
Asida bayardi	1982
Asida bayardi	1967
Asida bayardi	1967
Asida bayardi	1981
Asida bayardi	1998
Asida bayardi	1906
Asida bayardi	1989
Asida bayardi	1991
Asida bayardi	1988
Asida bayardi	1971
Asida bayardi	1985
Asida bayardi	1985
Asida bayardi	1985
Asida bayardi	1985
Asida bayardi	1977
Asida bayardi	1977
Asida bayardi	1977
Asida bayardi	1978
Asida bayardi	1978
Asida bayardi	1978
Asida bayardi	1978
Asida bayardi	1978
Asida bayardi	1978
Asida bayardi	1978
Asida bayardi	1978
Asida bayardi	1978
Asida bayardi	1978
Asida bayardi	1908
Asida luigionii luigionii	2001
Asida luigionii luigionii	1945
Asida luigionii luigionii	1945
Asida luigionii luigionii	1978
Asida luigionii luigionii	1911
Asida luigionii luigionii	1911
Asida luigionii luigionii	1916
Asida luigionii luigionii	1982
Asida luigionii luigionii	1982
Asida luigionii luigionii	1982
Asida luigionii luigionii	1969
Asida luigionii luigionii	1987
Asida luigionii luigionii	1987
Asida luigionii luigionii	1987
Asida luigionii luigionii	1987
Asida luigionii luigionii	1988
Asida luigionii luigionii	1988

Asida luigionii luigionii	1988
Asida luigionii luigionii	1988
Asida luigionii luigionii	1988
Asida luigionii luigionii	1988
Asida luigionii luigionii	1980
Asida luigionii luigionii	1982
Asida luigionii luigionii	1982
Asida luigionii luigionii	1983
Asida luigionii luigionii	1983
Asida luigionii luigionii	1983
Asida luigionii luigionii	1983
Asida luigionii luigionii	1983
Asida luigionii luigionii	1983
Asida luigionii luigionii	1983
Asida luigionii luigionii	1983
Asida luigionii luigionii	1999
Asida luigionii luigionii	1999
Asida luigionii luigionii	1999
Asida luigionii luigionii	1999
Asida luigionii luigionii	1999
Asida luigionii luigionii	1894
Asida luigionii luigionii	1982
Asida luigionii luigionii	1982
Asida luigionii luigionii	1878
Asida luigionii luigionii	1988
Asida luigionii luigionii	1988
Asida luigionii luigionii	1985
Asida luigionii luigionii	1991
Asida luigionii luigionii	1960
Asida luigionii luigionii	1960
Asida luigionii luigionii	1942
Asida luigionii luigionii	1930
Asida luigionii luigionii	1937
Asida luigionii luigionii	1941
Asida luigionii luigionii	1945
Asida luigionii luigionii	1992
Asida luigionii luigionii	1992
Asida luigionii luigionii	1992
Asida luigionii luigionii	1992
Asida luigionii luigionii	1992
Asida luigionii luigionii	1992
Asida luigionii luigionii	1992
Asida luigionii luigionii	1992
Asida luigionii luigionii	1938
Asida luigionii luigionii	1910
Asida luigionii luigionii	1913
Asida luigionii luigionii	1917
Asida luigionii luigionii	1917
Asida luigionii luigionii	1936
Asida luigionii luigionii	1969
Asida luigionii luigionii	1970
Asida luigionii luigionii	1970
Asida luigionii luigionii	1970
Asida luigionii luigionii	1971

Asida luigionii luigionii	1971
Asida luigionii luigionii	1973
Asida luigionii luigionii	1994
Asida luigionii luigionii	1995
Asida luigionii luigionii	1995
Asida luigionii luigionii	1995
Asida luigionii luigionii	1995
Asida luigionii luigionii	1995
Asida luigionii luigionii	1996
Asida luigionii luigionii	1996
Asida luigionii luigionii	1996
Asida luigionii luigionii	1996
Asida luigionii luigionii	1996
Asida luigionii luigionii	1996
Asida luigionii luigionii	1997
Asida luigionii luigionii	1997
Asida luigionii luigionii	1997
Asida luigionii luigionii	1997
Asida luigionii luigionii	1997
Asida luigionii luigionii	1997
Asida luigionii luigionii	1998
Asida luigionii luigionii	1998
Asida luigionii luigionii	1998
Asida luigionii luigionii	1998
Asida luigionii luigionii	1998
Asida luigionii luigionii	1999
Asida luigionii luigionii	1999
Asida luigionii luigionii	1998
Asida luigionii luigionii	1984
Asida luigionii luigionii	1984
Asida luigionii luigionii	1997
Asida luigionii luigionii	1998
Asida luigionii luigionii	2004
Asida luigionii luigionii	2010
Asida luigionii luigionii	1948
Asida luigionii luigionii	1897
Asida luigionii luigionii	1904
Asida luigionii luigionii	1908
Asida luigionii luigionii	1908
Asida luigionii luigionii	1909
Asida luigionii luigionii	1910
Asida luigionii luigionii	1938
Asida luigionii luigionii	1960
Asida pirazzolii pirazzolii	1987
Asida pirazzolii pirazzolii	1990
Asida pirazzolii pirazzolii	1997
Asida pirazzolii pirazzolii	1998
Asida pirazzolii pirazzolii	1998
Asida pirazzolii pirazzolii	1998
Asida pirazzolii pirazzolii	1997
Asida pirazzolii pirazzolii	1997
Asida pirazzolii pirazzolii	1996
Asida pirazzolii pirazzolii	1992
Asida pirazzolii pirazzolii	1938

Asida pirazzolii pirazzolii	1965
Asida pirazzolii pirazzolii	1912
Asida pirazzolii pirazzolii	1987
Asida pirazzolii pirazzolii	1995
Asida pirazzolii pirazzolii	1992
Asida pirazzolii pirazzolii	1992
Asida pirazzolii pirazzolii	1992
Asida pirazzolii pirazzolii	1992
Asida pirazzolii pirazzolii	2000
Asida pirazzolii pirazzolii	1942
Asida pirazzolii pirazzolii	1968
Asida pirazzolii pirazzolii	1969
Asida pirazzolii pirazzolii	1969
Asida pirazzolii sardiniensis	1987
Asida pirazzolii sardiniensis	1986
Asida pirazzolii sardiniensis	1959
Asida pirazzolii sardiniensis	1913
Asida pirazzolii sardiniensis	1913
Asida pirazzolii sardiniensis	1992
Asida pirazzolii sardiniensis	1967
Asida pirazzolii sardiniensis	1973
Asida pirazzolii sardiniensis	1993
Asida pirazzolii sardiniensis	1993
Asida pirazzolii sardiniensis	1974
Asida pirazzolii sardiniensis	1960
Asida pirazzolii sardiniensis	1994
Asida pirazzolii sardiniensis	1994
Asida pirazzolii sardiniensis	1992
Asida pirazzolii sardiniensis	1898
Asida pirazzolii sardiniensis	1908
Asida pirazzolii sardiniensis	1912
Asida pirazzolii sardiniensis	1917
Asida pirazzolii sardiniensis	1912
Asida pirazzolii sardiniensis	1988
Asida pirazzolii sardiniensis	1989
Asida pirazzolii sardiniensis	1898
Asida pirazzolii sardiniensis	1908
Asida pirazzolii sardiniensis	1912
Asida pirazzolii sardiniensis	1917
Asida pirazzolii sardiniensis	1917
Asida pirazzolii sardiniensis	1923
Asida pirazzolii sardiniensis	1993
Asida pirazzolii sardiniensis	1994
Asida sabulosa	1992
Asida sabulosa	1915
Asida sabulosa	1992
Asida sabulosa	1994
Asida sabulosa	1979
Asida sabulosa	1989
Asida sabulosa	1989
Asida sabulosa	1984
Asida sabulosa	1976
Asida sabulosa	1923
Asida sabulosa	1905

Asida sabulosa	1919
Asida sabulosa	1919
Asida sabulosa	1927
Asida sabulosa	1940
Asida sabulosa	1947
Asida sabulosa	1961
Asida sabulosa	1963
Asida sabulosa	1973
Asida sabulosa	1985
Blaps gibba	1997
Blaps gibba	1997
Blaps gibba	1997
Blaps gibba	1998
Blaps gibba	1999
Blaps gibba	2002
Blaps gibba	2001
Blaps gibba	1974
Blaps gibba	1974
Blaps gibba	1927
Blaps gibba	1946
Blaps gibba	1954
Blaps gibba	1986
Blaps gibba	1987
Blaps gibba	1997
Blaps gibba	1982
Blaps gibba	1939
Blaps gibba	1944
Blaps gibba	1938
Blaps gibba	1988
Blaps gibba	1988
Blaps gibba	1988
Blaps gibba	1960
Blaps gibba	1997
Blaps gibba	1992
Blaps gibba	1999
Blaps gibba	1999
Blaps gibba	1999
Blaps gibba	1980
Blaps gibba	1977
Blaps gibba	1967
Blaps gibba	1968
Blaps gibba	1968
Blaps gibba	1969
Blaps gibba	1969
Blaps gibba	1986
Blaps gibba	1989
Blaps gibba	1999
Blaps gibba	1983
Blaps gibba	1997
Blaps gibba	1997
Blaps gibba	1997
Blaps gibba	1980
Blaps gibba	1980
Blaps gibba	1982

Blaps gibba	1982
Blaps gibba	1982
Blaps gibba	1983
Blaps gibba	1985
Blaps gibba	1986
Blaps gibba	1987
Blaps gibba	1987
Blaps gibba	1987
Blaps gibba	1987
Blaps gibba	1994
Blaps gibba	1995
Blaps gibba	1997
Blaps gibba	1958
Blaps gibba	1978
Blaps gibba	1965
Blaps gibba	1999
Blaps gibba	1936
Blaps gibba	1990
Blaps gibba	1997
Blaps gibba	1997
Blaps gibba	1900
Blaps gibba	1990
Blaps gibba	1990
Blaps gibba	1990
Blaps gibba	1984
Blaps gibba	1999
Blaps gibba	1988
Blaps gibba	1988
Blaps gibba	1989
Blaps gibba	1989
Blaps gibba	1989
Blaps gibba	1989
Blaps gibba	1989
Blaps gibba	1931
Blaps gibba	1975
Blaps gibba	1975
Blaps gibba	1900
Blaps gibba	1901
Blaps gibba	1935
Blaps gibba	1950
Blaps gibba	1969
Blaps gibba	1969
Blaps gibba	1969
Blaps gibba	1969
Blaps gibba	1969
Blaps gibba	1969
Blaps gibba	1969
Blaps gibba	1971
Blaps gibba	1971
Blaps gibba	1971
Blaps gibba	1972
Blaps gibba	1972
Blaps gibba	1973
Blaps gibba	1973

Blaps gibba	1974
Blaps gibba	1997
Blaps gibba	1999
Blaps gibba	1942
Blaps gibba	1989
Blaps gibba	1995
Blaps gibba	1997
Blaps gibba	1997
Blaps gibba	1997
Blaps gibba	1997
Blaps gibba	1997
Blaps gibba	1997
Blaps gibba	1997
Blaps gibba	1998
Blaps gibba	1999
Blaps gibba	1999
Blaps gibba	1999
Blaps gibba	2004
Blaps gibba	2006
Blaps gibba	2009
Blaps gibba	1996
Blaps gibba	1996
Blaps gibba	1997
Blaps gibba	1997
Blaps gibba	1997
Blaps gibba	1981
Blaps gibba	1982
Blaps gibba	1982
Blaps gibba	1984
Blaps gibba	1984
Blaps gibba	1984
Blaps gibba	1985
Blaps gibba	1987
Blaps gibba	1988
Blaps gibba	1988
Blaps gibba	1991
Blaps gibba	1992
Blaps gibba	1993
Blaps gibba	1993
Blaps gibba	1993
Blaps gibba	1993
Blaps gibba	1995
Blaps gibba	1995
Blaps gibba	1997
Blaps gibba	1997
Blaps gibba	1977
Blaps gibba	1978
Blaps gibba	1990
Blaps gibba	1991
Blaps gibba	1991
Blaps gibba	1992
Blaps gibba	1992
Blaps gibba	1992
Blaps gibba	1995

Blaps gibba	1996
Blaps gibba	1963
Blaps gibba	1994
Blaps gibba	1904
Blaps gibba	1909
Blaps gibba	1932
Blaps gibba	1932
Blaps gibba	1932
Blaps gibba	1936
Blaps gibba	1938
Blaps gibba	1938
Blaps gibba	1938
Blaps gibba	1940
Blaps gibba	1940
Blaps gibba	1940
Blaps gibba	1940
Blaps gibba	1940
Blaps gibba	1940
Blaps gibba	1941
Blaps gibba	1942
Blaps gibba	1942
Blaps gibba	1942
Blaps gibba	1948
Blaps gibba	1953
Blaps gibba	1957
Blaps gibba	1960
Blaps gibba	1960
Blaps gibba	1965
Blaps gibba	1969
Blaps gibba	1969
Blaps gibba	1970
Blaps gibba	1976
Blaps gibba	1979
Blaps gibba	1986
Blaps gibba	1992
Blaps gibba	
Blaps gigas	1961
Blaps gigas	1960
Blaps gigas	1969
Blaps gigas	1915
Blaps gigas	1937
Blaps gigas	1937
Blaps gigas	1975
Blaps gigas	1901
Blaps gigas	1969
Blaps gigas	1950
Blaps gigas	1958
Blaps gigas	1960
Blaps gigas	1968
Blaps gigas	1969
Blaps lethifera lethifera	1900
Blaps lethifera lethifera	1962
Blaps lethifera lethifera	1968
Blaps lethifera lethifera	1993

Blaps lethifera lethifera	1971
Blaps lethifera lethifera	1901
Blaps lethifera lethifera	1953
Blaps lethifera lethifera	1960
Blaps mucronata	1968
Blaps mucronata	1994
Blaps mucronata	1960
Blaps mucronata	1980
Blaps mucronata	1975
Blaps mucronata	1995
Blaps mucronata	1901
Blaps mucronata	1969
Blaps mucronata	1938
Blaps mucronata	1997
Blaps mucronata	1997
Blaps mucronata	2009
Blaps mucronata	2005
Blaps mucronata	2006
Blaps mucronata	1997
Blaps mucronata	1999
Blaps mucronata	1938
Blaps mucronata	1942
Blaps mucronata	1960
Blaps mucronata	1969
Blaps mucronata	1979
Bolitophagus interruptus	1908
Bolitophagus interruptus	1908
Bolitophagus interruptus	1910
Bolitophagus interruptus	1917
Bolitophagus reticulatus	1989
Bolitophagus reticulatus	1989
Bolitophagus reticulatus	1961
Bolitophagus reticulatus	1988
Bolitophagus reticulatus	1988
Bolitophagus reticulatus	1995
Bolitophagus reticulatus	1925
Bolitophagus reticulatus	1999
Bolitophagus reticulatus	1999
Bolitophagus reticulatus	2000
Bolitophagus reticulatus	1907
Bolitophagus reticulatus	1908
Bolitophagus reticulatus	1908
Bolitophagus reticulatus	1908
Bolitophagus reticulatus	1908
Bolitophagus reticulatus	1908
Bolitophagus reticulatus	1908
Bolitophagus reticulatus	1909
Bolitophagus reticulatus	1999
Catomus rotundicollis	1998
Catomus rotundicollis	2001
Catomus rotundicollis	2001
Catomus rotundicollis	1997
Catomus rotundicollis	1988
Catomus rotundicollis	1978

Catomus rotundicollis	1950
Catomus rotundicollis	1958
Catomus rotundicollis	1958
Catomus rotundicollis	1959
Catomus rotundicollis	1959
Catomus rotundicollis	1959
Catomus rotundicollis	1959
Catomus rotundicollis	1959
Catomus rotundicollis	1959
Catomus rotundicollis	1959
Catomus rotundicollis	1959
Catomus rotundicollis	1981
Catomus rotundicollis	1981
Catomus rotundicollis	1995
Catomus rotundicollis	1995
Catomus rotundicollis	1971
Catomus rotundicollis	1988
Catomus rotundicollis	1999
Catomus rotundicollis	1956
Catomus rotundicollis	1973
Catomus rotundicollis	1973
Catomus rotundicollis	1989
Catomus rotundicollis	1989
Catomus rotundicollis	1989
Catomus rotundicollis	1989
Catomus rotundicollis	1993
Catomus rotundicollis	1993
Catomus rotundicollis	1994
Catomus rotundicollis	1975
Catomus rotundicollis	1983
Catomus rotundicollis	1987
Catomus rotundicollis	1939
Catomus rotundicollis	1976
Catomus rotundicollis	1982
Catomus rotundicollis	1982
Catomus rotundicollis	1983
Catomus rotundicollis	1988
Catomus rotundicollis	1988
Catomus rotundicollis	1988
Catomus rotundicollis	1988
Catomus rotundicollis	1989
Catomus rotundicollis	1990
Catomus rotundicollis	1983
Catomus rotundicollis	1977
Catomus rotundicollis	1977
Catomus rotundicollis	1937
Catomus rotundicollis	1938
Catomus rotundicollis	1939
Catomus rotundicollis	1992
Catomus rotundicollis	1982
Catomus rotundicollis	2001
Catomus rotundicollis	2001
Catomus rotundicollis	1941
Catomus rotundicollis	1974

Catomus rotundicollis	1974
Catomus rotundicollis	1986
Catomus rotundicollis	1998
Catomus rotundicollis	1972
Catomus rotundicollis	1994
Catomus rotundicollis	1995
Catomus rotundicollis	1996
Catomus rotundicollis	1997
Catomus rotundicollis	1997
Catomus rotundicollis	1997
Catomus rotundicollis	1998
Catomus rotundicollis	1999
Catomus rotundicollis	1942
Catomus rotundicollis	1992
Catomus rotundicollis	1994
Catomus rotundicollis	1994
Catomus rotundicollis	1981
Catomus rotundicollis	1997
Catomus rotundicollis	1999
Catomus rotundicollis	1933
Catomus rotundicollis	1977
Catomus rotundicollis	1977
Catomus rotundicollis	1977
Catomus rotundicollis	1977
Catomus rotundicollis	1978
Catomus rotundicollis	1978
Catomus rotundicollis	1978
Catomus rotundicollis	1978
Catomus rotundicollis	1898
Catomus rotundicollis	1898
Catomus rotundicollis	1898
Catomus rotundicollis	1898
Catomus rotundicollis	1902
Catomus rotundicollis	1908
Catomus rotundicollis	1916
Catomus rotundicollis	1927
Catomus rotundicollis	1930
Catomus rotundicollis	1931
Catomus rotundicollis	1931
Catomus rotundicollis	1932
Catomus rotundicollis	1938
Catomus rotundicollis	1938
Catomus rotundicollis	1938
Catomus rotundicollis	1939
Catomus rotundicollis	1939
Catomus rotundicollis	1940
Catomus rotundicollis	1956
Cheirodes sardous	1974
Cheirodes sardous	1986
Cnemeplatia atropos atropos	1945
Cnemeplatia atropos atropos	2001
Cnemeplatia atropos atropos	1903
Cnemeplatia atropos atropos	1904
Cnemeplatia atropos atropos	1906

Cnemeplatia atropos atropos	1908
Colpotus strigosus ganglbaueri	1993
Colpotus strigosus ganglbaueri	1996
Colpotus strigosus ganglbaueri	1996
Colpotus strigosus ganglbaueri	1992
Colpotus strigosus ganglbaueri	1908
Colpotus strigosus strigosus	1988
Colpotus strigosus strigosus	1998
Colpotus strigosus strigosus	1986
Colpotus strigosus strigosus	1986
Colpotus strigosus strigosus	1986
Colpotus strigosus strigosus	1986
Colpotus strigosus strigosus	1986
Colpotus strigosus strigosus	1987
Colpotus strigosus strigosus	1993
Colpotus strigosus strigosus	1976
Colpotus strigosus strigosus	1900
Colpotus strigosus strigosus	1903
Colpotus strigosus strigosus	1904
Colpotus strigosus strigosus	1908
Colpotus strigosus strigosus	1908
Colpotus strigosus strigosus	1908
Colpotus strigosus strigosus	1988
Colpotus strigosus strigosus	1988
Colpotus strigosus strigosus	1988
Colpotus strigosus strigosus	1988
Colpotus strigosus strigosus	1988
Colpotus strigosus strigosus	1988
Colpotus strigosus strigosus	1988
Colpotus strigosus strigosus	1988
Colpotus strigosus strigosus	1988
Colpotus strigosus strigosus	1988
Colpotus strigosus strigosus	1988
Colpotus strigosus strigosus	1988
Colpotus strigosus strigosus	1988
Colpotus strigosus strigosus	1981
Colpotus strigosus strigosus	1988
Colpotus strigosus strigosus	1988
Colpotus strigosus strigosus	1988
Colpotus strigosus strigosus	1995
Colpotus strigosus strigosus	1969
Colpotus strigosus strigosus	1987
Colpotus strigosus strigosus	1987
Colpotus strigosus strigosus	1987
Colpotus strigosus strigosus	1987
Colpotus strigosus strigosus	1987
Colpotus strigosus strigosus	1987
Colpotus strigosus strigosus	1988
Colpotus strigosus strigosus	1988
Colpotus strigosus strigosus	1988
Colpotus strigosus strigosus	1988
Colpotus strigosus strigosus	1988
Colpotus strigosus strigosus	1988
Colpotus strigosus strigosus	1988

Colpotus strigosus strigosus	1988
Colpotus strigosus strigosus	1988
Colpotus strigosus strigosus	1988
Colpotus strigosus strigosus	1988
Colpotus strigosus strigosus	1988
Colpotus strigosus strigosus	1988
Colpotus strigosus strigosus	1988
Colpotus strigosus strigosus	1988
Colpotus strigosus strigosus	1988
Colpotus strigosus strigosus	1988
Colpotus strigosus strigosus	1988
Colpotus strigosus strigosus	1988
Colpotus strigosus strigosus	1958
Colpotus strigosus strigosus	1960
Colpotus strigosus strigosus	1964
Colpotus strigosus strigosus	1967
Colpotus strigosus strigosus	1973
Colpotus strigosus strigosus	1981
Colpotus strigosus strigosus	1987
Colpotus strigosus strigosus	1979
Colpotus strigosus strigosus	1979
Colpotus strigosus strigosus	1979
Colpotus strigosus strigosus	1980
Colpotus strigosus strigosus	1980
Colpotus strigosus strigosus	1980
Colpotus strigosus strigosus	1983
Colpotus strigosus strigosus	1983
Colpotus strigosus strigosus	1999
Colpotus strigosus strigosus	1999
Colpotus strigosus strigosus	1999
Colpotus strigosus strigosus	1999
Colpotus strigosus strigosus	1999
Colpotus strigosus strigosus	1999
Colpotus strigosus strigosus	1999
Colpotus strigosus strigosus	1999
Colpotus strigosus strigosus	1999
Colpotus strigosus strigosus	1980
Colpotus strigosus strigosus	1946
Colpotus strigosus strigosus	1972
Colpotus strigosus strigosus	1978
Colpotus strigosus strigosus	1996
Colpotus strigosus strigosus	1982
Colpotus strigosus strigosus	1974
Colpotus strigosus strigosus	1974
Colpotus strigosus strigosus	1961
Colpotus strigosus strigosus	1961
Colpotus strigosus strigosus	1983
Colpotus strigosus strigosus	1992
Colpotus strigosus strigosus	1965
Colpotus strigosus strigosus	1979
Colpotus strigosus strigosus	1970
Colpotus strigosus strigosus	1937
Colpotus strigosus strigosus	1942
Colpotus strigosus strigosus	1992

Colpotus strigosus strigosus	1992
Colpotus strigosus strigosus	1992
Colpotus strigosus strigosus	1992
Colpotus strigosus strigosus	1992
Colpotus strigosus strigosus	2001
Colpotus strigosus strigosus	2001
Colpotus strigosus strigosus	2001
Colpotus strigosus strigosus	2001
Colpotus strigosus strigosus	2001
Colpotus strigosus strigosus	2001
Colpotus strigosus strigosus	1939
Colpotus strigosus strigosus	1932
Colpotus strigosus strigosus	1972
Colpotus strigosus strigosus	1973
Colpotus strigosus strigosus	1973
Colpotus strigosus strigosus	1986
Colpotus strigosus strigosus	1994
Colpotus strigosus strigosus	1994
Colpotus strigosus strigosus	1994
Colpotus strigosus strigosus	1996
Colpotus strigosus strigosus	1996
Colpotus strigosus strigosus	1996
Colpotus strigosus strigosus	1996
Colpotus strigosus strigosus	1996
Colpotus strigosus strigosus	1996
Colpotus strigosus strigosus	1996
Colpotus strigosus strigosus	1997
Colpotus strigosus strigosus	1997
Colpotus strigosus strigosus	1998
Colpotus strigosus strigosus	2000
Colpotus strigosus strigosus	2000
Colpotus strigosus strigosus	1985
Colpotus strigosus strigosus	1985
Colpotus strigosus strigosus	1985
Colpotus strigosus strigosus	1985
Colpotus strigosus strigosus	1985
Colpotus strigosus strigosus	1985
Colpotus strigosus strigosus	1985
Colpotus strigosus strigosus	1985
Colpotus strigosus strigosus	1985
Colpotus strigosus strigosus	1985
Colpotus strigosus strigosus	1985
Colpotus strigosus strigosus	1985
Colpotus strigosus strigosus	1985
Colpotus strigosus strigosus	1985
Colpotus strigosus strigosus	1985
Colpotus strigosus strigosus	1985
Colpotus strigosus strigosus	1986
Colpotus strigosus strigosus	1986
Colpotus strigosus strigosus	1986
Colpotus strigosus strigosus	1986
Colpotus strigosus strigosus	1986
Colpotus strigosus strigosus	1986
Colpotus strigosus strigosus	1986

Colpotus strigosus strigosus	1986
Colpotus strigosus strigosus	1989
Colpotus strigosus strigosus	1985
Colpotus strigosus strigosus	1985
Colpotus strigosus strigosus	1985
Colpotus strigosus strigosus	1985
Colpotus strigosus strigosus	1985
Colpotus strigosus strigosus	1985
Colpotus strigosus strigosus	1985
Colpotus strigosus strigosus	1985
Colpotus strigosus strigosus	1985
Colpotus strigosus strigosus	1985
Colpotus strigosus strigosus	1985
Colpotus strigosus strigosus	1985
Colpotus strigosus strigosus	1985
Colpotus strigosus strigosus	1985
Colpotus strigosus strigosus	1999
Colpotus strigosus strigosus	1999
Colpotus strigosus strigosus	2001
Colpotus strigosus strigosus	2004
Colpotus strigosus strigosus	1977
Colpotus strigosus strigosus	1977
Colpotus strigosus strigosus	1977
Colpotus strigosus strigosus	1977
Colpotus strigosus strigosus	1977
Colpotus strigosus strigosus	1977
Colpotus strigosus strigosus	1978
Colpotus strigosus strigosus	1978
Colpotus strigosus strigosus	1978
Colpotus strigosus strigosus	1978
Colpotus strigosus strigosus	1978
Colpotus strigosus strigosus	1978
Colpotus strigosus strigosus	1978
Colpotus strigosus strigosus	1978
Colpotus strigosus strigosus	1978
Colpotus strigosus strigosus	1978
Colpotus strigosus strigosus	1978
Colpotus strigosus strigosus	1978
Colpotus strigosus strigosus	1978
Colpotus strigosus strigosus	1978
Colpotus strigosus strigosus	1978
Colpotus strigosus strigosus	1989
Colpotus strigosus strigosus	1989
Colpotus strigosus strigosus	1989
Colpotus strigosus strigosus	1989
Colpotus strigosus strigosus	1989
Colpotus strigosus strigosus	1989
Colpotus strigosus strigosus	1898
Colpotus strigosus strigosus	1908
Colpotus strigosus strigosus	1932
Colpotus strigosus strigosus	1933
Colpotus strigosus strigosus	1933
Colpotus strigosus strigosus	1937
Colpotus strigosus strigosus	1942

Colpotus strigosus strigosus	1953
Colpotus strigosus strigosus	1954
Colpotus strigosus strigosus	1984
Colpotus strigosus strigosus	1999
Corticeus bicolor	1970
Corticeus bicolor	1937
Corticeus bicolor	1903
Corticeus bicolor	1908
Corticeus fasciatus	1931
Corticeus fasciatus	1931
Corticeus fasciatus	1931
Corticeus fasciatus	1919
Corticeus fasciatus	1939
Corticeus fasciatus	1936
Corticeus pini	1966
Corticeus pini	1993
Corticeus pini	1932
Corticeus pini	1929
Corticeus unicolor	1967
Corticeus unicolor	1989
Corticeus unicolor	1939
Corticeus unicolor	1931
Corticeus unicolor	1986
Corticeus unicolor	1903
Corticeus unicolor	1910
Corticeus unicolor	1919
Corticeus unicolor	1912
Corticeus unicolor	1992
Corticeus unicolor	2002
Corticeus unicolor	1999
Corticeus unicolor	1948
Corticeus unicolor	1904
Corticeus unicolor	1908
Corticeus unicolor	1908
Corticeus unicolor	1909
Corticeus unicolor	1909
Corticeus unicolor	1909
Corticeus unicolor	1933
Corticeus unicolor	1988
Cossyphus tauricus	1988
Cossyphus tauricus	1961
Cossyphus tauricus	1949
Cossyphus tauricus	1952
Cossyphus tauricus	1960
Cossyphus tauricus	1981
Cossyphus tauricus	1996
Cossyphus tauricus	1961
Cossyphus tauricus	1972
Cossyphus tauricus	1946
Cossyphus tauricus	1948
Cossyphus tauricus	1948
Cossyphus tauricus	1992
Cossyphus tauricus	1932
Cossyphus tauricus	1932

Cossyphus tauricus	1932
Cossyphus tauricus	1939
Cossyphus tauricus	1945
Cossyphus tauricus	1910
Cossyphus tauricus	1983
Cossyphus tauricus	1894
Cossyphus tauricus	1913
Cossyphus tauricus	1949
Cossyphus tauricus	1960
Cossyphus tauricus	1961
Cossyphus tauricus	1965
Crypticus gibbulus	
Crypticus quisquilius aprutianus	1982
Crypticus quisquilius aprutianus	1932
Crypticus quisquilius aprutianus	1993
Crypticus quisquilius aprutianus	1937
Dendarus coarticollis	1973
Dendarus coarticollis	1988
Dendarus coarticollis	1999
Dendarus coarticollis	1947
Dendarus coarticollis	1988
Dendarus coarticollis	1986
Dendarus coarticollis	1986
Dendarus coarticollis	1936
Dendarus coarticollis	1938
Dendarus coarticollis	1963
Dendarus coarticollis	1967
Dendarus coarticollis	1973
Dendarus coarticollis	1989
Dendarus coarticollis	1989
Dendarus coarticollis	1983
Dendarus coarticollis	1980
Dendarus coarticollis	1982
Dendarus coarticollis	1970
Dendarus coarticollis	1948
Dendarus coarticollis	1999
Dendarus coarticollis	1988
Dendarus coarticollis	1992
Dendarus coarticollis	1994
Dendarus coarticollis	1995
Dendarus coarticollis	1996
Dendarus coarticollis	1996
Dendarus coarticollis	1970
Dendarus coarticollis	1932
Dendarus coarticollis	1948
Dendarus coarticollis	1948
Dendarus coarticollis	1986
Dendarus coarticollis	1986
Dendarus coarticollis	1986
Dendarus coarticollis	1986
Dendarus coarticollis	1989
Dendarus coarticollis	1989
Dendarus coarticollis	1989
Dendarus coarticollis	1908

Dendarus coarticollis	1912
Dendarus lugens	1996
Dendarus lugens	1987
Dendarus lugens	1964
Dendarus lugens	1985
Dendarus lugens	1939
Dendarus lugens	1994
Dendarus lugens	1999
Dendarus lugens	2000
Dendarus lugens	1995
Dendarus lugens	1992
Dendarus lugens	1997
Dendarus lugens	1999
Dendarus lugens	1942
Dendarus lugens	1987
Diaclina testudinea	1988
Diaclina testudinea	1978
Diaclina testudinea	1973
Diaclina testudinea	1939
Diaclina testudinea	1939
Diaclina testudinea	2002
Diaclina testudinea	1997
Diaperis boleti	1997
Diaperis boleti	1998
Diaperis boleti	1958
Diaperis boleti	1969
Diaperis boleti	1973
Diaperis boleti	1995
Diaperis boleti	1997
Diaperis boleti	1943
Diaperis boleti	1982
Diaperis boleti	1995
Diaperis boleti	1900
Diaperis boleti	1907
Diaperis boleti	1910
Diaperis boleti	1913
Diaperis boleti	1932
Diaperis boleti	1994
Diaperis boleti	1995
Diaperis boleti	1995
Diaperis boleti	1997
Diaperis boleti	1998
Diaperis boleti	1999
Diaperis boleti	1999
Diaperis boleti	1999
Diaperis boleti	2000
Diaperis boleti	2000
Diaperis boleti	1908
Diaperis boleti	1909
Diaperis boleti	1933
Diaperis boleti	1934
Diaperis boleti	1936
Diaperis boleti	1942
Dichillus minutus	1971

Dichillus minutus	1941
Dichillus minutus	1994
Dichillus minutus	1994
Dichillus minutus	1995
Dichillus minutus	1943
Dichillus minutus	1947
Dichillus minutus	1948
Dichillus minutus	1948
Dichillus minutus	1905
Dichillus minutus	1978
Dichillus minutus	1908
Eledona agricola	1995
Eledona agricola	1899
Eledona agricola	1910
Erodius siculus neapolitanus	1988
Erodius siculus neapolitanus	1998
Erodius siculus neapolitanus	1972
Erodius siculus neapolitanus	2001
Erodius siculus neapolitanus	1974
Erodius siculus neapolitanus	1986
Erodius siculus neapolitanus	1986
Erodius siculus neapolitanus	1987
Erodius siculus neapolitanus	1990
Erodius siculus neapolitanus	1995
Erodius siculus neapolitanus	1995
Erodius siculus neapolitanus	1974
Erodius siculus neapolitanus	1910
Erodius siculus neapolitanus	1977
Erodius siculus neapolitanus	1962
Erodius siculus neapolitanus	1983
Erodius siculus neapolitanus	1983
Erodius siculus neapolitanus	1983
Erodius siculus neapolitanus	1983
Erodius siculus neapolitanus	1983
Erodius siculus neapolitanus	1983
Erodius siculus neapolitanus	1983
Erodius siculus neapolitanus	1983
Erodius siculus neapolitanus	1983
Erodius siculus neapolitanus	1983
Erodius siculus neapolitanus	1983
Erodius siculus neapolitanus	1983
Erodius siculus neapolitanus	1983
Erodius siculus neapolitanus	1983
Erodius siculus neapolitanus	1983
Erodius siculus neapolitanus	1983
Erodius siculus neapolitanus	1983
Erodius siculus neapolitanus	1983
Erodius siculus neapolitanus	1983
Erodius siculus neapolitanus	1984
Erodius siculus neapolitanus	1984
Erodius siculus neapolitanus	1984
Erodius siculus neapolitanus	1984
Erodius siculus neapolitanus	1969
Erodius siculus neapolitanus	1961

Erodius siculus neapolitanus	1988
Erodius siculus neapolitanus	1987
Erodius siculus neapolitanus	1918
Erodius siculus neapolitanus	1963
Erodius siculus neapolitanus	1966
Erodius siculus neapolitanus	1967
Erodius siculus neapolitanus	1969
Erodius siculus neapolitanus	1969
Erodius siculus neapolitanus	1969
Erodius siculus neapolitanus	1977
Erodius siculus neapolitanus	1978
Erodius siculus neapolitanus	1978
Erodius siculus neapolitanus	1974
Erodius siculus neapolitanus	1974
Erodius siculus neapolitanus	1980
Erodius siculus neapolitanus	1985
Erodius siculus neapolitanus	1986
Erodius siculus neapolitanus	1986
Erodius siculus neapolitanus	1988
Erodius siculus neapolitanus	1953
Erodius siculus neapolitanus	1977
Erodius siculus neapolitanus	1937
Erodius siculus neapolitanus	1941
Erodius siculus neapolitanus	1947
Erodius siculus neapolitanus	1982
Erodius siculus neapolitanus	1989
Erodius siculus neapolitanus	1990
Erodius siculus neapolitanus	1997
Erodius siculus neapolitanus	1997
Erodius siculus neapolitanus	1997
Erodius siculus neapolitanus	1997
Erodius siculus neapolitanus	1997
Erodius siculus neapolitanus	1997
Erodius siculus neapolitanus	1997
Erodius siculus neapolitanus	1997
Erodius siculus neapolitanus	1998
Erodius siculus neapolitanus	1998
Erodius siculus neapolitanus	1998
Erodius siculus neapolitanus	1998
Erodius siculus neapolitanus	1998
Erodius siculus neapolitanus	1998
Erodius siculus neapolitanus	1998
Erodius siculus neapolitanus	1999
Erodius siculus neapolitanus	1897
Erodius siculus neapolitanus	1913
Erodius siculus neapolitanus	1972
Erodius siculus neapolitanus	1994
Erodius siculus neapolitanus	1994
Erodius siculus neapolitanus	1994
Erodius siculus neapolitanus	1995
Erodius siculus neapolitanus	1997
Erodius siculus neapolitanus	1942
Erodius siculus neapolitanus	1988
Erodius siculus neapolitanus	1915

Erodius siculus neapolitanus	1917
Erodius siculus neapolitanus	1982
Erodius siculus neapolitanus	1986
Erodius siculus neapolitanus	1996
Erodius siculus neapolitanus	1997
Erodius siculus neapolitanus	1997
Erodius siculus neapolitanus	1997
Erodius siculus neapolitanus	1997
Erodius siculus neapolitanus	1975
Erodius siculus neapolitanus	1978
Erodius siculus neapolitanus	1978
Erodius siculus neapolitanus	1992
Erodius siculus neapolitanus	1992
Erodius siculus neapolitanus	1939
Erodius siculus neapolitanus	1904
Erodius siculus neapolitanus	1907
Erodius siculus neapolitanus	1927
Erodius siculus neapolitanus	1931
Erodius siculus neapolitanus	1932
Erodius siculus neapolitanus	1940
Erodius siculus neapolitanus	1940
Erodius siculus neapolitanus	1940
Erodius siculus neapolitanus	1940
Erodius siculus neapolitanus	1941
Erodius siculus neapolitanus	1941
Erodius siculus neapolitanus	1941
Erodius siculus neapolitanus	1941
Erodius siculus neapolitanus	1941
Erodius siculus neapolitanus	1942
Erodius siculus neapolitanus	1970
Erodius siculus neapolitanus	1997
Erodius siculus neapolitanus	1999
Gonocephalum assimile	1997
Gonocephalum granulatum nigrum	1972
Gonocephalum granulatum nigrum	1935
Gonocephalum granulatum nigrum	1946
Gonocephalum granulatum nigrum	1999
Gonocephalum granulatum nigrum	1980
Gonocephalum granulatum nigrum	1984
Gonocephalum granulatum nigrum	1967
Gonocephalum granulatum nigrum	1968
Gonocephalum granulatum nigrum	1983
Gonocephalum granulatum nigrum	1936
Gonocephalum granulatum nigrum	1937
Gonocephalum granulatum nigrum	1945
Gonocephalum granulatum nigrum	1945
Gonocephalum granulatum nigrum	1988
Gonocephalum granulatum nigrum	1969
Gonocephalum granulatum nigrum	1896
Gonocephalum granulatum nigrum	1898
Gonocephalum granulatum nigrum	1914
Gonocephalum granulatum nigrum	1921
Gonocephalum granulatum nigrum	1972
Gonocephalum granulatum nigrum	1942

Gonocephalum granulatum nigrum	1994
Gonocephalum granulatum nigrum	1975
Gonocephalum granulatum nigrum	1898
Gonocephalum granulatum nigrum	1981
Gonocephalum granulatum nigrum	1959
Gonocephalum granulatum nigrum	1984
Gonocephalum granulatum nigrum	1984
Gonocephalum granulatum nigrum	1984
Gonocephalum granulatum nigrum	1984
Gonocephalum granulatum nigrum	1984
Gonocephalum granulatum nigrum	2010
Gonocephalum granulatum nigrum	1992
Gonocephalum granulatum nigrum	1999
Gonocephalum granulatum nigrum	1900
Gonocephalum granulatum nigrum	1901
Gonocephalum granulatum nigrum	1904
Gonocephalum granulatum nigrum	1907
Gonocephalum granulatum nigrum	1908
Gonocephalum granulatum nigrum	1932
Gonocephalum granulatum nigrum	1934
Gonocephalum granulatum nigrum	1938
Gonocephalum granulatum nigrum	1940
Gonocephalum granulatum nigrum	1940
Gonocephalum granulatum nigrum	1940
Gonocephalum granulatum nigrum	1940
Gonocephalum granulatum nigrum	1941
Gonocephalum granulatum nigrum	1942
Gonocephalum granulatum nigrum	1942
Gonocephalum granulatum nigrum	1987
Gonocephalum granulatum nigrum	1998
Gonocephalum obscurum obscurum	2000
Gonocephalum obscurum obscurum	1946
Gonocephalum obscurum obscurum	1946
Gonocephalum obscurum obscurum	2007
Gonocephalum obscurum obscurum	2006
Gonocephalum obscurum obscurum	1920
Gonocephalum obscurum obscurum	1944
Gonocephalum obscurum obscurum	1988
Gonocephalum rusticum	1940
Gonocephalum rusticum	1946
Gonocephalum rusticum	1947
Gonocephalum rusticum	1972
Gonocephalum rusticum	1969
Gonocephalum rusticum	1920
Gonocephalum rusticum	1936
Gonocephalum rusticum	1946
Gonocephalum rusticum	1946
Gonocephalum rusticum	2006
Gonocephalum rusticum	1907
Gonocephalum rusticum	1908
Gonocephalum rusticum	1931
Gonocephalum rusticum	1933
Gonocephalum rusticum	1935
Gonocephalum rusticum	1942

Gonocephalum rusticum	1942
Gunarus parvulus	1998
Gunarus parvulus	1936
Halammobia pellucida	1977
Halammobia pellucida	1983
Halammobia pellucida	1983
Halammobia pellucida	1983
Halammobia pellucida	1983
Halammobia pellucida	1983
Halammobia pellucida	1983
Halammobia pellucida	1983
Halammobia pellucida	1983
Halammobia pellucida	1983
Halammobia pellucida	1983
Halammobia pellucida	1983
Halammobia pellucida	1983
Halammobia pellucida	1983
Halammobia pellucida	1983
Halammobia pellucida	1983
Halammobia pellucida	1983
Halammobia pellucida	1983
Halammobia pellucida	1983
Halammobia pellucida	1983
Halammobia pellucida	1983
Halammobia pellucida	1984
Halammobia pellucida	1984
Halammobia pellucida	1984
Halammobia pellucida	1984
Halammobia pellucida	1988
Halammobia pellucida	1987
Halammobia pellucida	1938
Halammobia pellucida	1931
Halammobia pellucida	1963
Halammobia pellucida	1978
Halammobia pellucida	1903
Halammobia pellucida	1997
Halammobia pellucida	1997
Halammobia pellucida	1997
Halammobia pellucida	1997
Halammobia pellucida	1997
Halammobia pellucida	1997
Halammobia pellucida	1997
Halammobia pellucida	1997
Halammobia pellucida	1997
Halammobia pellucida	1997
Halammobia pellucida	1998
Halammobia pellucida	1998
Halammobia pellucida	1998
Halammobia pellucida	1998
Halammobia pellucida	1998
Halammobia pellucida	1998
Halammobia pellucida	1998
Halammobia pellucida	1998
Halammobia pellucida	1998

Halammobia pellucida	1998
Halammobia pellucida	1998
Halammobia pellucida	1998
Halammobia pellucida	1999
Halammobia pellucida	1999
Halammobia pellucida	1997
Halammobia pellucida	1997
Halammobia pellucida	1997
Halammobia pellucida	1998
Halammobia pellucida	1998
Halammobia pellucida	1933
Halammobia pellucida	1941
Halammobia pellucida	1908
Halammobia pellucida	1938
Helops caeruleus	1997
Helops caeruleus	1998
Helops caeruleus	2001
Helops caeruleus	1986
Helops caeruleus	1986
Helops caeruleus	1986
Helops caeruleus	1986
Helops caeruleus	1986
Helops caeruleus	1986
Helops caeruleus	1987
Helops caeruleus	1987
Helops caeruleus	1987
Helops caeruleus	1999
Helops caeruleus	1974
Helops caeruleus	1973
Helops caeruleus	1992
Helops caeruleus	1988
Helops caeruleus	1988
Helops caeruleus	1947
Helops caeruleus	1993
Helops caeruleus	1981
Helops caeruleus	1988
Helops caeruleus	1988
Helops caeruleus	1988
Helops caeruleus	1941
Helops caeruleus	1994
Helops caeruleus	1961
Helops caeruleus	1999
Helops caeruleus	1987
Helops caeruleus	1987
Helops caeruleus	1987
Helops caeruleus	1988
Helops caeruleus	1988
Helops caeruleus	1988
Helops caeruleus	1988
Helops caeruleus	1988
Helops caeruleus	1988
Helops caeruleus	1988
Helops caeruleus	1988
Helops caeruleus	1958

Helops caeruleus	1959
Helops caeruleus	1963
Helops caeruleus	1964
Helops caeruleus	1966
Helops caeruleus	1967
Helops caeruleus	1967
Helops caeruleus	1973
Helops caeruleus	1973
Helops caeruleus	1979
Helops caeruleus	1979
Helops caeruleus	1988
Helops caeruleus	1980
Helops caeruleus	1981
Helops caeruleus	2001
Helops caeruleus	1982
Helops caeruleus	1982
Helops caeruleus	1997
Helops caeruleus	1982
Helops caeruleus	1988
Helops caeruleus	1988
Helops caeruleus	1988
Helops caeruleus	1989
Helops caeruleus	1975
Helops caeruleus	1902
Helops caeruleus	1978
Helops caeruleus	1942
Helops caeruleus	1970
Helops caeruleus	1937
Helops caeruleus	1938
Helops caeruleus	1938
Helops caeruleus	1948
Helops caeruleus	1998
Helops caeruleus	1998
Helops caeruleus	1932
Helops caeruleus	1932
Helops caeruleus	1932
Helops caeruleus	1998
Helops caeruleus	2000
Helops caeruleus	1908
Helops caeruleus	1910
Helops caeruleus	1910
Helops caeruleus	1912
Helops caeruleus	1930
Helops caeruleus	1912
Helops caeruleus	1952
Helops caeruleus	1967
Helops caeruleus	1969
Helops caeruleus	1971
Helops caeruleus	1972
Helops caeruleus	1972
Helops caeruleus	1973
Helops caeruleus	1983
Helops caeruleus	1991
Helops caeruleus	1994

Helops caeruleus	1994
Helops caeruleus	1995
Helops caeruleus	1995
Helops caeruleus	1995
Helops caeruleus	1996
Helops caeruleus	1996
Helops caeruleus	1996
Helops caeruleus	1996
Helops caeruleus	1997
Helops caeruleus	1997
Helops caeruleus	1997
Helops caeruleus	1998
Helops caeruleus	1999
Helops caeruleus	1985
Helops caeruleus	1985
Helops caeruleus	1985
Helops caeruleus	1985
Helops caeruleus	1985
Helops caeruleus	1985
Helops caeruleus	1985
Helops caeruleus	1985
Helops caeruleus	1986
Helops caeruleus	1987
Helops caeruleus	1988
Helops caeruleus	1989
Helops caeruleus	1989
Helops caeruleus	2001
Helops caeruleus	1965
Helops caeruleus	1985
Helops caeruleus	1985
Helops caeruleus	1997
Helops caeruleus	1998
Helops caeruleus	1998
Helops caeruleus	2006
Helops caeruleus	1970
Helops caeruleus	1970
Helops caeruleus	1900
Helops caeruleus	1903
Helops caeruleus	1906
Helops caeruleus	1906
Helops caeruleus	1908
Helops caeruleus	1908
Helops caeruleus	1909
Helops caeruleus	1913
Helops caeruleus	1915
Helops caeruleus	1931
Helops caeruleus	1931
Helops caeruleus	1941
Helops caeruleus	1943
Helops caeruleus	1983
Helops caeruleus	1998
Helops rossii	1987
Helops rossii	1906
Helops rossii	1912

Helops rossii	1912
Helops rossii	1913
Helops rossii	1983
Helops rossii	1936
Helops rossii	1966
Helops rossii	1970
Helops rossii	1974
Helops rossii	1982
Helops rossii	1986
Helops rossii	1988
Helops rossii	1972
Helops rossii	1913
Helops rossii	1917
Helops rossii	1940
Helops rossii	1942
Helops rossii	1903
Helops rossii	1909
Iphthiminus italicus italicus	1906
Iphthiminus italicus italicus	1971
Iphthiminus italicus italicus	1988
Iphthiminus italicus italicus	1939
Iphthiminus italicus italicus	1977
Iphthiminus italicus italicus	1960
Iphthiminus italicus italicus	1963
Iphthiminus italicus italicus	1963
Iphthiminus italicus italicus	1964
Iphthiminus italicus italicus	1969
Iphthiminus italicus italicus	1984
Iphthiminus italicus italicus	1984
Iphthiminus italicus italicus	1961
Iphthiminus italicus italicus	1962
Iphthiminus italicus italicus	1963
Iphthiminus italicus italicus	1974
Iphthiminus italicus italicus	1903
Iphthiminus italicus italicus	1961
Iphthiminus italicus italicus	1983
Iphthiminus italicus italicus	1993
Iphthiminus italicus italicus	1969
Iphthiminus italicus italicus	1974
Iphthiminus italicus italicus	1932
Iphthiminus italicus italicus	1932
Iphthiminus italicus italicus	1906
Iphthiminus italicus italicus	1932
Iphthiminus italicus italicus	1932
Iphthiminus italicus italicus	1994
Iphthiminus italicus italicus	1994
Iphthiminus italicus italicus	1994
Iphthiminus italicus italicus	1995
Iphthiminus italicus italicus	1995
Iphthiminus italicus italicus	1995
Iphthiminus italicus italicus	1995
Iphthiminus italicus italicus	1995
Iphthiminus italicus italicus	1996
Iphthiminus italicus italicus	1996

Iphthiminus italicus italicus	1996
Iphthiminus italicus italicus	1997
Iphthiminus italicus italicus	1997
Iphthiminus italicus italicus	1997
Iphthiminus italicus italicus	1999
Iphthiminus italicus italicus	1999
Iphthiminus italicus italicus	1964
Iphthiminus italicus italicus	1968
Iphthiminus italicus italicus	1933
Iphthiminus italicus italicus	1948
Iphthiminus italicus italicus	1948
Iphthiminus italicus italicus	1933
Iphthiminus italicus italicus	1973
Iphthiminus italicus italicus	1906
Iphthiminus italicus italicus	1932
Iphthiminus italicus italicus	1932
Iphthiminus italicus italicus	1932
Iphthiminus italicus italicus	1932
Iphthiminus italicus italicus	1933
Leichenum pulchellum pulchellum	1962
Leichenum pulchellum pulchellum	1997
Leichenum pulchellum pulchellum	1997
Leichenum pulchellum pulchellum	1997
Leichenum pulchellum pulchellum	1946
Leptoderis collaris	1899
Leptoderis collaris	1949
Leptoderis collaris	1949
Leptoderis collaris	1942
Leptoderis collaris	1947
Lyphia tetraphylla	1932
Lyphia tetraphylla	1969
Melanimon tibialis	1902
Melanimon tibialis	1990
Melanimon tibialis	1963
Melanimon tibialis	1914
Melanimon tibialis	1919
Menephilus cylindricus cylindricus	1973
Menephilus cylindricus cylindricus	1975
Menephilus cylindricus cylindricus	1977
Menephilus cylindricus cylindricus	1948
Menephilus cylindricus cylindricus	1986
Menephilus cylindricus cylindricus	2001
Menephilus cylindricus cylindricus	2001
Menephilus cylindricus cylindricus	1914
Menephilus cylindricus cylindricus	1998
Menephilus cylindricus cylindricus	1998
Menephilus cylindricus cylindricus	2001
Menephilus cylindricus cylindricus	1929
Menephilus cylindricus cylindricus	1997
Menephilus cylindricus cylindricus	1997
Menephilus cylindricus cylindricus	1999
Menephilus cylindricus cylindricus	2000
Menephilus cylindricus cylindricus	1993
Menephilus cylindricus cylindricus	1997

Menephilus cylindricus cylindricus	1908
Menephilus cylindricus cylindricus	1908
Menephilus cylindricus cylindricus	1931
Menephilus cylindricus cylindricus	1932
Nalassus aemulus aemulus	1995
Nalassus aemulus aemulus	1995
Nalassus assimilis	1962
Nalassus dryadophilus	1988
Nalassus dryadophilus	1998
Nalassus dryadophilus	2001
Nalassus dryadophilus	2001
Nalassus dryadophilus	1986
Nalassus dryadophilus	2002
Nalassus dryadophilus	1991
Nalassus dryadophilus	1997
Nalassus dryadophilus	1981
Nalassus dryadophilus	1941
Nalassus dryadophilus	1979
Nalassus dryadophilus	1946
Nalassus dryadophilus	1960
Nalassus dryadophilus	1961
Nalassus dryadophilus	1967
Nalassus dryadophilus	1973
Nalassus dryadophilus	1973
Nalassus dryadophilus	1973
Nalassus dryadophilus	1979
Nalassus dryadophilus	1989
Nalassus dryadophilus	1993
Nalassus dryadophilus	1941
Nalassus dryadophilus	1981
Nalassus dryadophilus	1982
Nalassus dryadophilus	1982
Nalassus dryadophilus	1983
Nalassus dryadophilus	1983
Nalassus dryadophilus	1983
Nalassus dryadophilus	1982
Nalassus dryadophilus	1991
Nalassus dryadophilus	1941
Nalassus dryadophilus	1993
Nalassus dryadophilus	1932
Nalassus dryadophilus	1987
Nalassus dryadophilus	1982
Nalassus dryadophilus	1990
Nalassus dryadophilus	2001
Nalassus dryadophilus	1986
Nalassus dryadophilus	1932
Nalassus dryadophilus	1908
Nalassus dryadophilus	1970
Nalassus dryadophilus	1974
Nalassus dryadophilus	1994
Nalassus dryadophilus	1994
Nalassus dryadophilus	1994
Nalassus dryadophilus	1994
Nalassus dryadophilus	1994

Nalassus dryadophilus	1994
Nalassus dryadophilus	1995
Nalassus dryadophilus	1996
Nalassus dryadophilus	1996
Nalassus dryadophilus	1996
Nalassus dryadophilus	1996
Nalassus dryadophilus	1996
Nalassus dryadophilus	1996
Nalassus dryadophilus	1997
Nalassus dryadophilus	1997
Nalassus dryadophilus	1997
Nalassus dryadophilus	1997
Nalassus dryadophilus	1999
Nalassus dryadophilus	1989
Nalassus dryadophilus	2002
Nalassus dryadophilus	1997
Nalassus dryadophilus	1999
Nalassus dryadophilus	1999
Nalassus dryadophilus	1999
Nalassus dryadophilus	1997
Nalassus dryadophilus	1997
Nalassus dryadophilus	1997
Nalassus dryadophilus	1946
Nalassus dryadophilus	1970
Nalassus dryadophilus	1992
Nalassus planipennis	1997
Nalassus planipennis	1988
Nalassus planipennis	1989
Nalassus planipennis	1981
Nalassus planipennis	1946
Nalassus planipennis	1988
Nalassus planipennis	1973
Nalassus planipennis	1973
Nalassus planipennis	1975
Nalassus planipennis	1989
Nalassus planipennis	1989
Nalassus planipennis	1993
Nalassus planipennis	1994
Nalassus planipennis	1941
Nalassus planipennis	1974
Nalassus planipennis	1980
Nalassus planipennis	1983
Nalassus planipennis	1983
Nalassus planipennis	1983
Nalassus planipennis	1984
Nalassus planipennis	1984
Nalassus planipennis	1990
Nalassus planipennis	1990
Nalassus planipennis	1990
Nalassus planipennis	1994
Nalassus planipennis	1995
Nalassus planipennis	1931
Nalassus planipennis	1931
Nalassus planipennis	1945

Nalassus planipennis	1946
Nalassus planipennis	1994
Nalassus planipennis	2001
Nalassus planipennis	1975
Nalassus planipennis	1911
Nalassus planipennis	1912
Nalassus planipennis	1916
Nalassus planipennis	1995
Nalassus planipennis	1997
Nalassus planipennis	1998
Nalassus planipennis	2002
Nalassus planipennis	2002
Nalassus planipennis	1999
Nalassus planipennis	1901
Nalassus planipennis	1902
Nalassus planipennis	1906
Nalassus planipennis	1908
Nalassus planipennis	1909
Nalassus planipennis	1911
Nalassus planipennis	1936
Nalassus planipennis	1938
Nalassus planipennis	1938
Nalassus planipennis	1938
Nalassus planipennis	1938
Nalassus planipennis	1938
Nalassus planipennis	1938
Nalassus planipennis	1941
Nalassus planipennis	1942
Nalassus planipennis	1948
Nalassus planipennis	1969
Nalassus planipennis	1973
Nalassus planipennis	1973
Nalassus planipennis	1973
Nalassus planipennis	1984
Neatus noctivagus	2001
Neatus noctivagus	1961
Neatus noctivagus	1967
Neatus noctivagus	1999
Neatus noctivagus	1994
Neatus noctivagus	1994
Neatus noctivagus	1994
Neatus noctivagus	1997
Neatus noctivagus	2000
Neatus noctivagus	2001
Neatus noctivagus	1903
Neatus noctivagus	1972
Neomida haemorrhoidalis	1989
Neomida haemorrhoidalis	1989
Neomida haemorrhoidalis	1999
Neomida haemorrhoidalis	1917
Neomida haemorrhoidalis	1906
Neomida haemorrhoidalis	1907
Neomida haemorrhoidalis	1908
Neomida haemorrhoidalis	1909

Oochrotus unicolor ardoini	1906
Oochrotus unicolor ardoini	1989
Oochrotus unicolor ardoini	1995
Oochrotus unicolor ardoini	1945
Oochrotus unicolor ardoini	1945
Oochrotus unicolor ardoini	1998
Oochrotus unicolor ardoini	1997
Oochrotus unicolor ardoini	1997
Oochrotus unicolor ardoini	1997
Oochrotus unicolor ardoini	1999
Oochrotus unicolor ardoini	1999
Oochrotus unicolor ardoini	1999
Oochrotus unicolor ardoini	1936
Oochrotus unicolor ardoini	1907
Oochrotus unicolor ardoini	1907
Oochrotus unicolor ardoini	1938
Opatrum sabulosum sabulosum	
Opatrum sabulosum sculptum	1978
Opatrum sabulosum sculptum	1969
Opatrum sabulosum sculptum	1974
Opatrum sabulosum sculptum	1989
Opatrum sabulosum sculptum	1973
Opatrum sabulosum sculptum	1969
Opatrum sabulosum sculptum	1970
Opatrum sabulosum sculptum	1986
Opatrum sabulosum sculptum	1927
Opatrum sabulosum sculptum	1987
Opatrum sabulosum sculptum	1988
Opatrum sabulosum sculptum	1988
Opatrum sabulosum sculptum	1988
Opatrum sabulosum sculptum	1953
Opatrum sabulosum sculptum	1960
Opatrum sabulosum sculptum	1960
Opatrum sabulosum sculptum	1961
Opatrum sabulosum sculptum	1968
Opatrum sabulosum sculptum	1968
Opatrum sabulosum sculptum	1978
Opatrum sabulosum sculptum	1983
Opatrum sabulosum sculptum	1983
Opatrum sabulosum sculptum	1983
Opatrum sabulosum sculptum	1983
Opatrum sabulosum sculptum	1985
Opatrum sabulosum sculptum	1985
Opatrum sabulosum sculptum	1985
Opatrum sabulosum sculptum	1986
Opatrum sabulosum sculptum	1987
Opatrum sabulosum sculptum	1987
Opatrum sabulosum sculptum	1987
Opatrum sabulosum sculptum	1987
Opatrum sabulosum sculptum	1988
Opatrum sabulosum sculptum	1988
Opatrum sabulosum sculptum	1989
Opatrum sabulosum sculptum	1989
Opatrum sabulosum sculptum	1990

Opatrum sabulosum sculptum	1993
Opatrum sabulosum sculptum	1960
Opatrum sabulosum sculptum	1960
Opatrum sabulosum sculptum	1960
Opatrum sabulosum sculptum	1967
Opatrum sabulosum sculptum	1970
Opatrum sabulosum sculptum	1936
Opatrum sabulosum sculptum	1936
Opatrum sabulosum sculptum	1945
Opatrum sabulosum sculptum	1945
Opatrum sabulosum sculptum	1987
Opatrum sabulosum sculptum	1984
Opatrum sabulosum sculptum	1986
Opatrum sabulosum sculptum	1971
Opatrum sabulosum sculptum	1914
Opatrum sabulosum sculptum	1969
Opatrum sabulosum sculptum	1969
Opatrum sabulosum sculptum	1969
Opatrum sabulosum sculptum	1974
Opatrum sabulosum sculptum	1974
Opatrum sabulosum sculptum	1974
Opatrum sabulosum sculptum	1978
Opatrum sabulosum sculptum	1981
Opatrum sabulosum sculptum	1983
Opatrum sabulosum sculptum	1989
Opatrum sabulosum sculptum	1995
Opatrum sabulosum sculptum	1984
Opatrum sabulosum sculptum	1984
Opatrum sabulosum sculptum	1984
Opatrum sabulosum sculptum	1984
Opatrum sabulosum sculptum	1986
Opatrum sabulosum sculptum	1941
Opatrum sabulosum sculptum	1993
Opatrum sabulosum sculptum	1898
Opatrum sabulosum sculptum	1900
Opatrum sabulosum sculptum	1908
Opatrum sabulosum sculptum	1939
Opatrum sabulosum sculptum	1940
Opatrum sabulosum sculptum	1940
Opatrum sabulosum sculptum	1940
Opatrum sabulosum sculptum	1940
Opatrum sabulosum sculptum	1940
Opatrum sabulosum sculptum	1940
Opatrum sabulosum sculptum	1941
Opatrum sabulosum sculptum	1941
Opatrum sabulosum sculptum	1941
Opatrum sabulosum sculptum	1942
Opatrum sabulosum sculptum	1942
Opatrum sabulosum sculptum	1949
Opatrum sabulosum sculptum	1950
Opatrum sabulosum sculptum	1953
Opatrum sabulosum sculptum	1959
Opatrum sabulosum sculptum	1960
Opatrum sabulosum sculptum	1960

Opatrum sabulosum sculptum	1962
Opatrum sabulosum sculptum	1974
Opatrum sabulosum sculptum	1976
Pachychila frioli	1996
Pachychila frioli	1998
Pachychila frioli	1998
Pachychila frioli	1977
Palorus depressus	1999
Palorus depressus	1969
Palorus depressus	1974
Palorus depressus	1991
Palorus depressus	1932
Palorus depressus	1974
Palorus depressus	1974
Palorus depressus	1931
Palorus depressus	1919
Palorus depressus	1902
Palorus depressus	1909
Palorus depressus	1931
Palorus depressus	1944
Pedinus meridianus	1997
Pedinus meridianus	1998
Pedinus meridianus	1973
Pedinus meridianus	1986
Pedinus meridianus	1989
Pedinus meridianus	1999
Pedinus meridianus	1977
Pedinus meridianus	1961
Pedinus meridianus	1981
Pedinus meridianus	1981
Pedinus meridianus	1981
Pedinus meridianus	1982
Pedinus meridianus	1939
Pedinus meridianus	1940
Pedinus meridianus	1980
Pedinus meridianus	1992
Pedinus meridianus	1969
Pedinus meridianus	1987
Pedinus meridianus	1987
Pedinus meridianus	1988
Pedinus meridianus	1988
Pedinus meridianus	1988
Pedinus meridianus	1988
Pedinus meridianus	1988
Pedinus meridianus	1988
Pedinus meridianus	1988
Pedinus meridianus	1961
Pedinus meridianus	1973
Pedinus meridianus	1993
Pedinus meridianus	1963
Pedinus meridianus	1970
Pedinus meridianus	1997
Pedinus meridianus	1997
Pedinus meridianus	1997

Pedinus meridianus	1997
Pedinus meridianus	1997
Pedinus meridianus	1961
Pedinus meridianus	1989
Pedinus meridianus	1979
Pedinus meridianus	1942
Pedinus meridianus	1980
Pedinus meridianus	1936
Pedinus meridianus	1937
Pedinus meridianus	1940
Pedinus meridianus	1945
Pedinus meridianus	1945
Pedinus meridianus	1992
Pedinus meridianus	1992
Pedinus meridianus	1996
Pedinus meridianus	1983
Pedinus meridianus	1989
Pedinus meridianus	1990
Pedinus meridianus	2001
Pedinus meridianus	2001
Pedinus meridianus	1971
Pedinus meridianus	1972
Pedinus meridianus	1979
Pedinus meridianus	1989
Pedinus meridianus	1976
Pedinus meridianus	1976
Pedinus meridianus	1969
Pedinus meridianus	1969
Pedinus meridianus	1969
Pedinus meridianus	1969
Pedinus meridianus	1972
Pedinus meridianus	1973
Pedinus meridianus	1985
Pedinus meridianus	1986
Pedinus meridianus	1987
Pedinus meridianus	1988
Pedinus meridianus	1989
Pedinus meridianus	1990
Pedinus meridianus	1991
Pedinus meridianus	1992
Pedinus meridianus	1994
Pedinus meridianus	1996
Pedinus meridianus	1996
Pedinus meridianus	1996
Pedinus meridianus	1998
Pedinus meridianus	1988
Pedinus meridianus	1996
Pedinus meridianus	2002
Pedinus meridianus	1978
Pedinus meridianus	1980
Pedinus meridianus	1984
Pedinus meridianus	1984
Pedinus meridianus	1984
Pedinus meridianus	1984

Pedinus meridianus	1984
Pedinus meridianus	1984
Pedinus meridianus	1984
Pedinus meridianus	1985
Pedinus meridianus	1997
Pedinus meridianus	1999
Pedinus meridianus	2010
Pedinus meridianus	2010
Pedinus meridianus	2006
Pedinus meridianus	1977
Pedinus meridianus	1978
Pedinus meridianus	1978
Pedinus meridianus	1978
Pedinus meridianus	1992
Pedinus meridianus	1898
Pedinus meridianus	1900
Pedinus meridianus	1901
Pedinus meridianus	1901
Pedinus meridianus	1902
Pedinus meridianus	1908
Pedinus meridianus	1908
Pedinus meridianus	1908
Pedinus meridianus	1912
Pedinus meridianus	1923
Pedinus meridianus	1928
Pedinus meridianus	1932
Pedinus meridianus	1932
Pedinus meridianus	1940
Pedinus meridianus	1940
Pedinus meridianus	1940
Pedinus meridianus	1940
Pedinus meridianus	1940
Pedinus meridianus	1940
Pedinus meridianus	1940
Pedinus meridianus	1940
Pedinus meridianus	1940
Pedinus meridianus	1940
Pedinus meridianus	1940
Pedinus meridianus	1940
Pedinus meridianus	1941
Pedinus meridianus	1941
Pedinus meridianus	1942
Pedinus meridianus	1942
Pedinus meridianus	1942
Pedinus meridianus	1943
Pedinus meridianus	1946
Pedinus meridianus	1948
Pedinus meridianus	1960
Pedinus meridianus	1961
Pedinus meridianus	1962
Pedinus meridianus	1963
Pedinus meridianus	1965
Pedinus meridianus	1965
Pedinus meridianus	1972

Pentaphyllus chrysomeloides	1992
Pentaphyllus chrysomeloides	1992
Pentaphyllus chrysomeloides	1907
Pentaphyllus chrysomeloides	1907
Pentaphyllus chrysomeloides	1909
Pentaphyllus testaceus	1974
Pentaphyllus testaceus	1909
Pentaphyllus testaceus	1910
Pentaphyllus testaceus	1995
Phaleria acuminata	1997
Phaleria acuminata	1960
Phaleria acuminata	2003
Phaleria acuminata	1983
Phaleria acuminata	1983
Phaleria acuminata	1983
Phaleria acuminata	1983
Phaleria acuminata	1983
Phaleria acuminata	1983
Phaleria acuminata	1983
Phaleria acuminata	1983
Phaleria acuminata	1983
Phaleria acuminata	1983
Phaleria acuminata	1983
Phaleria acuminata	1983
Phaleria acuminata	1983
Phaleria acuminata	1983
Phaleria acuminata	1983
Phaleria acuminata	1984
Phaleria acuminata	1984
Phaleria acuminata	1984
Phaleria acuminata	1984
Phaleria acuminata	1986
Phaleria acuminata	1975
Phaleria acuminata	1996
Phaleria acuminata	1977
Phaleria acuminata	1978
Phaleria acuminata	1983
Phaleria acuminata	1997
Phaleria acuminata	1992
Phaleria acuminata	1993
Phaleria acuminata	1995
Phaleria acuminata	1995
Phaleria acuminata	1997
Phaleria acuminata	1984
Phaleria acuminata	1990
Phaleria acuminata	1972
Phaleria acuminata	1975
Phaleria acuminata	1998
Phaleria acuminata	1998
Phaleria acuminata	1998
Phaleria acuminata	1998
Phaleria acuminata	1999
Phaleria acuminata	1940
Phaleria acuminata	1913

Phaleria acuminata	2004
Phaleria acuminata	1998
Phaleria acuminata	1997
Phaleria acuminata	1906
Phaleria acuminata	1914
Phaleria bimaculata bimaculata	1913
Phaleria bimaculata bimaculata	1913
Phaleria bimaculata bimaculata	1916
Phaleria bimaculata bimaculata	1907
Phaleria provincialis ghidinii	1938
Phaleria provincialis ghidinii	1952
Phaleria provincialis ghidinii	1921
Phaleria provincialis ghidinii	1947
Phaleria provincialis ghidinii	1983
Phaleria provincialis ghidinii	1983
Phaleria provincialis ghidinii	1983
Phaleria provincialis ghidinii	1983
Phaleria provincialis ghidinii	1983
Phaleria provincialis ghidinii	1983
Phaleria provincialis ghidinii	1983
Phaleria provincialis ghidinii	1983
Phaleria provincialis ghidinii	1983
Phaleria provincialis ghidinii	1983
Phaleria provincialis ghidinii	1983
Phaleria provincialis ghidinii	1983
Phaleria provincialis ghidinii	1983
Phaleria provincialis ghidinii	1983
Phaleria provincialis ghidinii	1983
Phaleria provincialis ghidinii	1983
Phaleria provincialis ghidinii	1984
Phaleria provincialis ghidinii	1984
Phaleria provincialis ghidinii	1984
Phaleria provincialis ghidinii	1984
Phaleria provincialis ghidinii	1975
Phaleria provincialis ghidinii	1960
Phaleria provincialis ghidinii	1960
Phaleria provincialis ghidinii	1960
Phaleria provincialis ghidinii	1967
Phaleria provincialis ghidinii	1980
Phaleria provincialis ghidinii	1988
Phaleria provincialis ghidinii	1966
Phaleria provincialis ghidinii	1978
Phaleria provincialis ghidinii	1978
Phaleria provincialis ghidinii	1978
Phaleria provincialis ghidinii	1958
Phaleria provincialis ghidinii	1958
Phaleria provincialis ghidinii	1993
Phaleria provincialis ghidinii	1995
Phaleria provincialis ghidinii	1932
Phaleria provincialis ghidinii	1971
Phaleria provincialis ghidinii	1988
Phaleria provincialis ghidinii	1988
Phaleria provincialis ghidinii	1997
Phaleria provincialis ghidinii	1997

Phaleria provincialis ghidinii	1894
Phaleria provincialis ghidinii	1942
Phaleria provincialis ghidinii	1994
Phaleria provincialis ghidinii	1994
Phaleria provincialis ghidinii	2004
Phaleria provincialis ghidinii	1931
Phaleria provincialis ghidinii	1933
Phaleria provincialis ghidinii	1937
Phaleria provincialis ghidinii	1937
Phaleria provincialis ghidinii	1952
Phaleria provincialis ghidinii	1952
Phtora crenata	1969
Phylan italicus	1946
Phylan italicus	1938
Phylan italicus	1902
Phylan italicus	2001
Phylan italicus	1912
Phylan italicus	1919
Phylan italicus	1910
Phylan italicus	1942
Phylan italicus	1953
Pimelia bipunctata	1996
Pimelia bipunctata	1996
Pimelia bipunctata	1998
Pimelia bipunctata	2001
Pimelia bipunctata	1972
Pimelia bipunctata	2002
Pimelia bipunctata	1986
Pimelia bipunctata	1986
Pimelia bipunctata	1987
Pimelia bipunctata	1987
Pimelia bipunctata	1987
Pimelia bipunctata	1996
Pimelia bipunctata	1996
Pimelia bipunctata	1974
Pimelia bipunctata	1971
Pimelia bipunctata	1949
Pimelia bipunctata	1959
Pimelia bipunctata	1962
Pimelia bipunctata	1981
Pimelia bipunctata	1981
Pimelia bipunctata	1982
Pimelia bipunctata	1979
Pimelia bipunctata	1938
Pimelia bipunctata	1983
Pimelia bipunctata	1983
Pimelia bipunctata	1983
Pimelia bipunctata	1983
Pimelia bipunctata	1983
Pimelia bipunctata	1983
Pimelia bipunctata	1983
Pimelia bipunctata	1983
Pimelia bipunctata	1983
Pimelia bipunctata	1983

Pimelia bipunctata	1983
Pimelia bipunctata	1983
Pimelia bipunctata	1983
Pimelia bipunctata	1983
Pimelia bipunctata	1983
Pimelia bipunctata	1983
Pimelia bipunctata	1983
Pimelia bipunctata	1984
Pimelia bipunctata	1984
Pimelia bipunctata	1901
Pimelia bipunctata	1960
Pimelia bipunctata	1961
Pimelia bipunctata	1976
Pimelia bipunctata	1979
Pimelia bipunctata	1979
Pimelia bipunctata	1979
Pimelia bipunctata	1986
Pimelia bipunctata	1990
Pimelia bipunctata	1918
Pimelia bipunctata	1977
Pimelia bipunctata	1963
Pimelia bipunctata	1968
Pimelia bipunctata	1968
Pimelia bipunctata	1969
Pimelia bipunctata	1977
Pimelia bipunctata	1978
Pimelia bipunctata	1978
Pimelia bipunctata	2001
Pimelia bipunctata	2001
Pimelia bipunctata	2001
Pimelia bipunctata	1981
Pimelia bipunctata	1984
Pimelia bipunctata	1985
Pimelia bipunctata	1986
Pimelia bipunctata	1986
Pimelia bipunctata	1986
Pimelia bipunctata	1986
Pimelia bipunctata	1986
Pimelia bipunctata	1964
Pimelia bipunctata	1958
Pimelia bipunctata	1958
Pimelia bipunctata	1942
Pimelia bipunctata	1977
Pimelia bipunctata	1980
Pimelia bipunctata	1931
Pimelia bipunctata	1936
Pimelia bipunctata	1945
Pimelia bipunctata	1947
Pimelia bipunctata	1992
Pimelia bipunctata	1992
Pimelia bipunctata	1992
Pimelia bipunctata	1989
Pimelia bipunctata	1990
Pimelia bipunctata	1990

Pimelia bipunctata	2001
Pimelia bipunctata	2001
Pimelia bipunctata	2001
Pimelia bipunctata	2001
Pimelia bipunctata	1971
Pimelia bipunctata	1941
Pimelia bipunctata	1942
Pimelia bipunctata	1997
Pimelia bipunctata	1997
Pimelia bipunctata	1997
Pimelia bipunctata	1997
Pimelia bipunctata	1997
Pimelia bipunctata	1997
Pimelia bipunctata	1997
Pimelia bipunctata	1997
Pimelia bipunctata	1998
Pimelia bipunctata	1998
Pimelia bipunctata	1998
Pimelia bipunctata	1998
Pimelia bipunctata	1998
Pimelia bipunctata	1998
Pimelia bipunctata	1998
Pimelia bipunctata	1998
Pimelia bipunctata	1906
Pimelia bipunctata	1923
Pimelia bipunctata	1923
Pimelia bipunctata	1927
Pimelia bipunctata	1969
Pimelia bipunctata	1974
Pimelia bipunctata	1989
Pimelia bipunctata	1994
Pimelia bipunctata	1994
Pimelia bipunctata	1994
Pimelia bipunctata	1996
Pimelia bipunctata	1997
Pimelia bipunctata	2000
Pimelia bipunctata	2000
Pimelia bipunctata	1977
Pimelia bipunctata	1942
Pimelia bipunctata	1988
Pimelia bipunctata	1992
Pimelia bipunctata	1992
Pimelia bipunctata	1979
Pimelia bipunctata	1986
Pimelia bipunctata	1996
Pimelia bipunctata	1997
Pimelia bipunctata	1997
Pimelia bipunctata	1997
Pimelia bipunctata	1997
Pimelia bipunctata	1997
Pimelia bipunctata	1977
Pimelia bipunctata	1977
Pimelia bipunctata	1977
Pimelia bipunctata	1978

Pimelia bipunctata	1978
Pimelia bipunctata	1978
Pimelia bipunctata	1991
Pimelia bipunctata	1992
Pimelia bipunctata	1992
Pimelia bipunctata	1992
Pimelia bipunctata	1992
Pimelia bipunctata	1992
Pimelia bipunctata	1982
Pimelia bipunctata	1931
Pimelia bipunctata	1932
Pimelia bipunctata	1938
Pimelia bipunctata	1938
Pimelia bipunctata	1940
Pimelia bipunctata	1940
Pimelia bipunctata	1940
Pimelia bipunctata	1940
Pimelia bipunctata	1940
Pimelia bipunctata	1940
Pimelia bipunctata	1940
Pimelia bipunctata	1941
Pimelia bipunctata	1941
Pimelia bipunctata	1942
Pimelia bipunctata	1942
Pimelia bipunctata	1942
Pimelia bipunctata	1951
Pimelia bipunctata	1952
Pimelia bipunctata	1952
Pimelia bipunctata	1956
Pimelia bipunctata	1972
Pimelia bipunctata	1980
Pimelia bipunctata	
Platydema europaea	1929
Platydema europaea	1931
Platydema violacea	1986
Platydema violacea	1988
Platydema violacea	1965
Platydema violacea	1989
Platydema violacea	1977
Platydema violacea	1903
Platydema violacea	1994
Platydema violacea	1977
Platydema violacea	1977
Platydema violacea	1991
Platydema violacea	1901
Platydema violacea	1992
Platydema violacea	1996
Platydema violacea	1996
Platydema violacea	1999
Platydema violacea	2001
Platydema violacea	1996
Platydema violacea	1932
Platydema violacea	1908
Platydema violacea	1909

Pseudoseriscius normandi pacificii	1977
Pseudoseriscius normandi pacificii	1977
Pseudoseriscius normandi pacificii	1980
Pseudoseriscius normandi pacificii	1908
Pseudoseriscius normandi pacificii	1910
Pseudoseriscius normandi pacificii	2003
Pseudoseriscius normandi pacificii	1977
Pseudoseriscius normandi pacificii	1983
Pseudoseriscius normandi pacificii	1983
Pseudoseriscius normandi pacificii	1983
Pseudoseriscius normandi pacificii	1983
Pseudoseriscius normandi pacificii	1983
Pseudoseriscius normandi pacificii	1983
Pseudoseriscius normandi pacificii	1983
Pseudoseriscius normandi pacificii	1983
Pseudoseriscius normandi pacificii	1983
Pseudoseriscius normandi pacificii	1983
Pseudoseriscius normandi pacificii	1983
Pseudoseriscius normandi pacificii	1983
Pseudoseriscius normandi pacificii	1984
Pseudoseriscius normandi pacificii	1984
Pseudoseriscius normandi pacificii	1984
Pseudoseriscius normandi pacificii	1963
Pseudoseriscius normandi pacificii	1978
Pseudoseriscius normandi pacificii	1978
Pseudoseriscius normandi pacificii	1995
Pseudoseriscius normandi pacificii	1996
Pseudoseriscius normandi pacificii	1996
Pseudoseriscius normandi pacificii	1965
Pseudoseriscius normandi pacificii	1974
Pseudoseriscius normandi pacificii	1997
Pseudoseriscius normandi pacificii	1997
Pseudoseriscius normandi pacificii	1997
Pseudoseriscius normandi pacificii	1997
Pseudoseriscius normandi pacificii	1997
Pseudoseriscius normandi pacificii	1998
Pseudoseriscius normandi pacificii	1998
Pseudoseriscius normandi pacificii	1998
Pseudoseriscius normandi pacificii	1998
Pseudoseriscius normandi pacificii	1998
Pseudoseriscius normandi pacificii	2004
Scaphidema metallica	1998
Scaphidema metallica	2001
Scaphidema metallica	1998
Scaphidema metallica	1989
Scaphidema metallica	1989
Scaphidema metallica	1989
Scaphidema metallica	1994
Scaphidema metallica	1999
Scaphidema metallica	1999
Scaphidema metallica	1999
Scaphidema metallica	1999
Scaphidema metallica	1918
Scaphidema metallica	1919

Scaphidema metallica	1919
Scaphidema metallica	1986
Scaphidema metallica	1986
Scaphidema metallica	1999
Scaphidema metallica	2000
Scaphidema metallica	1989
Scaurus striatus	1997
Scaurus striatus	1997
Scaurus striatus	1997
Scaurus striatus	2002
Scaurus striatus	1999
Scaurus striatus	1904
Scaurus striatus	1960
Scaurus striatus	1964
Scaurus striatus	1988
Scaurus striatus	1919
Scaurus striatus	1967
Scaurus striatus	1969
Scaurus striatus	1997
Scaurus striatus	1941
Scaurus striatus	1999
Scaurus striatus	1992
Scaurus striatus	1992
Scaurus striatus	1992
Scaurus striatus	1989
Scaurus striatus	1990
Scaurus striatus	1990
Scaurus striatus	1930
Scaurus striatus	1931
Scaurus striatus	1898
Scaurus striatus	1903
Scaurus striatus	1907
Scaurus striatus	1909
Scaurus striatus	1911
Scaurus striatus	1919
Scaurus striatus	1919
Scaurus striatus	1925
Scaurus striatus	1970
Scaurus striatus	1971
Scaurus striatus	1971
Scaurus striatus	1974
Scaurus striatus	1994
Scaurus striatus	1994
Scaurus striatus	1994
Scaurus striatus	1995
Scaurus striatus	1995
Scaurus striatus	1996
Scaurus striatus	1996
Scaurus striatus	1996
Scaurus striatus	1996
Scaurus striatus	1996
Scaurus striatus	1997
Scaurus striatus	1997
Scaurus striatus	1997

Scaurus striatus	1998
Scaurus striatus	1998
Scaurus striatus	1942
Scaurus striatus	1988
Scaurus striatus	1992
Scaurus striatus	1901
Scaurus striatus	1978
Scaurus striatus	2001
Scaurus striatus	2009
Scaurus striatus	2010
Scaurus striatus	2006
Scaurus striatus	1997
Scaurus striatus	1997
Scaurus striatus	1989
Scaurus striatus	1949
Scaurus striatus	1997
Scaurus striatus	1877
Scaurus striatus	1878
Scaurus striatus	1880
Scaurus striatus	1894
Scaurus striatus	1904
Scaurus striatus	1907
Scaurus striatus	1907
Scaurus striatus	1927
Scaurus striatus	1931
Scaurus striatus	1931
Scaurus striatus	1938
Scaurus striatus	1938
Scaurus striatus	1938
Scaurus striatus	1938
Scaurus striatus	1940
Scaurus striatus	1940
Scaurus striatus	1940
Scaurus striatus	1940
Scaurus striatus	1940
Scaurus striatus	1940
Scaurus striatus	1940
Scaurus striatus	1940
Scaurus striatus	1941
Scaurus striatus	1941
Scaurus striatus	1941
Scaurus striatus	1941
Scaurus striatus	1941
Scaurus striatus	1941
Scaurus striatus	1951
Scaurus striatus	1959
Scaurus striatus	1960
Scaurus striatus	1962
Scaurus striatus	1969
Scaurus striatus	1991
Scaurus striatus	1992
Scaurus striatus	1993
Scaurus striatus	1994
Scaurus striatus	1997

Scaurus striatus	1997
Stenomax lanipes	1985
Stenomax lanipes	1985
Stenomax lanipes	1985
Stenomax lanipes	1986
Stenomax lanipes	1986
Stenomax lanipes	1986
Stenomax lanipes	1986
Stenomax lanipes	1986
Stenomax lanipes	1986
Stenomax lanipes	1987
Stenomax lanipes	1987
Stenomax lanipes	1988
Stenomax lanipes	1987
Stenomax lanipes	1987
Stenomax lanipes	1986
Stenomax lanipes	1992
Stenomax lanipes	1988
Stenomax lanipes	1987
Stenomax lanipes	1988
Stenomax lanipes	1988
Stenomax lanipes	1988
Stenomax lanipes	1933
Stenomax lanipes	1969
Stenomax lanipes	1989
Stenomax lanipes	1992
Stenomax lanipes	1975
Stenomax lanipes	1946
Stenomax lanipes	1970
Stenomax lanipes	1937
Stenomax lanipes	1903
Stenomax lanipes	1910
Stenomax lanipes	1912
Stenomax lanipes	1927
Stenomax lanipes	1985
Stenomax lanipes	1989
Stenomax lanipes	1909
Stenomax lanipes	1985
Stenomax lanipes	1997
Stenomax lanipes	1997
Stenomax lanipes	1906
Stenomax lanipes	1908
Stenomax lanipes	1908
Stenomax lanipes	1908
Stenomax lanipes	1913
Stenosis brenthoides brenthoides	1950
Stenosis brenthoides brenthoides	1999
Stenosis brenthoides brenthoides	1959
Stenosis brenthoides brenthoides	1941
Stenosis brenthoides brenthoides	1982
Stenosis brenthoides brenthoides	1983
Stenosis brenthoides brenthoides	1975
Stenosis brenthoides brenthoides	1983
Stenosis brenthoides brenthoides	1984

Stenosis brenthoides brenthoides	1985
Stenosis brenthoides brenthoides	1985
Stenosis brenthoides brenthoides	1985
Stenosis brenthoides brenthoides	1986
Stenosis brenthoides brenthoides	1986
Stenosis brenthoides brenthoides	1986
Stenosis brenthoides brenthoides	1987
Stenosis brenthoides brenthoides	1936
Stenosis brenthoides brenthoides	1937
Stenosis brenthoides brenthoides	1938
Stenosis brenthoides brenthoides	1940
Stenosis brenthoides brenthoides	1941
Stenosis brenthoides brenthoides	1944
Stenosis brenthoides brenthoides	1945
Stenosis brenthoides brenthoides	1945
Stenosis brenthoides brenthoides	1999
Stenosis brenthoides brenthoides	1901
Stenosis brenthoides brenthoides	1995
Stenosis brenthoides brenthoides	1996
Stenosis brenthoides brenthoides	1999
Stenosis brenthoides brenthoides	1928
Stenosis brenthoides brenthoides	1941
Stenosis brenthoides brenthoides	1942
Stenosis brenthoides brenthoides	1943
Stenosis brenthoides brenthoides	1961
Stenosis brenthoides brenthoides	1973
Stenosis brenthoides brenthoides	1979
Stenosis intermedia	1981
Stenosis intermedia	1981
Stenosis intermedia	1981
Stenosis intermedia	1981
Stenosis intermedia	1981
Stenosis intermedia	1981
Stenosis intermedia	1982
Stenosis intermedia	1938
Stenosis intermedia	1963
Stenosis intermedia	1978
Stenosis intermedia	1978
Stenosis intermedia	1978
Stenosis intermedia	1993
Stenosis intermedia	1937
Stenosis intermedia	2001
Stenosis intermedia	2001
Stenosis intermedia	2001
Stenosis intermedia	2001
Stenosis intermedia	1998
Stenosis intermedia	1998
Stenosis intermedia	1998
Stenosis intermedia	1998
Stenosis intermedia	1998
Stenosis intermedia	1977
Stenosis intermedia	1978
Stenosis intermedia	1978
Stenosis intermedia	1978

Stenosis intermedia	1978
Stenosis intermedia	1978
Stenosis intermedia	1978
Stenosis intermedia	1978
Stenosis intermedia	1930
Stenosis intermedia	1978
Stenosis sardoa	1998
Stenosis sardoa	1998
Stenosis sardoa	1996
Stenosis sardoa	1988
Stenosis sardoa	1979
Stenosis sardoa	1985
Stenosis sardoa	1946
Stenosis sardoa	1950
Stenosis sardoa	1969
Stenosis sardoa	1983
Stenosis sardoa	1988
Stenosis sardoa	1999
Stenosis sardoa	1999
Stenosis sardoa	1999
Stenosis sardoa	1999
Stenosis sardoa	1999
Stenosis sardoa	1980
Stenosis sardoa	1982
Stenosis sardoa	1983
Stenosis sardoa	1983
Stenosis sardoa	1995
Stenosis sardoa	1875
Stenosis sardoa	1982
Stenosis sardoa	1982
Stenosis sardoa	1982
Stenosis sardoa	1984
Stenosis sardoa	1984
Stenosis sardoa	1988
Stenosis sardoa	1990
Stenosis sardoa	1990
Stenosis sardoa	1990
Stenosis sardoa	1901
Stenosis sardoa	1901
Stenosis sardoa	1901
Stenosis sardoa	1928
Stenosis sardoa	1936
Stenosis sardoa	1937
Stenosis sardoa	1945
Stenosis sardoa	1946
Stenosis sardoa	1990
Stenosis sardoa	1990
Stenosis sardoa	2001
Stenosis sardoa	2001
Stenosis sardoa	2001
Stenosis sardoa	2001
Stenosis sardoa	2001
Stenosis sardoa	2001
Stenosis sardoa	1971

Stenosis sardoa	1999
Stenosis sardoa	1933
Stenosis sardoa	1941
Stenosis sardoa	1900
Stenosis sardoa	1901
Stenosis sardoa	1969
Stenosis sardoa	1975
Stenosis sardoa	1975
Stenosis sardoa	1995
Stenosis sardoa	1995
Stenosis sardoa	1996
Stenosis sardoa	1998
Stenosis sardoa	1998
Stenosis sardoa	1998
Stenosis sardoa	1999
Stenosis sardoa	1999
Stenosis sardoa	1988
Stenosis sardoa	1989
Stenosis sardoa	1993
Stenosis sardoa	1993
Stenosis sardoa	1984
Stenosis sardoa	1999
Stenosis sardoa	1999
Stenosis sardoa	1999
Stenosis sardoa	2001
Stenosis sardoa	2006
Stenosis sardoa	1997
Stenosis sardoa	1997
Stenosis sardoa	1965
Stenosis sardoa	1999
Stenosis sardoa	1872
Stenosis sardoa	1880
Stenosis sardoa	1894
Stenosis sardoa	1899
Stenosis sardoa	1908
Stenosis sardoa	1933
Stenosis sardoa	1933
Stenosis sardoa	1934
Stenosis sardoa	1934
Stenosis sardoa	1937
Stenosis sardoa	1940
Stenosis sardoa	1940
Stenosis sardoa	1941
Stenosis sardoa	1941
Stenosis sardoa	1942
Tentyria grossa grossa	1996
Tentyria grossa grossa	1996
Tentyria grossa grossa	1996
Tentyria grossa grossa	1998
Tentyria grossa grossa	1998
Tentyria grossa grossa	1998
Tentyria grossa grossa	2001
Tentyria grossa grossa	1972
Tentyria grossa grossa	1974

Tentyria grossa grossa	1987
Tentyria grossa grossa	1995
Tentyria grossa grossa	1973
Tentyria grossa grossa	1974
Tentyria grossa grossa	1912
Tentyria grossa grossa	1977
Tentyria grossa grossa	1977
Tentyria grossa grossa	1952
Tentyria grossa grossa	1996
Tentyria grossa grossa	1949
Tentyria grossa grossa	1962
Tentyria grossa grossa	1981
Tentyria grossa grossa	1981
Tentyria grossa grossa	1982
Tentyria grossa grossa	1974
Tentyria grossa grossa	1978
Tentyria grossa grossa	1978
Tentyria grossa grossa	1961
Tentyria grossa grossa	1964
Tentyria grossa grossa	1976
Tentyria grossa grossa	1979
Tentyria grossa grossa	1980
Tentyria grossa grossa	1988
Tentyria grossa grossa	1986
Tentyria grossa grossa	1936
Tentyria grossa grossa	1963
Tentyria grossa grossa	1966
Tentyria grossa grossa	1967
Tentyria grossa grossa	1969
Tentyria grossa grossa	1978
Tentyria grossa grossa	2001
Tentyria grossa grossa	1982
Tentyria grossa grossa	1974
Tentyria grossa grossa	1986
Tentyria grossa grossa	1986
Tentyria grossa grossa	1988
Tentyria grossa grossa	1988
Tentyria grossa grossa	1989
Tentyria grossa grossa	1989
Tentyria grossa grossa	1989
Tentyria grossa grossa	1990
Tentyria grossa grossa	1990
Tentyria grossa grossa	1992
Tentyria grossa grossa	1941
Tentyria grossa grossa	1977
Tentyria grossa grossa	1980
Tentyria grossa grossa	1985
Tentyria grossa grossa	1936
Tentyria grossa grossa	1937
Tentyria grossa grossa	1982
Tentyria grossa grossa	1990
Tentyria grossa grossa	1990
Tentyria grossa grossa	2001
Tentyria grossa grossa	2001

Tentyria grossa grossa	2001
Tentyria grossa grossa	2001
Tentyria grossa grossa	2001
Tentyria grossa grossa	2001
Tentyria grossa grossa	2002
Tentyria grossa grossa	2002
Tentyria grossa grossa	2002
Tentyria grossa grossa	1972
Tentyria grossa grossa	1990
Tentyria grossa grossa	1941
Tentyria grossa grossa	1954
Tentyria grossa grossa	1903
Tentyria grossa grossa	1904
Tentyria grossa grossa	1909
Tentyria grossa grossa	1911
Tentyria grossa grossa	1913
Tentyria grossa grossa	1929
Tentyria grossa grossa	1968
Tentyria grossa grossa	1972
Tentyria grossa grossa	1974
Tentyria grossa grossa	1974
Tentyria grossa grossa	1974
Tentyria grossa grossa	1991
Tentyria grossa grossa	1992
Tentyria grossa grossa	1994
Tentyria grossa grossa	1995
Tentyria grossa grossa	1995
Tentyria grossa grossa	1995
Tentyria grossa grossa	1995
Tentyria grossa grossa	1996
Tentyria grossa grossa	1997
Tentyria grossa grossa	1997
Tentyria grossa grossa	1998
Tentyria grossa grossa	1942
Tentyria grossa grossa	1988
Tentyria grossa grossa	1989
Tentyria grossa grossa	1992
Tentyria grossa grossa	1994
Tentyria grossa grossa	1995
Tentyria grossa grossa	1977
Tentyria grossa grossa	1979
Tentyria grossa grossa	1997
Tentyria grossa grossa	2001
Tentyria grossa grossa	1997
Tentyria grossa grossa	1997
Tentyria grossa grossa	1997
Tentyria grossa grossa	1997
Tentyria grossa grossa	1997
Tentyria grossa grossa	1998
Tentyria grossa grossa	1969
Tentyria grossa grossa	1975
Tentyria grossa grossa	1911
Tentyria grossa grossa	1977
Tentyria grossa grossa	1977

Tentyria grossa grossa	1978
Tentyria grossa grossa	1978
Tentyria grossa grossa	1978
Tentyria grossa grossa	1992
Tentyria grossa grossa	1992
Tentyria grossa grossa	1992
Tentyria grossa grossa	1907
Tentyria grossa grossa	1908
Tentyria grossa grossa	1931
Tentyria grossa grossa	1931
Tentyria grossa grossa	1931
Tentyria grossa grossa	1938
Tentyria grossa grossa	1940
Tentyria grossa grossa	1940
Tentyria grossa grossa	1940
Tentyria grossa grossa	1940
Tentyria grossa grossa	1940
Tentyria grossa grossa	1940
Tentyria grossa grossa	1940
Tentyria grossa grossa	1940
Tentyria grossa grossa	1940
Tentyria grossa grossa	1940
Tentyria grossa grossa	1941
Tentyria grossa grossa	1941
Tentyria grossa grossa	1941
Tentyria grossa grossa	1942
Tentyria grossa grossa	1942
Tentyria grossa grossa	1942
Tentyria grossa grossa	1952
Tentyria grossa grossa	1953
Tentyria grossa grossa	1959
Tentyria grossa grossa	1974
Tentyria grossa grossa	1978
Tentyria grossa grossa	1978
Tentyria italica	2002
Tentyria italica	1974
Tentyria italica	1945
Tentyria italica	1987
Tentyria italica	1969
Tentyria italica	1936
Tentyria italica	1960
Tentyria italica	1973
Tentyria italica	1983
Tentyria italica	1879
Tentyria italica	1982
Tentyria italica	1983
Tentyria italica	1983
Tentyria italica	1984
Tentyria italica	1984
Tentyria italica	1984
Tentyria italica	1985
Tentyria italica	1987
Tentyria italica	1988
Tentyria italica	1988

Tentyria italica	1988
Tentyria italica	1989
Tentyria italica	1990
Tentyria italica	1931
Tentyria italica	1992
Tentyria italica	1992
Tentyria italica	1992
Tentyria italica	1987
Tentyria italica	1989
Tentyria italica	1988
Tentyria italica	1971
Tentyria italica	1896
Tentyria italica	1898
Tentyria italica	1899
Tentyria italica	1902
Tentyria italica	1903
Tentyria italica	1910
Tentyria italica	1912
Tentyria italica	1914
Tentyria italica	1917
Tentyria italica	1920
Tentyria italica	1921
Tentyria italica	1968
Tentyria italica	1968
Tentyria italica	1983
Tentyria italica	1983
Tentyria italica	1939
Tentyria italica	1992
Tentyria italica	2002
Tentyria italica	1908
Tentyria italica	1909
Tentyria italica	1938
Tentyria italica	1938
Tentyria italica	1938
Tentyria italica	1938
Tentyria italica	1939
Tentyria italica	1940
Tentyria italica	1940
Tentyria italica	1940
Tentyria italica	1940
Tentyria italica	1940
Tentyria italica	1941
Tentyria italica	1941
Tentyria italica	1976
Trachyscelis aphodioides	1960
Trachyscelis aphodioides	1983
Trachyscelis aphodioides	1983
Trachyscelis aphodioides	1983
Trachyscelis aphodioides	1983
Trachyscelis aphodioides	1983
Trachyscelis aphodioides	1983
Trachyscelis aphodioides	1983
Trachyscelis aphodioides	1983
Trachyscelis aphodioides	1983

Trachyscelis aphodioides	1983
Trachyscelis aphodioides	1983
Trachyscelis aphodioides	1983
Trachyscelis aphodioides	1983
Trachyscelis aphodioides	1983
Trachyscelis aphodioides	1984
Trachyscelis aphodioides	1984
Trachyscelis aphodioides	1900
Trachyscelis aphodioides	1963
Trachyscelis aphodioides	1978
Trachyscelis aphodioides	1978
Trachyscelis aphodioides	1966
Trachyscelis aphodioides	1913
Trachyscelis aphodioides	1988
Trachyscelis aphodioides	1901
Uloma culinaris	1997
Uloma culinaris	1998
Uloma culinaris	1998
Uloma culinaris	2001
Uloma culinaris	1974
Uloma culinaris	1958
Uloma culinaris	1967
Uloma culinaris	1989
Uloma culinaris	1989
Uloma culinaris	1983
Uloma culinaris	1939
Uloma culinaris	1982
Uloma culinaris	1936
Uloma culinaris	1909
Uloma culinaris	1910
Uloma culinaris	1913
Uloma culinaris	1927
Uloma culinaris	1994
Uloma culinaris	1994
Uloma culinaris	1995
Uloma culinaris	1995
Uloma culinaris	1995
Uloma culinaris	1996
Uloma culinaris	1996
Uloma culinaris	1996
Uloma culinaris	1996
Uloma culinaris	1997
Uloma culinaris	1998
Uloma culinaris	1999
Uloma culinaris	1999
Uloma culinaris	2000
Uloma culinaris	2000
Uloma culinaris	1964
Uloma culinaris	2001
Uloma culinaris	1948
Uloma culinaris	1993
Uloma culinaris	1999
Uloma culinaris	1907
Uloma culinaris	1908

Uloma culinaris	1908
Uloma culinaris	1932
Uloma culinaris	1932
Xanthomus pallidus	1978
Xanthomus pallidus	1983
Xanthomus pallidus	1983
Xanthomus pallidus	1983
Xanthomus pallidus	1984
Xanthomus pallidus	1971
Xanthomus pallidus	1958
Xanthomus pallidus	1976
Xanthomus pallidus	1998
Xanthomus pallidus	2000
Xanthomus pellucidus	1978
Xanthomus pellucidus	1978
Xanthomus pellucidus	1980
Xanthomus pellucidus	1983
Xanthomus pellucidus	1983
Xanthomus pellucidus	1983
Xanthomus pellucidus	1983
Xanthomus pellucidus	1983
Xanthomus pellucidus	1983
Xanthomus pellucidus	1983
Xanthomus pellucidus	1983
Xanthomus pellucidus	1983
Xanthomus pellucidus	1983
Xanthomus pellucidus	1983
Xanthomus pellucidus	1983
Xanthomus pellucidus	1984
Xanthomus pellucidus	1984
Xanthomus pellucidus	1984
Xanthomus pellucidus	1984
Xanthomus pellucidus	1988
Xanthomus pellucidus	1977
Xanthomus pellucidus	1977
Xanthomus pellucidus	1977
Xanthomus pellucidus	1978
Xanthomus pellucidus	1978
Xanthomus pellucidus	1978
Xanthomus pellucidus	1994
Xanthomus pellucidus	1995
Xanthomus pellucidus	1995
Xanthomus pellucidus	1997
Xanthomus pellucidus	1997
Xanthomus pellucidus	1997
Xanthomus pellucidus	1998
Xanthomus pellucidus	1998
Xanthomus pellucidus	1998
Xanthomus pellucidus	1998
Xanthomus pellucidus	1999
Xanthomus pellucidus	1999
Xanthomus pellucidus	1913
Xanthomus pellucidus	1998

Collector	Specimens
A. Biscaccianti	3
A. Biscaccianti	3
A. Biscaccianti	1
A. Ciceroni	1
A. Ciceroni	2
A. Ciceroni	5
A. Ciceroni	5
A. Ciceroni	1
A. Ciceroni	1
A. Ciceroni	3
A. Ciceroni	1
A. Ciceroni	6
A. Ciceroni	1
A. Ciceroni	5
A. Ciceroni	4
A. Ciceroni	12
A. Ciceroni	4
A. Ciceroni	2
A. Ciceroni	1
A. Ciceroni	7
A. Ciceroni	12
A. Ciceroni	1
A. Ciceroni	9
A. Ciceroni	1
A. Ciceroni	1
A. Ciceroni	2
A. Ciceroni	5
A. Ciceroni	1
A. Ciceroni	1
A. Ciceroni	5
A. Ciceroni	1
A. Ciceroni	3
A. Ciceroni	1
A. Ciceroni	9
A. Ciceroni	1
A. Ciceroni	13
A. Ciceroni	41
A. Ciceroni	27
A. Ciceroni	6
A. Ciceroni	15
A. Ciceroni	6
A. Ciceroni	1
A. Ciceroni	9
A. Ciceroni	22
A. Ciceroni	10
A. Ciceroni	2
A. Ciceroni	1
A. Di Giulio	1
A. Liuti & B. Labella	2
A. Mazziotta	2
A. Vigna Taglianti & De Liberato	1
A. Vigna Taglianti & De Liberato	1

A. Vigna Taglianti & De Liberato	1
A. Vigna Taglianti & De Liberato	1
A. Vigna Taglianti & De Liberato	2
A. Vigna Taglianti & De Liberato	1
A. Vigna Taglianti & De Liberato	3
A. Vigna Taglianti & De Liberato	3
A. Vigna Taglianti & De Liberato	1
Angeloni & Vigna	1
Angeloni & Vigna	2
Angeloni & Vigna	9
Angeloni & Vigna	1
Angeloni & Vigna	2
Angeloni & Vigna	1
Angeloni & Vigna	2
Angeloni & Vigna	2
Angeloni & Vigna	2
Angeloni & Vigna	2
Angeloni & Vigna	1
Angeloni & Vigna	2
C. Consiglio	2
Calvario & Ruvolo	1
Calvario & Ruvolo	1
Calvario & Ruvolo	1
Calvario & Ruvolo	1
Calvario & Ruvolo	1
Calvario & Ruvolo	1
Calvario & Ruvolo	4
Calvario & Ruvolo	1
Calvario & Ruvolo	3
Calvario & Ruvolo	4
Calvario & Ruvolo	2
Calvario & Ruvolo	4
Calvario & Ruvolo	1
Cecchetti & A. Vigna	3
Cecchetti & A. Vigna	10
Cecchetti & A. Vigna	1
Cecchetti & A. Vigna	4
Cecchetti & A. Vigna	7
Cecchetti & A. Vigna	10
Cecchetti & A. Vigna	4
Cotta	3
Damazia	2
Damazia	1
E. Colonnelli	2
E. Improta	1
E. Improta	2
E. Improta	1
E. Improta	2
E. Improta	2
E. Improta	2
E. Improta	1
E. Improta	2
E. Improta	3
E. Improta	1

E. Improta	1
E. Improta	2
E. Improta	3
E. Improta	3
E. Improta	2
E. Improta	1
E. Improta	1
E. Migliaccio	4
E. Migliaccio	2
E. Migliaccio	4
E. Migliaccio	7
E. Migliaccio	1
F. Cechetti	1
F. Comandini	1
F. Comandini	3
F. Comandini	1
F. Comandini	1
F. Comandini	1
F. Comandini	1
F. Comandini	3
F. Comandini	1
F. Comandini	1
F. Comandini	1
F. Comandini	1
F. Comandini	2
F. Comandini	2
F. Comandini	1
F. Comandini	8
F. Pelloni	2
F. Pelloni	4
F. Pelloni	5
F. Pelloni	3
F. Pelloni	2
F. Pelloni	2
F. Pelloni	4
F. Pelloni	6
F. Pelloni	4
F. Simmi	1
F.lli Garavaglia	3
G. Di Giulio	3
G. Di Giulio	1
G. Di Giulio	2
G. Di Giulio	2
G. Di Stefano	1
G. Di Stefano, C. Tronci & E. Romer	6
G. Franzini	3
G. Nardi	5
G. Papini & R. Magrone	18
Ingrao	1
L. Tizi	2
M. Bologna	3
M. Bologna	1
M. Cerruti	1
M. Cerruti	1

M. de Mei	1
M. de Mei	6
M. de Mei	2
M. de Mei	4
M. de Mei	2
M. de Mei	1
M. Mei	2
M. Mei	2
M. Panella	2
M. Pitzalis & E. Trucchi	17
M. Pitzalis & E. Trucchi	1
M. Pitzalis & E. Trucchi	3
M. Pitzalis & E. Trucchi	1
M. Zapparoli	8
P. Angelini	1
P. Angelini. A.Di Giulio & A. Vigna	2
P. Audisio	2
P. Barbieri	1
P. Crucitti	1
P. Crucitti	5
P. Luigioni	4
P. Luigioni	1
P. Luigioni	1
P. Maltzeff	1
P. Maltzeff	1
P. Maltzeff	1
P. Maltzeff	1
P. Maltzeff	3
P. Maltzeff	2
P. Maltzeff	3
P. Maltzeff	3
P. Maltzeff	3
P. Maltzeff	2
P. Maltzeff	3
P. Maltzeff	2
P. Maltzeff	3
P. Maltzeff	5
P. Maltzeff	3
P. Maltzeff	1
P. Maltzeff	1
P. Maltzeff	2
Pedullà, Rellori & Vigna	1
Pedullà, Rellori & Vigna	2
Pedullà, Rellori & Vigna	1
Pedullà, Rellori & Vigna	4
Pedullà, Rellori & Vigna	4
Pedullà, Rellori & Vigna	8
Pedullà, Rellori & Vigna	3
Pedullà, Rellori & Vigna	12
Pedullà, Rellori & Vigna	14
Pedullà, Rellori & Vigna	2
Pedullà, Rellori & Vigna	1
Pedullà, Rellori & Vigna	2
Pedullà, Rellori & Vigna	1

Pedullà, Rellori & Vigna	7
Pedullà, Rellori & Vigna	5
Pedullà, Rellori & Vigna	9
Pedullà, Rellori & Vigna	1
Pedullà, Rellori & Vigna	1
Pedullà, Rellori & Vigna	3
Pedullà, Rellori & Vigna	5
Pedullà, Rellori & Vigna	2
Pedullà, Rellori & Vigna	2
Pedullà, Rellori & Vigna	1
Pedullà, Rellori & Vigna	1
Pedullà, Rellori & Vigna	1
Pedullà, Rellori & Vigna	1
Pedullà, Rellori & Vigna	4
Pedullà, Rellori & Vigna	7
Pedullà, Rellori & Vigna	5
Pedullà, Rellori & Vigna	15
Pedullà, Rellori & Vigna	12
Pedullà, Rellori & Vigna	2
Pedullà, Rellori & Vigna	5
Pedullà, Rellori & Vigna	2
Pedullà, Rellori & Vigna	10
Pedullà, Rellori & Vigna	9
Pedullà, Rellori & Vigna	1
Pedullà, Rellori & Vigna	4
Pedullà, Rellori & Vigna	6
Pedullà, Rellori & Vigna	5
Pedullà, Rellori & Vigna	5
Pedullà, Rellori & Vigna	19
Pedullà, Rellori & Vigna	5
Pedullà, Rellori & Vigna	4
Pedullà, Rellori & Vigna	4
Pedullà, Rellori & Vigna	7
Pedullà, Rellori & Vigna	1
Pedullà, Rellori & Vigna	2
Pedullà, Rellori & Vigna	6
Pedullà, Rellori & Vigna	2
Pedullà, Rellori & Vigna	7
Pedullà, Rellori & Vigna	2
Pedullà, Rellori & Vigna	1
Pedullà, Rellori & Vigna	3
Pedullà, Rellori & Vigna	2
Pedullà, Rellori & Vigna	9
Pedullà, Rellori & Vigna	1
Pedullà, Rellori & Vigna	2
Pedullà, Rellori & Vigna	17
Pedullà, Rellori & Vigna	1
Pedullà, Rellori & Vigna	1
Pedullà, Rellori & Vigna	2
Pedullà, Rellori & Vigna	5
Pedullà, Rellori & Vigna	8
Pedullà, Rellori & Vigna	7
Pedullà, Rellori & Vigna	4
Pedullà, Rellori & Vigna	8

Pedullà, Rellori & Vigna	9
Pedullà, Rellori & Vigna	1
Pedullà, Rellori & Vigna	3
Pedullà, Rellori & Vigna	1
Pedullà, Rellori & Vigna	1
Pedullà, Rellori & Vigna	9
Pedullà, Rellori & Vigna	6
Pedullà, Rellori & Vigna	4
Pedullà, Rellori & Vigna	3
Pedullà, Rellori & Vigna	16
Pischedda	1
Prola	2
Prola	1
R. Antonelli	3
R. Antonelli	2
R. Antonelli	2
R. Mignani	16
S. de Felici	1
S. de Felici	7
S. de Felici	2
S. de Felici	1
S. de Felici	1
S. de Felici	2
S. de Felici	12
S. de Felici	9
S. de Felici	1
S. de Felici	1
S. de Felici	1
S. de Felici	6
S. de Felici	2
S. de Felici	4
S. de Felici	1
S. de Felici	2
S. de Felici	2
S. de Felici	3
S. de Felici	13
S. de Felici	22
S. de Felici	2
S. de Felici	3
S. de Felici	14
S. de Felici	24
S. de Felici	5
S. de Felici	1
S. de Felici	1
S. de Felici	1
S. Fattorini	12
S. Fattorini	2
S. Fattorini	2
S. Fattorini	2
S. Fattorini	1
S. Fattorini	4
S. Fattorini	2
S. Fattorini	2
S. Fattorini	5

S. Fattorini	1
S. Fattorini	7
S. Fattorini	3
S. Fattorini & F. Vicari	2
S. Fattorini & G. Di Stefano	2
S. Fattorini & G. Di Stefano	35
S. Fattorini & G. Di Stefano	1
S. Fattorini, G. Di Stefano & E. Rom	3
S. Fattorini, S. & G. Di Stefano	2
S. Maugeri	1
S. Patrizi & M. Cerruti	1
Tripaldi & Vigna Taglianti	1
Tripaldi & Vigna Taglianti	3
Tripaldi & Vigna Taglianti	1
V. Sbordoni	5
V. Sbordoni	1
Vigna Taglianti & Lanci	1
Vigna Taglianti & Lanci	1
Vigna Taglianti & Lanci	5
Vigna Taglianti & Lanci	1
Vigna Taglianti & Lanci	1
Vigna Taglianti & Lanci	4
Vigna Taglianti & Lanci	2
Vigna Taglianti & Lanci	3
Vigna Taglianti & Lanci	1
Vigna Taglianti & Lanci	2
Vigna Taglianti & Lanci	2
Vigna Taglianti & Lanci	2
Vigna Taglianti & Lanci	6
Vigna Taglianti & Lanci	1
Vigna Taglianti & Lanci	1
Vigna Taglianti & Lanci	1
Vigna Taglianti & Lanci	4
Vigna Taglianti & Lanci	6
Vigna Taglianti & Lanci	8
	2
	1
	1
	1
	1
	1
	1
	2
	1
	3
	2
	1
	2
	2
	1
	2
	1
	2
	3

	1
	11
	1
	1
	1
	1
A. Biscaccianti	3
A. Di Giulio	2
A. Di Giulio, S. Di Giulio, C. Baroni	2
Bisecchi	1
Brunelli	1
C. Consiglio	1
C. Consiglio	1
D. Baiocchi	31
E. De Maggi	1
E. De Maggi	1
E. Migliaccio	10
E. Migliaccio	1
E. Migliaccio	1
F. Simmi	1
G. Carpaneto	1
G. Carpaneto	1
G. Carpaneto	3
G. Carpaneto	1
G. Castellini	2
G. Di Giulio	1
G. Di Stefano	2
G. Nardi	1
G. Nardi	2
M. Cerruti	5
M. Cerruti	1
M. Mei	1
M. Mei	1
M. Mei	9
M. Zapparoli	1
P. Leo	26
P. Luigioni	1
P. Luigioni	1
P. Luigioni	1
P. Luigioni	6
P. Luigioni	1
P. Luigioni	2
P. Maltzeff	2
P. Maltzeff	4
P. Maltzeff	1
P. Maltzeff	1
P. Maltzeff	1
Prola	1
Prola	1
R. Antonelli	2
R. Consorti	1
R. Consorti	1
R. Consorti	4
R. Consorti	2

S. & S. Fattorini	1
S. Fattorini	1
S. Fattorini	5
S. Fattorini	20
S. Fattorini	5
S. Fattorini	2
S. Fattorini	5
S. Fattorini & F. Fedele	2
S. Nardi	1
S. Nardi	1
S. Nardi	1
S. Nardi	1
S. Nardi	1
Sesino	2
U. Pessolano	2
U. Pessolano	1
U. Pessolano	2
V. Viglioglia	1
	1
	1
	1
	1
	1
	1
	1
	1
	1
	2
	1
	2
	1
	2
	4
	1
	1
	1
	1
	1
	1
	1
	1
	2
	1
	1
	1
	1
	1
	2
A. Pacifici	1
A. Pacifici	4
A. Tirelli	1
E. Migliaccio	1
G. Di Giulio	1
M. Cerruti	2

M. Cerruti	2
M. Cerruti	4
M. Mei	1
P. Luigioni	1
P. Luigioni	1
P. Luigioni	1
P. Luigioni	1
P. Luigioni	1
P. Luigioni	4
P. Luigioni	1
P. Luigioni	1
P. Luigioni	2
P. Luigioni	1
P. Maltzeff	1
P. Maltzeff	3
P. Maltzeff	7
P. Maltzeff	2
R. Montali	1
R. Montali	1
R. Montali	1
R. Montali	1
S. Fattorini & F. Fedele	1
S. Nardi	1
Sesino	1
Sesino	1
	1
	1
	1
	1
	3
	1
	1
	1
	1
	1
	1
	1
	1
	1
Ciacchi	9
M. Zampetti	1
M. Zampetti	2
Anguissola	1
Ciacchi	1
D. Cagiotti & L. Bertolaccini	756
D. Cagiotti & L. Bertolaccini	678
D. Cagiotti & L. Bertolaccini	467
D. Cagiotti & L. Bertolaccini	266
D. Cagiotti & L. Bertolaccini	282
D. Cagiotti & L. Bertolaccini	365
D. Cagiotti & L. Bertolaccini	344
D. Cagiotti & L. Bertolaccini	383
D. Cagiotti & L. Bertolaccini	364
D. Cagiotti & L. Bertolaccini	321

D. Cagiotti & L. Bertolaccini	98
D. Cagiotti & L. Bertolaccini	201
D. Cagiotti & L. Bertolaccini	181
D. Cagiotti & L. Bertolaccini	134
D. Cagiotti & L. Bertolaccini	140
D. Cagiotti & L. Bertolaccini	424
D. Cagiotti & L. Bertolaccini	421
D. Cagiotti & L. Bertolaccini	297
D. Cagiotti & L. Bertolaccini	282
D. Cagiotti & L. Bertolaccini	98
D. Cagiotti & L. Bertolaccini	225
D. Cagiotti & L. Bertolaccini	274
D. Cagiotti & L. Bertolaccini	258
D. Cagiotti & L. Bertolaccini	154
E. Colonnelli	4
E. Colonnelli	4
E. Migliaccio	1
E. Pacieri	3
G. Binaghi	100
G. Carpaneto	11
G. Carpaneto	19
G. Carpaneto	2
G. Carpaneto	10
G. Nardi	6
G. Sabatinelli	5
L. Picco	1
P. De Salvo	6
P. De Salvo	13
P. De Salvo	6
P. De Salvo	7
P. De Salvo	10
P. De Salvo	13
P. De Salvo	14
P. De Salvo	16
P. De Salvo	19
P. De Salvo	19
P. De Salvo	5
P. De Salvo	8
P. De Salvo	15
P. De Salvo	8
P. De Salvo	10
P. De Salvo	10
P. De Salvo	20
P. De Salvo	35
P. De Salvo	12
P. De Salvo	23
P. De Salvo	18
P. De Salvo	15
P. De Salvo	4
P. De Salvo	8
S. Fattorini	1
S. Fattorini & G. Di Stefano	26
S. Fattorini & G. Di Stefano	33
S. Fattorini & G. Di Stefano	7

S. Patrizi	2
A. Tirelli	2
Angeloni & Vigna	2
Angeloni & Vigna	1
Angeloni & Vigna	1
Angeloni & Vigna	2
Calvario & Ruvolo	1
Calvario & Ruvolo	1
Calvario & Ruvolo	1
Calvario & Ruvolo	2
E. Migliaccio	1
E. Migliaccio	1
E. Migliaccio	1
G. Pace	1
L. Picco	1
Lombardi	1
M. & G. Osella	2
M. Mei	1
M. Zampetti	1
S. De Felici	2
S. De Felici	8
S. De Felici	13
S. De Felici	1
Tripaldi & Vigna Taglianti	1
Tripaldi & Vigna Taglianti	1
Tripaldi & Vigna Taglianti	2
Tripaldi & Vigna Taglianti	1
Tripaldi & Vigna Taglianti	1
Tripaldi & Vigna Taglianti	4
Tripaldi & Vigna Taglianti	1
Tripaldi & Vigna Taglianti	3
Tripaldi & Vigna Taglianti	6
Tripaldi & Vigna Taglianti	3
Tripaldi & Vigna Taglianti	3
Tripaldi & Vigna Taglianti	1
Tripaldi & Vigna Taglianti	1
	1
A. Biscaccianti & Facchinelli	1
A. Focarile	57
A. Focarile	5
A. Pacifici	20
A. Tirelli	1
A. Tirelli	1
A. Tirelli	5
Calvario & Ruvolo	1
Calvario & Ruvolo	1
Calvario & Ruvolo	1
E. Colonnelli	1
E. Improta	4
E. Improta	1
E. Improta	1
E. Improta	1
E. Improta	1
E. Improta	18

E. Improta	6
E. Improta	13
E. Improta	9
E. Improta	5
F. Comandini	2
F. Comandini	1
F. Comandini	12
F. Comandini	1
F. Comandini	1
F. Comandini	4
F. Comandini	3
F. Comandini	2
F. Comandini	3
F. Comandini	6
F. Comandini	8
F. Pelloni	4
F. Pelloni	1
F. Pelloni	3
F. Pelloni	1
F. Pelloni	1
F. Silvestri	1
G. Di Giulio	1
G. Di Giulio	2
G. Doria	6
G. Nardi	6
G. Nardi	12
G. Pace	1
G. Pace	2
G. Papini	1
G. Papini	1
Grimm	1
M. Burlini	2
M. Cerruti	1
M. Cerruti	1
M. Cerruti	3
M. De Mei	2
M. De Mei	1
M. De Mei	3
M. De Mei	10
M. De Mei	5
M. De Mei	5
M. De Mei	1
M. De Mei	6
O. Castellani	1
P. Luigioni	1
P. Luigioni	1
P. Luigioni	1
P. Luigioni	1
P. Luigioni	1
P. Maltzeff	1
P. Maltzeff	1
P. Maltzeff	1
P. Maltzeff	1
P. Maltzeff	1

P. Maltzeff	1
P. Maltzeff	1
P. Maltzeff	1
P. Maltzeff	1
P. Maltzeff	2
P. Maltzeff	4
P. Maltzeff	2
P. Maltzeff	1
P. Maltzeff	1
P. Maltzeff	3
P. Maltzeff	2
P. Maltzeff	1
P. Maltzeff	3
P. Maltzeff	1
P. Maltzeff	1
P. Maltzeff	1
P. Maltzeff	2
P. Maltzeff	1
P. Maltzeff	3
P. Maltzeff	1
P. Maltzeff	1
P. Maltzeff	1
P. Maltzeff	1
P. Maltzeff	4
P. Maltzeff	2
P. Maltzeff	1
P. Maltzeff	2
R. Antonelli	1
S. De Felici	2
S. De Felici	1
S. Fattorini	1
S. Fattorini	1
S. Fattorini	1
S. Fattorini	1
S. Patrizi & M. Cerruti	1
	2
	1
	1
	4
	1
	2
	1
	1
A. Vigna Taglianti	1
B. Osella	5
B. Osella	5
B. Osella	3
B. Osella	9
B. Osella	9
Cappelletti	2
Di Marco,Trivellane & Stagnini	3
G. Pace	1
M. Bologna & B. Di Gaetano	1
M. Cerruti	1

Malatesta	2
P. Luigioni & A. Tirelli	1
Pischedda	1
R. Antonelli	1
Sclano	2
Sclano	1
Sclano	2
Sclano	2
Trella & Vacca	1
	2
	4
	2
	7
A. Liberto	1
A. Pacifici	6
A. Parenti	1
A. Tirelli	7
A. Tirelli	6
C. Bellò	3
E. Migliaccio	1
E. Migliaccio	1
E. Migliaccio	1
E. Migliaccio	3
G. Franzini	3
G. Montelli	1
G. Pace	2
G. Pace	1
M. Bologna & B. Di Gaetano	1
P. Luigioni	2
P. Luigioni	1
P. Luigioni	2
P. Luigioni	4
P. Luigioni & A. Tirelli	1
R. Antonelli	1
R. Antonelli	2
	6
	1
	3
	1
	1
	1
	4
	2
Di Gaetano & B. Osella	1
G. Lepri	1
G. Pace	2
G. Pace	1
Giannelli	1
Lombardi	2
M. Zampetti	1
P. Angelini	1
P. Audisio	1
P. Bensa	1
P. Luigioni	1

P. Luigioni	1
	1
	2
	1
	3
	23
	11
	1
	1
A. Biscaccianti	1
A. Biscaccianti	1
A. Biscaccianti	1
A. Biscaccianti	1
A. Di Giulio	1
A. Di Giulio & M. Bologna	1
A. Di Giulio, S. Di Giulio, C. Baroni	2
A. Liuti & B. Labella	1
A. Liuti & B. Labella	1
Berardi	1
C. Consiglio	1
C. Consiglio	1
C. Sabbatini	1
C. Sabbatini	2
C. Tronci	3
Calvario & Ruvolo	1
Cotta	1
D. Baiocchi	36
E. De Maggi	1
E. Improta	2
E. Improta	2
E. Improta	2
E. Migliaccio	1
E. Romero	1
F. Caruso	1
F. Pelloni	44
F. Pelloni	18
F. Pelloni	15
F. Simmi	1
G. Arcà	2
G. Carpaneto	1
G. Carpaneto	1
G. Carpaneto	1
G. Carpaneto	4
G. Carpaneto	3
G. Carpaneto	2
G. Carpaneto	2
G. Carpaneto	2
G. Di Giulio	1
G. Di Stefano	2
G. Di Stefano	2
G. Di Stefano, C. Tronci & E. Romer	1
G. Nardi	1
G. Nardi	1
G. Nardi	1

G. Nardi	1
G. Nardi	4
G. Nardi	1
G. Nardi	1
G. Nardi	1
G. Nardi	2
G. Nardi	2
G. Nardi	1
G. Nardi	11
G. Nardi	1
G. Nardi	1
G. Nardi	1
G. Pavia	3
L. Tizi	2
Lucidirdehw	1
M. Bologna	2
M. Cerruti	5
M. Fraschetti	1
M. Fraschetti	1
M. Fraschetti	1
M. Mei	11
M. Mei	1
M. Mei	11
M. Mei	1
M. Panella	1
M. Zapparoli	2
M. Zapparoli & P. Jonales	1
M. Zapparoli & P. Jonales	5
M. Zapparoli & P. Jonales	1
M. Zapparoli & P. Jonales	2
M. Zapparoli & P. Jonales	1
M. Zapparoli & P. Jonales	2
M. Zapparoli & P. Jonales	2
O. Castellani	3
P. Leo	18
P. Leo	1
P. Luigioni	1
P. Luigioni	1
P. Luigioni	1
P. Maltzeff	1
P. Maltzeff	1
P. Maltzeff	1
P. Maltzeff	1
P. Maltzeff	1
P. Maltzeff	1
P. Maltzeff	1
P. Maltzeff	1
P. Maltzeff	1
P. Maltzeff	1
P. Maltzeff	1
P. Maltzeff	1
P. Maltzeff	1
P. Maltzeff	1
P. Maltzeff	1

P. Maltzeff	1
P. Maltzeff	8
P. Maltzeff	1
Prola	2
R. Antonelli	1
R. Antonelli	1
S. & G. Di Stefano	1
S. Fattorini	3
S. Fattorini	1
S. Fattorini	2
S. Fattorini	15
S. Fattorini	1
S. Fattorini	1
S. Fattorini	1
S. Fattorini	2
S. Fattorini	3
S. Fattorini	1
S. Fattorini	1
S. Fattorini	1
S. Fattorini	2
S. Fattorini & G. Di Stefano	3
S. Fattorini & G. Di Stefano	2
S. Fattorini & G. Di Stefano	1
S. Fattorini & G. Di Stefano	1
S. Fattorini, S. & G. Di Stefano	2
S. Nardi	1
S. Nardi	1
S. Nardi	1
S. Nardi	1
S. Nardi	1
S. Nardi	1
S. Nardi	1
S. Nardi	1
S. Nardi	1
S. Nardi	1
S. Nardi	1
S. Nardi	4
S. Nardi	3
S. Nardi	3
S. Nardi	2
S. Nardi	2
S. Nardi	2
S. Nardi	2
S. Nardi	1
S. Nardi	1
Tripaldi	1
Tripaldi & Vigna Taglianti	1
U. Pessolano	1
U. Pessolano	1
U. Pessolano	1
U. Pessolano	1
U. Pessolano	1
U. Pessolano	2
U. Pessolano	1

U. Pessolano	1
V. Sbordoni	5
V. Viglioglia	1
	1
	1
	1
	1
	1
	1
	1
	4
	2
	1
	1
	2
	1
	2
	1
	1
	3
	1
	1
	1
	1
	1
	1
	1
	2
	1
	2
	2
	2
	1
	1
	1
Berardi	1
E. Migliaccio	17
G. Carpaneto	1
Gressi	2
M. Cerruti	1
M. Cerruti	4
P. leo	1
P. Luigioni	7
P. Maltzeff	1
	1
	1
	1
	1
	1
Bolasco	1
F. Tassi	1
G. Carpaneto	1
G. Nardi	1

M. Zampetti	1
P. Luigioni	1
	2
	1
C. Utzeri	1
G. Nardi	1
G. Papini	1
M. Mei	1
P. Leo	11
P. Leo	1
P. Luigioni	2
P. Maltzeff	1
Prola	1
S. & S. Fattorini	5
S. Fattorini	2
S. Fattorini	2
S. Fattorini & F. Fedele	1
S. Fattorini & F. Fedele	1
S. Fattorini & G. Di Stefano	1
V. Viglioglia	1
	1
	1
	1
	1
	1
A. Dodero	1
P. Luigioni	1
P. Luigioni	3
P. Luigioni	3
C. Esposito	4
C. Esposito	11
E. Migliaccio	4
G. Nardi	3
G. Nardi	6
G. Pace	2
P. Luigioni	2
P. Maltzeff	1
P. Maltzeff	1
P. Maltzeff	1
	3
	2
	8
	2
	1
	1
	1
	1
	1
A. Biscaccianti	1
A. Biscaccianti	1
A. Biscaccianti	2
A. Ceccarelli	1
A. Ciceroni	1
A. Pacifici	21

C. Consiglio	1
C. Consiglio	1
C. Consiglio	1
C. Consiglio	4
C. Consiglio	1
C. Consiglio	2
C. Consiglio	1
C. Consiglio	4
C. Consiglio	1
C. Consiglio	2
C. Consiglio	1
Calvario & Ruvolo	1
Calvario & Ruvolo	1
D. Baiocchi	2
D. Baiocchi	2
E. Colonnelli	2
E. Colonnelli	3
E. Colonnelli	1
E. Migliaccio	1
E. Migliaccio	4
E. Migliaccio	1
E. Migliaccio	2
E. Migliaccio	2
E. Migliaccio	2
E. Migliaccio	1
E. Migliaccio	1
E. Migliaccio	2
E. Migliaccio	3
E. Pacieri	3
F. Comandini	3
F. Comandini	1
F. Hartig	2
G. Carpaneto	2
G. Di Giulio	4
G. Di Giulio	2
G. Di Giulio	8
G. Nardi	7
G. Nardi	1
G. Nardi	2
G. Nardi	2
G. Nardi	7
G. Nardi	23
L. Bertolaccini	1
M. Bologna	1
M. Bologna	1
M. Cerruti	1
M. Cerruti	1
M. Cerruti	4
M. de Mei	1
M. Mei	1
M. Pitzalis & E. Trucchi	2
M. Pitzalis & E. Trucchi	1
O. Castellani	8
P. Audisio	1

P. Audisio	1
P. Bonavita & A. Vigna Taglianti	1
P. De Salvo	1
P. Maltzeff	3
P. Maltzeff	1
P. Maltzeff	1
P. Maltzeff	1
P. Maltzeff	2
P. Maltzeff	1
P. Maltzeff	2
P. Maltzeff	2
P. Maltzeff	2
Prola	1
R. Antonelli	2
R. Antonelli	1
R. Antonelli	6
S. Cafaro	4
S. Fattorini	1
S. Fattorini	6
S. Patrizi & M. Cerruti	1
Tripaldi & Vigna Taglianti	3
Tripaldi & Vigna Taglianti	3
Tripaldi & Vigna Taglianti	1
Tripaldi & Vigna Taglianti	1
Tripaldi & Vigna Taglianti	1
Tripaldi & Vigna Taglianti	1
Tripaldi & Vigna Taglianti	1
Tripaldi & Vigna Taglianti	1
	2
	1
	1
	1
	2
	1
	1
	1
	1
	1
	3
	1
	1
	1
	2
	1
	2
	7
	1
A. Pacifici	1
A. Pacifici	1
M. Mariani	1
M. Pitzalis & E. Trucchi	1
P. Luigioni	1
	1
	2

	2
E. Migliaccio	3
G. Pace	1
G. Pace	1
Sclano	1
	1
A. Biscaccianti	2
A. Biscaccianti	1
A. Ciceroni	1
A. Ciceroni	1
A. Ciceroni	1
A. Ciceroni	3
A. Ciceroni	1
A. Ciceroni	2
A. Letardi & De Liberato	3
A. Pacifici	1
A. Tirelli	1
A. Tirelli	1
A. Tirelli	1
A. Tirelli	1
A. Tirelli	5
A. Tirelli	2
Angeloni & Vigna	2
Angeloni & Vigna	1
Angeloni & Vigna	1
Angeloni & Vigna	1
Angeloni & Vigna	12
Angeloni & Vigna	3
Angeloni & Vigna	3
Angeloni & Vigna	7
Angeloni & Vigna	15
Angeloni & Vigna	1
Angeloni & Vigna	3
Angeloni & Vigna	68
Angeloni & Vigna	2
Calvario & Ruvolo	24
Cecchetti & A. Vigna	5
Cecchetti & A. Vigna	5
Cecchetti & A. Vigna	5
D. Baiocchi	1
E. Colonnelli	1
E. Improta	2
E. Improta	3
E. Improta	36
E. Improta	1
E. Improta	6
E. Improta	3
E. Improta	5
E. Improta	14
E. Improta	1
E. Improta	20
E. Improta	15
E. Improta	37
E. Improta	6

E. Improta	6
E. Improta	3
E. Improta	11
E. Improta	26
E. Improta	56
E. Improta	3
E. Improta	1
E. Improta	9
E. Improta	2
E. Improta	6
E. Improta	1
E. Improta	6
E. Migliaccio	3
E. Migliaccio	1
E. Migliaccio	3
E. Migliaccio	1
E. Migliaccio	1
E. Migliaccio	5
E. Pacieri	3
F. Comandini	1
F. Comandini	6
F. Comandini	15
F. Comandini	13
F. Comandini	1
F. Comandini	14
F. Comandini	28
F. Comandini	3
F. Pelloni	9
F. Pelloni	2
F. Pelloni	27
F. Pelloni	8
F. Pelloni	1
F. Pelloni	6
F. Pelloni	2
F. Pelloni	1
F. Pelloni	1
F. Simmi	1
Filipp.	1
G. Carpaneto	1
G. Carpaneto	2
G. Carpaneto	1
G. Di Giulio	5
G. Franzini	2
G. Franzini	1
G. Montelli	1
G. Montelli	1
G. Nardi	1
G. Nardi	1
G. Papini & R. Magrone	1
Giannelli	1
M. Bologna	1
M. Cerruti	1
M. Cerruti	1
M. De Mei	8

M. De Mei	2
M. De Mei	1
M. De Mei	4
M. de Mei	3
M. Pitzalis & E. Trucchi	1
M. Pitzalis & E. Trucchi	2
M. Pitzalis & E. Trucchi	12
M. Pitzalis & E. Trucchi	25
M. Pitzalis & E. Trucchi	3
M. Pitzalis & E. Trucchi	1
O. Castellani	1
P. Luigioni	1
P. Maltzeff	1
P. Maltzeff	1
P. Maltzeff	1
P. Maltzeff	1
P. Maltzeff	1
P. Maltzeff	1
P. Maltzeff	2
P. Maltzeff	2
P. Maltzeff	2
P. Maltzeff	4
P. Maltzeff	1
P. Maltzeff	2
P. Maltzeff	2
P. Maltzeff	1
P. Maltzeff	1
P. Maltzeff	2
P. Maltzeff	1
P. Maltzeff	1
P. Maltzeff	3
Pedullà, Rellori & Vigna	1
Pedullà, Rellori & Vigna	3
Pedullà, Rellori & Vigna	1
Pedullà, Rellori & Vigna	1
Pedullà, Rellori & Vigna	27
Pedullà, Rellori & Vigna	3
Pedullà, Rellori & Vigna	3
Pedullà, Rellori & Vigna	1
Pedullà, Rellori & Vigna	34
Pedullà, Rellori & Vigna	1
Pedullà, Rellori & Vigna	1
Pedullà, Rellori & Vigna	3
Pedullà, Rellori & Vigna	1
Pedullà, Rellori & Vigna	4
Pedullà, Rellori & Vigna	4
Pedullà, Rellori & Vigna	2
Pedullà, Rellori & Vigna	2
Pedullà, Rellori & Vigna	1
Pedullà, Rellori & Vigna	2
Pedullà, Rellori & Vigna	2
Pedullà, Rellori & Vigna	2
Pedullà, Rellori & Vigna	9
Pedullà, Rellori & Vigna	4

Pedullà, Rellori & Vigna	3
R. Antonelli	1
S. de Felici	1
S. de Felici	1
S. de Felici	32
S. de Felici	14
S. de Felici	43
S. de Felici	2
S. de Felici	74
S. de Felici	5
S. de Felici	1
S. de Felici	6
S. de Felici	1
S. De Felici	15
S. De Felici	3
S. De Felici	1
S. Fattorini	1
S. Fattorini	1
S. Fattorini	1
S. Fattorini	1
Tripaldi & Vigna Taglianti	1
Tripaldi & Vigna Taglianti	9
Tripaldi & Vigna Taglianti	12
Tripaldi & Vigna Taglianti	19
Tripaldi & Vigna Taglianti	5
Tripaldi & Vigna Taglianti	1
Tripaldi & Vigna Taglianti	14
Tripaldi & Vigna Taglianti	1
Tripaldi & Vigna Taglianti	8
Tripaldi & Vigna Taglianti	61
Tripaldi & Vigna Taglianti	21
Tripaldi & Vigna Taglianti	6
Tripaldi & Vigna Taglianti	21
Tripaldi & Vigna Taglianti	3
Tripaldi & Vigna Taglianti	15
Tripaldi & Vigna Taglianti	1
Tripaldi & Vigna Taglianti	8
Tripaldi & Vigna Taglianti	7
Tripaldi & Vigna Taglianti	1
Tripaldi & Vigna Taglianti	38
Tripaldi & Vigna Taglianti	38
Vigna Taglianti & Lanci	17
Vigna Taglianti & Lanci	7
Vigna Taglianti & Lanci	1
Vigna Taglianti & Lanci	18
Vigna Taglianti & Lanci	1
Vigna Taglianti & Lanci	1
	1
	6
	1
	1
	1
	2
	1

	1
	1
	1
	1
G. Gobbi	1
M. Cerruti	2
	5
	1
O. Castellani	1
O. Castellani	1
O. Castellani	8
P. Luigioni	1
P. Luigioni	2
	1
E. Migliaccio	8
G. Pace	4
O. Castellani	1
P. Luigioni	1
E. Migliaccio	2
E. Migliaccio	2
F. Hartig	4
O. Castellani	1
P. Bonavita & A. Vigna Taglianti	1
P. Luigioni	1
P. Luigioni	1
P. Luigioni	2
P. Luigioni & A. Tirelli	2
R. Antonelli	1
R. Antonelli	2
S. Fattorini	5
S. Patrizi & M. Cerruti	1
	8
	16
	4
	4
	2
	1
	4
	1
A. Liberto	1
A. Parenti	2
De Maggi	2
De Maggi	2
E. Migliaccio	1
F. Gravina	1
F. Vegliante	1
G. Montelli	3
G. Rinaldi	1
J. Wolkhemer	4
M. Cerruti & Brivio	20
M. Cerruti & Brivio	4
M. de Mei	1
O. Castellani	1
O. Castellani	3

O. Castellani	3
O. Castellani	4
O. Castellani	1
P. Luigioni	3
P. Maltzeff	6
	25
	1
	2
	1
	1
	1
	1
M. Bologna	1
Straneo	3
Zuppa & Di Gaetano	1
	1
A. Bordoni	1
A. Ciceroni	1
Bonaventura	1
C. Consiglio	1
Cecchetti & A. Vigna	1
E. Colonnelli	1
E. Colonnelli	1
E. De Maggi	1
E. Garavaglia	3
E. Migliaccio	2
E. Migliaccio	3
E. Migliaccio	3
E. Migliaccio	4
E. Migliaccio	1
F. Comandini	2
F. Simmi	1
G. Di Giulio	1
M. Bologna	5
M. Cerruti	7
M. Zapparoli	2
M. Zapparoli & P. Jonales	1
R. Antonelli	1
R. Antonelli	1
R. Antonelli	2
R. Antonelli	3
S. Fattorini	4
S. Maugeri	2
S. Patrizi	1
S. Patrizi & M. Cerruti	1
S. Patrizi & M. Cerruti	6
S. Pedullà & R. Mellori	3
S. Pedullà & R. Mellori	8
S. Pedullà & R. Mellori	3
S. Pedullà & R. Mellori	2
Vigna Taglianti & Lanci	1
Vigna Taglianti & Lanci	2
Vigna Taglianti & Lanci	1
	1

	1
A. Biscaccianti	1
A. Liberto	1
E. Migliaccio	1
E. Migliaccio	1
M. Cerruti	2
P. Maltzeff	2
P. Maltzeff	1
P. Maltzeff	1
R. Antonelli	2
U. Pessolano	1
V. Viglioglia	1
V. Viglioglia	1
	2
	1
A. Liberto	2
E. Colonnelli	1
E. Migliaccio	1
G. Saccà	1
G. Saccà	1
L. Ciavaglia	1
S. Fattorini	1
A. Biscaccianti	5
A. Biscaccianti	4
E. Migliaccio	2
E. Migliaccio	1
E. Migliaccio	2
E. Piattella	12
G. Di Stefano	1
M. Cerruti	9
M. Mei	1
M. Zampetti	2
P. Luigioni	2
P. Luigioni	1
P. Luigioni	1
P. Luigioni	1
P. Luigioni	12
P. Maltzeff	2
P. Maltzeff	2
P. Maltzeff	2
P. Maltzeff	1
P. Maltzeff	1
P. Maltzeff	1
P. Maltzeff	1
P. Maltzeff	2
P. Maltzeff	2
P. Maltzeff	1
	23
	4
	1
	1
	1
	1
E. Colonnelli	3

F. Hartig	1
G. Pace	6
G. Pace	6
G. Pace	1
M. Cerruti	1
M. Cerruti	1
M. Cerruti	20
M. Cerruti & Brivio	2
P. Luigioni	6
Tripaldi & Vigna Taglianti	1
	2
M. Zampetti	28
P. Luigioni	15
	7
A. Biscaccianti	1
A. Biscaccianti	1
A. Bordoni	1
A. Di Giulio	2
A. Liuti & B. Labella	4
A. Nardi	2
A. Nardi	2
A. Nardi	3
A. Nardi	1
A. Nardi	2
A. Nardi	2
A. Pacifici	2
A. Tirelli	3
A. Vigna Taglianti	3
C. Consiglio	1
D. Cagiotti & L. Bertolaccini	7
D. Cagiotti & L. Bertolaccini	9
D. Cagiotti & L. Bertolaccini	4
D. Cagiotti & L. Bertolaccini	9
D. Cagiotti & L. Bertolaccini	5
D. Cagiotti & L. Bertolaccini	1
D. Cagiotti & L. Bertolaccini	7
D. Cagiotti & L. Bertolaccini	3
D. Cagiotti & L. Bertolaccini	3
D. Cagiotti & L. Bertolaccini	6
D. Cagiotti & L. Bertolaccini	11
D. Cagiotti & L. Bertolaccini	2
D. Cagiotti & L. Bertolaccini	2
D. Cagiotti & L. Bertolaccini	3
D. Cagiotti & L. Bertolaccini	1
D. Cagiotti & L. Bertolaccini	10
D. Cagiotti & L. Bertolaccini	10
D. Cagiotti & L. Bertolaccini	7
D. Cagiotti & L. Bertolaccini	10
D. Cagiotti & L. Bertolaccini	4
D. Cagiotti & L. Bertolaccini	5
D. Cagiotti & L. Bertolaccini	6
D. Cagiotti & L. Bertolaccini	2
E. Colonnelli	2
E. Migliaccio	3

E. Migliaccio	2
E. Pacieri	1
F. Neri	5
G. Binaghi	50
G. Carpaneto	1
G. Carpaneto	4
G. Carpaneto	1
G. Carpaneto	1
G. Carpaneto	1
G. Carpaneto	6
G. Carpaneto	2
G. Carpaneto	3
G. Franzini	3
G. Franzini	3
G. Nardi	1
G. Nardi	2
G. Nardi	1
G. Nardi	1
G. Nardi	2
G. Papini	1
M. Bologna	2
M. Cerruti	1
M. Cerruti	1
M. Cerruti	1
M. Mei	1
M. Mei	2
M. Mei	3
P. De Salvo	3
P. De Salvo	3
P. De Salvo	3
P. De Salvo	7
P. De Salvo	2
P. De Salvo	6
P. De Salvo	2
P. De Salvo	4
P. De Salvo	1
P. De Salvo	3
P. De Salvo	14
P. De Salvo	8
P. De Salvo	8
P. De Salvo	5
P. De Salvo	5
P. De Salvo	1
P. Luigioni	1
P. Luigioni	6
P. Maltzeff	3
P. Maltzeff	8
P. Maltzeff	2
P. Maltzeff	2
P. Maltzeff	1
P. Maltzeff	2
Prola	4
R. Antonelli	1
R. Montali	1

R. Montali	1
S. Cafaro	4
S. Fattorini	1
S. Fattorini	2
S. Fattorini & G. Di Stefano	2
S. Fattorini & G. Di Stefano	5
S. Fattorini & G. Di Stefano	1
S. Fattorini & G. Di Stefano	2
S. Rocchi	6
Tripaldi & Vigna Taglianti	1
Tripaldi & Vigna Taglianti	1
U. Pessolano	2
U. Pessolano	2
Wilson	1
	1
	3
	1
	1
	1
	1
	3
	26
	1
	1
	1
	3
	2
	1
	2
	1
	4
	2
G. Pace	1
C. Pesarini	1
Cotta	1
De Maggi	1
E. Colonnelli	1
E. Migliaccio	1
E. Migliaccio	1
G. Carpaneto	1
G. Carpaneto	1
L. Bertolaccini	1
M. Cerruti	1
M. Cerruti	1
M. Cerruti	6
M. Cerruti	2
M. Mei	1
O. Castellani	1
P. Luigioni	1
P. Luigioni	1
P. Luigioni	1
P. Luigioni	2
P. Maltzeff	1
Prola	1

R. Antonelli	2
R. Pittino	2
R. Tomaciello	1
S. Cafaro	2
S. De Angelis	3
S. de Felici	58
S. de Felici	318
S. de Felici	67
S. de Felici	40
S. de Felici	132
S. Fattorini	1
U. Pessolano	1
V. Viglioglia	1
	1
	1
	3
	1
	2
	1
	1
	1
	2
	1
	1
	2
	2
	1
	1
	1
	1
A. Mazziotta	1
M. Cerruti	2
M. Cerruti	2
S. Fattorini	1
S. Fattorini & F. Fedele	68
	1
	1
	1
C. Consiglio	1
C. Consiglio	1
C. Consiglio	1
C. Pesarini	1
E. Colonnelli	1
M. Cerruti	1
M. Cerruti	1
M. Cerruti	2
M. Cerruti	2
S. Fattorini & F. Fedele	2
	3
	6
	1
	1
	1
	1

	1
P. De Salvo	1
Torre Tasso	1
A. Vigna Taglianti	1
D. Cagiotti & L. Bertolaccini	65
D. Cagiotti & L. Bertolaccini	39
D. Cagiotti & L. Bertolaccini	38
D. Cagiotti & L. Bertolaccini	21
D. Cagiotti & L. Bertolaccini	21
D. Cagiotti & L. Bertolaccini	29
D. Cagiotti & L. Bertolaccini	41
D. Cagiotti & L. Bertolaccini	44
D. Cagiotti & L. Bertolaccini	54
D. Cagiotti & L. Bertolaccini	134
D. Cagiotti & L. Bertolaccini	116
D. Cagiotti & L. Bertolaccini	159
D. Cagiotti & L. Bertolaccini	63
D. Cagiotti & L. Bertolaccini	38
D. Cagiotti & L. Bertolaccini	36
D. Cagiotti & L. Bertolaccini	148
D. Cagiotti & L. Bertolaccini	170
D. Cagiotti & L. Bertolaccini	129
D. Cagiotti & L. Bertolaccini	132
D. Cagiotti & L. Bertolaccini	165
D. Cagiotti & L. Bertolaccini	54
D. Cagiotti & L. Bertolaccini	37
D. Cagiotti & L. Bertolaccini	192
D. Cagiotti & L. Bertolaccini	128
E. Migliaccio	67
E. Pacieri	2
F. Hartig	1
F.lli Garavaglia	1
G. Binaghi	24
G. Carpaneto	1
O. Castellani	1
P. De Salvo	7
P. De Salvo	6
P. De Salvo	8
P. De Salvo	2
P. De Salvo	2
P. De Salvo	2
P. De Salvo	3
P. De Salvo	6
P. De Salvo	5
P. De Salvo	4
P. De Salvo	5
P. De Salvo	4
P. De Salvo	13
P. De Salvo	6
P. De Salvo	3
P. De Salvo	15
P. De Salvo	1
P. De Salvo	7
P. De Salvo	5

P. De Salvo	2
P. De Salvo	2
P. De Salvo	5
P. De Salvo	8
P. De Salvo	6
S. Fattorini & G. Di Stefano	73
S. Fattorini & G. Di Stefano	3
S. Fattorini & G. Di Stefano	65
S. Fattorini & G. Di Stefano	1
S. Fattorini & G. Di Stefano	2
S. Patrizi	1
Settepassi	1
	1
	6
A. Biscaccianti	1
A. Biscaccianti	2
A. Biscaccianti	3
A. Ciceroni	1
A. Ciceroni	1
A. Ciceroni	1
A. Ciceroni	1
A. Ciceroni	1
A. Ciceroni	1
A. Ciceroni	1
A. Ciceroni	1
A. Ciceroni	2
A. Di Giulio	1
A. Liuti & B. Labella	4
A. Pacifici	4
A. Vigna Taglianti & De Liberato	1
Angeloni & Vigna	2
Angeloni & Vigna	1
C. Consiglio	1
C. Esposito	1
Calvario & Ruvolo	1
Cecchetti & A. Vigna	1
Cecchetti & A. Vigna	2
Cecchetti & A. Vigna	1
Cotta	3
D. Baiocchi	2
De Maggi	1
E. Colonnelli	2
E. Improta	1
E. Improta	1
E. Improta	2
E. Improta	1
E. Improta	2
E. Improta	1
E. Improta	1
E. Improta	1
E. Improta	1
E. Improta	1
E. Improta	1
E. Migliaccio	1

E. Migliaccio	1
E. Migliaccio	1
E. Migliaccio	5
E. Migliaccio	1
E. Migliaccio	2
E. Migliaccio	14
E. Migliaccio	1
E. Migliaccio	1
E. Migliaccio	4
F. Comandini	1
F. Comandini	1
F. Simmi	2
F. Simmi	2
G. Carpaneto	1
G. Di Giulio	1
G. Di Giulio	3
G. Di Stefano, C. Tronci & E. Romer	2
G. Garavini	2
G. Nardi	3
G. Nardi	10
G. Nardi	1
G. Nardi	1
G. Sama	1
L. Picco	3
L. Tizi	1
Leo	1
M. Bologna	2
M. Cerruti	2
M. Cerruti	1
M. Cerruti	2
M. Cerruti	1
M. Mei	1
M. Mei	3
O. Castellani	1
O. Castellani	2
O. Castellani	4
P. Angelini. A.Di Giulio & A. Vigna	1
P. Blasi leg.	1
P. Luigioni	1
P. Luigioni	1
P. Luigioni	8
P. Luigioni	1
P. Luigioni	1
P. Luigioni & A. Tirelli	1
P. Maltzeff	1
P. Maltzeff	4
P. Maltzeff	3
P. Maltzeff	1
P. Maltzeff	1
P. Maltzeff	1
P. Maltzeff	1
P. Maltzeff	1
P. Maltzeff	2
P. Maltzeff	1

P. Maltzeff	2
P. Maltzeff	2
P. Maltzeff	1
P. Maltzeff	1
P. Maltzeff	1
P. Maltzeff	2
P. Maltzeff	2
P. Maltzeff	1
P. Maltzeff	1
P. Maltzeff	2
P. Maltzeff	2
P. Maltzeff	1
P. Maltzeff	2
Pedullà, Rellori & Vigna	1
Pedullà, Rellori & Vigna	2
Pedullà, Rellori & Vigna	2
Pedullà, Rellori & Vigna	6
Pedullà, Rellori & Vigna	1
Pedullà, Rellori & Vigna	1
Pedullà, Rellori & Vigna	1
Pedullà, Rellori & Vigna	1
Pedullà, Rellori & Vigna	1
Pischedda	2
R. Antonelli	3
R. Antonelli	1
R. Antonelli	1
R. Antonelli	1
S. Bruno	1
S. de Felici	1
S. de Felici	1
S. Fattorini	1
S. Fattorini	1
S. Fattorini	1
S. Fattorini & G. Di Stefano	1
S. Maugeri	1
S. Maugeri	1
	1
	1
	1
	1
	1
	7
	1
	1
	2
	1
	1
	1
	1
	1
	4
A. Nardi	1
A. Tirelli	1
A. Tirelli	2

A. Tirelli	1
A. Tirelli	1
E. Colonnelli	4
F. de Maggi	1
F. Tassi	1
G. Carpaneto	1
G. Franzini	3
G. Nardi	2
G. Nardi	1
G. Nardi	2
P. Audisio	1
P. Luigioni	1
P. Luigioni	1
Prola	1
Prola	1
	1
	1
A. Tirelli	1
Altobelli	1
C. Esposito	1
De Maggi	6
E. Colonnelli	3
E. Migliaccio	2
E. Migliaccio	1
E. Migliaccio	1
E. Migliaccio	1
E. Migliaccio	9
E. Pacieri	5
E. Pacieri	1
F. Tassi	1
F. Tassi	1
F. Tassi	1
G. Franzini	1
G. Lepri	1
G. Montelli	1
G. Nardi	1
G. Nardi	1
G. Pinzari	1
G. sabatinelli	2
O. Castellani	1
O. Castellani	1
P. Luigioni	1
P. Luigioni	2
P. Luigioni	1
P. Maltzeff	1
P. Maltzeff	1
P. Maltzeff	8
P. Maltzeff	2
P. Maltzeff	2
P. Maltzeff	1
P. Maltzeff	1
P. Maltzeff	1
P. Maltzeff	1
P. Maltzeff	1

P. Maltzeff	11
P. Maltzeff	4
P. Maltzeff	1
P. Maltzeff	2
P. Maltzeff	2
P. Maltzeff	2
S. Bruno	1
S. Bruschi	2
S. Patrizi	1
S. Patrizi & M. Cerruti	4
S. Patrizi & M. Cerruti	1
Vita	1
W. Rossi	1
	4
	3
	1
	1
	2
	1
O. Castellani	1
S. Fattorini & G. Di Stefano	34
S. Fattorini & G. Di Stefano	39
S. Fattorini & G. Di Stefano	3
Stagnitta	1
P. Luigioni	1
P. Vaglio	1
P. Vaglio	1
	1
	1
	1
	1
Ciampi	1
E. Colonnelli	1
G. Binaghi	2
	1
	5
A. Pacifici	2
A. Pacifici	1
A. Pacifici	5
De Maggi	1
E. Pacieri	3
G. Carpaneto	2
G. Carpaneto	2
G. Lepri	1
G. Pace	1
G. Pace	1
P. Bombi & S. Fattorini	1
P. Luigioni	2
P. Maltzeff	3
P. Maltzeff	5
P. Maltzeff	2
P. Maltzeff	1
R. Antonelli	2
S. Fattorini	2

	3
	2
	4
	1
D. Baiocchi	10
D. Baiocchi	10
	1
A. Biscaccianti	2
A. Biscaccianti	2
A. Biscaccianti	1
A. Biscaccianti & Facchinelli	1
A. Ciceroni	4
A. Di Giulio	1
A. Liberto	2
C. Tronci	1
Calvario & Ruvolo	1
Cotta	3
Di Rao	9
E. De Maggi	2
E. Migliaccio	1
E. Migliaccio	3
E. Migliaccio	1
E. Migliaccio	1
E. Migliaccio	1
E. Migliaccio	1
E. Migliaccio	1
E. Migliaccio	11
E. Migliaccio	1
F. Hartig	2
G. Di Giulio	1
G. Di Giulio	1
G. Di Giulio	1
G. Di Giulio	3
G. Di Giulio	8
G. Di Giulio	1
G. Nardi	4
G. Nardi	3
L. Barbera	8
L. Corsetti	1
M. de Mei	1
M. Iglesia	1
M. Mei	2
M. Mei	2
M. Pitzalis & E. Trucchi	1
M. Zampetti	1
P. Luigioni	2
P. Luigioni & A. Tirelli	1
P. Maltzeff	1
P. Maltzeff	2
P. Maltzeff	1
P. Maltzeff	1
P. Maltzeff	1
P. Maltzeff	2
P. Maltzeff	3

P. Maltzeff	2
P. Maltzeff	4
P. Maltzeff	1
P. Maltzeff	1
P. Maltzeff	3
P. Maltzeff	2
P. Maltzeff	1
P. Maltzeff	2
P. Maltzeff	1
P. Maltzeff	1
P. Maltzeff	1
P. Maltzeff	8
P. Maltzeff	1
R. Antonelli	1
R. Antonelli	2
S. Fattorini	1
S. Fattorini	2
S. Fattorini	1
S. Fattorini	1
S. Fattorini & G. Di Stefano	1
S. Fattorini & G. Di Stefano	1
S. Fattorini & G. Di Stefano	1
	1
	1
	1
A. Biscaccianti	1
A. Ciceroni	1
A. Ciceroni	1
A. Pacifici	4
C. Consiglio	3
E. Improta	1
E. Migliaccio	1
E. Migliaccio	1
E. Migliaccio	1
E. Migliaccio	6
E. Migliaccio	2
E. Migliaccio	1
E. Migliaccio	1
F. Hartig	2
G. Franzini	9
G. Nardi	1
G. Nardi	1
G. Nardi	1
G. Nardi	1
G. Nardi	1
G. Nardi	2
G. Nardi	1
G. Nardi	4
G. Nardi	8
G. Pace	6
G. Pace	3
M. Cerruti	1
M. Cerruti	1
M. Cerruti	1

M. Cerruti	1
M. Mosca	1
M. Pitzalis & E. Trucchi	1
M. Zampetti	2
P. Luigioni	2
P. Luigioni	1
P. Luigioni	1
P. Maltzeff	1
P. Maltzeff	1
P. Maltzeff	1
R. Antonelli	1
R. Antonelli	1
S. Fattorini	1
	1
	1
	1
	4
	1
	4
	3
	2
	1
	1
	1
	1
	2
	3
	2
	1
	1
	1
	1
	1
	1
A. Biscaccianti	1
E. Migliaccio	1
E. Migliaccio	1
G. Pace	1
P. Maltzeff	1
P. Maltzeff	2
P. Maltzeff	2
P. Maltzeff	2
P. Maltzeff	1
P. Maltzeff	1
	1
	1
C. Esposito	2
C. Esposito	3
G. Pace	2
P. Luigioni	6
	4
	15
	4
	7

Barb. ?	2
G. Pace	1
G. Pace	1
M. Cerruti	3
M. Cerruti	2
M. Mei	4
P. Maltzeff	2
P. Maltzeff	3
P. Maltzeff	1
P. Maltzeff	1
P. Maltzeff	4
P. Maltzeff	5
S. Patrizi	1
	2
	2
	1
B. Osella	3
A. Pacifici	9
A. Parenti	3
B. Labella	1
C. Esposito	1
Campo	2
E. Colonnelli	1
E. Colonnelli	1
E. Colonnelli & A. Liberto	1
E. Gridelli	2
E. Improta	62
E. Improta	4
E. Improta	4
E. Improta	4
E. Migliaccio	1
E. Migliaccio	1
E. Migliaccio	1
E. Migliaccio	3
G. Carpaneto	1
G. Carpaneto	1
G. Carpaneto	1
G. Nardi	1
G. Nardi	2
G. Nardi	1
G. Nardi	2
G. Nardi	1
G. Nardi	1
G. Nardi	1
G. Nardi	2
G. Nardi	3
G. Nardi	2
G. Nardi	1
G. Nardi	1
G. Nardi	1
G. Nardi	1
G. Nardi	3
G. Nardi	1
G. Nardi	1

G. Pace	1
G. Papini	1
G. Papini	1
G. Papini	1
G. Papini	2
M. Bologna	1
M. Cerruti	2
M. Cerruti	2
M. Cerruti	4
M. Cerruti	4
M. Inglisa	1
M. Mei	2
M. Paris	1
M. Zampetti	2
P. Luigioni	2
P. Maltzeff	1
P. Maltzeff	2
P. Maltzeff	1
P. Maltzeff	2
P. Maltzeff	1
P. Maltzeff	1
P. Maltzeff	1
P. Maltzeff	1
P. Maltzeff	1
P. Maltzeff	1
R. Antonelli	1
R. Pergolesi	1
S. de Felici	1
S. de Felici	118
S. de Felici	3
S. Fattorini	1
Settepassi	1
U. Pessolano	1
	2
	3
	2
	1
	1
	1
	1
	81
	6
	1
	2
	1
	1
	1
	1
	1
	1
	1
	1
	1
	1

	2
	2
	3
A. Biscaccianti	1
A. Biscaccianti	1
A. Biscaccianti	1
A. Penniri	1
A. Biscaccianti	1
E. Colonnelli	1
E. Colonnelli	4
G. Pace	2
M. Cerruti	4
M. Zampetti	2
M. Zampetti	1
O. Castellani	10
P. Luigioni	9
	1
	3
	1
	15
A. Biscaccianti	1
A. Biscaccianti	1
A. Bordoni	1
A. Ciceroni	1
A. Ciceroni	1
A. Di Giulio	1
A. Vigna Taglianti	1
Calvario & Ruvolo	1
Calvario & Ruvolo	1
Calvario & Ruvolo	5
Calvario & Ruvolo	1
Calvario & Ruvolo	1
Cotta	1
Cotta	1
Damazia	1
Di Gaetano	3
E. Colonnelli	2
E. Improta	13
E. Improta	5
E. Improta	11
E. Improta	2
E. Improta	18
E. Improta	3
E. Improta	10
E. Improta	31
E. Improta	3
E. Migliaccio	1
E. Migliaccio	1
E. Migliaccio	2
G. Binaghi	7
G. Carpaneto	1
G. Di Stefano	2
G. Di Stefano	2
G. Di Stefano	1

G. Di Stefano	1
G. Di Stefano	1
G. Montelli	1
G. Nardi	2
Giannelli	1
Leo	2
M. Bologna	1
M. Cerruti	1
M. Cerruti	1
M. Cerruti	4
M. Cerruti	1
M. Cerruti	2
M. De Mei	1
M. De Mei	1
M. Fraschetti	1
M. Mei	1
M. Mei	1
M. Mei	1
M. Pitzalis & E. Trucchi	1
M. Pitzalis & E. Trucchi	2
M. Zampetti	2
M. Zampetti	2
M. Zapparoli & Giannelli	1
M. Zapparoli & P. Jonales	1
Narducci	1
Narducci	1
P. Maltzeff	1
P. Maltzeff	1
P. Maltzeff	1
P. Maltzeff	1
P. Maltzeff	1
P. Maltzeff	1
P. Maltzeff	1
P. Maltzeff	1
P. Maltzeff	1
P. Maltzeff	1
P. Maltzeff	1
P. Maltzeff	1
P. Maltzeff	1
P. Maltzeff	1
P. Maltzeff	1
P. Maltzeff	1
P. Maltzeff	1
P. Maltzeff	1
P. Maltzeff	1
R. Antonelli	1
R. Antonelli	1
R. Antonelli	1
S. Cafaro	1
S. Cafaro	1
S. De Felici	8
S. De Felici	3
S. De Felici	10
S. De Felici	7

S. De Felici	29
S. De Felici	9
S. De Felici	2
S. De Felici	1
S. Fattorini	1
S. Fattorini	1
S. Fattorini	1
S. Fattorini	1
S. Fattorini & F. Fedele	3
Tripaldi & Vigna Taglianti	2
Tripaldi & Vigna Taglianti	1
Tripaldi & Vigna Taglianti	1
Tripaldi & Vigna Taglianti	2
U. Pessolano	1
	1
	1
	3
	1
	3
	1
	2
	1
	1
	1
	1
	1
	1
	1
	12
	3
	12
	1
	2
	1
	1
	1
	3
	42
	1
	1
	1
	3
	3
	1
	1
	1
	1
	1
	23
	1
	11
	1
	1
	1

G. Pace	13
G. Pace	2
P. Luigioni	1
	2
	15
M. Zampetti	1
P. Luigioni	10
P. Luigioni	6
R. Pace	1
A. Ceccarelli	2
A. Parenti	29
A. Vigna & E. DeMatthaeis	9
D. Cagiotti & L. Bertolaccini	179
D. Cagiotti & L. Bertolaccini	32
D. Cagiotti & L. Bertolaccini	6
D. Cagiotti & L. Bertolaccini	3
D. Cagiotti & L. Bertolaccini	96
D. Cagiotti & L. Bertolaccini	393
D. Cagiotti & L. Bertolaccini	208
D. Cagiotti & L. Bertolaccini	131
D. Cagiotti & L. Bertolaccini	38
D. Cagiotti & L. Bertolaccini	18
D. Cagiotti & L. Bertolaccini	13
D. Cagiotti & L. Bertolaccini	52
D. Cagiotti & L. Bertolaccini	59
D. Cagiotti & L. Bertolaccini	151
D. Cagiotti & L. Bertolaccini	94
D. Cagiotti & L. Bertolaccini	126
D. Cagiotti & L. Bertolaccini	178
D. Cagiotti & L. Bertolaccini	148
D. Cagiotti & L. Bertolaccini	72
D. Cagiotti & L. Bertolaccini	5
E. Colonnelli	3
G. Carissimi	1
G. Carpaneto	10
G. Carpaneto	3
G. Di Giulio	1
G. Nardi & C. Esposito	1
G. Pace	1
G. Pace	1
G. Pace	1
G. Pace	40
G. Pace	12
M. Mei	1
M. Mei	1
M. Zampetti	1
M. Zampetti	2
P. De Salvo	2
P. De Salvo	1
P. De Salvo	1
P. De Salvo	1
P. De Salvo	1
Prola	1
R. Montali	4

S. Fattorini	1
S. Fattorini & G. Di Stefano	1
V. De Sanctis	1
	1
	1
R. Montali	1
R. Montali	1
R. Montali	2
	4
A. Palmirani	2
Berti	11
Ciacchi	13
Ciacchi	1
D. Cagiotti & L. Bertolaccini	7
D. Cagiotti & L. Bertolaccini	12
D. Cagiotti & L. Bertolaccini	1
D. Cagiotti & L. Bertolaccini	1
D. Cagiotti & L. Bertolaccini	18
D. Cagiotti & L. Bertolaccini	20
D. Cagiotti & L. Bertolaccini	14
D. Cagiotti & L. Bertolaccini	17
D. Cagiotti & L. Bertolaccini	12
D. Cagiotti & L. Bertolaccini	5
D. Cagiotti & L. Bertolaccini	2
D. Cagiotti & L. Bertolaccini	11
D. Cagiotti & L. Bertolaccini	34
D. Cagiotti & L. Bertolaccini	7
D. Cagiotti & L. Bertolaccini	10
D. Cagiotti & L. Bertolaccini	8
D. Cagiotti & L. Bertolaccini	15
D. Cagiotti & L. Bertolaccini	15
D. Cagiotti & L. Bertolaccini	10
D. Cagiotti & L. Bertolaccini	5
E. Colonnelli	1
E. Migliaccio	2
E. Migliaccio	5
E. Migliaccio	2
E. Migliaccio	1
E. Migliaccio	1
E. Migliaccio	9
G. Carpaneto	1
G. Carpaneto	17
G. Carpaneto	18
G. Carpaneto	5
G. M. Ghidini & M. Pavan	1
G. M. Ghidini & M. Pavan	1
G. Pace	1
G. Pace	5
Garavaglia	1
M. Zampetti	1
M. Zampetti	3
M. Zampetti	3
P. De Salvo	1
P. De Salvo	2

P. Luigioni	3
Prola	3
R. Antonelli	3
R. Antonelli	3
S. Fattorini	2
	1
	1
	1
	7
	2
	2
v. Poli	1
C. Consiglio	1
M. Cerruti	1
P. Luigioni	1
S. Fattorini	1
Sesino	5
Straneo	4
	3
	4
	1
A. Biscaccianti	1
A. Biscaccianti	1
A. Biscaccianti	1
A. Biscaccianti	1
A. Bordoni	4
A. Di Giulio & M. Bologna	2
A. Nardi	1
A. Nardi	1
A. Nardi	4
A. Nardi	4
A. Nardi	10
A. Nardi	3
A. Nardi	3
A. Pacifici	6
B. Maltzeff	1
C. Consiglio	6
C. Consiglio	3
C. Consiglio	1
Calvario & Ruvolo	2
Calvario & Ruvolo	1
Calvario & Ruvolo	13
Cirocchi	2
D. Cagiotti & L. Bertolaccini	3
D. Cagiotti & L. Bertolaccini	3
D. Cagiotti & L. Bertolaccini	7
D. Cagiotti & L. Bertolaccini	3
D. Cagiotti & L. Bertolaccini	1
D. Cagiotti & L. Bertolaccini	1
D. Cagiotti & L. Bertolaccini	7
D. Cagiotti & L. Bertolaccini	5
D. Cagiotti & L. Bertolaccini	3
D. Cagiotti & L. Bertolaccini	1
D. Cagiotti & L. Bertolaccini	7

D. Cagiotti & L. Bertolaccini	8
D. Cagiotti & L. Bertolaccini	4
D. Cagiotti & L. Bertolaccini	3
D. Cagiotti & L. Bertolaccini	13
D. Cagiotti & L. Bertolaccini	6
D. Cagiotti & L. Bertolaccini	2
D. Cagiotti & L. Bertolaccini	1
D. Cagiotti & L. Bertolaccini	3
D. Cagiotti & L. Bertolaccini	5
E. Gridelli	2
E. Migliaccio	1
E. Migliaccio	1
E. Migliaccio	16
E. Migliaccio	11
E. Migliaccio	1
E. Migliaccio	1
E. Pacieri	1
F. Comandini	1
F. Neri	2
Falcio	2
G. Binaghi	60
G. Carpaneto	3
G. Carpaneto	2
G. Carpaneto	2
G. Carpaneto	4
G. Carpaneto	2
G. Carpaneto	1
G. Carpaneto	1
G. Carpaneto	1
G. Carpaneto	1
G. Di Giulio	8
G. Nardi	1
G. Nardi	3
G. Nardi	5
G. Nardi	4
G. Nardi	1
G. Nardi	1
G. Nardi	1
G. Papini	1
G. Pavia	1
G. Pavia	3
L. Barbera	1
M. Bologna	1
M. Bologna	2
M. Cerruti	1
M. Cerruti	1
M. Cerruti	1
M. Cerruti	4
M. De Mei	135
M. De Mei	84
M. De Mei	114
M. Mei	1
M. Mei	2
M. Mei	1

M. Pitzalis & E. Trucchi	1
M. Pitzalis & E. Trucchi	7
M. Pitzalis & E. Trucchi	5
M. Pitzalis & E. Trucchi	6
M. Zampetti	1
O. Castellani	1
O. Castellani	2
P. De Salvo	17
P. De Salvo	14
P. De Salvo	15
P. De Salvo	21
P. De Salvo	7
P. De Salvo	7
P. De Salvo	36
P. De Salvo	1
P. De Salvo	3
P. De Salvo	23
P. De Salvo	7
P. De Salvo	29
P. De Salvo	24
P. De Salvo	20
P. De Salvo	14
P. De Salvo	2
P. Luigioni	2
P. Luigioni	3
P. Luigioni	1
P. Luigioni	3
P. Maltzeff	4
P. Maltzeff	1
P. Maltzeff	2
P. Maltzeff	3
P. Maltzeff	1
P. Maltzeff	2
P. Maltzeff	2
P. Maltzeff	2
P. Maltzeff	2
P. Tollis	1
P. Vaglio	1
Prola	2
R. Antonelli	1
R. Antonelli	2
R. Antonelli	1
S. Cafaro	2
S. Fattorini	1
S. Fattorini	2
S. Fattorini & G. Di Stefano	10
S. Fattorini & G. Di Stefano	10
S. Fattorini & G. Di Stefano	4
S. Fattorini & G. Di Stefano	5
S. Fattorini & G. Di Stefano	1
Tripaldi & Vigna Taglianti	11
Tripaldi & Vigna Taglianti	1
Tripaldi & Vigna Taglianti	3
Tripaldi & Vigna Taglianti	3

Tripaldi & Vigna Taglianti		2
Tripaldi & Vigna Taglianti		3
U. Pessolano		2
U. Pessolano		2
U. Pessolano		5
U. Pessolano		4
U. Pessolano		2
U. Pessolano		1
V. Aliquò	numerous	
		3
		4
		2
		10
		11
		3
		2
		4
		43
		4
		7
		2
		2
		22
		1
		3
		1
		1
		1
		1
		3
		1
P. Luigioni		1
		1
E. Colonnelli		3
E. Colonnelli		1
E. Migliaccio		1
E. Migliaccio		2
G. Carpaneto		1
G. Lepri		2
G. Pace		3
M. Bologna		1
M. Bologna		1
M. Zampetti		5
P. Luigioni		2
P. Maltzeff		4
P. Maltzeff		14
P. Maltzeff		1
P. Maltzeff		2
R. Antonelli		1
S. Fattorini & G. Di Stefano		4
S. Patrizi		1
		1
		2

A. Pacifici	about 350	
A. Pacifici		350
A. Pacifici		52
A. Tirelli		2
A. Tirelli		1
A. Vigna & E. DeMatthaeis		10
A. Vigna Taglianti		1
D. Cagiotti & L. Bertolaccini		13
D. Cagiotti & L. Bertolaccini		44
D. Cagiotti & L. Bertolaccini		36
D. Cagiotti & L. Bertolaccini		93
D. Cagiotti & L. Bertolaccini		86
D. Cagiotti & L. Bertolaccini		49
D. Cagiotti & L. Bertolaccini		2
D. Cagiotti & L. Bertolaccini		140
D. Cagiotti & L. Bertolaccini		52
D. Cagiotti & L. Bertolaccini		120
D. Cagiotti & L. Bertolaccini		102
D. Cagiotti & L. Bertolaccini		16
D. Cagiotti & L. Bertolaccini		1
D. Cagiotti & L. Bertolaccini		1
D. Cagiotti & L. Bertolaccini		1
G. Binaghi		100
G. Carpaneto		17
G. Carpaneto		17
G. Pace		2
G. Pace		7
G. Pace		10
Messori		1
P. Binaghi		54
P. De Salvo		4
P. De Salvo		1
P. De Salvo		5
P. De Salvo		4
P. De Salvo		1
P. De Salvo		2
P. De Salvo		3
P. De Salvo		11
P. De Salvo		12
P. De Salvo		14
S. Fattorini		1
A. Biscaccianti		1
A. Biscaccianti		1
A. Mazziotta		2
E. Migliaccio		1
E. Migliaccio		1
E. Migliaccio		1
E. Migliaccio		1
F. Pelloni		1
F. Pelloni		4
F. Pelloni		2
F. Pelloni		1
Giaquinto		3
P. Luigioni		10

P. Luigioni	1
Pedullà, Rellori & Vigna	1
Pedullà, Rellori & Vigna	1
S. Fattorini	1
S. Fattorini & A. Aradis	1
Vigna Taglianti & Lanci	1
A. Biscaccianti	1
A. Biscaccianti	3
A. Biscaccianti	1
A. Di Giulio & M. Bologna	3
Bonaventura	1
De Wagner	1
E. Migliaccio	2
E. Migliaccio	2
E. Migliaccio	2
F. Neri	5
G. Carpaneto	1
G. Carpaneto	3
G. Di Stefano	6
L. Barbera	1
M. Bologna	2
M. De Mei	2
M. De Mei	29
M. De Mei	31
M. Mei	1
M. Mei	1
M. Mei	1
O. Castellani	1
O. Castellani	1
P. Luigioni	1
P. Luigioni	1
P. Luigioni	1
P. Luigioni	1
P. Luigioni	1
P. Luigioni	1
P. Luigioni	1
P. Luigioni	1
P. Maltzeff	2
P. Maltzeff	2
P. Maltzeff	2
P. Maltzeff	3
P. Maltzeff	1
P. Maltzeff	4
P. Maltzeff	4
P. Maltzeff	1
P. Maltzeff	1
P. Maltzeff	1
P. Maltzeff	2
P. Maltzeff	1
P. Maltzeff	1
P. Maltzeff	1
P. Maltzeff	1
P. Maltzeff	1
P. Maltzeff	1

P. Maltzeff	2
P. Maltzeff	2
Prola	3
R. Antonelli	1
R. Antonelli	1
R. Montali	1
S. Cafaro	1
S. Fattorini	1
S. Fattorini	2
S. Fattorini	1
S. Fattorini & F. Fedele	6
S. Fattorini & G. Di Stefano	1
S. Fattorini, S. & G. Di Stefano	7
S. Nardi	1
S. Patrizi	1
V. Viglioglia	1
	1
	1
	1
	1
	1
	1
	3
	1
	5
	1
	1
	1
	1
	1
	1
	1
	1
	1
	2
	1
	5
	1
	1
	1
	1
	1
	1
	1
	1
	1
	1
	7
	1
	2
	1
	1
	1
	1

	1
A. Ciceroni	2
A. Ciceroni	1
A. Ciceroni	1
A. Ciceroni	1
A. Ciceroni	1
A. Ciceroni	3
A. Ciceroni	1
A. Ciceroni	2
A. Ciceroni	2
A. Ciceroni	1
A. Ciceroni	1
A. Ciceroni	2
A. Liberto	1
A. Liberto	2
A. Pacifici	9
A. Vigna Taglianti & De Liberato	1
Cecchetti & A. Vigna	1
E. Colonnelli	3
E. Improta	1
E. Improta	1
E. Improta	1
E. Migliaccio	1
E. Migliaccio	1
E. Migliaccio	1
G. Pace	1
G. Sama	2
I. Mosca	1
M. Bologna	4
March	1
P. Luigioni	4
P. Luigioni	1
P. Luigioni	1
P. Luigioni	1
Pedullà, Rellori & Vigna	1
R. Antonelli	2
Rossi	1
S. de Felici	1
S. Fattorini & G. Di Stefano	1
S. Fattorini & G. Di Stefano	3
	1
	1
	2
	1
	1
C. Consiglio	1
E. Colonnelli	1
F. Cassola	2
F. Hartig	11
G. Di Giulio	1
G. Di Giulio	1
G. Franzini	2
G. Nardi	2
G. Nardi	2

G. Nardi	1
G. Nardi	2
G. Nardi	3
G. Nardi	1
G. Nardi	2
G. Nardi	1
G. Nardi	2
M. Cerruti	2
M. Cerruti	1
M. Cerruti	3
M. Cerruti	8
M. Cerruti	1
M. Cerruti	6
M. Cerruti	1
M. Cerruti	1
M. Zapparoli	1
P. Luigioni	1
P. Maltzeff	1
P. Maltzeff	1
S. Fattorini & B. Renna	3
	2
	5
	185
	6
	1
	1
	2
Calvario & Ruvolo	1
Calvario & Ruvolo	1
Calvario & Ruvolo	2
Calvario & Ruvolo	8
Calvario & Ruvolo	2
Calvario & Ruvolo	6
Calvario & Ruvolo	1
F. Hartig	1
G. Binaghi	100
G. Carpaneto	2
G. Carpaneto	2
G. Carpaneto	3
L. Bertolaccini	1
M. Cerruti	1
M. Pitzalis & E. Trucchi	1
M. Pitzalis & E. Trucchi	7
M. Pitzalis & E. Trucchi	2
M. Pitzalis & E. Trucchi	10
P. De Salvo & M. Bologna	1
P. De Salvo & M. Bologna	1
P. De Salvo & M. Bologna	1
P. De Salvo & M. Bologna	1
S. Fattorini & G. Di Stefano	1
Tripaldi & Vigna Taglianti	1
Tripaldi & Vigna Taglianti	1
Tripaldi & Vigna Taglianti	4
Tripaldi & Vigna Taglianti	3

Tripaldi & Vigna Taglianti	1
Tripaldi & Vigna Taglianti	1
Tripaldi & Vigna Taglianti	1
Tripaldi & Vigna Taglianti	1
	2
	1
A. Biscaccianti	1
A. Biscaccianti	3
A. Ceccarelli	2
A. Ciceroni	2
A. Pacifici	2
A. Pacifici	15
C. Consiglio	1
C. Consiglio	1
E. Colonnelli	2
E. Colonnelli	1
E. Migliaccio	1
F. Pelloni	1
F. Pelloni	1
F. Pelloni	1
F. Pelloni	2
F. Pelloni	1
G. Di Giulio	1
G. Di Giulio	9
G. Di Giulio	12
G. Di Giulio	8
G. Di Stefano	1
G. Franzini	1
G. Nardi	2
G. Nardi	1
G. Nardi	3
G. Nardi	1
G. Nardi	1
G. Nardi	1
G. Nardi	4
G. Nardi	27
G. Nardi	6
L. Picco	1
L. Picco	3
L. Picco	4
M. Cerruti	3
M. Cerruti	1
M. Cerruti	1
M. Cerruti	1
M. Cerruti	1
M. Mei	1
M. Mei	1
M. Pitzalis & E. Trucchi	11
M. Pitzalis & E. Trucchi	24
M. Pitzalis & E. Trucchi	26
M. Pitzalis & E. Trucchi	22
M. Pitzalis & E. Trucchi	5
M. Pitzalis & E. Trucchi	2
M. Zampetti	1

M. Zapparoli	1
O. Castellani	1
O. Castellani	4
P. Luigioni	8
P. Luigioni	8
P. Maltzeff	4
P. Maltzeff	3
P. Maltzeff	10
P. Maltzeff	1
P. Maltzeff	1
P. Maltzeff	1
P. Maltzeff	5
P. Maltzeff	90
P. Maltzeff	4
P. Maltzeff	1
P. Maltzeff	4
R. Antonelli	1
R. Antonelli	2
R. Consorti	2
R. Consorti	5
R. Pergolesi	1
S. Fattorini	8
S. Fattorini	1
S. Fattorini	1
S. Fattorini	1
S. Fattorini & F. Fedele	4
S. Fattorini & G. Di Stefano	3
S. Fattorini, S. & G. Di Stefano	1
S. Riese	1
V. Viglioglia	1
	1
	1
	1
	1
	3
	1
	2
	1
	2
	3
	44
	1
	2
	2
	5
A. Biscaccianti	1
A. Biscaccianti	1
A. Biscaccianti	1
A. Biscaccianti	1
A. Biscaccianti	1
A. Biscaccianti	1
A. Biscaccianti	1
A. Bordoni	1
A. Liuti & B. Labella	1

A. Nardi	1
A. Nardi	2
A. Pacifici	1
A. Pacifici	2
A. Tirelli	1
A. Vigna Taglianti	1
A. Vigna Taglianti	1
Berti	2
Brunelli	1
C. Consiglio	3
C. Consiglio	1
Calvario & Ruvolo	1
Calvario & Ruvolo	1
Calvario & Ruvolo	1
E. Colonnelli	2
E. Colonnelli	2
E. Colonnelli	2
E. Migliaccio	1
E. Migliaccio	3
E. Migliaccio	3
E. Migliaccio	1
E. Migliaccio	1
E. Migliaccio	7
E. Pacieri	1
EdM	1
G. Binaghi	30
G. Carpaneto	1
G. Carpaneto	2
G. Carpaneto	2
G. Carpaneto	4
G. Carpaneto	1
G. Di Giulio	1
G. Franzini	1
G. Nardi	1
G. Nardi	1
G. Nardi	2
G. Nardi	8
G. Nardi	1
G. Nardi	6
G. Nardi	1
G. Nardi	1
G. Nardi	3
G. Pace	2
L. Barbera	1
M. Bologna	1
M. Bologna	2
M. Bologna	2
M. Cerruti	1
M. Cerruti	1
M. Mei	1
M. Mei	2
M. Mei	2
M. Pitzalis & E. Trucchi	5
M. Pitzalis & E. Trucchi	7

M. Pitzalis & E. Trucchi	137
M. Pitzalis & E. Trucchi	60
M. Pitzalis & E. Trucchi	40
M. Pitzalis & E. Trucchi	1
M. Pitzalis & E. Trucchi	1
M. Pitzalis & E. Trucchi	22
M. Pitzalis & E. Trucchi	20
M. Zampetti	1
M. Zampetti	1
Mazzi	1
O. Castellani	1
P. Luigioni	2
P. Luigioni	1
P. Luigioni	1
P. Luigioni	3
P. Luigioni	1
P. Luigioni	14
P. Maltzeff	4
P. Maltzeff	1
P. Maltzeff	2
P. Maltzeff	2
P. Maltzeff	6
P. Maltzeff	2
P. Maltzeff	1
P. Maltzeff	5
P. Maltzeff	2
P. Maltzeff	2
P. Maltzeff	1
P. Maltzeff	1
P. Maltzeff	1
P. Maltzeff	2
P. Maltzeff	1
P. Maltzeff	4
Prola	3
R. Antonelli	2
R. Antonelli	1
R. Antonelli	1
R. Antonelli	3
R. Antonelli	1
S. Cafaro	3
S. Cafaro	3
S. Fattorini	3
S. Fattorini	1
S. Fattorini & G. Di Stefano	1
S. Fattorini & G. Di Stefano	2
S. Fattorini & G. Di Stefano	1
S. Fattorini & G. Di Stefano	7
S. Fattorini & G. Di Stefano	3
S. Fattorini & G. Di Stefano	1
S. Gazzo	1
S. Rocchi	5
Settepassi	1
Tripaldi & Vigna Taglianti	3
Tripaldi & Vigna Taglianti	8

Tripaldi & Vigna Taglianti	1
Tripaldi & Vigna Taglianti	1
Tripaldi & Vigna Taglianti	1
U. Pessolano	1
U. Pessolano	2
U. Pessolano	1
	1
	3
	1
	2
	1
	2
	3
	7
	3
	12
	4
	20
	5
	1
	8
	10
	1
	12
	2
	9
	2
	1
	1
	1
	8
	1
	1
	1
A. Di Giulio & M. Bologna	1
A. Liuti & B. Labella	1
Cotta	1
D. Luchetti	1
E. Colonnelli	1
E. Gridelli	4
E. Migliaccio	2
E. Migliaccio	1
G. Di Giulio	1
G. Doria	1
G. Nardi	1
G. Nardi	1
G. Nardi	1
G. Nardi	1
G. Nardi	1
G. Nardi	1
G. Nardi	1
G. Nardi	1
G. Nardi	1
G. Nardi	1

G. Nardi	1
G. Nardi	1
G. Nardi	1
M. Cerruti	1
M. De Mei	48
M. De Mei	44
M. De Mei	39
M. Inglisa	1
M. Inglisa	2
M. Mei	3
M. Zampetti	1
P. Luigioni	1
P. Luigioni	1
P. Luigioni	1
P. Luigioni	1
P. Luigioni	2
P. Luigioni	1
P. Luigioni	1
P. Luigioni	1
P. Luigioni	1
P. Luigioni	1
P. Luigioni	4
P. Maltzeff	1
P. Maltzeff	14
P. Maltzeff	1
P. Maltzeff	1
Prola	1
R. Antonelli	1
R. Antonelli	1
	1
	1
	1
	1
	2
	2
	1
	4
	2
	2
	14
	1
	1
	2
	2
A. Parenti	1
D. Cagiotti & L. Bertolaccini	21
D. Cagiotti & L. Bertolaccini	32
D. Cagiotti & L. Bertolaccini	6
D. Cagiotti & L. Bertolaccini	11
D. Cagiotti & L. Bertolaccini	5
D. Cagiotti & L. Bertolaccini	23
D. Cagiotti & L. Bertolaccini	32
D. Cagiotti & L. Bertolaccini	20
D. Cagiotti & L. Bertolaccini	8

D. Cagiotti & L. Bertolaccini	1
D. Cagiotti & L. Bertolaccini	5
D. Cagiotti & L. Bertolaccini	3
D. Cagiotti & L. Bertolaccini	2
D. Cagiotti & L. Bertolaccini	2
D. Cagiotti & L. Bertolaccini	4
D. Cagiotti & L. Bertolaccini	4
E. Colonnelli	1
G. Binaghi	100
G. Carpaneto	1
G. Carpaneto	2
O. Castellani	1
R. Montali	5
S. Fattorini & G. Di Stefano	1
	1
A. Biscaccianti	1
A. Biscaccianti	1
A. Biscaccianti	1
A. Biscaccianti	1
A. Pacifici	3
E. Migliaccio	1
E. Migliaccio	5
E. Migliaccio	4
E. Migliaccio	7
E. Pacieri	1
F. Hartig	1
G. Di Giulio	2
M. Cerruti	3
P. Luigioni	2
P. Luigioni	3
P. Luigioni	2
P. Luigioni	10
P. Maltzeff	1
P. Maltzeff	2
P. Maltzeff	1
P. Maltzeff	1
P. Maltzeff	1
P. Maltzeff	2
P. Maltzeff	2
P. Maltzeff	1
P. Maltzeff	2
P. Maltzeff	2
P. Maltzeff	4
P. Maltzeff	1
P. Maltzeff	1
P. Maltzeff	1
P. Maltzeff	1
S. Bruno	2
S. Fattorini	1
S. Patrizi & M. Cerruti	5
U. Pessolano	1
U. Pessolano	1
	8
	2

	1
	2
	1
A. Pacifici	11
D. Cagiotti & L. Bertolaccini	1
D. Cagiotti & L. Bertolaccini	3
D. Cagiotti & L. Bertolaccini	1
D. Cagiotti & L. Bertolaccini	1
E. Colonnelli	2
G. M. Ghidini & M. Pavan	3
M. Zampetti	1
P. De Salvo	1
R. Antonelli	1
A. Pacifici	13
A. Pacifici	5
A. Pacifici	52
D. Cagiotti & L. Bertolaccini	2
D. Cagiotti & L. Bertolaccini	3
D. Cagiotti & L. Bertolaccini	6
D. Cagiotti & L. Bertolaccini	20
D. Cagiotti & L. Bertolaccini	30
D. Cagiotti & L. Bertolaccini	11
D. Cagiotti & L. Bertolaccini	30
D. Cagiotti & L. Bertolaccini	10
D. Cagiotti & L. Bertolaccini	9
D. Cagiotti & L. Bertolaccini	3
D. Cagiotti & L. Bertolaccini	1
D. Cagiotti & L. Bertolaccini	2
D. Cagiotti & L. Bertolaccini	28
D. Cagiotti & L. Bertolaccini	21
D. Cagiotti & L. Bertolaccini	15
D. Cagiotti & L. Bertolaccini	11
E. Migliaccio	7
G. Carpaneto	1
G. Carpaneto	1
G. Carpaneto	1
G. Carpaneto	1
G. Carpaneto	1
G. Carpaneto	1
G. Pace	2
G. Pace	1
G. Pace	2
P. De Salvo	7
P. De Salvo	2
P. De Salvo	3
P. De Salvo	1
P. De Salvo	1
P. De Salvo	3
P. De Salvo	1
P. De Salvo	1
P. De Salvo	7
P. Luigioni & A. Dodero	40
S. Fattorini & G. Di Stefano	4

Collection	Reference
CF	
CF	
CF	
MZUR	
MZUR	
MZUR	
MZUR	
MZUR	
MZUR	
MZUR	
MZUR	
MZUR	
MZUR	
MZUR	
MZUR	
MZUR	
MZUR	
MZUR	
MZUR	
MZUR	
MZUR	
MZUR	
MZUR	
MZUR	
MZUR	
MZUR	
MZUR	
MZUR	
MZUR	
MZUR	
MZUR	
MZUR	
MZUR	
MZUR	
MZUR	
MZUR	
MZUR	
MZUR	
MZUR	
MZUR	
MZUR	
MZUR	
MZUR	
MZUR	
MZUR	
MZUR	
CF	
MZUR	
CF	
MZUR	
MZUR	

MZUR
MZUR
MZUR
MZUR
MZUR
MZUR
MZUR
MZUR
MZUR
MZUR
MZUR
MZUR
MZUR
MZUR
MZUR
MZUR
MZUR
MZUR
MZUR
MZUR
MZUR
MZUR
MZUR
MZUR
MZUR
MZUR
MZUR
MZUR
MZUR
MZUR
MZUR
MZUR
MZUR
MZUR
MZUR
MZUR
MZUR
MZUR
MZUR
MZUR
CCO
MZUR
MZUR
CMZ
MZUR
MZUR
MZUR
MZUR
MZUR
MZUR
MZUR
MZUR
MZUR
MZUR

MZUR
MZUR
MZUR
MZUR
MZUR
MZUR
MZUR
CMI
CMI
CMI
CMI
CMI
MZUR
MZUR
MZUR
MZUR
MZUR
MZUR
MZUR
MZUR
MZUR
MZUR
MZUR
MZUR
MZUR
MZUR
MZUR
MZUR
CF
CF
CF
CF
CF
CF
CF
CF
CF
MZUR
CGA
CF
CF
CF
CF
CF
CF
CLE
CLE
CPNA
CF
MZUR
MZUR
CF
MZUR
MZUR

MZUR
MZUR
MZUR
MZUR
MZUR
MZUR
MZUR
CF
MZUR
UR3
UR3
UR3
UR3
CF
CF
CF
CLE
CLE
MZUR
CF
CLM
CLM
CLM
CF
CMZ
CMZ
CMZ
CMZ
CMZ
CMZ
CMZ
CMZ
CMZ
CMZ
CMZ
CMZ

CMZ
CMZ
CMZ
CMZ
MZUR
MZUR
MZUR
MZUR
MZUR
MZUR
MZUR
MZUR
MZUR
MZUR
MZUR
MZUR
MZUR

MZUR
MZUR
MZUR
MZUR
MZUR
MZUR
MZUR
MZUR
MZUR
MZUR
MZUR
MZUR
MZUR
MZUR
MZUR
MZUR
MZUR
MZUR
MZUR
MZUR
MZUR
MZUR
MZUR
MZUR
MZUR
MZUR
MZUR
MZUR
MZUR
MZUR
MZUR
MZUR
MZUR
MZUR
MZUR
MZUR
MZUR
MZUR
MZUR
MZUR
MZUR
MZUR
MZUR
MZUR
MZUR
MZUR
MZUR
MZUR
MZUR
MZUR
MZUR
MZUR
MZUR
MZUR

MZUR
MZUR
MZUR
MZUR
MZUR
MZUR
MZUR
MZUR
MZUR
MZUR
MZUR
MZUR
MZUR
CA
CA
CA
CLE
MZUR
MZUR
MZUR
MZUR
MZUR
MZUR
MZUR
MZUR
MZUR
MZUR
MZUR
MZUR
MZUR
MZUR
MZUR
MZUR
MZUR
MZUR
MZUR
MZUR
MZUR
MZUR
MZUR
MZUR
MZUR
MZUR
MZUR
MZUR
CF
CF
CF
CF
CF
CF
CF
CF
CF

CF
CF
CF
CF
CF
CF
CF
CF
CF
MZUR
MZUR
MZUR
MZUR
MZUR
MZUR
MZUR
MZUR
MZUR
MZUR
MZUR
MZUR
MZUR
MZUR
MZUR
MZUR
MZUR
MZUR
MZUR
MZUR
MZUR
MZUR
MZUR
MZUR
MZUR
MZUR
CLM
CLM
CLM
CLM
CLM
CLM
CLM
CVI
CVI
CVI
CLM
CLM
CVI
CRA
CGA
MZUR
MZUR
MZUR
MZUR

MZUR	
MZUR	
MZUR	
	Gardini 1997
	Gardini 1997
CF	
CF	
CF	
CF	
CLM	
CPE	
MZUR	
MZUR	
CLE	
MZUR	
MZUR	
CMI	
CMI	
CMI	
MZUR	
MZUR	
MZUR	
MZUR	
MZUR	
CLE	
CF	
CF	
CLE	
CNA	
MZUR	
MZUR	
MZUR	
MZUR	
MZUR	
MZUR	
CLE	
CLM	
CLM	
CLM	
CLM	
CLM	
CLM	
CMZ	
CMZ	
CMZ	
CMZ	
CMZ	
MZUR	
MZUR	
CA	
CLE	
CLE	
CRC	
CRC	

CF	
CF	
CF	
CNA	
CNA	
CNA	
CNA	
CNA	
CLM	
CPE	
CPE	
CPE	
	Viglioglia 2004
	Gardini 1997
	Gardini 1997
	Gardini 1997
	Gardini 1997
	Gardini 1997
CVI	
CLM	
	Luigioni 1921b
	Gardini 1997
CVI	
CGA	
CGA	
	Gardini 1997
MZUR	
MZUR	
MZUR	
	Gardini 1997
MZUR	
	Gardini 1997
	Gardini 1997
	Gardini 1997
	Gardini 1997
	Gardini 1997
MZUR	
	Gardini 1997
	Gardini 1997
	Gardini 1997
	Gardini 1997
	Gardini 1997
CMZ	
CLE	
CLE	
CLM	
CMI	
CF	
MZUR	

MZUR	
MZUR	
CF	
CLM	
CLM	
CLM	
	Luigioni 1921a
CLM	
CLM	
CLM	
CLM	
CLM	
CLM	
CF	
CMZ	
CMZ	
	Luigioni 1921a
	Luigioni 1921a
CLM	
CLM	
CF	
CNA	
CLM	
CLM	
MZUR	
CVI	
CVI	
CVI	
CLM	
	Gardini 1997
	Gardini 1997
	Gardini 1997
	Gardini 1997
	Gardini 1997
	Gardini 1997
CLE	
	Gardini 1997
	Gardini 1997
CLM	
	Leo 1984c
CLM	
MZUR	
	Cagiotti 1985; Bertolaccini 1986
	Cagiotti 1985; Bertolaccini 1986
	Cagiotti 1985; Bertolaccini 1986
	Cagiotti 1985; Bertolaccini 1986
	Cagiotti 1985; Bertolaccini 1986
	Cagiotti 1985; Bertolaccini 1986
	Cagiotti 1985; Bertolaccini 1986
	Cagiotti 1985; Bertolaccini 1986
	Cagiotti 1985; Bertolaccini 1986
	Cagiotti 1985; Bertolaccini 1986

	Cagiotti 1985; Bertolaccini 1986
	Cagiotti 1985; Bertolaccini 1986
	Cagiotti 1985; Bertolaccini 1986
	Cagiotti 1985; Bertolaccini 1986
	Cagiotti 1985; Bertolaccini 1986
	Cagiotti 1985; Bertolaccini 1986
	Cagiotti 1985; Bertolaccini 1986
	Cagiotti 1985; Bertolaccini 1986
	Cagiotti 1985; Bertolaccini 1986
	Cagiotti 1985; Bertolaccini 1986
	Cagiotti 1985; Bertolaccini 1986
	Cagiotti 1985; Bertolaccini 1986
	Cagiotti 1985; Bertolaccini 1986
	Cagiotti 1985; Bertolaccini 1986
CLE	
CLE	
CMI	
CCP	
	Binaghi 1964
MZUR	
MZUR	
MZUR	
MZUR	
CNA	
CLE	
CLM	
	De Salvo 1999
	De Salvo 1999
	De Salvo 1999
	De Salvo 1999
	De Salvo 1999
	De Salvo 1999
	De Salvo 1999
	De Salvo 1999
	De Salvo 1999
	De Salvo 1999
	De Salvo 1999
	De Salvo 1999
	De Salvo 1999
	De Salvo 1999
	De Salvo 1999
	De Salvo 1999
	De Salvo 1999
	De Salvo 1999
	De Salvo 1999
	De Salvo 1999
	De Salvo 1999
	De Salvo 1999
	De Salvo 1999
	De Salvo 1999
CF	
CF	
CF	
CF	

MZUR	
CLM	
MZUR	
MZUR	
MZUR	
MZUR	
MZUR	
MZUR	
MZUR	
MZUR	
CMI	
CMI	
CMI	
CPA	
CLM	
CLE	
	Andreetti et al. 1997
MZUR	
CLE	
MZUR	
MZUR	
MZUR	
MZUR	
MZUR	
MZUR	
MZUR	
MZUR	
MZUR	
MZUR	
MZUR	
MZUR	
MZUR	
MZUR	
MZUR	
MZUR	
MZUR	
CVI	
CF	
	Gridelli 1972
MZUR	
CLE	
	Gridelli 1972
CLM	
CLM	
MZUR	
MZUR	
MZUR	
CLE	
MZUR	
MZUR	
MZUR	
MZUR	
MZUR	
MZUR	

MZUR	
MZUR	
MZUR	
MZUR	
MZUR	
MZUR	
MZUR	
MZUR	
MZUR	
MZUR	
MZUR	
MZUR	
MZUR	
MZUR	
MZUR	
CF	
CF	
CF	
CF	
CF	
	Gridelli 1972
CF	
CF	
	Gridelli 1972
CF	
CNA	
CPA	
CPA	
CMI	
CMI	
MZUR	
	Gridelli 1972
MZUR	
MZUR	
MZUR	
MZUR	
MZUR	
MZUR	
MZUR	
MZUR	
MZUR	
MZUR	
MZUR	
	Gridelli 1972
	Gridelli 1972
CLM	
CLM	
CLM	
MZUR	
CMZ	
CF	
CF	
CF	
CMZ	

CMZ	
CMZ	
CMZ	
CF	
CMZ	
CMZ	
CMZ	
CMZ	
CMZ	
CMZ	
CMZ	
CMZ	
CMZ	
CMZ	
CMZ	
CMZ	
CMZ	
CMZ	
CMZ	
CMZ	
CMZ	
CMZ	
CMZ	
CMZ	
CMZ	
CMZ	
CMZ	
CA	
MZUR	
MZUR	
CF	
CF	
CF	
CF	
MZUR	
	Gridelli 1972
CVI	
	Luigioni & Tirelli 1910
CVI	
CVI	
	Gridelli 1972
MZUR	
	Gardini 1997
MZUR	
CO	
CO	
CO	
CO	
CO	
CO	
CO	
CPA	
MZUR	
MZUR	

CA	
CLM	
MZUR	
CA	
CLE	
CLE	
CMA	
CMA	
CMI	
	Gridelli 1972
	Canzoneri 1977
	Canzoneri 1977
	Canzoneri 1977
CLI	
CLE	
CMI	
	Gridelli 1972
CO	
CMI	
CMI	
CLE	
CMI	
CLE	
CMI	
CLE	
CLE	
CF	
CLM	
CLM	
CLM	
CLM	
CLM	
CA	
CA	
	Gridelli 1972
	Gridelli 1972
	Gridelli 1972
	Gridelli 1972
	Gridelli 1972
CRA	
CLE	
CLE	
CLM	
CPA	
CLE	
MZUR	
CLE	
CMI	
CAN	
CLE	
CLM	

CLM

MZUR
MZUR

MZUR
MZUR
CF
CF
CF
CF
CF
CF
CF
MZUR
MZUR
CLM
MZUR
MZUR
CNA
CNA
CF
MZUR
CCO
CLE
MZUR
MZUR
MZUR
MZUR
CMI
CF
CPNA
CF
CF
CF
MZUR
MZUR
MZUR
MZUR
MZUR
MZUR
MZUR
MZUR
MZUR
CF
CF
CF
CF
CF
CNA
CNA
CNA

CNA
CNA
CNA
CNA
CNA
CF
CNA
CNA
CNA
CNA
CNA
CF
CMI
MZUR
MZUR
CF
MZUR
MZUR
MZUR
MZUR
MZUR
MZUR
MZUR
MZUR
MZUR
CF
MZUR
MZUR
MZUR
MZUR
MZUR
MZUR
MZUR
CLM
CLE
CPE
CLM
CLM
CLM
CMZ
CMZ
CMZ
CMZ
CMZ
CMZ
CMZ
CMZ
CMZ
CMZ
CMZ
CMZ
CMZ
CMZ
CMZ

CMZ
CMZ
CMZ
MZUR
CA
CA
CF
CF
CF
CF
CF
CF
CF
CF
CF
CF
CF
CF
CF

CF
CF
CF
CF
CF
CNA
CNA
CNA
CNA
CNA
CNA
CNA
CNA
CF
CF
CNA
CNA
CNA
CNA
CNA
CNA
CNA
CNA
CF
CNA
MZUR
MZUR
CPE
CPE
CPE
CPE
CPE
CPE
CPE

CPE	
MZUR	
	Viglioglia 2004
CLM	
CLM	
CGA	
CGA	
CGA	
CGA	
CGA	
MZUR	
MZUR	
MZUR	
MZUR	
MZUR	
MZUR	
MZUR	
MZUR	
MZUR	
MZUR	
MZUR	
MZUR	
MZUR	
	Marcuzzi 1985
	Gardini 1997
	Gardini 1997
	Gardini 1997
MZUR	
MZUR	
MZUR	
MZUR	
CLE	
	Gardini 1997
	Gardini 1997
	Gardini 1997
CBE	
MZUR	
CMI	
MZUR	
CLM	
CPE	
MZUR	
CLE	
CLM	
CMZ	
	Gardini 1997
	Gardini 1997
	Gardini 1997
MZUR	
CMZ	
CLM	
CPNA	
MZUR	
CNA	

CLE	
CLM	
	Marcuzzi 1985a
	Gardini 1997
MZUR	
CNA	
	Magrone & Papini 1966
MZUR	
CLE	
CLM	
CMZ	
MZUR	
CF	
CF	
CF	
CF	
CF	
CF	
	Viglioglia 2004
MZUR	
MZUR	
	Gardini 1997
MZUR	
	Gardini 1997
CLM	
CLM	
CLM	
CLM	
CLE	
CNA	
CMI	
CLE	
CNA	
CPA	
CLE	
CF	
CF	
CMZ	
CLM	
CLM	
CLM	
CLM	
CVI	
CVI	
CVI	
CLM	
CF	
CF	
CF	
CF	
CF	
MZUR	
CLE	

MZUR	
MZUR	
MZUR	
MZUR	
MZUR	
MZUR	
MZUR	
MZUR	
MZUR	
MZUR	
MZUR	
MZUR	
MZUR	
CLE	
CMZ	
MZUR	
CF	
CMI	
CMI	
CMI	
CMI	
CMI	
CMI	
CMI	
CMI	
CMI	
CMI	
CMI	
MZUR	
MZUR	
MZUR	
MZUR	
CF	
CF	
CF	
CNA	
CNA	
CNA	
CNA	
CNA	
CNA	
	Bertolaccini 1986
MZUR	
MZUR	
MZUR	
MZUR	
MZUR	
MZUR	
MZUR	
UR3	
UR3	
MZUR	
CLE	

MZUR	
	De Salvo 1999
CMZ	
CMZ	
CF	
CMZ	
CMZ	
CMZ	
CMZ	
CF	
CF	
MZUR	
CA	
CA	
CA	
CLE	
CF	
CF	
MZUR	
MZUR	
MZUR	
MZUR	
MZUR	
MZUR	
MZUR	
MZUR	
MZUR	
CLI	
CLM	
CLM	
CLM	
CLM	
CVI	
CLM	
MZUR	
MZUR	
CGA	
CVI	
MZUR	
CGA	
MZUR	
MZUR	
MZUR	
MZUR	
MZUR	
	Gardini 1997
CPA	
CLE	
MZUR	
CLM	
CLM	
CLM	

CVI
CMI
CLE
CPA
CMA
CVI
CF
CF
MZUR
MZUR
MZUR
MZUR
MZUR
MZUR
CNA
CLE
CLM
CLM
CLM
CLM
CLM
CLM
MZUR
MZUR
MZUR
MZUR
MZUR
MZUR
MZUR
MZUR
MZUR
MZUR
MZUR
MZUR
MZUR
MZUR
MZUR
MZUR
MZUR
CLE
CMZ
MZUR
MZUR
MZUR
MZUR
MZUR
MZUR
MZUR
MZUR
MZUR
MZUR
MZUR
MZUR
MZUR

MZUR
MZUR
MZUR
MZUR
MZUR
MZUR
MZUR
MZUR
MZUR
MZUR
MZUR
MZUR
CMI
CMI
CMI
CMI
CMI
CMI
CCP
MZUR
MZUR
MZUR
MZUR
MZUR
MZUR
MZUR
MZUR
CF
CF
CF
CF
CF
CF
CF
CF
CF
MZUR
MZUR
MZUR
MZUR
MZUR
CF
CLE
CLE
CMI
CMI
CNA
CNA
CPNA
MZUR
MZUR
MZUR
MZUR
MZUR

MZUR
MZUR
MZUR
MZUR
UR3
UR3
UR3
UR3
UR3
UR3
MZUR
MZUR
CMZ
CMZ
CMZ
CMZ
CMZ
CMZ
CMZ
CMZ
CMZ
CMZ
CMZ
CMZ
CMZ
CMZ
CMZ
CMZ
CMZ
CMZ
CMZ
MZUR
MZUR
MZUR
MZUR
MZUR
MZUR
MZUR
MZUR
MZUR
MZUR
MZUR
MZUR
MZUR
MZUR
MZUR
MZUR
MZUR
MZUR
MZUR
MZUR
MZUR
MZUR
MZUR

MZUR
CA
MZUR
MZUR
MZUR
MZUR
MZUR
MZUR
MZUR
MZUR
MZUR
MZUR
MZUR
MZUR
MZUR
MZUR
CF
CF
CF
CF
MZUR
MZUR
MZUR
MZUR
MZUR
MZUR
MZUR
MZUR
MZUR
MZUR
MZUR
MZUR
MZUR
MZUR
MZUR
MZUR
MZUR
MZUR
MZUR
MZUR
MZUR
MZUR
MZUR
MZUR
MZUR
MZUR
MZUR
CLM
CVI
CGA
CGA
CGA
MZUR
MZUR

CVI	
CLE	
	Gardini 1997
CF	
CLE	
MZUR	
CLM	
CLM	
CLM	
CLM	
CLM	
CLM	
CGA	
CGA	
CMI	
CLE	
CLM	
CLM	
CMI	
CMI	
MZUR	
CLM	
MZUR	
CLM	
CLM	
CLM	
CLM	
CA	
CA	
CF	
MZUR	
CLM	
CLM	
CLM	
CLM	
CLM	
CLM	
CGA	
	Gardini 1997
CLE	
CPNA	
CVI	
CME	Leo 1991b
CMI	
MZUR	
CF	
CMI	
	Leo 1991b
MZUR	
MZUR	
MZUR	
MZUR	
CGA	
CLM	

MZUR	
MZUR	
MZUR	
CLM	
CMZ	
CLM	
CLM	
CVI	
	Gardini 1997
	Gardini 1997
	Gardini 1997
	Gardini 1976
MZUR	
CLM	
CO	
CGA	
CLE	
MZUR	
CF	
MZUR	
MZUR	
MZUR	
MZUR	
MZUR	
CGA	
CMI	
CMI	
CMI	
CMI	
CMI	
MZUR	
MZUR	
CF	
MZUR	
MZUR	
CF	
MZUR	
CA	
CA	
CA	
CA	
CF	
MZUR	
MZUR	
MZUR	
MZUR	
MZUR	
MZUR	
MZUR	
MZUR	
MZUR	
MZUR	
MZUR	
CVI	

CVI	
CF	
CLI	
CMI	
CMI	
MZUR	
CMZ	
CMZ	
CMZ	
CA	
CPE	
	Viglioglia 2004
	Viglioglia 2004
MZUR	
MZUR	
CLE	
MZUR	
CMI	
CLE	
CLE	
CF	
CF	
CF	
CF	
CMI	
CMI	
CMI	
MZUR	
CF	
MZUR	
MZUR	
CMI	
CLM	
CLM	
CLM	
CLM	
CLM	
CMZ	
CMZ	
CMZ	
CMZ	
CMZ	
CF	
CMZ	
CMZ	
CMZ	
CMZ	
CLM	
CVI	
CGA	
CGA	
MZUR	
CVI	
CMZ	

MZUR
CLE
CLE
CLE
MZUR
MZUR
MZUR
MZUR
CLM

MZUR
CMI
CLM
CLM
CF
CF

CF
MZUR
CNA
CNA
CNA
CNA
CNA
CNA
CLE
CLM
MZUR
MZUR

Cagiotti 1985; Bertolaccini 1986
Cagiotti 1985; Bertolaccini 1986
Cagiotti 1985; Bertolaccini 1986
Cagiotti 1985; Bertolaccini 1986
Cagiotti 1985; Bertolaccini 1986
Cagiotti 1985; Bertolaccini 1986
Cagiotti 1985; Bertolaccini 1986
Cagiotti 1985; Bertolaccini 1986
Cagiotti 1985; Bertolaccini 1986
Cagiotti 1985; Bertolaccini 1986
Cagiotti 1985; Bertolaccini 1986
Cagiotti 1985; Bertolaccini 1986
Cagiotti 1985; Bertolaccini 1986
Cagiotti 1985; Bertolaccini 1986
Cagiotti 1985; Bertolaccini 1986
Cagiotti 1985; Bertolaccini 1986
Cagiotti 1985; Bertolaccini 1986
Cagiotti 1985; Bertolaccini 1986
Cagiotti 1985; Bertolaccini 1986
Cagiotti 1985; Bertolaccini 1986
Cagiotti 1985; Bertolaccini 1986
Cagiotti 1985; Bertolaccini 1986
Cagiotti 1985; Bertolaccini 1986

CMZ
CMI

CMI	
CCP	
MZUR	
	Binaghi 1964
MZUR	
MZUR	
MZUR	
MZUR	
MZUR	
MZUR	
MZUR	
MZUR	
CLE	
CNA	
CNA	
CNA	
CNA	
CNA	
CMI	
MZUR	
MZUR	
MZUR	
MZUR	
MZUR	
MZUR	
MZUR	
	De Salvo 1999
	De Salvo 1999
	De Salvo 1999
	De Salvo 1999
	De Salvo 1999
	De Salvo 1999
	De Salvo 1999
	De Salvo 1999
	De Salvo 1999
	De Salvo 1999
	De Salvo 1999
	De Salvo 1999
	De Salvo 1999
	De Salvo 1999
	De Salvo 1999
	De Salvo 1999
CLM	
CLM	
CMZ	
CMZ	
CMZ	
CMZ	
CMZ	
CMZ	
MZUR	
CA	
CLM	

CLM	
CLE	
CF	
CF	
CF	
CF	
CF	
CF	
CLE	
MZUR	
MZUR	
CPE	
CPE	
MZUR	
CVI	
CVI	
MZUR	
CGA	
CGA	
MZUR	
MZUR	
MZUR	
MZUR	
MZUR	
MZUR	
MZUR	
MZUR	
MZUR	
MZUR	
CLE	
CF	
CPE	
CPA	
CLE	
CCO	
MZUR	
CF	
CMI	
CMI	
MZUR	
MZUR	
	Bertolaccini 1986
MZUR	
MZUR	
MZUR	
MZUR	
MZUR	
MZUR	
CLM	
CLM	
CLM	
CLM	
CMZ	
MZUR	

CA	
CLE	
CMI	
CLE	
CMI	
MZUR	
MZUR	
MZUR	
MZUR	
MZUR	
CF	
CPE	
	Viglioglia 2004
CLM	
CLM	
CLM	
CVI	
CVI	
CGA	
MZUR	
MZUR	
MZUR	
MZUR	
MZUR	
MZUR	
MZUR	
MZUR	
MZUR	
	Gardini 1997
CLM	
CF	
MZUR	
MZUR	
CF	
CF	
MZUR	
MZUR	
	Canzoneri 1959
MZUR	
MZUR	
MZUR	
CLE	
CMZ	
MZUR	
MZUR	
MZUR	
MZUR	
CF	
CVI	
CVI	
CVI	
CGA	
CGA	
MZUR	

MZUR	
CF	
	Leo 1983
MZUR	
	Cagiotti 1985; Bertolaccini 1986
	Cagiotti 1985; Bertolaccini 1986
	Cagiotti 1985; Bertolaccini 1986
	Cagiotti 1985; Bertolaccini 1986
	Cagiotti 1985; Bertolaccini 1986
	Cagiotti 1985; Bertolaccini 1986
	Cagiotti 1985; Bertolaccini 1986
	Cagiotti 1985; Bertolaccini 1986
	Cagiotti 1985; Bertolaccini 1986
	Cagiotti 1985; Bertolaccini 1986
	Cagiotti 1985; Bertolaccini 1986
	Cagiotti 1985; Bertolaccini 1986
	Cagiotti 1985; Bertolaccini 1986
	Cagiotti 1985; Bertolaccini 1986
	Cagiotti 1985; Bertolaccini 1986
	Cagiotti 1985; Bertolaccini 1986
	Cagiotti 1985; Bertolaccini 1986
	Cagiotti 1985; Bertolaccini 1986
	Cagiotti 1985; Bertolaccini 1986
	Cagiotti 1985; Bertolaccini 1986
	Cagiotti 1985; Bertolaccini 1986
	Cagiotti 1985; Bertolaccini 1986
	Cagiotti 1985; Bertolaccini 1986
	Cagiotti 1985; Bertolaccini 1986
CMI	
CCP	
MZUR	
CGA	
	Binaghi 1964
MZUR	
MZUR	
	De Salvo 1999
	De Salvo 1999
	De Salvo 1999
	De Salvo 1999
	De Salvo 1999
	De Salvo 1999
	De Salvo 1999
	De Salvo 1999
	De Salvo 1999
	De Salvo 1999
	De Salvo 1999
	De Salvo 1999
	De Salvo 1999
	De Salvo 1999
	De Salvo 1999
	De Salvo 1999
	De Salvo 1999
	De Salvo 1999
	De Salvo 1999

	De Salvo 1999
	De Salvo 1999
	De Salvo 1999
	De Salvo 1999
	De Salvo 1999
CF	
CF	
CF	
CF	
CF	
CLM	
MZUR	
CLM	
CGA	
CF	
CF	
CF	
MZUR	
MZUR	
MZUR	
MZUR	
MZUR	
MZUR	
MZUR	
MZUR	
MZUR	
CF	
MZUR	
CLE	
MZUR	
MZUR	
MZUR	
MZUR	
CNA	
MZUR	
MZUR	
MZUR	
MZUR	
CCO	
CLE	
CF	
CF	
MZUR	
MZUR	
MZUR	
MZUR	
MZUR	
MZUR	
MZUR	
MZUR	
MZUR	
MZUR	
MZUR	
CMI	

CMI
CMI
CMI
CMI
CMI
CMI
CMI
CMI
CMI
MZUR
MZUR
MZUR
MZUR
CF
CF
CF
CF
CLE
CLE
CNA
CNA
CNA
CLE
CLM
MZUR
MZUR
MZUR
MZUR
MZUR
MZUR
MZUR
CF
CF
CGA
CLM
CLM
CF
CF
CLM
CLM
CLM
CLM
CLM
CLM
CMZ
CMZ
CMZ
CMZ
CMZ
CMZ
CMZ
CMZ
CMI
CMZ

CMZ
CMZ
CMZ
CMZ
CMZ
CMZ
CMZ
CMZ
CMZ
CMZ
CMZ

CMZ
MZUR
MZUR
MZUR
MZUR
MZUR
MZUR
MZUR
MZUR
MZUR
MZUR
CA
CA
CA
CA
MZUR
MZUR
MZUR
CF
CF
CF
CF
MZUR
MZUR
CLM
CLM
CLM
CLM
CLM
CVI
CLM
CVI
CRA
CGA
CGA
MZUR
CGA
MZUR
CF
CNA
CLM
CLM

CLM	
CLM	
MZUR	
MZUR	
CPNA	
CLE	
CNA	
CF	
CLE	
CLE	
CLM	
CLM	
MZUR	
MZUR	
CLM	
CLM	
CLM	
	Nardi 1994
	Nardi 1994
MZUR	
MZUR	
CMI	
CMI	Nardi 1994
CMI	
CMI	
CMI	
	Nardi 1994
	Nardi 1994
	Cassola 1965
	Cassola 1965
	Cassola 1965
	Gardini 1976e
CLM	
CMI	
	Nardi 1994
	Nardi 1994
	Nardi 1994
CLE	
MZUR	
MZUR	
	Luigioni & Tirelli 1910
MZUR	
MZUR	
CF	
CMZ	
CMZ	
CF	
CF	
CMZ	
CMZ	
CMZ	
CF	
CLE	

CMZ	
CMZ	
CMZ	
CMZ	
CMZ	
CMZ	
MZUR	
CLE	
CLM	
MZUR	
MZUR	
MZUR	
	Gardini 1976e
CLM	
CGA	
MZUR	
MZUR	
MZUR	
MZUR	
MZUR	
CF	
CF	
CF	
MZUR	
CLM	
MZUR	
MZUR	
CVI	
MZUR	
CLM	
CLE	
	Ciampi 1905
MZUR	
	Binaghi 1964
CVI	
CLM	
CLE	
CLE	
CLE	
MZUR	
CCP	
CF	
CF	
CLM	
CPA	
CPA	
CF	
MZUR	
CMZ	
CMZ	
CF	
CMZ	
CA	
CF	

CLM	
CLM	
CGA	
CGA	
CLE	
MZUR	Marcuzzi 1985a
CF	
CF	
CF	
CF	
MZUR	
CF	
CLE	
CF	
MZUR	
CCO	
MZUR	
MZUR	
CMI	
CMI	
CMI	
CMI	
CMI	
CMI	
CMI	
CMI	
CMI	
MZUR	
CF	
CF	
CF	
CF	
CF	
CF	
CNA	
CLE	
MZUR	
CNA	
MZUR	
CMI	
MZUR	
MZUR	
UR3	
CMI	
CGA	
CLM	
CMZ	
CLE	
CF	
CMZ	
CMZ	
CMZ	
CMZ	

CMZ
CMZ
CF
CMZ
CMZ
CMZ
CMZ
CMZ
CF
CMZ
CMZ
CMZ
CF
CA
CA
CF
CF
CF
CF
CF
CF
CF
MZUR
CF

Gardini 1997

CF
MZUR
MZUR
CLE
MZUR
MZUR
CMI
CMI
CMI
CMI
CMI
CMI
CMI
MZUR
CLE
CF
CF
CF
CNA
CF
CNA
CF
CLE
CNA
CLE
CLE
MZUR
MZUR
MZUR

MZUR	
CF	
UR3	
CLE	
CLM	
MZUR	
CLM	
CF	
CF	
CF	
CA	
CA	
CF	
	Gardini 1997
CLM	
	Gardini 1997
CVI	
CLM	
CLM	
MZUR	
MZUR	
MZUR	
MZUR	
MZUR	
MZUR	
MZUR	
MZUR	
MZUR	
	Gardini 1997
CMZ	
	Gardini 1997
CLE	
	Gardini 1997
CF	
CMI	
CMI	
CPA	
CF	
CF	
CMZ	
CF	
CMZ	
CPE	
CMZ	
CLE	
CNA	
CPA	
CLM	
CLM	
CLM	
CLM	
CLM	

CLM
CPA
CLE
MZUR
MZUR
CF
CF
CMZ
CMZ
CF
CMZ
CMZ
MZUR
CLM
CLM
MZUR
CO
CLE
CMI
MZUR
CF
CLE
CMZ
CMZ
CLE
MZUR
MZUR
MZUR
MZUR
MZUR
CMI
CMI
CMI
CMI
MZUR
MZUR
MZUR
CNA
CNA
CNA
CNA
CNA
CNA
CNA
CNA
CF
CNA
CNA
CNA
CF
CNA
CNA
CNA
CNA

CPA
CMI
CMI
CMI
CPNA
MZUR
MZUR
MZUR
MZUR
MZUR
MZUR
MZUR
CNA
CLE
CLM
CF
CMZ
CMZ
CLE
CMZ
CMZ

CMZ
CMZ
CMZ
CA
CNA
MZUR
MZUR
MZUR
CF
MZUR
CPE
CLM
CLM
CVI
CGA
MZUR
MZUR
MZUR
MZUR
MZUR
MZUR
MZUR
MZUR
MZUR
MZUR
MZUR
MZUR

Gardini 1997
Gardini 1997
Gardini 1997
Gardini 1997
Gardini 1997

	Canzoneri 1977
CLE	
CLE	
CF	
CF	
CF	
	Leo 1982c
CF	
CLE	
CLE	
CLE	
MZUR	
CLE	
CLE	
CLM	
CLM	
CLM	
CLM	
MZUR	
CGA	
CF	
CF	
CLE	
MZUR	
MZUR	
CF	
MZUR	
MZUR	
MZUR	
MZUR	
MZUR	
MZUR	
CCO	
CCO	
MZUR	
	Andreetti et al. 1997
CMZ	
MZUR	
MZUR	
MZUR	
MZUR	
MZUR	
MZUR	
MZUR	
MZUR	
MZUR	
CMI	
CMI	
CMI	
	Binaghi 1964
MZUR	
CF	
CF	
CF	

CF
CF
CMI
CLE
MZUR
MZUR
MZUR
MZUR
MZUR
MZUR
MZUR
MZUR
MZUR
MZUR
MZUR
MZUR
MZUR
MZUR
UR3
UR3
CLE
CLE
MZUR
MZUR
CLE
CLE
CMZ
CMZ
CMZ
CMZ
CMZ
CMZ
CMZ
CMZ
CMZ
CMZ
CMZ
CMZ
CMZ
CMZ
CMZ
CMZ
CMZ
CMZ
CMZ
CA
CA
CA
CLE
CLE
MZUR
MZUR
MZUR
MZUR

MZUR	
MZUR	
MZUR	
MZUR	
CF	
CF	
CF	
CF	
CF	
MZUR	
MZUR	
MZUR	
MZUR	
CPE	
CLM	
CLM	
CLM	
CLM	
CLM	
CVI	
CVI	
CVI	
CGA	
CRA	
	Gardini 1997
CGA	
CGA	
CPE	
MZUR	
MZUR	
MZUR	
MZUR	
MZUR	
MZUR	
MZUR	
MZUR	
MZUR	
MZUR	
MZUR	
MZUR	
MZUR	
MZUR	
MZUR	
MZUR	
CVI	
CPE	
MZUR	
	Gardini 1997
	Canzoneri 1977
	Canzoneri 1977
	Canzoneri 1977
	Gardini 1997
MZUR	
CPE	

CLE	
CPA	
CLM	
CLM	
CLM	
CLE	
CLM	
CLM	
CPA	
CF	
CPNA	
CF	
	Cagiotti 1985; Bertolaccini 1986
	Cagiotti 1985; Bertolaccini 1986
	Cagiotti 1985; Bertolaccini 1986
	Cagiotti 1985; Bertolaccini 1986
	Cagiotti 1985; Bertolaccini 1986
	Cagiotti 1985; Bertolaccini 1986
	Cagiotti 1985; Bertolaccini 1986
	Cagiotti 1985; Bertolaccini 1986
	Cagiotti 1985; Bertolaccini 1986
	Cagiotti 1985; Bertolaccini 1986
	Cagiotti 1985; Bertolaccini 1986
	Cagiotti 1985; Bertolaccini 1986
	Cagiotti 1985; Bertolaccini 1986
	Cagiotti 1985; Bertolaccini 1986
	Cagiotti 1985; Bertolaccini 1986
	Cagiotti 1985; Bertolaccini 1986
	Cagiotti 1985; Bertolaccini 1986
	Cagiotti 1985; Bertolaccini 1986
	Cagiotti 1985; Bertolaccini 1986
	Cagiotti 1985; Bertolaccini 1986
CLE	
CMI	
MZUR	
MZUR	
CF	
CNA	
CPA	
CPA	
CLE	
CLE	
CLE	
MZUR	
MZUR	
CLE	
CLE	
	De Salvo 1999
	De Salvo 1999
	De Salvo 1999
	De Salvo 1999
	De Salvo 1999
MZUR	
CLM	

CF	
CF	
CF	
CGA	
CVI	
CLM	
CLM	
CLM	
CVI	
MZUR	
MZUR	
CLM	
MZUR	
	Cagiotti 1985; Bertolaccini 1986
	Cagiotti 1985; Bertolaccini 1986
	Cagiotti 1985; Bertolaccini 1986
	Cagiotti 1985; Bertolaccini 1986
	Cagiotti 1985; Bertolaccini 1986
	Cagiotti 1985; Bertolaccini 1986
	Cagiotti 1985; Bertolaccini 1986
	Cagiotti 1985; Bertolaccini 1986
	Cagiotti 1985; Bertolaccini 1986
	Cagiotti 1985; Bertolaccini 1986
	Cagiotti 1985; Bertolaccini 1986
	Cagiotti 1985; Bertolaccini 1986
	Cagiotti 1985; Bertolaccini 1986
	Cagiotti 1985; Bertolaccini 1986
	Cagiotti 1985; Bertolaccini 1986
	Cagiotti 1985; Bertolaccini 1986
	Cagiotti 1985; Bertolaccini 1986
	Cagiotti 1985; Bertolaccini 1986
	Cagiotti 1985; Bertolaccini 1986
	Cagiotti 1985; Bertolaccini 1986
CLE	
CLE	
CMI	
CMI	
CMI	
CMI	
MZUR	
MZUR	
MZUR	
MZUR	
	Canzoneri 1961c
	Canzoneri 1968b
CPA	
CLE	
CGA	
CLE	
CMI	
CMI	
	De Salvo 1999
	De Salvo 1999

CLM	
MZUR	
CA	
CNA	
CF	
MZUR	
CGA	
CGA	
CGA	
CVI	
CVI	
	Leo 1990
MZUR	
MZUR	
CLM	
CF	
CLM	
CLM	
CVI	
MZUR	
CVI	
CF	
CF	
CF	
CF	
CLE	
CF	
CNA	
CNA	
CNA	
CNA	
CNA	
CNA	
CNA	
CLE	
CMZ	
MZUR	
MZUR	
MZUR	
MZUR	
MZUR	
MZUR	
CLE	
	Cagiotti 1985; Bertolaccini 1986
	Cagiotti 1985; Bertolaccini 1986
	Cagiotti 1985; Bertolaccini 1986
	Cagiotti 1985; Bertolaccini 1986
	Cagiotti 1985; Bertolaccini 1986
	Cagiotti 1985; Bertolaccini 1986
	Cagiotti 1985; Bertolaccini 1986
	Cagiotti 1985; Bertolaccini 1986
	Cagiotti 1985; Bertolaccini 1986
	Cagiotti 1985; Bertolaccini 1986
	Cagiotti 1985; Bertolaccini 1986

	Cagiotti 1985; Bertolaccini 1986
	Cagiotti 1985; Bertolaccini 1986
	Cagiotti 1985; Bertolaccini 1986
	Cagiotti 1985; Bertolaccini 1986
	Cagiotti 1985; Bertolaccini 1986
	Cagiotti 1985; Bertolaccini 1986
	Cagiotti 1985; Bertolaccini 1986
	Cagiotti 1985; Bertolaccini 1986
	Cagiotti 1985; Bertolaccini 1986
MZUR	
CMI	
CMI	
CMI	
CLE	
CMI	
CMI	
CCP	
CNA	
CLM	
MZUR	
	Binaghi 1964
MZUR	
MZUR	
MZUR	
MZUR	
MZUR	
MZUR	
CF	
CF	
CF	
CF	
CNA	
CNA	
CNA	
CNA	
CNA	
CNA	
CNA	
CPNA	
CMI	
CMI	
MZUR	
MZUR	
MZUR	
MZUR	
MZUR	
MZUR	
MZUR	
MZUR	
MZUR	
MZUR	
MZUR	
MZUR	
MZUR	

UR3	
UR3	
UR3	
UR3	
CLE	
MZUR	
MZUR	
	De Salvo 1999
	De Salvo 1999
	De Salvo 1999
	De Salvo 1999
	De Salvo 1999
	De Salvo 1999
	De Salvo 1999
	De Salvo 1999
	De Salvo 1999
	De Salvo 1999
	De Salvo 1999
	De Salvo 1999
	De Salvo 1999
	De Salvo 1999
	De Salvo 1999
	De Salvo 1999
CLM	
CLM	
CLM	
CLM	
CMZ	
CMZ	
CMZ	
CMZ	
CMZ	
CMZ	
CMZ	
CMZ	
CPNA	
MZUR	
MZUR	
CA	
CA	
CA	
CLE	
CF	
CF	
CF	
CF	
CF	
CF	
CF	
MZUR	
MZUR	
MZUR	
MZUR	

MZUR	
MZUR	
CPE	
CPE	
CPE	
CPE	
CPE	
CPE	
	Aliquò 1990
CVI	
CGA	
MZUR	
MZUR	
MZUR	
MZUR	
MZUR	
MZUR	
MZUR	
MZUR	
MZUR	
MZUR	
MZUR	
MZUR	
MZUR	
MZUR	
	Marcuzzi 1952
CVI	
CVI	
	Marcuzzi 1985a
CLE	
CNA	
MSF	
CLM	
CGA	
MZUR	
MZUR	
CMI	
CMI	
MZUR	
CLM	
CLE	
MZUR	
MZUR	
CMI	
CLM	
CMZ	
CMZ	
CMZ	
CMZ	
CA	
CF	
CLM	
CVI	
CLM	

	Leo 1982a
	Leo 1982a
	Leo 1982a
CLM	
CLM	
CF	
MZUR	
	Cagiotti 1985; Bertolaccini 1986
	Cagiotti 1985; Bertolaccini 1986
	Cagiotti 1985; Bertolaccini 1986
	Cagiotti 1985; Bertolaccini 1986
	Cagiotti 1985; Bertolaccini 1986
	Cagiotti 1985; Bertolaccini 1986
	Cagiotti 1985; Bertolaccini 1986
	Cagiotti 1985; Bertolaccini 1986
	Cagiotti 1985; Bertolaccini 1986
	Cagiotti 1985; Bertolaccini 1986
	Cagiotti 1985; Bertolaccini 1986
	Cagiotti 1985; Bertolaccini 1986
	Cagiotti 1985; Bertolaccini 1986
	Cagiotti 1985; Bertolaccini 1986
	Cagiotti 1985; Bertolaccini 1986
	Binaghi 1964
MZUR	
MZUR	
CLE	
CLE	
CLE	
	Leo 1982a
	Leo 1982a
	De Salvo 1999
	De Salvo 1999
	De Salvo 1999
	De Salvo 1999
	De Salvo 1999
	De Salvo 1999
	De Salvo 1999
	De Salvo 1999
	De Salvo 1999
	De Salvo 1999
CF	
CF	
CF	
CF	
CMI	
CMI	
CMI	
CMI	
CF	
CF	
CF	
CF	
CLM	
CLM	

CRA
MZUR
MZUR
CF
CF
MZUR
CF
CF
CF
CF
CF
CLM
CMI
CMI
CMI
CLM
MZUR
MZUR
CF
MZUR
CF
MZUR
MZUR
MZUR
MZUR
MZUR
MZUR
CLM
CLM
CLM
CLM
CLM
CLM
CLM
CLM
CLM
CLM
CMZ
CMZ
CMZ
CMZ
CMZ
CMZ
CMZ
CMZ
CMZ
CMZ
CMZ
CMZ
CMZ
CMZ
CMZ
CMZ
CMZ

CMZ	
CMZ	
MZUR	
CA	
CA	
CLM	
CLE	
CF	
CF	
CF	
CF	
CNA	
MZUR	
	Viglioglia 2004
	Gardini 1997
	Gardini 1997
	Gardini 1997
	Gardini 1997
CVI	
CVI	
CVI	
MZUR	
MZUR	
MZUR	
MZUR	
MZUR	
MZUR	
MZUR	
CPE	
MZUR	
MZUR	
MZUR	
MZUR	
MZUR	
MZUR	
MZUR	
CGA	
CPE	
MZUR	
MZUR	
MZUR	
MZUR	
	Marcuzzi 1952
	Gardini 1997
	Gardini 1997
	Marcuzzi 1985a
	Marcuzzi 1985a
CPE	
	Gardini 1997
	Gardini 1997
	Gardini 1997
CF	

MZUR
MZUR
MZUR
MZUR
MZUR
MZUR
MZUR
MZUR
MZUR
MZUR
MZUR
MZUR
CLE
CNA
CLE
MZUR
MZUR
MZUR
MZUR
MZUR
MZUR
CMI
CMI
CMI
CPA
CLE
MZUR
MZUR
MZUR
CLM
CLM
CLM
MZUR
MZUR
CA
MZUR
MZUR
CF
CF
CLM
CVI
CVI
CVI
CVI
MZUR
CF
CLE
MZUR
CF
CF
MZUR
CNA
CNA

CNA	
CNA	
CNA	
CNA	
CNA	
CNA	
CNA	
MZUR	
MZUR	
MZUR	
MZUR	
MZUR	
MZUR	
MZUR	
MZUR	
CF	
MZUR	
CMZ	
CMZ	
CF	
MZUR	
MZUR	
MZUR	
MZUR	
	Gardini 1997
	Gardini 1997
CLE	
MZUR	
MZUR	
MZUR	
MZUR	
MZUR	
MZUR	
MZUR	
MZUR	
	Binaghi 1964
MZUR	
MZUR	
MZUR	
	Bertolaccini 1986
MZUR	
UR3	
UR3	
UR3	
UR3	
CF	
CF	
CF	
CF	
CF	
MZUR	
MZUR	
MZUR	
MZUR	

MZUR
MZUR
MZUR
MZUR
MZUR
MZUR
CF
CF
CF
MZUR
CLE
CLE
MZUR
MZUR
CMZ
MZUR
CMI
CF
CF
CF
CF
CF
CF
CF
CF
CF
CF
MZUR
CNA
CNA
CNA
CNA
CNA
CNA
CLE
CNA
CNA
MZUR
MZUR
MZUR
MZUR
MZUR
MZUR
MZUR
MZUR
MZUR
MZUR
UR3
UR3
UR3
UR3
UR3
UR3
CLE

CF	
MZUR	
MZUR	
MZUR	
MZUR	
CF	
CF	
CMZ	
CF	
CMZ	
CMZ	
CF	
CF	
CMZ	
CF	
CF	
CA	
CA	
CLE	
CMA	
CNA	
CF	
CF	
CF	
CF	
CF	
CF	
CF	
MZUR	
	Viglioglia 2004
	Gardini 1997
	Gardini 1997
	Gardini 1997
CGA	
CRA	
MZUR	
MZUR	
MZUR	
MZUR	
MZUR	
MZUR	
MZUR	
MZUR	
MZUR	
MZUR	
CF	
CF	
CF	
CF	
CF	
CF	
CF	
CLE	
MZUR	

CNA	
CNA	
CLE	
CLE	
CLM	
MZUR	
MZUR	
MZUR	
CPE	
MZUR	
MZUR	
MZUR	
MZUR	
MZUR	
MZUR	
MZUR	
MZUR	
CMI	
CMI	
CMI	
CMI	
CMI	
CMI	
CCP	
MZUR	
	Binaghi 1964
MZUR	
MZUR	
MZUR	
MZUR	
CF	
CF	
CLE	
CNA	
CNA	
CF	
CNA	
CLE	
CNA	
CNA	
CF	
CNA	
CLE	
MZUR	
MZUR	
MZUR	
MZUR	
MZUR	
MZUR	
MZUR	
MZUR	
MZUR	
UR3	
UR3	

UR3
UR3
UR3
UR3
UR3
UR3
UR3
CLE
CMI
MZUR
MZUR
CLM
CLM
CLM
CLM
CLM
CLM
CMZ
CMZ
CF
CLE
CMZ
CMZ
CMZ
CMZ
CMZ
CMZ
CMZ
CMZ
CMZ
CMZ
CMZ

MZUR
CA
CA
CA
CA
CA
CLE
CLE
CF
CF
CF
CF
CF
CF
CF
CF
CMZ
CLE
MZUR
MZUR
MZUR

MZUR	
MZUR	
MZUR	
CPE	
CPE	
CPE	
CVI	
CVI	
CGA	
CGA	
CVI	
MZUR	
MZUR	
MZUR	
MZUR	
MZUR	
MZUR	
MZUR	
MZUR	
MZUR	
MZUR	
MZUR	
MZUR	
MZUR	
MZUR	
MZUR	
MZUR	
MZUR	
CVI	
CVI	
	Marcuzzi 1985a
MZUR	
MZUR	
MZUR	
CF	
MZUR	
CCO	
MZUR	
CMZ	
MZUR	
CMI	
CMI	
CF	
	Gridelli 1950
CNA	
CNA	
CNA	
CNA	
CNA	
CNA	
CNA	
CNA	
CNA	
CNA	

CNA
CNA
CNA
MZUR
MZUR
MZUR
MZUR
MZUR
MZUR
MZUR
CLE
CLM
CLM
CLM
CLM
CLM
CLM
CLM
CLM
CLM
CLM
CLM
CF
CMZ
CF
CMZ
MZUR
CA
CA
CVI
CVI
MZUR
MZUR
MZUR
MZUR
MZUR
MZUR
MZUR
MZUR
MZUR
MZUR
MZUR
MZUR
CLE
CMI

Cagiotti 1985; Bertolaccini 1986
Cagiotti 1985; Bertolaccini 1986
Cagiotti 1985; Bertolaccini 1986
Cagiotti 1985; Bertolaccini 1986
Cagiotti 1985; Bertolaccini 1986
Cagiotti 1985; Bertolaccini 1986
Cagiotti 1985; Bertolaccini 1986
Cagiotti 1985; Bertolaccini 1986
Cagiotti 1985; Bertolaccini 1986

	Cagiotti 1985; Bertolaccini 1986
	Cagiotti 1985; Bertolaccini 1986
	Cagiotti 1985; Bertolaccini 1986
	Cagiotti 1985; Bertolaccini 1986
	Cagiotti 1985; Bertolaccini 1986
	Cagiotti 1985; Bertolaccini 1986
	Cagiotti 1985; Bertolaccini 1986
MZUR	
	Binaghi 1964
MZUR	
MZUR	
MZUR	
CLM	
CF	
CLM	
CF	
CF	
CF	
CF	
CLE	
CMI	
CMI	
CMI	
CMI	
CCP	
MZUR	
CF	
MZUR	
CLM	
CLM	
CLM	
MZUR	
CMZ	
CMZ	
CF	
CF	
CMZ	
CMZ	
CMZ	
CMZ	
CMZ	
CMZ	
CMZ	
CMZ	
CMZ	
CMZ	
MZUR	
CF	
MZUR	
CPE	
CF	
CVI	
CLM	

CLM	
CGA	
CLM	
CLE	
	Cagiotti 1985; Bertolaccini 1986
	Cagiotti 1985; Bertolaccini 1986
	Cagiotti 1985; Bertolaccini 1986
	Cagiotti 1985; Bertolaccini 1986
CMI	
	Canzoneri 1959
	De Salvo 1999
CA	
CLE	
	Leo 1982a
	Cagiotti 1985; Bertolaccini 1986
	Cagiotti 1985; Bertolaccini 1986
	Cagiotti 1985; Bertolaccini 1986
	Cagiotti 1985; Bertolaccini 1986
	Cagiotti 1985; Bertolaccini 1986
	Cagiotti 1985; Bertolaccini 1986
	Cagiotti 1985; Bertolaccini 1986
	Cagiotti 1985; Bertolaccini 1986
	Cagiotti 1985; Bertolaccini 1986
	Cagiotti 1985; Bertolaccini 1986
	Cagiotti 1985; Bertolaccini 1986
	Cagiotti 1985; Bertolaccini 1986
	Cagiotti 1985; Bertolaccini 1986
	Cagiotti 1985; Bertolaccini 1986
	Cagiotti 1985; Bertolaccini 1986
	Cagiotti 1985; Bertolaccini 1986
CMI	
MZUR	
MZUR	
MZUR	
MZUR	
MZUR	
MZUR	
CLE	
CLE	
CLE	
	De Salvo 1999
	De Salvo 1999
	De Salvo 1999
	De Salvo 1999
	De Salvo 1999
	De Salvo 1999
	De Salvo 1999
	De Salvo 1999
	De Salvo 1999
CLM	
CF	

UTM cell
TG84
TG84
TG72
UG09
UG09
UG18
UG18
UG18
UG18
UG18
UG18
UG18
UG19
UG19
UG19
UG19
UG19
UG19
UG19
UG19
UG19
UG19
UG19
UG19
UG09
UG09
UG09
UG09
UG19
UG19
UG19
UG19
UG19
UG18
UG18
UG18
UG18
UG18
UG18
UG18
UG18
UG18
UG18
UG18
UG18
UG19
UG19
TG82
UG02
TG84
UG30
UG30

UG30
UG40
UG40
UG30
UG30
UG30
UG30
UF78
UF78
UF78
UF78
UF81
UF81
UF81
UF81
UF81
UF81
UF68
UF68
UG63
UF38
UF38
UF38
UF38
UF38
TG54
TG54
TG54
TG54
TG54
TG54
TG54
UF38
TG69
TG69
TG69
TH60
TH60
TH60
TH60
TG84
UG62
UG62
UG02
UG12
UG12
UG12
UG12
UG12
UG12
UG12
UG12
UG12
UG12

UG12
UG12
UG12
UG12
UG12
UG12
UG12
TG81
UG12
UG12
UF37
UG57
TG69
UF81
UF81
QM46
QM47
QM47
QM47
QM47
QM47
UF81
UF81
QM46
QM47
QM47
QM37
QM37
TG83
TG84
TG84
TG84
TG84
TG84
TG84
TG94
TG94
TG69
TG85
TG84
QM47
TG72
QM47
TG94
TG96
UG11
UG21
UG12
UG30
QM47
UF38
TG84
UG02
UG02

TG63
TG63
TG63
TG63
TG63
TG63
UG26
TG66
TG74
TG72
TG72
TG72
TG72
TG82
UH10
UH10
UG40
TG81
VF19
UG54
TG73
UG40
TG66
UG12
UG12
QM46
TG66
TG76
TG66
TG91
TG91
TG72
TG82
TG82
TG82
TG82
TG81
TG82
TG82
TG81
TG81
TG69
TG69
TG69
TG69
TG69
TG69
TG69
TG69
TG69
TG69
TG69
TG69
TG69

TG69
TG69
TG69
TG69
TG69
TG69
TG69
TG69
TG69
TG69
TG69
TG69
TG69
TG69
TG69
TG69
TG69
TG69
TG69
TG69
TG69
TG69
TG69
TG69
TG69
TG69
TG69
TG69
TG69
TG69
TG69
TG69
TG69
TG69
TG69
TG69
TG69
TG69
TG69
TG69
TG69
TG69
TG69
TG69
TG69
TG69
TG69
TG69
TG69
TG69
TG69
TG69
TG69
TG69

TG69
TG69
TG69
TG69
TG69
TG69
TG69
TG69
TG69
TG69
TG72
TG90
UG02
UG12
QN34
TG66
UG12
UG02
UG12
UG12
UG12
UG12
UG12
UG12
UG12
UG12
UG12
UG12
UG12
UG12
UG12
UG02
UG02
UG02
UG02
UG02
UG02
UG02
UG02
UG02
UG02
UG02
UG12
UG12
UG12
TG66
TG84
UG07
UG07
TG72
TG84
TG83
TG94
TG95

TG67
TG82
TG72
UG15
TG86
UG02
UG12
TG96
TG86
TG69
TG55
UF38
UF38
UF38
TG97
UG50
QN34
QN34
QN34
QN34
QN34
QN34
QN34
QN34
QN43
QN43
QN43
QN43
QN43
QN43
QN43
QN43
QN43
QN43
QN43
UG63
UG02
TG67
TG66
TG66
TG84
TG67
TG66
TG67
TG67
TG67
TG67
UG10
TH60
TG86
TG84
UG12
TG74
UF28

UG17
TG94
UF38
TG84
TG94
UG26
TG93
TG94
UG14
TG92
TG94
TG94
TG94
TG94
TG93
TG93
TG94
TG94
TG94
UF57
TG93
TG94
TG94
TG94
QM36
TG94
TG84
UG30
UG30
TG93
TG94
TG93
TG94
TG94
TG93
TG94
TG94
TG94
UG04
UG35
TG94
TG72
TG94
TG94
TG62
TG93
TG93
TG94
UF09
TG55
QN42
TH62
QN42
TH62

TG94
TG94
TG94
UG14
TG94
TG94
TG94
UG14
TG94
TG94
TG84
TG94
TG94
TG94
TG93
QM47
TH62
TG93
TG94
TG94
TG94
TG94
TG94
UG02
TG72
UF09
TG94
TG94
TG94
TG94
TG94
TG94
TG94
UG12
TG84
TG93
TG94
TG93
TG94
TG94
TG93
TG88
TG94
TG83
TG94
TG94
TG94
UF57
TG94
TG94
TG83
TG94
TG94
TG94

TG93
TG94
TG94
TG93
TG94
TG94
TG94
TG94
TG94
TG94
TG94
TG94
TG94
TG94
TG94
TG94
TG94
TG94
TG94
TG94
TG94
UG14
TG84
TG94
TG94
TG94
TG94
TG94
TG94
TG94
TG94
TG94
TG94
TG94
TG84
TG84
TG94
TG94
TG93
UF86
UG02
UF89
UF09
TG72
UF37
UF37
UF37
UF37
UF37
UF37
UF37
UF37
UF37
UF37

TG82
TG82
TG82
TG82
TG82
TG82
TG82
TG82
TG82
TG82
UF37
UF37
TG82
TG82
UF18
TG73
TG81
TG82
TG63
UF36
UF37
QM27
UF36
UF28
TG72
TG72
TG82
TG82
TG82
TG82
TG82
TG82
TG82
TG82
TG82
TG82
TG82
TG82
TG82
TG82
TG82
TG82
TG82
TG82
TG82
TG82
TG82
TG82
TG82
UF37
TG72
TG72
TG72

TG55
TG73
UF68
UF68
UF68
UF68
UF36
UF36
UF36
UF36
UF37
UF37
UG30
UG21
UG40
UG30
UG49
UF37
UG01
UG12
UG12
UG12
UG12
UF36
UF36
UF36
UF38
UF36
UF36
UF36
UF36
UF36
UF36
UF36
UF36
UF36
TG73
TG72
TG93
TG93
TG72
TG72
TG72
TG93
TG54
TG54
TG54
UG04
UG02
UG02
UG02
UG02
UG02
UG02

UG02
UG02
UG02
UG02
QM45
QM37
QM37
QM37
QM37
QM37
QM37
QM47
QM37
QM37
QM37
TG84
TG84
TG94
TG94
TG94
TG94
TG94
QM35
TG94
UG02
UG02
TG93
UG02
TG93
TG93
TG73
TG72
TG93
TG94
TG93
TG63
TG63
TG63
TG63
TG63
TG63
TG63
TG74
TG82
TG67
TG67
TG94
TG94
TG94
TG85
TG84
TG84
TG75
TG75

TG75
TG75
TG82
TG82
TG82
TG82
TG82
TG82
TG82
TG82
TG82
TG82
TG82
TG82
TG82
TG82
TG82
TG82
TG82
TG82
TG82
TG82
TG82
TG82
TG82
TG82
TG82
QM37
UG02
UG12
TG94
TG94
TG94
TG94
TG55
TG72
TG84
TG68
TG66
TG66
TG58
TG94
TG93
UG63
UG64
UH62
UH62
UH62
UH62
UH61
UG64
UG64
UG67
UG35

UG63
UG63
UH62
UG44
UG63
UG63
UG63
UG63
UG92
UH62
UH61
UH62
UH61
UF87
UG44
UG64
UG43
UG43
UG44
UG64
UG63
UG44
UG44
UG44
UG63
UG44
UG44
UG67
UG63
UG44
UG63
UG63
UG63
UG21
UG40
UG63
UG44
UG63
UF99
UG63
UG43
UG44
UG44
UG49
TG69
UG21
UG44
UG26
UG30
UG24
UH30
UH30
UH30
TG69

UG63
UG63
UG35
TG94
UG35
UH30
UH30
UH31
UG49
TG94
TG94
TG94
TG82
TG84
TG74
UG14
TG94
TG94
TG94
TH51
TG94
TH60
TG59
TG96
UF36
TG84
TG94
TG85
UG02
UG02
UG02
TG94
UG14
UG30
TG94
TG94
TG94
TG79
TG68
TG93
TG94
TG72
TG94
TG66
TG81
TG81
TG93
TG84
TG94
TG94
TG96
UG10
UG20
UG10

UG10
UG10
UG10
UG20
UG10
UG20
UG10
UG10
UG20
UG10
UG10
UG10
TG72
QM37
UG43
TG82
TG85
TG84
TG84
TG84
TG94
TG94
TG94
UF37
TG74
TG95
QM29
QM29
QM29
QM29
QM29
QM29
QM29
TG82
TG94
TG94
TG94
UG10
UG52
UG12
TG75
TG75
TG75
TG75
TG75
TG75
TG75
TG75
TG75
TG75
TG75
TG75
TG75
TG75

TG75
TG82
TG82
TG73
TG76
TG58
TG94
UG07
TG94
TG94
TG94
TG94
TG94
TG94
TG84
TG92
TG67
TG94
TG94
TG94
TG94
TG66
TG94
TG96
TG94
TG94
TG94
TG94
TG94
TG94
TG94
TG93
TG94
TG93
TG93
TG94
UG10
TG94
TG94
UG02
UG02
TG94
TG94
UG10
TG94
TG76
UF36
TG72
TG84
TG72
TG93
TG66
TG72
TG95

TG72
UG13
TG93
TG82
TG67
TG94
TG86
TG64
TG72
TG82
TG94
TG94
UG11
TG94
TG94
TG94
UG14
UG02
TG95
TG94
TG94
TG72
QM39
TG94
TG94
TG92
TG94
TG66
TG69
TG94
TG94
TG75
TG94
TG94
TG83
TG92
TG94
TG94
TG94
TG79
TG94
TG94
TG94
TG94
TG94
TG94
TG94
TG94
TG94
TG94
UG02
TG69
TG94
UG10

UG01
TG84
TG94
TG94
UF67
UG21
TG94
TG94
TG94
TG94
TG94
TG84
TG94
TG94
TG94
UG14
UG14
UF57
UG02
TG93
TG94
TG94
TG94
TG94
TG93
TG67
TG67
TG69
TG67
UG41
UG41
UG63
UG40
UG40
TG66
TG69
TG81
TG81
TG81
TG67
UG44
TG67
TG67
UG45
TG67
TG67
UG63
TG72
TG84
TG72
TG72
TG82
TG85
TG72

TG82
UF28
UF28
UF28
UF28
UF28
UF28
UF28
UF28
UF28
UF28
UF36
UF36
UF36
UF36
UG04
TG82
TG83
TG93
UF28
TG82
TG73
UF28
UF28
TG54
TG63
TG63
TG63
TG83
QM37
QM46
TG86
TG73
TG72
TG72
TG73
UG21
UG02
UF28
UF28
UG21
UF18
TG82
UF36
UF36
TG72
TG96
TG63
TG74
TG90
TG72
TG72
TG82
UF36

UF36
TG82
TG82
TG91
TG82
TG82
TG82
TG82
TG82
TG82
TG82
TG82
TG74
TG72
UG02
TG73
TG73
TG84
TG92
TG55
UF36
UF36
UF36
UF36
UF37
UF37
UF37
UF37
TG93
UF36
UF36
UF57
TG93
TG93
TG83
TG93
TG55
TG72
TG93
TG55
TG73
TG84
TG94
UG12
TG72
TG82
TG93
TG82
TG99
TG94
TG72
UG25
TG94
TG94

TG84
UG44
UG64
UG64
UG64
UG45
TG94
TG84
UG18
UG19
UG19
UG19
UG19
UG19
UG30
TG72
TG73
TG73
TG73
UF18
TG73
TG73
UF78
UF78
UF78
UF78
UF81
UF81
UF81
QM47
UF68
UF68
UF68
UF68
UF68
UF36
TH60
TH60
TH60
UF36
TG93
UG02
UG02
UG12
UG12
UG12
UG12
UG02
UG02
UG02
UG12
UG12
UG12
UG12

UG12
UG12
UG12
UG12
UG12
UG12
UG12
UG12
UG12
UG12
UG12
UG12
TG81
UG12
UG12
TG82
TG82
TG82
TG82
QM46
QM47
QM47
QM45
QM47
QM47
QM37
QM37
TG83
TG84
TG84
TG84
TG84
TG84
TG93
TG94
TG94
TG79
TG93
TG82
UF37
TG81
TG72
UG44
UG30
UG12
UG30
UG10
UG30
UG12
UG26
TG84
TG72
TG72
TG63

TG63
TG63
TG63
TG74
TG72
TG72
TG72
TG72
TG72
TG72
TG82
UF38
TG66
TG66
TG66
TG69
UG15
TG82
TG82
TG81
TG81
TG82
TG82
TG82
TG82
TG82
TG93
TG82
TG81
TG81
TG81
TG69
TG69
TG69
TG69
TG69
TG69
TG69
TG69
TG69
TG69
TG69
TG69
TG69
TG69
TG69
TG69
TG69
TG69
TG69
TG69
TG69
TG69
TG69

TG69
UG40
UG12
UG12
UG12
UG12
UG12
UG12
UG02
UG02
UG02
UG02
UG02
UG12
UG12
UG12
TG84
TG84
UG14
UF37
UF37
UF36
UF36
UF36
UF36
UF36
UF37
UF37
UF37
UF37
UF37
UF36
UF36
UF36
UF36
UF36
UF36
UF36
UF36
UF36
UF36
QN34
QN34
QN34
QN34
QN34
QN43
UF46
TG73
UH41
UF28
UF28
UG02
UF36

TG72
TG72
TG84
TG66
TG95
UG12
TG73
TG73
TG82
TG82
TG82
TG55
UF28
UF37
TG94
TG72
TG82
TG72
UG64
TG91
TG82
TG82
TG81
TG73
UG40
UG63
UG63
UG09
TG66
TG95
TG55
TG67
UG44
TG67
UG63
UG63
TG67
TG82
TG83
QM46
TG73
TG72
TG72
TG94
QM46
QM47
TG83
TG55
TG94
TG82
TG82
TG74
TG82
TG82

TG82
TG82
TG82
TG73
TG83
TG94
TG73
TG72
TG94
TG83
TG94
QM35
UG68
UG63
UG48
UG29
TG69
TG85
QM38
TH51
TH60
TG66
TG73
TG73
TG75
TG54
TG54
TG54
TG54
TG64
TG64
TG78
QM47
TG84
TG55
TG83
QM29
TG66
TG66
TG73
TG66
TG66
TG69
TG55
TG55
TG55
TG69
TG69
TG69
TG69
QN34
QN34
QN34
TG66

TG79
UF47
UF67
UG12
UG57
TG63
TG82
TG81
TG81
TG73
TG63
TG93
TG93
TG63
UF81
TG91
TG82
TG82
TG82
TG82
TG94
TG94
TG95
TG95
TG81
TG82
TG82
UG07
TG94
TG72
TG94
UG30
UG63
TG73
UG04
TG67
TG57
TG82
TG81
TG81
TG81
TG81
TG81
TG81
TG81
TG81
TG82
TG67
TG67
UF28
TG94
TG94
TG94
TG84

TG84
QM46
QM46
UG21
UG35
TG72
TG55
TG82
TG94
UF36
TG93
UG30
TG74
UG04
TG72
UF47
UF37
QM45
QM18
UF28
UF28
UF28
UF18
UF28
UF28
TG72
UF18
TF99
TG99
UF37
UF37
UF37
UF37
UF37
UF37
UF37
UF37
UF37
TG82
TG82
TG82
TG82
TG82
TG82
TG82
TG82
TG82
TG82
UF37
UF37
TG82
TG82
TG90
TF99

TG81
TG82
TG72
TG63
TG63
TG72
TG63
TG63
TG72
UF37
UF37
UF37
UF28
UF28
UF18
UG37
UF37
UG37
UF28
TG81
UF36
TG72
TG72
TG72
UF67
UF37
UF37
TG82
TG82
TG82
TG82
TG82
TG82
TG82
TG82
TG82
TG82
TG82
TG82
TG82
TG82
TG82
TG82
TG72
TG72
TG82
TG82
TG82
TG82
TG82
TG82
TG73
UF67
TG54

TG54
TG72
TG73
UF18
TG72
TG72
TG72
TG72
QM18
UF36
UF36
TG63
TG72
TG63
TG72
TG72
TG72
TG72
TG72
TG63
TG72
TG72
TG72
UG04
TG72
TG72
TG72
TG72
UF36
TG72
TG72
UG30
TG63
TG94
TG84
TG93
TG83
TG82
UG05
TG93
TG94
TG82
TG94
UG35
TG93
TG93
UG04
TG82
UG02
TG72
TG94
TG84
UG04
TG74

TG67
UF66
TG96
TG82
UF18
UG12
UG12
UG12
UG12
UG12
TG94
UG07
TG93
TG94
TG72
UG02
TG84
TG94
TG64
TG82
TG84
TG93
TG94
TG94
TG94
TG94
TG94
TG72
TG83
TG94
TG95
QM35
QM35
TG94
UF57
TG94
TG94
TG94
TG94
TG94
TG94
TG94
TG94
TG93
UG22
QM35
QM35
UF57
TG84
TG84
TG93
TG94
TG72
TG94

TG72
TG82
TG72
TF99
UF37
UF37
UF37
UF37
UF37
UF37
UF37
UF37
UF37
UF37
TG82
TG82
TG82
TG82
TG82
TG82
TG82
TG82
TG82
TG82
UF37
UF37
TG82
TG82
TG81
TG82
TG63
TG72
TG63
UF37
TG81
TG82
TG82
TG82
TG82
TG82
TG82
TG82
TG82
TG82
TG82
TG82
TG82
TG82
TG82
TG82
TG82
TG82
TG82
TG82

TG82
TG82
TG82
TG82
TG82
TG72
TG72
TG72
TG81
TG81
TG63
UF46
UF09
TG63
TG94
UG02
TG72
UG18
UG18
UG19
UG19
UG19
UG19
UG18
UG18
UG19
TG82
UG02
TG66
UG30
UG91
UF67
TG94
UG31
UF38
TG69
TH70
TH60
TG84
UG62
UG52
TG84
UG12
UG12
UG12
UG12
UG12
UG12
UG12
UG12
UG12
UG12
UG12
TG81

TG81
UG12
UG12
UG63
UG12
TG82
UG63
TG82
TG82
UF81
QM37
TG78
TG79
TG72
QM47
TG72
TG96
TG58
UG30
UG30
UG54
UG40
UG63
TG94
QM47
UF36
UG12
TG72
UG35
UG35
TG93
TG66
TG66
TG82
TG82
TG82
UH10
UG43
UG44
UG44
UG40
UG02
TG82
UG63
UG12
UG12
TG76
TG91
TG69
TG91
QM46
UG12
TG72
TG82

TG82
TG81
TG82
TG82
TG81
TG82
TG82
TG82
TG82
TG82
TG82
TG81
TG82
TG69
TG69
TG69
TG69
TG69
TG69
TG69
TG69
TG69
TG72
UG12
QN34
TG58
TG65
UG12
UG12
UG12
TG86
TG84
TG84
TG56
UG30
TG69
UG35
TG73
TG69
TG67
TG67
TG82
TG67
UG10
TH60
TG85
UF18
UG35
TG94
QM37
UG26
UG10
TG73
TG73

TG73
TG73
TG73
TG73
UG50
UG21
UG11
UG10
UG10
UG30
UG40
TG73
UG11
UG14
UG14
TG73
TG73
TG73
UF49
UG30
TG82
TG82
TG81
TG81
TG81
TG82
TG82
TG91
TG91
TG91
QM47
UG40
UG40
TG57
UG30
UG30
UG21
QM46
TG82
TG82
TG82
UF19
UF38
UF37
TG82
TG82
TG82
TG82
TG82
TG82
TG82
TG82
TG82
TG82

TG82
TG81
TG81
TG82
TG82
TG82
TG82
TG81
TG55
TG55
TG55
UG10
TG82
UG10
UF38
TG82
TG82
UF38
TG82
TG72
TG72
TG72
TG72
TG69
TG72
UF86
UF86
TG94
UF89
TG55
UF76
TG94
TG62
TG63
TG72
TG72
TG72
TG72
TG72
TG72
TG82
TG72
TG72
UF18
TG72
TG72
TG72
TG72
TG82
TG82
TG81
TG82
TG72
TG72

UF09
UF09
TG72
UF18
UF36
UF36
UF36
TG94
TG84
TG72
TG72
UG19
QM47
UG35
TG96
UF38
TG84
TG77
TG82
UG12
TG73
UF37
TG66
TG82
TG82
TG76
TG64
TG63
TG84
TG94
TG94
TG94
UG26
TG84
TG84
UG20
UG30
TG84
UG41
TG63
UG33
UG02
UG26
TG72
UG02
TG96
TG73
TG84
UG12
TG82
TG82
TG82
TG82
TG82

TG82
TG81
TG82
TG82
TG82
TG82
TG82
TG82
TG82
TG81
TG81
TG81
TG82
QN34
TG87
UG07
TG82
TG67
TG82
TG84
UG02
TG84
TG94
TG75
TG84
TG84
TG85
UG09
TG72
TG94
UG12
TG94
TG54
QM18
TG54
TG64
UF86
UF87
TG84
UG11
UG20
UG20
UG20
UG20
UG20
UG20
UG10
UG30
UG30
TG66
QM46
UG12
UG02
TG93

TG93
UG10
TG72
UF86
TG72
UG02
TG83
TG82
TG82
TG82
TG66
TG87
TG82
TG94
TG85
TG94
TG84
TG73
TG73
TG93
TG84
TG84
TG84
TG84
TG84
TG94
TG72
TG94
TG94
UG44
TG94
TG84
TG84
TG93
TG72
TG94
TG82
UF09
TG82
TG82
TG82
TG82
TG81
TG82
TG73
TG91
UG41
UG41
TG66
TG67
TG67
TG67
TG67
TG67

TG94
UG12
QM46
TG93
TG93
TG56
TG82
TG82
TG82
TG82
TG82
TG82
TG55
TG94
TG94
UG14
UG48
TG72
TG83
TG94
UG30
TG72
UG04
TG93
UF87
UG02
UG02
UG02
UG02
UG02
TG93
TG82
UG43
UG40
TG94
TG94
UG11
UG11
UG20
UG20
UG20
UG10
UG10
UG20
UG20
UG20
UG20
UG20
UG20
UG10
UG10
UF69
UG10
UG20

UG43
TG73
TG93
TG93
UG02
TG84
TG93
UG02
TG93
TG93
TF99
TG94
UG10
UG01
TG94
TG84
TG84
TG75
TG75
TG75
TG75
TG75
TG75
TG83
TG75
UG44
UG10
UG12
UG12
UG12
TG94
UF09
TG99
TG94
TG94
TG94
TG86
TG94
TG94
TG94
UG14
UG02
TG72
TG94
UG04
TG72
TG94
UG14
TG94
TG94
TG93
TG83
TG93
TG94

UG12
UG11
TG75
UF47
UF47
UF47
UF46
TG95
UG04
TG84
UG12
UG02
UG02
TG90
TG82
TG94
TG66
TG67
TG82
TG94
TG76
TG94
TG69
UG19
TG92
TG94
TF99
UF36
UF36
UF36
UF36
UF36
TG84
TG84
UG52
UG49
TG93
UG02
UG02
UG02
UG02
UG02
UG02
UG02
UG02
UG02
TF99
UF28
UF86
TG63
TG63
TG94
TG94
TG94

TG94
QM37
UG12
UG10
UG26
TG74
TG83
TG85
UG12
UG35
TG93
TG93
TG63
TG74
TG75
TG94
TG83
UF37
TG72
TG72
UG01
UF86
UG15
QM29
UG04
UG04
TG75
TG75
TG75
TG75
TG75
TG75
TG75
TG75
TG75
TG75
TG75
TG75
TG75
TG75
TG82
TG81
TG82
TG82
TG82
UG15
UG25
TG99
TG72
TG73
UG02
UG02
UG12
UG12

UG12
UG12
UG12
UG12
TG94
TG67
TG94
TG94
UF57
UF36
UF36
UF36
UF36
QM47
UG63
TG94
UG00
QM19
QM19
TG94
TG94
TG94
TG64
UG43
TG94
TG94
TG86
TG93
TG93
TG93
TG93
TG94
TG94
TG94
TG94
TG94
TG94
UG14
TG73
TG82
UG04
TG94
TG94
TG94
TG83
TG94
TG82
TG93
UH30
UG12
UH30
TG94
TG82
TG94

QM46
QM46
TG67
TG67
TG67
UF77
TG74
UG04
UF28
TG81
UF37
UF37
UF37
UF37
UF37
UF37
UF37
UF37
UF37
UF37
TG82
TG82
TG82
TG82
TG82
TG82
TG82
UF37
UF37
TG82
TG82
TG82
TG73
TG63
UF36
TG63
QM27
UG37
QM45
TG73
TG54
TG63
QM27
TG90
UF37
UF89
UF76
TG82
TG82
TG82
TG82
TG82
TG64
TG54

UF37
TF99
TG72
UF09
TG72
QM36
TG54
TG54
TG72
TG72
UF57
UF86
UF86
UF37
UF37
UF37
UF37
UF37
UF37
UF37
UF37
TG82
TG82
TG82
TG82
TG82
TG82
TG82
TG82
UF37
UF37
TG82
TG82
TG73
UF37
UF37
UF37
UF18
TG63
TG81
TG63
UF37
UF37
TG63
UF37
UF37
TG54
TG63
TG72
UF28
UF37
UG37
TG82
TG82

UF09
TG73
TG63
UF28
UF37
TG72
TG72
TG63
TG63
UF37
UF37
UF28
TH51
UG14
TG66
UG14
UG16
TG69
TG68
UG14
TG66
UF47
UF47
UF47
TG72
UF37
TG74
UF28
UF28
UF28
UF28
UF28
UF28
UF28
TG81
TG90
TG72
UF28
TG72
UF36
UF36
UF36
UF09
UF37
UF37
UF37
UF37
UF37
UF37
UF37
UF37
UF37
UF37
TG81

TG81
TG81
TG81
TG81
TG81
TG81
TG81
UF37
UF37
TG72
TG81
TF99
TG63
TG63
TG81
TG81
TG81
UF09
TG72
UF28
TG63
TG63
TG72
TG72
UF37
UF37
UF37
TG72
TG72
TG72
TG81
UF28
UG37
TG99
UF28
UF28
UF28
UF28
UF37
TG72
TG72
TG74
UF36
UF28
TG72
TG82
TG82
TG72
TG74
TG74
TG74
UG37
UG37
UG37

TG72
TG72
TG72
TG72
UF28
TG72
TG72
TG81
TG81
TG81
TG81
TG81
TG81
TG81
TG81
TG81
TG81
TG81
TG81
TG81
TG81
TG81
TG81
TG73
TG73
TG73
TG73
QM27
TG72
TG72
TG82
TG82
TG82
TG82
TG81
TG81
UF18
UF38
TG73
UF67
TG99
TG72
TG72
TG73
UF18
TG72
TG72
TG72
TG72
TG72
UF36
UF36
UF36
UF36

UF36
UF36
TG72
TG63
TG72
TG72
TG72
TG73
QM27
TG72
TG73
TG72
TG72
TG94
TG72
TG72
TG72
TG72
TG72
TG72
TG94
TG72
TG72
TG72
UF36
TG94
UF37
UF37
TG72
UF18
UF18
TG72
TG72
TG72
TG73
TG95
TG81
TG73
TG81
TG73
UF26
UF38
UF38
UG00
UG10
UF18
TG82
TG82
TG82
QM46
TG94
TG55
TG73
UG63

TG82
TG81
UF28
UF18
UF18
UF37
TF99
UF37
UF37
UF37
UF37
UF37
UF37
TG82
TG82
TG82
TG82
TG82
TG82
UF37
TG82
TG82
TG63
UF37
UF37
TG73
TG73
TG73
TG72
TG72
TG82
TG82
TG82
TG82
TG82
TG82
TG82
TG82
TG82
TG82
UF37
TG84
TG72
TG84
TG56
UF28
UF28
TG63
TG93
TG94
TG94
TG94
TG84
TG55

TG55
TG69
TG69
TG94
UH52
QN43
TG94
TG94
TG94
TG74
QM38
UF09
TG94
TG82
TG81
TG72
TG94
UG04
TG94
TG84
TG82
TG63
TG74
TG74
UG04
TG94
TG94
TG82
TG82
TG94
TG73
TG73
TG72
TG94
TG94
TG94
TG73
TG84
TG84
TG84
TG84
TG81
TG81
TG82
TG82
TG82
TG81
TG81
TG81
TG82
TG82
TG93
TG93
TG81

TG81
TG81
TG73
TG94
TG72
TG54
TG94
TG94
TG94
TG94
UF57
TG72
TG94
TG94
UF66
TG93
TG94
TG94
TG94
TG94
TG94
TG72
TG73
TG94
TG82
TG94
TG84
TG84
TG84
TG84
UG14
TG93
TG94
TG94
TG94
TG94
UG14
TG72
TG94
UG04
UG04
TG72
TG72
TG72
TG94
TG94
TG94
UF36
TG72
TG93
TG83
TG94
TG94
TG94

TG94
TG69
TG69
TG69
UG18
UG18
UG18
UG19
UG19
TG69
UG18
UG19
UG19
UF87
UF87
UG21
UG30
TG69
QN22
UG12
UG12
UG12
UF86
UG63
UG12
UG32
UG63
UG56
UG12
UG02
UG02
UG02
UG02
UG02
TG69
TG69
TG67
UG12
UG02
UG12
UG02
TG67
TG67
UG02
UG45
TG82
TG84
TG94
TG84
TG94
TG84
TG91
UG20
UG20

UG20
UG20
UG20
UG20
UG20
UG20
UG20
TG93
QM35
TG82
TG85
TG84
TG93
TG93
TG93
TG83
TG93
TG81
TG82
TG94
UG02
UG04
TG94
UG04
TG93
TG83
TG66
UF37
TG54
UF36
UF36
UF36
UF36
UF36
TG63
TG63
UF37
UF37
QM27
TG82
TG72
TG72
TG72
TG72
TG72
TG82
TG82
TG82
TG82
TG81
UF36
UF37
UF37
UF37

UF37
UF36
UF36
UF36
TG55
UF37
TG84
TG95
TG82
TG85
TG73
TG72
TG94
TG82
TG93
TG73
TG91
TG93
TG93
TG94
TG94
TG94
TG94
TG72
TG73
TG95
TG94
TG91
UG10
UG10
UG10
UG10
UG20
UG10
UF18
UF18
UF18
TG84
TG93
TG93
UG02
TG93
QM35
TG93
TG93
UF37
UF37
TG72
TG72
TG72
TG72
TG72
TG72
TG90

TG83
TG82
TG93
TG84
TG93
TG84
TG84
TG84
TG82
TG81
TG82
TG82
TG82
TG82
TG81
TG82
UG07
UF28
TG73
TG73
UG10
TG82
TG83
TG94
TG94
UF57
TG86
TG94
TG72
TG93
TG94
TG94
TG94
TG94
TG84
TG82
QM28
TG93
TG72
TG94
TG84
UG14
TG84
TG72
TG94
UF47
UF47
UF47
UF46
UF47
UF47
TG72
UF37
QM27

UF28
UF28
TG72
TG72
TG73
TF99
TG99
UF57
TG72
TG72
TG72
TG54
UF36
UF36
TG72
TG94
TG82
TF99
UG12
TG63
TG81
TG63
TG81
TG82
TG73
TG63
TG63
TG81
TG63
UF37
TG72
TG72
UF28
TG99
UF37
UF28
UF28
UF18
UF09
UF46
UF28
UF28
TG82
TG94
UF37
UF28
TG63
TG82
TG72
UF67
UF37
UF37
TG72
TG72

TG72
TG72
TG72
TG72
TG72
TG72
TG72
UF67
UF09
UF46
TG82
TG73
TG73
TG73
TG73
TG73
TG72
TG72
TG82
TG72
TG72
TG72
TG72
TG75
TG82
TG82
TG82
TG82
TG82
TG81
TG81
TG82
TG81
TG73
UF67
UF37
TG72
TG73
TG73
TG73
TG72
TG72
TG72
TG72
TG72
TG72
TG72
TG72
TG81
TG72
QM18
UF46
UF36
UF36

UF36
UF36
UF36
TG94
TG72
UF66
TG72
TG73
TG96
TG82
TG72
TG72
TG94
TG94
TG72
TG72
TG72
TG72
TG72
TG72
TG72
TG72
TG72
TG72
TG72
TG72
TG72
UF36
UF37
UF37
TG72
TG72
UF36
UF37
TG74
QM28
TG84
UG01
TG94
UG22
UG12
TG84
TG73
TG85
UG10
UG10
UG10
UG10
UG10
UG10
UG10
UG10
UG10
UG10

UG10
UG10
UG10
TG93
TG63
TG74
TG74
TF99
TF99
UG04
UG00
TG83
TG94
TG66
TG84
TG94
TG93
UF09
TG94
UG13
TG94
TG93
TG72
TG72
TG83
TG83
UG00
UF09
TG99
TG67
TG67
TG93
TG94
TG94
TG94
TG94
TG93
TG94
TG94
UG02
TG72
TG82
TG94
TG75
UF37
UF37
UF37
UF37
UF37
UF37
UF37
UF37
UF37
UF37

UF37
TG82
TG82
TG82
TG82
UF37
UF37
TG62
TG63
UF37
QM27
QM28
TG54
TG81
TG54
TG84
TG84
TG95
TG72
TG72
TG81
TG82
TG91
TG91
TG82
TG82
TG72
UG02
TG67
TG67
TG67
UG10
TG82
TG82
TG81
TG81
TG81
TG81
TG81
TG82
TG82
TG82
TG81
TG81
TG81
TG81
TG82
TG82
TG72
TG55
TG72
TG72
TG67
TG67

UF09
UF28
UF38
TG81
UF37
TG82
TG82
UF37
TG72
UF37
UF76
TG82
UF37
TG81
TG82
UF28
UF37
UF37
UF37
UF37
UF37
TG82
TG82
TG82
TG82
TG82
TG82
TG82
UF37
UF37
TG82
TG82
TG81
UF37
UF36
UF36
UF37
UF37
TG73
TG73
TG54
TG73
TG82
TG82
TG82
TG82
TG82
TG82
TG82
TG82
TG82
TG73
TG81

Acronym	Collection
CA	Collezione Antonelli - Roma
CAN	Collezione Angelini - Francavilla Fontana, Bari (teste Leo)
CBE	Collezione Bertagni - Poglio, Prato (teste Leo)
CCO	Collezione Cotta - Torri di Barattano
CCP	Collezione Pacieri - Roma
CF	Collezione Fattorini - Roma
CGA	Collezione Garavglia - Museo Civico di Zoologia, Roma
CLE	Collezione Leo - Cagliari
CLI	Collezione Liberto - Roma (teste Leo)
CLM	Collezione Luigioni - Museo Civico di Zoologia, Roma
CMA	Collezione Masciello - Prato (teste Leo)
CME	Collezione Meloni - Cagliari (teste Leo)
CMI	Collezione Migliaccio - Roma
CMZ	Collezione Maltzeff - Roma
CNA	Collezione Nardi - Cisterna di Latina
CO	Collezione Osella - Università dell'Aquila
CPA	Collezione Pace - Roma
CPE	Collezione Pessolano - Roma
CPNA	Collezione Parco Nazionale d'Abruzzo - Pescasseroli
CRA	Collezione Rasetti - Museo Civico di Zoologia, Roma
CRC	Collezione Consorti - Prato (teste Leo)
CVI	Collezione Vita - Museo Civico di Zoologia, Roma
MSF	Collezione Museo di Zoologia Università di Firenze - Firenze (teste Leo)
MZUR	Collezione Museo di Zoologia Università di Roma Sapienza - Roma
UR3	Collezione Museo di Zoologia Università di Roma Tre - Roma

Author(s)	Year
Adorno, A.	2002
Alicata P., Caruso D., Costa G., Marcellino I.,	1979
Aliquò V.	1971
Aliquò V.	1990
Aliquò V.	1992
Aliquò V.	1993
Aliquò V.	1995a
Aliquò V.	1995b
Aliquò V., Aliquò A.	2000
Aliquò V., Leo P.	1996
Aliquò V., Leo P.	1999
Aliquò V., Massa B., Mignani R.	1973
Aliquò V., Rastelli, M., Rastelli, S., Soldati, F.	2006
Aliquò V., Soldati, F.	2010
Aliquò,V., Leo P., Lo Cascio P.	2006
Aloia A., Colombini I., Fallaci M., Chelazzi L.	1999
Andreetti A., Di Gaetano B., Di Marco C., Ose	1997
Angelini F.	1986
Angelini F.	1987
Angelini F.	1991
Angelini F.	1996
Angelini F.	1998
Angelini F.	1999
Angelini F., Montemurro F.	1986
Ardoin P.	1972
Ardoin P.	1973
Barabino, S., Gardini, G.	1968
Barberio C., Di Cello F., Chelazzi L., Colombir	2001
Baudi F.	1883
Baudi F.	1874a
Baudi F.	1874b
Baudi F.	1875a
Baudi F.	1875b
Baudi F.	1876a
Baudi F.	1876b
Baudi F.	1876c
Baudi F., Piccioli, F. & Cavanna G.	1882
Baviera C., Sparacio I.	2002
Baviera, C.	2006
Bertolaccini L.	1986
Bertolini S.	1876
Binaghi G.	1964
Binaghi G., Ghidini G. M.	1957
Bonometto L.	1976
Bonometto L., Canzoneri S.	1970
Bordoni A.	1973
Bordoni A.	1995
Bouyon H., Soldati, F., Soldati, L.	1999
Cagiotti D.	1985
Canzoneri S.	1959
Canzoneri S.	1962
Canzoneri S.	1964
Canzoneri S.	1966

Canzoneri S.	1976
Canzoneri S.	1977
Canzoneri S.	1981
Canzoneri S.	1984
Canzoneri S.	1960a
Canzoneri S.	1960b
Canzoneri S.	1960c
Canzoneri S.	1961a
Canzoneri S.	1961b
Canzoneri S.	1961c
Canzoneri S.	1961d
Canzoneri S.	1963a
Canzoneri S.	1963b
Canzoneri S.	1968a
Canzoneri S.	1968b
Canzoneri S.	1968c
Canzoneri S.	1969a
Canzoneri S.	1969b
Canzoneri S.	1970a
Canzoneri S.	1970b
Canzoneri S.	1972a
Canzoneri S.	1972c
Canzoneri S.	1972v
Canzoneri S., Vienna P.	1987
Carpaneto G. M.	1979
Carpaneto G. M., Fattorini S.	2001
Carpaneto G. M., Fattorini S.	2002
Carpaneto G. M., Fattorini S.	2003
Cassola F.	1965
Castelli V.D.	1986
Cecchi B., Bartolozzi L.	1997
Cecconi G.	1897
Cerruti, M.	1954
Chelazzi L., Colombini I.	1989
Chelazzi L., Colombini I., Bertin G., Cianfanell	1990
Chelazzi, L., Dematthaeis, E., Colombini, I., F	2005
Ciampi L.	1905
Colombini I., Chelazzi L., Fallaci M., Lucarelli	1991
Colombini I., Chelazzi L., Fallaci M., Palesse,	1994
Colombini I., Fallaci M., Chelazzi L.	2005
Contarini, E.	1992
Costa A.	1836
Costa A.	1843
Costa A.	1844
Crowson, R.A.	1958
Dahlgren G	1964
D'Amore Fracassi A.	1907
D'Antonio, Fimiani, P.	1988
De Salvo P.	1999
Deidun, A., Marrone, F., Sparacio, I., Arculeo	2011
Della Beffa G.	1909
Della Beffa G.	1911
Deville J. Sainte-Claire	1906
Di Stefano G., Fattorini S.	2000

Español Coll F.	1937
Español Coll F.	1949
Español Coll F.	1953
Faggioli D.	1955
Fallaci M., Aloia A., Colombini I., Palesse L.,	2002
Fallaci M., Colombini I., Chelazzi L.	1994
Fallaci M., Colombini I., Palesse L., Chelazzi I	1997
Fanfani A., Groppali R.	1979
Fattorini S, Carpaneto G.M.	2001a
Fattorini S, Maltzeff P.	2001b
Fattorini S.	2000
Fattorini S.	2002
Fattorini S.	2003
Fattorini S.	2005
Fattorini S.	2007
Fattorini S.	2008
Fattorini S.	2001a
Fattorini S.	2001b
Fattorini S.	2001c
Fattorini S.	2001d
Fattorini S.	2009a
Fattorini S.	2009b
Fattorini S.	2009c
Fattorini S.	2010a
Fattorini S.	2010b
Fattorini S.	2010c
Fattorini S.	2010d
Fattorini S.	2010e
Fattorini S.	2010f
Fattorini S.	2011a
Fattorini S.	2011b
Fattorini S.	2011c
Fattorini S., Manganaro A., Salvati L.	2001
Fattorini S., Distefano G.	2004
Fattorini S., Leo P.	2000
Fattorini S., Manganaro A., Piattella E., Salvat	2000
Fattorini S., Manganaro A., Ranazzi L., Cento	1999
Fattorini S., Vigna Taglianti A.	2002
Ferrer J.	2000
Ferrer J., Martínez Fernández J.C., Castro To	2007
Ferrer J., Whitehead P.F.	2002
Ferro G.	1996
Fiori A.	1903
Fiori A.	1906
Fiori G.	1947
Fiori G.	1977
Focarile A.	1969
Focarile A.	1970
Focarile A.	1972
Focarile A.	1973
Focarile A.	1975
Focarile A.	1987
Fontana, P.	1947
Freude H.	1995

Gardini G.	1969
Gardini G.	1973
Gardini G.	1977
Gardini G.	1979
Gardini G.	1981
Gardini G.	1986
Gardini G.	1997
Gardini G.	2010
Gardini G.	1968a
Gardini G.	1968b
Gardini G.	1968c
Gardini G.	1971a
Gardini G.	1971b
Gardini G.	1972a
Gardini G.	1972b
Gardini G.	1972c
Gardini G.	1972d
Gardini G.	1975a
Gardini G.	1975b
Gardini G.	1976a
Gardini G.	1976b
Gardini G.	1976c
Gardini G.	1976d
Gardini G.	1976e
Gatti E.	1991
Ghiliani V.	1887
Gobbi G.	1973
Gobbi G.	2002
Goggi G.	2004
Gridelli E.	1930
Gridelli E.	1939
Gridelli E.	1950
Gridelli E.	1956
Gridelli E.	1972
Gridelli E.	1949a
Gridelli E.	1949b
Gridelli E.	1960a
Gridelli E.	1960b
Grimm R.	1985
Grimm R.	1986
Gulli G.	1961
Heyrovsky L.	1969
Holdhaus C.	1923
Lanza B.	1956
Lanza B.	1958
Lanza B., Borri M.	1969
Leo P.	1980
Leo P.	1980
Leo P.	1980
Leo P.	1983
Leo P.	1985
Leo P.	1986
Leo P.	1990
Leo P.	1994

Leo P.	1998
Leo P.	2008
Leo P.	2009
Leo P.	1982a
Leo P.	1982b
Leo P.	1982c
Leo P.	1984a
Leo P.	1984b
Leo P.	1984c
Leo P.	1991a
Leo P.	1991b
Leo P., Fattorini, S.	2002
Leo P., Pisano P.	1978
Leo P., Pisano P.	1984
Leoni G.	1909
Leoni G.	1910
Leoni G.	1911
Lo Cascio P., Bartolozzi L., Cecchi B., Dappo	2000
Lucarelli E., Chelazzi L., Colombini I., Fallaci	1993
Luigioni P.	1892
Luigioni P.	1920
Luigioni P.	1923
Luigioni P.	1923
Luigioni P.	1924
Luigioni P.	1929
Luigioni P.	1931
Luigioni P.	1933
Luigioni P.	1921a
Luigioni P.	1921b
Luigioni P., Tirelli A.	1910
Magretti P.	1897
Magrone R., Papini G.	1966
Marcuzzi G	1952
Marcuzzi G	2001
Marcuzzi G.	1960
Marcuzzi G.	1962
Marcuzzi G.	1969
Marcuzzi G.	1970
Marcuzzi G.	1970
Marcuzzi G.	1970
Marcuzzi G.	1970
Marcuzzi G.	1982
Marcuzzi G.	1991
Marcuzzi G.	1994
Marcuzzi G.	1996
Marcuzzi G.	1997
Marcuzzi G.	1998
Marcuzzi G.	1985a
Marcuzzi G.	1985b
Marcuzzi G.	1985c
Marcuzzi G.	1985d
Marcuzzi G., Cravera C., Faccini E.	1980
Marcuzzi G., Turchetto Lafisca M.	1977
Marcuzzi G., Turchetto Lafisca M.	1981

Marcuzzi G., Turchetto M.	1982
Marzuttini G. B.	1955
Mattoli P.	1974
Mazzoldi P.	1982
Mignani R.	1971
Minelli A.	1968
Mingazzini P.	1885
Molinu A., Molinu A. V.	1998
Nardi G.	1994
Pardi L.	1958
Pescarolo R.	1997
Pilon N.	1999
Pisano P., Leo P.	1983
Pisano P., Leo P.	1985
Poggi R.	1976
Poggi R.	1977
Poggi R.	1983
Porta A.	1934
Porta A.	1949
Porta A.	1959
Ragusa E.	1872
Ragusa E.	1874
Ragusa E.	1897
Ragusa E.	1908
Ragusa E.	1921
Rallo G.	1969
Rallo G.	1969
Rallo G.	1970
Rallo G.	1974
Ratti E.	1978
Ratti E.	1986
Razzauti A.	1906
Razzauti A.	1917
Razzauti A.	1919
Reitter E.	1917
Sacchi C. F.	1960
Salerno G., Fanara S., Zunino M.	2000
Schatzmayr A.	1949
Schawaller W.	1987
Schawaller W.	1995
Schawaller W.	1998
Schawaller W., Grimm R.	1995
Schneider O.	1902
Schuster A.	1930
Scupola A.	1982
Scupola A.	2003
Scupola A.	2000a
Scupola A.	2000b
Scupola A.	2002a
Scupola A.	2002b
Soldati F., Leo P.	2005
Soldati F., Soldati L.	2000
Soldati F., Soldati L.	2001
Soldati F., Soldati L.	2002

Sparacio I.	2007
Stroscio S., Baviera C., Frati, F., Lo Paro, G.,	2011a
Stroscio S., Baviera C., Frati, F., Lo Paro, G.,	2011b
Tassi F.	1968
Tongiorgi P.	1969a
Vienna P.	1969b
Villa A., Villa G.	1844
Viñolas A., Cartagena M.C.	2003
Zangheri P.	1969
Zapparoli M.	1980
zur Strassen R.	1957

Title	Journal/Book Printer
Effetti del taglio del bosco e dell'erosione sulle zoocen	Bollettino dell'Accademia Gioenia di Scienze N
Comportamento, distribuzione spaziale e ritmi di attivit	Animalia
Brevi note sui Tenebrionidi di Levanzo (Is. Egadi) (Col.	Bollettino dell'Associazione romana di Entomo
Reperti. Pimelia bipunctata (Fabricius, 1781) ssp. papii	Bollettino dell'Associazione romana di Entomo
La collezione entomologica Alliata del Museo Regionale	Naturalista siciliano
Dati nuovi e riassuntivi sui Coleotteri Tenebrionidi delle	Naturalista siciliano
Coleoptera Tenebrionidae. In: Massa B., Arthropoda di L	Naturalista siciliano
Nuovi reperti di Tenebrionidae delle isole circumsiciliane	Naturalista siciliano
Terzo contributo alla revisione della collezione coleott	Naturalista siciliano
I Coleotteri Tenebrionidi delle Madonie (Sicilia)	Naturalista siciliano
I coleotteri tenebrionidi della regione iblea (Sicilia Su	Atti e Memorie dell'Ente Fauna Siciliana
Brevi note sulla fauna coleotterologica di un particolare	Bollettino della Società entomologica italiana
Coleotteri Tenebrionidi d'Italia. CD-ROM	Museo Civico di Storia Naturale di Carmagnol
Coleotteri Tenebrionidi di Sicilia Insecta: Coleoptera, T	Edizioni Danaus, Palermo
I Tenebrionidi dell'Arcipelago Eoliano: nuovi dati fauni	Naturalista Siciliano
Behavioural adaptations to zonal maintainance of five sp	Marine Biology
23. Coleoptera Tenebrionidae (Insecta). In: Osella B.G.,	Quaderni di Provincia oggi, 23/II. L'Aquila, Am
Coleotterofauna del Massiccio del Pollino (Basilicata-Ca	Entomologica, Bari
Coleotterofauna del Promontorio del Gargano (Coleopte	Atti del Museo civico di Storia naturale di Gro
Coleotterofauna dell'Altipiano della Sila (Calabria, Italia	Memorie della Società entomologica italiana
Coleotterofauna della Riserva Naturale WWF Lago di Pi	Alfagrafica Volonnino Lavello
Coleotterofauna reperita mediante trappola luminosa in	Quaderni della Stazione di Ecologia del Muse
Coleotterofauna della riserva naturale integrale "Isola d	Atti e Memorie dell'Ente Fauna Siciliana
Coleotterofauna del bosco di Policoro (Matera) (Coleopt	Lavori della Società italiana di Biogeografia
Deux nouvelles espèces du genre Isocerus Latr. (Col. T	Nouvelle Revue d'Entomologie
Contribution a l'étude des Tenebrionidae (Coleoptera)	Annales de la Société entomologique de Fran
Val Lerone (Arenzano - Prov. Genova) Relazione	Notiziario del Gruppo Entomologico Ligure
Molecular and physiological analysis of mesophilic aerob	Annals of Microbiology
Note entomologiche. I. Sugli Eteromeri di Sicilia	Naturalista Siciliano
Catalogo dei Tenebrioniti della fauna europea e circumn	Annali del Museo civico di Storia naturale di
Coleotteri Tenebrioniti delle collezioni italiane	Bullettino della Società entomologica italiana
Catalogo dei Tenebrioniti della fauna europea e circumn	Annali del Museo civico di Storia naturale di
Coleotteri Tenebrioniti delle collezioni italiane	Bullettino della Società entomologica italiana
Catalogo dei Tenebrioniti della fauna europea e circumn	Annali del Museo civico di Storia naturale di
Coleotteri Tenebrioniti delle collezioni italiane	Bullettino della Società entomologica italiana
Coleotteri Tenebrioniti delle collezioni italiane	Bullettino della Società entomologica italiana
. Ordo. Coleoptera. In: Al Vulture ed al Pollino - Parte II (	Bullettino della Società entomologica italiana
Coleotteri nuovi o poco noti di Sicilia. II.	Naturalista Siciliano
Coleotteri nuovi o poco noti di Sicilia III	Naturalista siciliano
Ricerche fauunistiche ed ecologiche sui coleotteri delle z	Tesi di laurea. Università di Roma "La Sapien
Escursioni entomologiche nella Calabria	Bullettino della Società entomologica italiana
Saggio sulla distribuzione della coleotterofauna sabulico	Annali del Museo civico di Storia naturale "G.
Sui Cylindronotus (Xanthomus) pallidus Curt. e pellucidu	Bollettino della Società entomologica italiana
Le Phaleria di Sardegna. Note sistematiche ed ecologic	Lavori della Società veneziana di Scienze nat
I Tenebrionidae delle spiagge e dune del litorale di Vene	Bollettino del Museo civico di Storia naturale
Coleotterofauna dei muschi in un ambiente palustre di P	Bollettino della Associazione Romana di Ento
I Coleotteri del Padule di Fucecchio (Coleotterofauna di	Centro di Ricerca, Documentazione e Promoz
Les Corticeus Piller & Mitterpacher, de France. C. bico	Bulletin de la Société entomologique de Fran
I Coleotteri Tenebrionidi di comunità psammofile delle du	Tesi di laurea. Università di Roma "La Sapien
Note sugli Xanthomus Muls. (Coleoptera, Tenebrionidae	Bollettino della Società entomologica italiana
Sugli ultimi sterniti introflessi della femmina in alcuni Bl	Bollettino della Società entomologica italiana
Breve nota sull'Oochrotus glaber Dem. (XIII Contributo a	Bollettino della Società entomologica italiana
I Tenebrionidae della laguna di Venezia (XIV Contributo	Bollettino del Museo civico di Storia naturale

I Tenebrionidae delle Isole Ponziane (Coleoptera)	Fragmenta entomologica
Contributo alla conoscenza dei Tenebrionidi appenninici	Bollettino del Museo civico di Storia naturale
Su alcuni interessanti Coleotteri Tenebrionidi dell'Italia	Lavori della Società veneziana di Scienze nat
Una nuova specie di Pedinus di Sicilia (XXXIV Contribut	Lavori della Società veneziana di Scienze nat
Diagnosi preliminare di una nuova entità del genere Pha	Bollettino della Società entomologica italiana
Note sistematiche e biogeografiche su alcuni Tenebrioni	Bollettino della Società entomologica italiana
Sulla Phaleria insulana Rey (V Contributo allo studio dei	Bollettino della Società entomologica italiana
Note sul genere Oochrotus Luc. VII contributo allo studi	Bollettino del Museo civico di Storia naturale
Sulla Phaleria bimaculata Lin. (IX Contributo allo studio	Bollettino della Società entomologica italiana
Sulla Phaleria provincialis Fauv. (X Contributo allo studi	Bollettino della Società entomologica italiana
Sulla Tentyria italica Sol. (VI Contributo allo studio dei T	Bollettino della Società entomologica italiana
Sulla Alphasida grossa Sol., con descrizione di una nuov	Bollettino della Società entomologica italiana
Sulla Pimelia bipunctata Fabr. (XI Contributo allo studio	Bollettino della Società entomologica italiana
Alcuni Coleotteri Tenebrionidi delle Isole Eolie (XVII con	Bollettino della Associazione Romana di Ento
Materiali per una monografia delle Phaleria del sottoge	Memorie della Società Entomologica Italiana
Primi dati sui Tenebrionidae di Favignana (XVI contribut	Bollettino del Museo civico di Storia naturale
Blaps sulcata ssp. mercatii nov.: diagnosi preliminare (X	Bollettino della Società entomologica italiana
Note sul sottogenere Pedinulus (Col. Tenebrionidae) (X	Bollettino della Associazione Romana di Ento
I Tenebrionidae delle Isole Egadi (XXII Contributo allo s	Memorie del Museo civico di Storia naturale d
Notulae tenebrionidologicae. XXI Contributo allo studio	Quaderni di documentazione. Congresso inter
Annotazioni su alcuni Nalassus (XXVI Contributo alla c	Bollettino della Società entomologica italiana
Nuovi dati sui Tenebrionidae di "piccole isole" italiane, c	Atti della Società italiana di Scienze naturali
Annotazioni sui Colpophorus italiani (XXVII contributo al	Bollettino del Museo civico di Storia naturale
I Tenebrionidae della Padania (Coleoptera Heteromera)	Bollettino del Museo civico di Storia naturale
Osservazioni sui Coleotteri Scarabaeoidea delle Isole P	Fragmenta Entomologica
Spatial and seasonal organisation of a darkling beetle	Italian Journal of Zoology
Flightlessness in psammophilous beetles inhabiting a M	Biogeographia
Seasonal occurrence and habitat distribution of tenebrio	Revue d'Ecologie (Terre et Vie)
Alcuni interessanti reperti della Tenuta di Castelporzian	Bollettino della Associazione Romana di Ento
Segnalazioni faunistiche italiane: 90. Tentyria laevigata	Bollettino della Società entomologica italiana
I Coleotteri xilofagi e subcorticicoli del Parco Naziona	Bollettino della Società entomologica italiana
Contributo alla fauna vallombrosana	Bullettino della Società entomologica italiana
Coleoptera. In: Zavattari, E. (ED.) Biogeografia dell'Iso	Rendiconti della Accademia Nazionale dei XL
Zonation and activity patterns of two species of the ge	Ethology Ecology and Evolution
Gli artropodi del tombolo antistante la Laguna di Buran	Redia
Abundance, zonation ane ecological indices of a coleop	Vie et Milieu
Specie di Coleotteri Romani non citati come tali od omes	Bollettino della Socità Zoologica Italiana
La coleotterofauna del tombolo antistante la Laguna di	Redia
Zonation and surface activity of some Tenebrionid beetl	Journal of Arid Environmentas
Micro-scale distribution of some arthropods inhabiting	Acta Oecologica
Eco-profili d'ambiente della coleotterofauna di Romagna	Bollettino del Museo Civico di Storia Naturale
Alcune notizie sull'entomologia dell'Isola d'Ischia	L'Iride
Esposizione sommaria delle osservazioni raccolte dura	Annali dell'Accademia degli Aspiranti Naturalis
Cenni intorno alle osservazioni zoologiche fatte durante	Annali dell'Accademia degli Aspiranti Naturalis
Some observations on a coleopterological visit to Centra	Entomologist's Monthly Magazine
Käfer aus Kalabrien	Atti del Museo civico di Storia naturale di Trie
Gen. Colpotus Muls. e Rey. Tavola sinottica e descrizio	Naturalista Siciliano
Approccio ad un inventario entomofaunistico dell'Isola di	Annuario dell'Istituto e Museo di Zoologia dell'
Struttura e dinamica di entomocenosi di dune naturali ec	Tesi di laurea. Università di Roma Tre (unpub
Morphological variability in and distributional data on	Bulletin of Insectology
Note coleotterologiche. I. Specie e varietà nuove per l'It	Rivista Coleotterologica Italina
I Coleotteri dell'agro torinese e loro raporti colla vegetaz	Annali della regia accademia di Agricoltura, T
Catalogue critique des Coléoptères de la Corse	Revue d'Entomologie, Caen
Corologia italiana di Erodius siculus ed osservazioni ec	Bollettino della Associazione Romana di Ento

Assaig Monogràfic del Subgènere Dendarus s. str. (Col.	Treballs del Museu de Ciències Naturals de B
Contribucion al conocimiento de los Crypticus del grupo	Eos
Los Colpotus del Mediterraneo occidental (Col. Tenebri	Eos
Campagna di ricerche dell'Istituto di Entomologia dell'Ur	Bollettino dell'Istituto di Entomologia dell'Univ
Population dynamics and life history of two Phlaeria spe	Acta Oecologica
An analysis of the Coleoptera living along a Tyrrhenia	Vie et Milieu
Spatial and temporal strategies in relation to environme	Journal of Arid Environmentas
La fauna di Montecristo - Arcipelago Toscano (Studi sull	Pubblicazioni dell'Istituto di Entomologia dell'
Tenebrionid density in Mediotyrrhenian coastal dunes: h	Fragmenta entomologica
I Coleotteri Tenebrionidi della Tenuta presidenziale di	Bollettino dell'Associazione Romana di Entom
Is the Kestrel Falco tinnunculus able to discriminate ag	Ornis Svecica
The Tenebrionidae (Coleoptera) of a Tyrrhenian coastal	Biogeographia
L'entomofauna: insetti. In: A. Filpa & B. Romano (eds) Pianificazione e reti ecologiche. Planeco -Plan	
I Coleotteri Tenebrionidi del Parco Nazionale del Circeo	Bollettino dell'Associazione Romana di Entom
The tenebrionid beetles of Mt Vesuvius: species assemblages and biogeographic kinetics on an active	
Ecology and conservation of tenebrionid beetles in Mediterranean coastal areas. In: S. Fattorini (Ed)	
I Colotteri Tenebrionidi (Coleoptera Tenebrionidae) delle isole circumsiciliane: diversità e livelli di	
Endemismi italiani. 44 Asida (Asida) luigionii Leoni, 190	Bollettino dell'Associazione Romana di Entom
Nuovi dati regionali e altre note di distribuzione su Tene	Bollettino della Società entomologica italiana
Temporal and spatial variations in darkling beetle preda	Biologia Bratislava
Both Recent and Pleistocene geography determines ani	Journal of Zoology
Faunal patterns in tenebrionids (Coleoptera: Tenebrion	European Journal of Entomology
On the General Dynamic Model of oceanic island bioge	Journal of Biogeography
Biogeographical kinetics on mainland and island volcan	Journal of Biogeography
Biotope prioritisation in the Central Apennines (Italy):	Biodiversity and Conservation
Effects of fire on tenebrionid communities of a Pinus pin	Biodiversity and Conservation
Insect extinction by urbanization: a long term study in	Biological Conservation
The influence of geographical and ecological factors on	Journal of Biogeography
Use of insect rarity for biotope prioritisation: the tenebri	Journal of Insect Conservation
Biogeography of tenebrionid beetles (Coleoptera: Teneb	European Journal of Entomology
Influence of island geography, age and landscape on sp	Journal of Biogeography
Insect rarity, extinction and conservation in urban Rome	Insect Conservation and Diversity
Insect identification in pellet analysis: implications for t	Buteo – Journal on Birds of Prey and Owls
Osservazioni biogeografiche ed eco-etologiche su Pimel	Bollettino dell'Associazione Romana di Entom
Darkling beetles from Mediterranean minor islands: new	Bollettino della Società Entomologica Italiana
Insect predation by the Little Owl, Athene noctua, in diffe	Rivista Italiana di Ornitologia
Role of the beetles in raptor diets from a Mediterranean	Fragmenta entomologica
Ecological and historical factors affecting carabid and	Biogeographia
Révision des espèces africaines et européennes apparte	Atti del Museo civico di Storia naturale, Triest
Aportación al conocimiento del género Akis Herbst, 179	Boletin de la Sociedad Entomológica Aragon
The genus Xanthomus Mulsant, 1854 (Coleoptera: Tene	Annales Zoologici
Reperti. Anemia sardoa Gené (Col. Tenebrionidae)	Bollettino della Associazione Romana di Ento
Studio critico dei Tenebrio Lin. italiani	Rivista coleotterologica italiana
Una breve escursione in Sicilia	Naturalista siciliano
Due nuovi carabidi e due tenebrionidi a regime dietetico	Bollettino della Società entomologica italiana
La cavità sottoelitrale dei Tenebrionidi apomorfi	Redia
Sintesi preliminare delle attuali conoscenze sui Coleotte	Memorie della Società entomologica italiana
Ricerche entomologiche nell'Arcipelago delle Eolie e nel	Bollettino della Società entomologica italiana
Ricerche entomologiche nell'Arcipelago delle Eolie e nel	Memorie della Società entomologica italiana
Cenni sugli aspetti zoogeografici della coleotterofauna o	Atti della Accademia delle Scienze di Torino
Alcuni interessanti coleotteri della Valle d'Aosta (Ricerc	RevueValdotaine d'Histore Naturelle, Aosta,
I Coleotteri del Ticino	Tipografia Poncioni SA - Losone
Contribuzione alla fauna coleotterologica ticinese	Bollettino della Società Ticinese di Scienze Na
Segnalazioni faunistiche italiane. 269. Hypophloeus fra	Bollettino della Società entomologica italiana

Note di Caccia. Asida (Polasida) jurinei Sol. E Asida (A	Notiziario del Gruppo Entomologico Ligure
Coleoptera Tenebrionidae	Notiziario del Gruppo Entomologico Ligure
Coleoptera Tenebrionidae	Notiziario del Gruppo Entomologico Ligure
Nuovi dati su Tenebrionidi (Col.) dell'Arcipelago Toscan	Bollettino del Museo civico di Storia naturale
Segnalazioni faunistiche italiane. 4. Nalassus convexus	Bollettino della Società entomologica italiana
Coleotteri Tenebrionidi raccolti in Sicilia e in Calabria d	Notiziario del Gruppo Entomologico Ligure
Coleoptera Alleculidae, Tenebrionidae. In: Zapparoli M.	Comune di Roma, Dip. X Area Risorsa Suolo
Boromorphu italicus n. sp. dell?Italia meridionale (Cole	Doriana. Supplemento agli Annali del Museo (
Note di Caccia. Dendarus tristis Cast. (Col. Tenebrionid	Notiziario del Gruppo Entomologico Ligure
Note di Caccia. Pimelia bipunctata Fabr. (Col. Tenebrior	Notiziario del Gruppo Entomologico Ligure
Reperti. Phaleria reveillierei Muls. & Rey. Stenosis angu	Bollettino dell'Associazione romana di Entomo
Coleoptera Tenebrionidae	Notiziario del Gruppo Entomologico Ligure
Nalassus dryadophilus Muls. E N. dermestoides Illig. (C	Notiziario del Gruppo Entomologico Ligure
Coleoptera Tenebrionidae	Notiziario del Gruppo Entomologico Ligure
Coleoptera Tenebrionidae	Notiziario del Gruppo Entomologico Ligure
Coleoptera Tenebrionidae	Notiziario del Gruppo Entomologico Ligure
Raccolte entomologiche nell'Isola di Capraia (Arc. Tosc	Bollettino della Società entomologica italiana
Brevi note e tabella per la fdeterminazione dei Blaps ligu	Notiziario del Gruppo Entomologico Ligure
Coleoptera Tenebrionidae	Notiziario del Gruppo Entomologico Ligure
Col. Tenebrionidae	Notiziario del Gruppo Entomologico Ligure
Col. Tenebrionidae	Notiziario del Gruppo Entomologico Ligure
Col. Tenebrionidae	Notiziario del Gruppo Entomologico Ligure
Coleotteri Tenebrionidi, Curculionidi e Pseudoscorpioni r	Notiziario del Gruppo Entomologico Ligure
Materiali per lo studio dei Tenebrionidi dell'Arcipelago	Lavori della Società italiana di Biogeografia
Ricerche sull'entomofauna della Riserva Naturale Vinch	Ministero dell'Agricoltura e delle Foreste, Coll
Elenco delle specie di Coleotteri trovate in Piemonte.	Annali della regia accademia di Agricoltura, T
Aggiunte inedite di Paolo Luigioni al catalogo "I coleotte	Bullettino della Società entomologica italiana
Nuovi reperti di Coleotteri nel biotopo di Policoro (Mater	Annali del Museo civico di Storia naturale di F
Indagine faunistica sui Coleotteri delle Isole Pelagie	Giornale Italiano di Entomologia
Risultati zoologici della missione inviata dalla R. Societ	Annali del Museo civico di Storia naturale di
Coleotteri dell'Africa Orientale Italiana. 10 contributo.	Atti del Museo civico di Storia naturale, Triest
Il problema delle specie a diffusione transadriatica con p	Memorie di Biogeografia adriatica
Ricerche zoologiche sul massiccio del Pollino (Lucania-(	Annuario dell'Istituto e Museo di Zoologia dell'
Materiali per lo studio dei Tenebrionidi italiani (Second	Atti del Museo civico di Storia naturale di Trie
in Porta A., Fauna Coleopterorum Italica	suppl. 2.
Raccolte faunistiche compiute nel Gargano da A. Ghigi e	Acta pontificiae academiae scientiarum
Materiali per lo studio dei Tenebrionidi italiani (Prima n	Atti del Museo civico di Storia naturale di Trie
Tenebrionidae. In: Zavattari E., Biogeografia delle Isole	Rendiconti dell'Accademia nazionale dei XL
Zur Kenntnis der Tenebrioniden aus Süditalien (Insecta:	Stuttgarter Beiträge zur Naturkunde
Tenebrionidae vom Maltesischen Archipel (Insecta: Cole	Stuttgarter Beiträge zur Naturkunde
Contributo alla conoscenza dei Coleotteri etnei	Bollettino della Associazione Romana di Ento
Contributo alla conoscenza della coleotterofauna del Tre	Bollettino della Associazione Romana di Ento
Elenco dei Coleotteri dell'Isola d'Elba, con studii sul pro	Memorie della Società entomologica italiana
Contributo alla migliore conoscenza di alcune forme ital	Monitore zoologico italiano
Notizie su due popolazioni insulari di Lacerta muralis e s	Annali del Museo civico di Storia naturale "G.
Su alcune popolazioni di Lacerta sicula Rafinesque dell	Annali del Museo civico di Storia naturale "G.
Asida argentierae n. sp. di Sardegna (Coleoptera Teneb	Bollettino della Società sarda di Scienze natur
Psammoardoinellus, nuovo genere di Opatrinae della S	Memorie della Società entomologica italiana
Un nuovo Stenohelops Reitt. Della Sardegna (Coleopter	Bollettino della Società entomologica italiana
Dati geonemici su alcuni Elopini italiani (Coleoptera, Te	Bollettino della Associazione Romana di Ento
Segnalazioni faunistiche italiane. 54. Dichillus minutus A	Bollettino della Società entomologica italiana
Le popolazioni italiane di Nalassus aemulus (Küster) e	Bollettino del Museo civico di Storia naturale
Segnalazioni faunistiche italiane. 159-160. Cnemeplati	Bollettino della Società entomologica italiana
Annotazioni sui Litoborini Antoine della Sardegna (Cole	Boll. Societat d'Història natural de les Balears

Nuovi dati sui Tenebrionidi delle isole toscane e descrizi	Atti del Museo di Storia naturale della Marem
Osservazioni su Dichillus corsicus e descrizione di tre	Annali del Museo civico di Storia naturale "G.
Observations on some Tenebrionidae (Coleoptera) from	Zootaxa
Gli Pseudoseriscius italiani del gruppo helvolus (Küster	Natura, Milano
Nalassus genei melonii ssp. nov., dell'Isola di San Pie	Doriana
Segnalazioni faunistiche italiane. 23-25. Menephilus cyli	Bollettino della Società entomologica italiana
Belopus (Centorus) proceroides n. sp. della Sardegna (	Bollettino della Società entomologica italiana
Due nuove Asida Latr. di Sardegna (Coleoptera Tenebri	Annali del Museo civico di Storia naturale "G.
Segnalazioni faunistiche italiane. 36-38-188. Allophylax	Bollettino della Società entomologica italiana
Eutagenia aegyptiaca tunisea Norm. nuova per la fauna	Bollettino della Società entomologica italiana
Segnalazioni faunistiche italiane. 186-188. Cossyphus ta	Bollettino della Società entomologica italiana
Discovery of natural hybridization in tenebrionid beetles	Entomological News
Tassonomia, geonemia ed ecologia delle razze di Pimel	Atti XI Congresso nazionale italiano di Entomo
Note geonemiche ed ecologiche sulle Phaleria Latr. di	Bollettino della Società sarda di Scienze natur
Le Asida italiane	Rivista coleotterologica italiana
Le Asida italiane	Rivista coleotterologica italiana
Specie e varietà nuove o rare di coleotteri italiani	Rivista coleotterologica italiana
Contributi alla conoscenza dell'artropodofauna dell'Isol	Bollettino della Società entomologica italiana
La coleotterofauna del tombolo antistante la Laguna di E	Bollettino della Associazione Romana di Ento
Coleotteri raccolti nelle inondazioni dell'Aniene dal 1889	Bollettino della Società romana di Storia zool.
Contributo allo studio della fauna coleotterica dl Lazio.	Atti della Pontificia Accademia romana dei Nu
Coleotteri raccolti nel Lazio dal Conte Flaminio Baudi di	Atti della Pontificia Accademia Romana dei Nu
Contributo allo studio della fauna entomologica italiana.	Annuario del Museo zoologico della regia Univ
Contributo allo studio della fauna entomologica italiana.	Atti della Pontificia Accademia Romana dei Nu
I Coleotteri d'Italia. Catalogo topografico, sinonimico e bi	Memorie della pontificia Accademia delle Scie
Terzo contributo alla conoscenza della fauna entomolog	Atti della Pontificia Accademia romana dei Nu
Quinto contributo alla conoscenza della fauna entomolo	Atti della Pontificia Accademia Romana dei Nu
Una nuova varietà dell'Akis italica Sol. (Coleoptera - F	Atti della Pontificia Accademia romana dei Nu
Sopra un caso di antenna bifida in un Akis bacarozzo S	Atti della Pontificia Accademia romana dei Nu
Coleotteri del Lazio non citati come tali nel "Catalogo de	Bullettino della Società entomologica italiana
Rapporto su di un'escursione nella Sardegna compiuta	Atti della Società italiana di Scienze naturali,
Su alcune teratologie nei Coleotteri. II	Bollettino della Associazione Romana di Ento
Esperienze preliminari sull'igropreferenza nei Coleotteri	Bollettino di Zoologia
Observations on the northernmost presence of xerother	Gortania, Atti del Museo friulano di Storia nat
Il bilancio idrico nei Coleotteri Tenebrionidi	Archivio zoologico italiano
Studi ecologici e faunistici sui Tenebrionidi della Puglia	Memorie di Biogeografia adriatica
Contributo alla zoogeografia dei Tenebrionidi della Sicili	Memorie della Società entomologica italiana
Contributo alla conoscenza dei Coleotteri Tenebrionidi d	Bollettino della Accademia Gioenia di Scienze
Contributo alla conoscenza dei Tenebrionidi delle Egadi	Bollettino della Società entomologica italiana
Contributo alla conoscenza dei Tenebrionidi delle Tremi	Bollettino della Società entomologica italiana
I Tenebrionidi (Col. Heteromera) delle Isole Eolie e di Us	Atti della Accademia Gioenia di Scienze natur
Ecologia dei Tenebrionidi (Insecta, Coleoptera) dell'ecos	C.N.R., Quaderni sulla "Struttura delle zoocen
Eterogeneità indotta dall'uomo in alcuni ambienti costieri	Atti della Società Italiana di Ecologia
Osservazioni ecologiche sui Coloetteri della Puglia a su	Thalassia Salentina
Il melanismo nel genere Phaleria Latreille (Insecta, Col	Bollettino dell'Accademia gioenia di Scienze n
Osservazioni ecologico-quantitative sui Tenebrionidi (Co	Atti del Museo di Storia naturale della Marem
Tenebrionidi conosciuti dal Friuli - Venezia Giulia ed ent	Gortania - Atti del Museo friulano di Storia nat
I Tenebrionidi (Coleoptera Heteromera) conservati press	Rivista del Museo di Scienze naturali di Berg
Osservazioni sulla distribuzione geografica e sull'ecol	Rivista del Museo di Scienze naturali di Berg
Tenebrionidi delle piccole isole italiane: Giglio e Giannut	Quaderni di ecologia animale
Ulteriori dati per una conoscenza della coleotterofauna	Quaderni di ecologia animale
III Contributo alla conoscenza delle forme larvali dei Te	Eos
Ricerche sui Coleotteri della Puglia raccolti da G. Marcu	Quaderni di ecologia animale
Nuovi dati faunistici sulla coleotterofauna pugliese	Atti dell'Istituto veneto di Scienze (Classe Sci.

Eutagenia elvirae, nuova specie di Coleottero Tenebrion	Memorie della Società entomologica italiana
Elenco delle specie più rare o non ancora segnalate per	Atti del I Convegno Friuliano di Scienze Natur
Note di morfologia e di biologia su Hoplocephala haemo	Entomologica, Bari
Contributo alla Conoscenza degli Insetti Coloetteri della	Pubblicazioni dell'Istituto di Entomologia dell'
Reperti: 47. Elenophorus collaris L. (Col. Tenebrionidae)	Bollettino dell'Associazione romana di Entomo
Descrizione di una notevole emimeria in Cantharis livida	Bollettino della Società entomologica italiana
Saggio di un Catalogo sui Coleotteri della Campagna	Lo Spallanzani
Segnalazioni faunistiche italiane. Helops coeruleus (Li	Bollettino della Società entomologica italiana
Note su alcuni Coleotteri rinvenuti sui Monti Lepini e ne	Quaderni del Museo di Storia naturale di Patr
Esperienze sull'orientamento solare di Phaleria provincia	Atti della Accademia delle Scienze di Torino
I Coleotteri dell'abete rosso Picea abies (L.) Karst. In Va	Rivista piemontese di Storia naturale
Coleotteri. Tenebrionidae In: Furlanetto, D. Atlante della	Nodo libri
Helopinae di Sardegna: zoogeografia e filogenesi (Col.	Lavori della Società italiana di Biogeografia
Le categorie ecologiche degli Opatrinae di Sardegna (C	Rendiconti Seminario Facoltà Scienze, Univer
Materiali per un elenco dei Coleotteri dell'Isola di Monte	Lavori della Società italiana di Biogeografia
Coleotteri rinvenuti sul versante marino del Capo di Cap	Annali del Museo civico di Storia naturale "G.
Note di caccia. V. Reperti di specie italiane rare o poco	Bollettino della Società entomologica italiana
Fauna Coleopterorum Italica, vol. IV, Heteromera-Phytophaga. LXXV Fam. Tenebrionidae; Piacenza	
Fauna Coleopterorum Italica, Supplementum 2; Sanremo	
Fauna Coleopterorum Italica, Supplementum 3; Sanremo	
Breve escursione entomologica fatta sulle Madonie e ne	Bullettino della Società entomologica italiana
Calendario coleotterologico per Palermo e dintorni	Bullettino della Società entomologica italiana
Catalogo dei Coleotteri di Sicilia.	Naturalista Siciliano
Due giorni di caccia sulle Madonie	Naturalista Siciliano
Coleotteri nuovi o poco conosciuti della Sicilia	Bullettino della Società entomologica italiana
Reperti 25-27.- Akis subterranea Sol., Tentyria laevigata	Bollettino dell'Associazione romana di Entomo
Reperti. 30.-Tentyria grossa grossa Sol., Opatroides pu	Bollettino dell'Associazione romana di Entomo
Su alcuni Tenebrionidi dell'Isola Levanzo (Col. Heterom	Bollettino dell'Associazione romana di Entomo
Annotazioni sugli Oochrotus italiani (Coleoptera, Hetero	Bollettino del Museo civico di Storia naturale
La coleotterofauna delle ferite di Quercus robur L. nelle	Atti Convegno Ecologia Prealpi Orientali e Gr
Ricerche faunistiche del Museo civico di Storia naturale	Bollettino del Museo civico di Storia naturale
Coleotteri dell'Isola d'Elba e di Pianosa	Rivista coleotterologica italiana
Contributi alla conoscenza faunistica delle isole toscane	Atti della Società toscana di Scienze naturali
Contributi alla conoscenza faunistica delle isole toscane	Atti della Società toscana di Scienze naturali
Bestimmungstabelle der Cossyphini und Misolampini.	Wiener Entomologische Zeitung
Effetti insulari nei Polmonati conchigliati e nei Prosobran	Annali del Museo civico di Storia naturale "G.
La ricollocazione dell'alimento di Pimelia grossa F. (Col	Naturalista siciliano
Appunti Coleotterologici. XVIII.	Rivista di Scienze Naturali "Natura"
Revision der wespaläarktischer Tenebrionidae (Coleopte	Stuttgarter Beiträge zur Naturkunde
Taxonomie und Faunistik von Stenomax aeneus Scopol	Entomologische Nachrichten und Berichte
Taxonomie und Faunistik von Eledona agricola (Herbst 1	Entomol. Z.
Neatus picipes in der Palaearktische Arten-Gruppe (Col	Annales historico-naturales Musei nationalis h
Phaleria ligurica n. sp. nebst Bemerkungen über Ph. Re	Wiener Entomologische Zeitung
Zwei neue Tenebrioniden aus dem Mittelmeergebiete	Bollettino della Società entomologica italiana
I Tenebrionidi della regione veronese (Coleoptera)	Bollettino del Museo civico di Storia naturale
Tenebrionidae: In: Cerretti P., Tagliapietra A., Tisato, M	Centro Nazionale per lo Studio e la Conserva
Diaclina fagi (Panzer, 1799) nuova per la fauna italiana	Bollettino del Museo civico di Storia naturale
Revisione della tribù Cossyphini Latreille, 1802. Parte	Atti del Museo civico di Storia naturale di Trie
A proposito di Ulomina carinata Baudi di Selve, 1876 (C	Bollettino del Museo regionale di Scienze Nat
Tenebrionidae: In: Mason F., Cerretti P., Tagliapietra A	Centro Nazionale per lo Studio e la Conserva
Revision of French and Corsican species of the genus A	Annales Zoologici
A propos d'Oochrotus unicolor Lucas, 1852 (Coleoptera	Bulletin de la Société linnéenne de Bordeaux
Asida (Polasida) poneli n.sp., nouvelle espèce de la Riv	Bulletin del la Socité Linneane de Bordeaux
Les Dichillus corso-sardes: taxonomie et géonémie (Col	Bulletin de la Societé entomologique de Fran

Nuovi Coleotteri di Sicilia (Coleoptera Carabidae et Ten	Naturalista Siciliano
geological and environmental relations	Biological Journal of the Linnean Society
Deep genetic divergence in the darkling beetle Pimelia r	Journal of Zoological Systematics and Evolut
Reperti. Iphthimus italicus Truqui	Bollettino dell'Associazione romana di Entomo
Ricerche ecologiche sugli Artropodi di una spiaggia sabl	Redia
Reperti. 24. Cylindronotus (Odocnemis) exaratus Germ.	Bollettino della Associazione Romana di Ento
Catalogo dei Coleotteri della Lombardia	Notizie naturalie civili sulal Lomabrdia, vol. ., (
Revisión del género Phylan Stephens, 1857 (Coleoptera	Argania Editio, Barcelona
Repertorio sistematico e topografico della flora e fauna	Memorie fuori serie del Museo civico di Storia
Note sulla fauna delle cavità artificiali di Roma	Notiziario del Circolo speleologico romano
Zur Kenntnis der Arten-Gruppe Akis spinosa Linnaeus, (	Senckenbergiana bilogica

Pagination
35: 727-744.
6: 33-48.
26: 17-18.
44: 83
(4)16(suppl.): 13-28.
(4)17: 111-125.
(4)19(suppl.):543-548.
(4)19: 131.
(4)24:103-144.
(4)20:281-304.
5(1997/98): 49-84
105: 59-68.
a, Carmagnola, Italy
30: 293-314.
133: 473-487.
425-443.
21: 37-125
11/12:5-84.
70: 171-254.
135 pp
11:7-37.
5 (1997-98): 85-100.
10: 545-604.
2: 239-241.
(n. s.)9: 257-307.
3 (2): 6-7.
51: 95-105.
3: 1-3.
6: 89-115.
6: 183-200, 275-301.
7: 1-20.
7: 3-36, 91-106, 137-165, 209-237.
8: 316-322.
8: 98-119, 199-207, 259-268.
9: 25-54, 93-152.
14: 63-91.
26:77-92.
30: 21-28.
za" (unpublished)
7: 1-7.
74: 213-222.
87: 87-90.
1: 28-31.
20-21: 223-231.
27(1-2): 9-25.
228 pp.
104: 441-445.
za" (unpublished)
89: 145-148.
92: 144-147.
94: 129-131.
17: 57-68.

12: 9-18.
4: 227-285.
6: 85-86.
9: 3-4.
90: 43-45.
90: 50-54.
90: 162-164.
13: 1-21.
91: 24-27.
91: 57-61.
91: 19-20.
92: 35-36.
92: 13-16.
23: 49-50.
47: 117-167.
18: 91-108.
99-101(1/2): 44.
24: 67-68.
18: 55-89.
Quaderno 1: 22-23, figg. 12-13.
104: 87-88.
113: 288-296.
22/23: 187-191.
36(1985): 7-62.
15: 111-126.
68: 207-214.
23: 71-80.
58: 293-306.
20(2): 23-26.
118: 117-118.
129(2): 119-139.
29: 145-224.
4 (4-5): 108-114
1:313-321.
73: 307-345.
55: 127-141.
6: 231-246.
74: 87-109.
28: 215-230.
28: 249-265.
46 (1990): 131-182
1 (11): 1-2.
1:37-68.
2: 56-64.
99: 248-251.
24: 133-137
19: 177-179.
26 (1983): 155-170.
lished)
64: 215-222.
7: 129-198.
54: 1-282.
3: 1-561.
55: 27-43.

11 (Ser. Entomol. 12): 1-89.
25(3/4): 199-239.
29(2/4): 123-142.
21: 167-177.
23: 69-79.
44: 243-256.
37: 45-64
9: 1-52.
33: 97-118.
56: 245-300.
10: 171-173.
23: 103-126.
99-105.
60 (1-4) (2005): 47-104.
221-243.
165-297.
22-26.
56: I.
133(1): 43-53.
56 /2: 165-170.
277: 291-301.
106: 415-423.
36:1100-1110.
37: 2158–2168.
19:3413–3429 .
9:1237–1250 .
144: 370–375.
37: 1061-1070.
14:367–378 .
108: 659-672.
38: 1318-1329.
4: 307-315.
12: 61-66.
59 (1-4)(2004): 23-41.
132 (3): 205-217.
70:139-142.
31 (1): 57-69.
23: 81-102.
48: 69-153.
43: 153-172
52: 383-401.
26(4): 62.
1(12): 221-227.
18(9/10): 1-12.
77: 45-46.
60: 1-111.
48: 402-416.
102(9/10): 156-162.
51: 19-37.
107: 565-588.
29: 8-52.

42: 16-94
121(1): 63.

4 (3-4): 5-6
8 (2): 1
12 (1): 10
6: 73-77.
113(1/3): 50.
21 (1-4): 7.
6: 204-206.
368: 1-12.
3 (3): 2-4
3 (4): without pagination
23(3): 71.
7 (2): 4
6 (3): 3-4
7 (2): 8
7 (2): 8
8 (1): 7-10
104(9-10): 211-217.
10 (1): 7-8
10 (1): 8-9
11 (2): 8
11 (3): 14
11 (3): 8
11 (1): 4
(n.s.)5(1974): 637-723.
ana Verde, 86, Roma
3: 1-616.
105: 29-35.
5: 41-50.
11: -127-143
54: 1-479.
14(15): 207-242.
1: 7-299.
8(9): 1-6.
27: 187-256.
: 270-272.
13(13): 145-196.
22: 25-43.
(4)11: 392-399
(A)379: 1-32.
(A)392: 1-17.
16(2): 9-14.
24(2): 47-54.
2: 77-175.
63: 259-284.
70: 305-322.
77: 671-693.
19(1979): 189-192.
59: 34-36.
112: 36-39.
36(1981): 45-48.
117(1-3): 64.
12(1985): 141-148.
121: 233-234.
37: 133-142.

17: 73-77.
99:603-627.
2318:400-420.
73: 97-107.
5(246): 1-4.
114: 118-119.
116: 165-167.
85: 73-77.
116:58.
123: 39-42.
123: 73-74.
113: 113-120.
79-83.
23: 133-136.
7: 142-189.
8: 9-16, 40-55, 69-95.
9:1-10.
132(2): 157-174.
47(1992): 7-34.
1: 183-194.
73: 1-29.
76(2): 78-85.
(n.s.)5(6): 1-8.
78: 47-49.
(2)13: 1-1160.
84(3): 120-180.
87: 3-35.
74: 61.
74 (4): 1-3.
42: 43-93.
21: 451-463.
21: 5-7.
19:129-144.
23: 169-185.
45: 281-324.
5: 1-78.
48: 499-518
(4)10: 406-428.
102: 87-91.
102: 35-38.
(7)2: 161-184.
3. Ambienti mediterranei. I. Le coste sabbiose: 121-152.
13: 21-24.
20: 29-47.
29(351): 9-13.
16: 47-69.
20: 173-213.
9: 117-125.
9: 103-116.
26: 5-11.
24: 5-21.
54(1978): 167-206.
9: 1-186.
139: 59-79.

60: 235-238.
439-464
10: 9-30.
18: 1-58.
24:30.
98: 146-149.
(2)14: 103-110.
130(1): 79.
6: 99-117.
92: 1-8.
18: 135-160.
240-241.
8(1980): 691-714.
54(suppl.): 231-244.
(n.s.)5(1974): 609-635.
81: 340-355.
115: 156-160.

3: 366-380.
6: 302-312.
2: 105-130.
20: 129-134
53: 85-100.
24: 82.
24: 105.
25: 75-76.
25: 61-63.
295-325.
35(1984): 7-41.
4: 111-115.
31: 196-221.
33: 96-122.
36: 129-150.
71(1959): 349-416.
(4)24: 97-102.
40: 27-29.
403:1-21.
39: 205-208.
108(12): 501-503.
87: 67-73.
21: 10-12.
62: 65-67.
9: 91-120.
113
24(Bot. Zool.): 219-222.
48: 185-249.
19: 185-189.
93
55: 335-373.
28(4): 181-187.
29: 103-108.
107: 165-168.

31: 249-259
104: 29–37
49(3): 196–203
23(3): 71.
51: 1-19.
24(2): 55.
Giuseppe Bernardoni di Giovanni, Milano

1: XXXIII+2176 pp.
25: 27-57.
38: 41-59.

Species name
Accanthopus velikensis
Akis bacarozzo
Akis italica
Allophylax picipes picipes
Ammobius rufus
Asida bayardi
Asida luigionii luigionii
Asida pirazzolii pirazzolii
Asida pirazzolii sardiniensis
Asida sabulosa
Blaps gibba
Blaps gigas
Blaps lethifera lethifera
Blaps mucronata
Bolitophagus interruptus
Bolitophagus reticulatus
Catomus rotundicollis
Cheirodes sardous
Cnemeplatia atropos atropos
Colpotus strigosus ganglbaueri
Colpotus strigosus strigosus
Corticeus bicolor
Corticeus fasciatus
Corticeus pini
Corticeus unicolor
Cossyphus tauricus
Crypticus gibbulus
Crypticus quisquilius aprutianus
Dendarus coarticollis
Dendarus lugens
Diaclina testudinea
Diaperis boleti
Dichillus minutus
Eledona agricola
Erodius siculus neapolitanus
Gonocephalum assimile
Gonocephalum granulatum nigrum
Gonocephalum obscurum obscurum
Gonocephalum rusticum
Gunarus parvulus
Halammobia pellucida
Helops caeruleus
Helops rossii
Iphthiminus italicus italicus
Leichenum pulchellum pulchellum
Leptoderis collaris
Lyphia tetraphylla
Melanimon tibialis
Menephilus cylindricus cylindricus
Nalassus aemulus aemulus
Nalassus assimilis
Nalassus dryadophilus
Nalassus planipennis

Neatus noctivagus
Neomida haemorrhoidalis
Oochrotus unicolor ardoini
Opatrum sabulosum sabulosum
Opatrum sabulosum sculptum
Pachychila frioli
Palorus depressus
Pedinus meridianus
Pentaphyllus chrysomeloides
Pentaphyllus testaceus
Phaleria acuminata
Phaleria bimaculata bimaculata
Phaleria provincialis ghidinii
Phtora crenata
Phylan italicus
Pimelia bipunctata
Platydema europaea
Platydema violacea
Pseudoseriscius normandi pacificii
Scaphidema metallica
Scaurus striatus
Stenomax lanipes
Stenosis brenthoides brenthoides
Stenosis intermedia
Stenosis sardoa
Tentyria grossa grossa
Tentyria italica
Trachyscelis aphodioides
Uloma culinaris
Xanthomus pallidus
Xanthomus pellucidus

Photo at

http://www.biolib.cz/en/taxonimage/id165589/
http://www.biolib.cz/en/taxonimage/id193590/?taxonid=55558
http://www.tenebrionidae.net/simplephpgallery3/sp_index.php?file=./A-B/Akis%20italica%20solier,%201836.jpg
http://www.naturamediterraneo.com/Public/data6/Velvet%20ant/Allophylax%20picipes,%20Sferracavallo,%2030%20genn
http://r.a.r.e.free.fr/interactif/photos%20tenebrionidae/rufus.jpg
http://www.entomologiitaliani.net/public/forum/phpBB3/viewtopic.php?f=178&t=21036&hilit=bayardi
http://www.entomologiitaliani.net/public/forum/phpBB3/viewtopic.php?f=178&t=34383&hilit=Asida
http://www.entomologiitaliani.net/public/forum/phpBB3/viewtopic.php?f=178&t=39510&hilit=Asida

http://www.kerbtier.de/cgi-bin/enFSearch.cgi?Fam=Tenebrionidae
http://www.galerie-insecte.org/galerie/blaps_gibba.html
http://r.a.r.e.free.fr/interactif/photos%20tenebrionidae/gigas.jpg
http://r.a.r.e.free.fr/interactif/photos%20tenebrionidae/lethifera.jpg
http://r.a.r.e.free.fr/interactif/photos%20tenebrionidae/mucronata.jpg
http://www.zin.ru/animalia/coleoptera/eng/bolintkm.htm
http://r.a.r.e.free.fr/interactif/photos%20tenebrionidae/reticulatus.jpg
http://www.entomologiitaliani.net/public/forum/phpBB3/viewtopic.php?f=178&t=31528&hilit=rotundicollis
http://www.entomologiitaliani.net/public/forum/phpBB3/viewtopic.php?f=178&t=40755&hilit=Cheirodes
http://www.entomologiitaliani.net/public/forum/phpBB3/viewtopic.php?f=178&t=16045&hilit=Cnemeplatia

http://www.entomologiitaliani.net/public/forum/phpBB3/viewtopic.php?f=178&t=35121&hilit=strigosus
http://r.a.r.e.free.fr/interactif/photos%20tenebrionidae/bicolor.jpg
http://r.a.r.e.free.fr/interactif/photos%20tenebrionidae/fasciatus.jpg
http://r.a.r.e.free.fr/interactif/photos%20tenebrionidae/pini.jpg
http://r.a.r.e.free.fr/interactif/photos%20tenebrionidae/unicolor%20(hypo.).jpg
http://www.entomologiitaliani.net/public/forum/phpBB3/viewtopic.php?f=178&t=29989&hilit=tauricus
http://www.entomologiitaliani.net/public/forum/phpBB3/viewtopic.php?f=178&t=41460&hilit=gibbulus
http://www.entomologiitaliani.net/public/forum/phpBB3/viewtopic.php?f=178&t=30275&hilit=aprutianus
http://www.entomologiitaliani.net/public/forum/phpBB3/viewtopic.php?f=178&t=41251&hilit=Dendarus
http://www.entomologiitaliani.net/public/forum/phpBB3/viewtopic.php?f=178&t=20508&hilit=Dendarus+lugens
http://www.entomologiitaliani.net/public/forum/phpBB3/viewtopic.php?f=178&t=39134&hilit=Diaclina
http://r.a.r.e.free.fr/interactif/photos%20tenebrionidae/boleti.jpg
http://r.a.r.e.free.fr/interactif/photos%20tenebrionidae/minutus.jpg
http://r.a.r.e.free.fr/interactif/photos%20tenebrionidae/agaricola.jpg
http://www.entomologiitaliani.net/public/forum/phpBB3/viewtopic.php?f=178&t=1570&hilit=Erodius+siculus

http://r.a.r.e.free.fr/interactif/photos%20tenebrionidae/granulatum%20nigrum.jpg
http://www.entomologiitaliani.net/public/forum/phpBB3/viewtopic.php?f=178&t=41458&hilit=Gonocephalum
http://r.a.r.e.free.fr/interactif/photos%20tenebrionidae/rusticum.jpg
http://www.entomologiitaliani.net/public/forum/phpBB3/viewtopic.php?f=178&t=30521&hilit=parvulus
http://r.a.r.e.free.fr/interactif/photos%20tenebrionidae/pellucida.jpg
http://r.a.r.e.free.fr/interactif/photos%20tenebrionidae/coeruleus.jpg
http://www.entomologiitaliani.net/public/forum/phpBB3/viewtopic.php?f=178&t=33335&hilit=Helops
http://www.entomologiitaliani.net/public/forum/phpBB3/viewtopic.php?f=178&t=40472&hilit=Iphthiminus
http://r.a.r.e.free.fr/interactif/photos%20tenebrionidae/pulchellum.jpg
http://r.a.r.e.free.fr/interactif/photos%20tenebrionidae/collaris.jpg
http://www.entomologiitaliani.net/public/forum/phpBB3/viewtopic.php?f=178&t=37450&hilit=tetraphylla
http://r.a.r.e.free.fr/interactif/photos%20tenebrionidae/tibiale.jpg
http://www.entomologiitaliani.net/public/forum/phpBB3/viewtopic.php?f=178&t=24513&hilit=menephilus
http://www.entomologiitaliani.net/public/forum/phpBB3/viewtopic.php?f=178&t=18090&hilit=aemulus
http://r.a.r.e.free.fr/interactif/photos%20tenebrionidae/assimilis.jpg
http://r.a.r.e.free.fr/interactif/photos%20tenebrionidae/dryadophilus.jpg
http://www.entomologiitaliani.net/public/forum/phpBB3/viewtopic.php?f=11&t=42786&hilit=planipennis

http://www.entomologiitaliani.net/public/forum/phpBB3/viewtopic.php?f=178&t=29668&hilit=noctivagus
http://www.kerbtier.de/cgi-bin/enFSearch.cgi?Fam=Tenebrionidae
http://www.entomologiitaliani.net/public/forum/phpBB3/viewtopic.php?f=178&t=6485&hilit=Oochrotus
http://www.kerbtier.de/cgi-bin/enFSearch.cgi?Fam=Tenebrionidae
http://r.a.r.e.free.fr/interactif/photos%20tenebrionidae/sabulosum.jpg
http://www.entomologiitaliani.net/public/forum/phpBB3/viewtopic.php?f=178&t=9300&hilit=Pachychila+frioli
http://r.a.r.e.free.fr/interactif/photos%20tenebrionidae/depressus.jpg
http://www.entomologiitaliani.net/public/forum/phpBB3/viewtopic.php?f=178&t=24580&hilit=Pedinus+meridianus
http://www.entomologiitaliani.net/public/forum/phpBB3/viewtopic.php?f=178&t=6635&hilit=chrysomeloides
http://r.a.r.e.free.fr/interactif/photos%20tenebrionidae/testaceus.jpg
http://www.entomologiitaliani.net/public/forum/phpBB3/viewtopic.php?f=178&t=24316&hilit=Phaleria+acuminata
http://www.entomologiitaliani.net/public/forum/phpBB3/viewtopic.php?f=178&t=25793&hilit=Phaleria+bimaculata
http://www.entomologiitaliani.net/public/forum/phpBB3/viewtopic.php?f=178&t=35773&hilit=Phaleria+provincialis
http://www.entomologiitaliani.net/public/forum/phpBB3/viewtopic.php?f=178&t=14427&hilit=Phtora+crenata
http://www.entomologiitaliani.net/public/forum/phpBB3/viewtopic.php?f=178&t=18554&hilit=Phylan+italicus
http://www.entomologiitaliani.net/public/forum/phpBB3/viewtopic.php?f=178&t=24414&hilit=Pimelia+bipunctata
http://www.entomologiitaliani.net/public/forum/phpBB3/viewtopic.php?f=178&t=19209&hilit=Platydema+europaeum
http://r.a.r.e.free.fr/interactif/photos%20tenebrionidae/violaceum.jpg

http://r.a.r.e.free.fr/interactif/photos%20tenebrionidae/metallicum.jpg
http://r.a.r.e.free.fr/interactif/photos%20tenebrionidae/striatus.jpg
http://www.entomologiitaliani.net/public/forum/phpBB3/viewtopic.php?f=178&t=7159&hilit=Stenomax
http://www.entomologiitaliani.net/public/forum/phpBB3/viewtopic.php?f=178&t=38154&hilit=Stenosis+brenthoides+brentho
http://r.a.r.e.free.fr/interactif/photos%20tenebrionidae/intermedia.jpg
http://r.a.r.e.free.fr/interactif/photos%20tenebrionidae/sardoa.jpg
http://www.entomologiitaliani.net/public/forum/phpBB3/viewtopic.php?f=178&t=19262&hilit=Tentyria+grossa+grossa
http://www.entomologiitaliani.net/public/forum/phpBB3/viewtopic.php?f=178&t=41016&hilit=Tentyria+italica
http://r.a.r.e.free.fr/interactif/photos%20tenebrionidae/aphodioides.jpg
http://r.a.r.e.free.fr/interactif/photos%20tenebrionidae/culinaris.jpg
http://www.entomologiitaliani.net/public/forum/phpBB3/viewtopic.php?f=178&t=30091&hilit=Xanthomus+pallidus
http://www.entomologiitaliani.net/public/forum/phpBB3/viewtopic.php?f=178&t=30075&hilit=pellucidus
